# Genetic dissection of metabolite variation in Arabidopsis seeds: evidence for mQTL hotspots and a master regulatory locus of seed metabolism

**DOI:** 10.1093/jxb/erx049

**Published:** 2017-03-06

**Authors:** Dominic Knoch, David Riewe, Rhonda Christiane Meyer, Anastassia Boudichevskaia, Renate Schmidt, Thomas Altmann

**Affiliations:** 1 Department of Molecular Genetics/Heterosis, Leibniz Institute of Plant Genetics and Crop Plant Research (IPK) Gatersleben, D-06466 Seeland/OT Gatersleben, Germany; 2 Department of Breeding Research/Genome Plasticity, Leibniz Institute of Plant Genetics and Crop Plant Research (IPK) Gatersleben, D-06466 Seeland/OT Gatersleben, Germany

**Keywords:** *Arabidopsis thaliana*, gas chromatography–mass spectrometry, metabolic quantitative trait locus, primary metabolism, recombinant inbred line, seed biology.

## Abstract

To gain insight into genetic factors controlling seed metabolic composition and its relationship to major seed properties, an Arabidopsis recombinant inbred line (RIL) population, derived from accessions Col-0 and C24, was studied using an MS-based metabolic profiling approach. Relative intensities of 311 polar primary metabolites were used to identify associated genomic loci and to elucidate their interactions by quantitative trait locus (QTL) mapping. A total of 786 metabolic QTLs (mQTLs) were unequally distributed across the genome, forming several hotspots. For the branched-chain amino acid leucine, mQTLs and candidate genes were elucidated in detail. Correlation studies displayed links between metabolite levels, seed protein content, and seed weight. Principal component analysis revealed a clustering of samples, with PC1 mapping to a region on the short arm of chromosome IV. The overlap of this region with mQTL hotspots indicates the presence of a potential master regulatory locus of seed metabolism. As a result of database queries, a series of candidate regulatory genes, including *bZIP10*, were identified within this region. Depending on the search conditions, metabolic pathway-derived candidate genes for 40–61% of tested mQTLs could be determined, providing an extensive basis for further identification and characterization of hitherto unknown genes causal for natural variation of Arabidopsis seed metabolism.

## Highlight

A comprehensive metabolic study of Arabidopsis seeds revealed mQTL hotspots, candidate genes, epistatic interactions, and a potential master regulatory locus of metabolism on the short arm of chromosome IV.

## Introduction

Natural variation is often the result of the contribution of multiple genes, detectable as quantitative trait loci (QTLs), and their interactions (Alonso-Blanco and Koornneef, 2000; Mackay, 2001; Remington and Purugganan, 2003; Koornneef *et al.*, 2004; Shindo *et al.*, 2007). However, it is often poorly understood to what extent the observed variation is attributed to genetic factors (Keurentjes *et al.*, 2006). Linkage and association mapping represent powerful, complementary approaches to connect genetic markers and phenotypic variation (Mackay, 2009). With recent advances in the field of ‘-omics’ sciences, mapping of molecular phenotypes such as transcript (West *et al.*, 2007; Keurentjes *et al.*, 2007) or metabolite levels (Lisec *et al.*, 2008; Toubiana *et al.*, 2012; Alseekh *et al.*, 2015) is increasingly utilized, opening up new possibilities for understanding complex molecular processes.

Recent developments of metabolomic platforms allow parallel and rapid quantification of hundreds to thousands of metabolites, offering new opportunities to study plant metabolomes (Alonso *et al.*, 2015; Soltis and Kliebenstein, 2015). One of the most frequently used metabolic approaches in plant biology is GC-MS (Fiehn, 2008). This technique allows quantification of a broad spectrum of primary metabolites including amino acids, organic acids, and sugars that can be utilized to map metabolic QTLs (mQTLs), as shown in maize (Riedelsheimer *et al.*, 2012), tomato (Schauer *et al.*, 2006, 2008), potato (Carreno-Quintero *et al.*, 2012), and rice (Matsuda *et al.*, 2012; Ying *et al.*, 2012; Li *et al.*, 2016). Based on mQTL mapping, genes encoding enzymes involved in specific biochemical pathways were identified (Saito and Matsuda, 2010; Chan *et al.*, 2011; Angelovici *et al.*, 2013; Li *et al.*, 2014; Strauch *et al.*, 2015; Francisco *et al.*, 2016) and systems for the regulation of metabolic networks elucidated (Fernie and Schauer, 2009; Wen *et al.*, 2015, 2016).

mQTL studies in the model organism *Arabidopsis thaliana* (Keurentjes *et al.*, 2006, 2008; Lisec *et al.*, 2008, 2009; Rowe *et al.*, 2008) provided the basis to identify genes and polymorphisms causal for natural trait variation (Brotman *et al.*, 2011; Riewe *et al.*, 2016), to characterize hitherto uncharacterized metabolic pathways and networks (Wentzell *et al.*, 2007; Chan *et al.*, 2010; Joseph *et al.*, 2014), and to unravel key regulators of metabolism (Li and Kliebenstein, 2014; Wu *et al.*, 2016). Arabidopsis seed mQTLs were studied for glucosinolates (Kliebenstein *et al.*, 2001), flavonoids (Routaboul *et al.*, 2012), oil content and fatty acids (Hobbs *et al.*, 2004; Sanyal and Randal Linder, 2012), carbohydrates (Calenge *et al.*, 2006), and branched-chain amino acids (BCAAs) (Angelovici *et al.*, 2013). A recent study focused on natural variation for oil, protein, carbon, and nitrogen content by near-infrared spectroscopy (Jasinski *et al.*, 2016). Comprehensive investigations of the genetic basis of the Arabidopsis seed primary metabolome, addressing a broader range of metabolite classes, have rarely been performed (Joosen *et al.*, 2013).

Recombinant inbred line (RIL) populations represent an important genetic resource for the investigation of natural variation in Arabidopsis (Mackay, 2001). A large set of reciprocal RILs was created by crossing the Arabidopsis accessions Col-0 and C24 (Törjék *et al.*, 2006). This RIL population was successfully utilized in several mapping studies, resulting in the identification of genomic regions involved in biomass heterosis at early developmental stages (Meyer *et al.*, 2010), and metabolic and biomass QTLs (Lisec *et al.*, 2008, 2009).

We used this population to address the following questions. (i) Are seed metabolite levels, seed weight, and seed protein content correlated? (ii) Which genomic regions are associated with particular metabolites in mature Arabidopsis seeds and do these loci interact? (iii) Which genomic regions contain genes encoding enzymes involved in pathway reactions related to these metabolites? (iv) Are there common genetic factors affecting multiple metabolites and thus acting as potential master regulators affecting the metabolic composition of mature seeds and what are the most promising candidate regulatory genes?

## Materials and methods

### Plant material and growing conditions

The mapping population consisted of 393 RILs originating from the reciprocal crosses Col-0×C24 (*n*=202) and C24×Col-0 (*n*=191) described by Törjék *et al.* (2006). F_2_ plants were propagated by self-pollination using single-seed descent to the F_10_ generation. Plants were cultivated in two consecutive experiments in a phytotron under controlled conditions [16 h sodium lamp light (250 µmol m^−2^ s^−1^); 20 °C; 70% relative humidity/8 h dark; 16 °C; 60% relative humidity] to ensure a constant environment during seed formation. A randomized block design and randomized positions within each block were used. Each line was replicated four times (individual plants). A total of 15–30 seeds per genotype were sown into 6 cm pots filled with Substrate 1 (Klasmann-Deilmann GmbH, Geeste, Germany). Stratification was performed for 3 d at 4 °C in the dark. After 1 week, well-developed and healthy plants were transferred into single pots. The orientation of trays was changed daily; every second day positions of trays in the room were shuffled to minimize position effects. Plants were treated with Novo Nem^®^ F (ÖRE Bio-Protect Biologischer Pflanzenschutz GmbH, Schwentinental, Germany) every second week. Mature seeds were collected using ARACONs (BETATECH, Gent, Belgium), purified, and stored in sealed screw-cap glass vials at 5 °C and 55% relative humidity.

### Fractionated metabolite and protein extraction

Twenty seeds of each of three individual plants per RIL were pooled. Polar metabolites and seed proteins were extracted via liquid–liquid extraction, modifying existing protocols (Lisec *et al.*, 2006; Erban *et al.*, 2007; Giavalisco *et al.*, 2011) using a liquid handling system (Biomek^®^ FX^P^, Beckman Coulter GmbH, Krefeld, Germany). Seed material was deep frozen at –80 °C and homogenized twice for 1 min at 30 Hz using a mixer mill (Retsch GmbH, Haan, Germany). Metabolites were extracted in 0.5 ml of chilled extraction buffer 1 (MTBE:MeOH:H_2_O; 3:1:0.5 plus internal standard 1:1000) by shaking and ultrasonication for 15 min at 4 °C. After adding 325 μl of extraction buffer 2 (MeOH:H_2_O; 1:3), samples were centrifuged and two 80 µl aliquots of the upper organic phase were stored for potential further investigation. Subsequently, 150 μl of chloroform were added, followed by shaking and centrifugation for 10 min, leading to a phase inversion. Three 120 μl aliquots of the upper polar phase were transferred into glass vials (CZT Klaus Trott, Kriftel, Germany), dried in a vacuum concentrator, filled with argon, capped, and stored in sealed plastic bags containing silica desiccant at –80 °C. The pellet containing proteins was dried as mentioned above.

### Seed protein quantification

Seed protein quantification was performed in 384-well plates using the Bio-Rad Protein Assay (Bio-Rad Laboratories GmbH, Munich, Germany) in three replicates, according to the manufacturer’s instructions. Protein pellets were washed five times with 800 µl of 70% ethanol, dissolved in 800 μl of 60 mM NaOH by shaking for 60 min at 70 °C, and centrifuged for 10 min. The total protein concentration of the supernatant was quantified at 595 nm using an Infinite 200 PRO microplate reader (Tecan Deutschland GmbH, Crailsheim, Germany) and a standard curve of 0–2.5 μg of BSA.

### Relative quantification of polar metabolites using GC-MS

Aliquots of the polar phases were in-line derivatized directly prior to injection (Erban *et al.*, 2007) and analyzed using a Gerstel MPS2-XL autosampler (Gerstel, Mühlheim, Germany) and an Agilent 7890 gas chromatograph (Agilent, Santa Clara, CA, USA) coupled to a LECO time-of-flight mass spectrometer (LECO, St. Joseph, MI, USA) (Riewe *et al.*, 2012). Metabolites were identified and assigned using LECO ChromaTOF software including the Statistical Compare package and electron impact spectra library provided by the Golm Metabolome Database (GMD, gmd.mpimp-golm.mpg.de). Extraction of quantitative data was performed using the R-package ‘TargetSearch’ (Cuadros-Inostroza *et al.*, 2009).

### Raw data processing and normalization

Metabolite intensities were normalized for seed weight, internal standard (l-alanine-2,3,3,3-d_4_, 98 atom% D, Isotec Inc., Miamisburg, OH, USA), and individual detector response to correct for potential extraction batch and measurement day effects. Outliers were removed (median ±4× SD), and metabolite data were power transformed to ensure a proximate normal distribution (Box and Cox, 1964). Protein concentrations were batch normalized and seed protein content calculated using the number of seeds, sample, and total buffer volume. The normalized, outlier-corrected, and transformed metabolite data and protein content are summarized in Supplementary Data S1 at *JXB* online.

### Statistical analyses

Statistical analyses were performed in R (R Core Team, 2015). Pearson correlation *P*-values were multiple testing corrected using false discovery rate (FDR) adjustment (Benjamini and Hochberg, 1995). Principal component analysis (PCA) was performed using the ‘pcaMethods’ package (Stacklies *et al.*, 2007) on centered and Pareto-scaled data.

### Molecular markers and linkage map

The RIL population was initially genotyped at the F_7_ generation with 110 single nucleotide polymorphism (SNP) markers (Törjék *et al.*, 2003, 2006). Further genotyping was performed at the F_9_ generation using 28 additional simple sequence repeat (SSR) markers (Supplementary Data S2). Markers were derived from previous studies (Loudet *et al.*, 2002; Salathia *et al.*, 2007; Andreuzza *et al.*, 2010; Hou *et al.*, 2010), and partially modified. The marker distributions of the reciprocal subpopulations were compared using a Mantel test (10 000 permutations) of the corresponding similarity matrices obtained by simple matching. The 138 markers were evenly distributed over the five chromosomes, with an average marker distance of 3.4 cM corresponding to ~1 Mbp (Supplementary Data S3).

### Analysis of quantitative trait loci

Power-transformed metabolite data, total protein content, and PCA scores were used to map QTLs with the R-package ‘qtl’ (Broman *et al.*, 2003). To check for main effect QTLs, interval mapping and composite interval mapping approaches were performed with Haley–Knott regression (Haley and Knott, 1992). The conditional genotype probabilities were calculated using the ‘calc.genoprob’ function with a step size of 1 cM and an assumed genotyping error probability of 0.0001 with the Kosambi map function. Composite interval mapping was performed using the ‘cim’ function with a pre-defined number of five covariates selected by forward selection and a window size of 20 cM. To correct for type I error rates (false-positive QTLs), a genome-wide logarithm of odds (LOD) score threshold was estimated by 10 000 permutations at alpha 0.05 (Churchill and Doerge, 1994). All QTLs detected were used as the initial QTL model. The ‘stepwiseqtl’ function was used for forward/backward selection of multiple QTL models, with model choice made via a penalized LOD score, with separate penalties on main effects and interactions. Individual ‘heavy’ and ‘light’ penalties were extracted from 3000 ‘scantwo’ permutations with the ‘calc.penalties’ function (Broman and Sen, 2009; Manichaikul *et al.*, 2009). For each trait, the derived multiple QTL model was plugged into the ‘fitqtl’ function to estimate QTL effects and percentages of variance (*R*^2^) explained by the individual QTLs from the final simultaneous fit of all QTLs. The 1.5-LOD support intervals (Manichaikul *et al.*, 2006) for each QTL were estimated with the ‘lodint’ function and expanded to the nearest flanking markers.

### Candidate gene identification

To identify candidate genes for mQTLs, compound and pathway information of the AraCyc 13.0 database was downloaded from the Plant Metabolic Network (PMN, plantcyc.org). Arabidopsis gene annotation information was obtained from the Arabidopsis Information Portal (ARAPORT 11, araport.org). For each mQTL, a search window was determined according to the 1.5-LOD support interval (Supplementary Data S4). Genes were extracted and tested for either direct association with the corresponding metabolite or indirect association with one of the pathways in which the metabolite is involved. To nominate the most promising candidate genes of the master regulatory locus detected on the short arm of chromosome IV, all 567 genes within the confidence interval of the hotspot on chromosome IV were analyzed for gene ontology (agriGO, bioinfo.cau.edu.cn/agriGO/) and matched against the plant transcription factor database (PlnTFDB 3.0, plntfdb.bio.uni-potsdam.de).

### Threshold determination for mQTL hotspots

For each mQTL, the nearest molecular marker to the LOD apex was determined and the numbers of associated mQTLs per marker were added up. The deviation from the random number of co-localizations was calculated by randomly distributing the mQTLs of each metabolite over the 138 marker positions and counting the maximum number of mQTL co-localizations. This procedure was repeated 10 000 times, yielding a distribution of the maximum numbers of mQTLs per marker. The 95% quantile of this distribution corresponded to 15 QTLs. Hence ≥15 mQTLs at the same genome position were regarded as significantly co-localized.

## Results and discussion

In the present study, we utilized a previously generated set of reciprocal RILs consisting of 202 Col-0×C24 and 191 C24×Col-0 F_10_ RILs (Törjék *et al.*, 2006). Pools of 60 seeds from three individuals per genotype were analyzed for their metabolic composition. After exclusion of contaminants and manual data curation, 311 metabolites (64 of known chemical structure) were detected in >85% of all samples. Only these metabolites were taken into further consideration. A comparison of the metabolic composition between the reciprocal subpopulations revealed no statistically significant differences (ANOVA/FDR, *P*>0.05 for all metabolites). No significant differences in marker distribution between the reciprocal RILs were found (association between marker matrices of 88.3% estimated by Mantel test with 10 000 permutations, *P*<0.001). Maternal effects, for example due to different cytoplasm, mitochondria, and chloroplasts, were excluded by a two-way ANOVA exemplarily testing for effects of markers, subsets, and marker–subset interactions on leucine abundance (Supplementary Data S5). In addition, a modified cross-validation (Melchinger *et al.*, 2004) was conducted treating the reciprocal RIL subsets individually and including randomly selected subsets in the mapping (Supplementary Data S6). In both cases, the reduction in population size led to a drastic decline of LOD scores and the detection of different combinations of leucine QTLs due to reduced detection power and random sampling effects. Hence the RILs were treated as one population and the corresponding results were used for all subsequent analyses.

### Correlation between metabolites, seed protein content, and seed weight

Correlations between levels of metabolites help to gain information about metabolic links (Carreno-Quintero *et al.*, 2012; Hill *et al.*, 2013). Correlated metabolites can be the result of shared metabolic pathways or enzymatic reactions, or indirect regulatory processes affecting different pathways. Pearson correlation/FDR (Supplementary Data S7) analyses resulted in far more positive (97%) than negative (3%) correlations between metabolites (Supplementary Figs S1, S2). At a global level, metabolites were mostly weakly or moderately correlated (0.2<*R*<0.6). This resembles previous studies in Arabidopsis (Cross *et al.*, 2006; Meyer *et al.*, 2007; Sulpice *et al.*, 2010) and tomato (Schauer *et al.*, 2006). Several significant correlations, including 55 correlations of known metabolites with seed protein content, seven correlations of known metabolites with seed weight content, and hundreds of pairwise correlations between metabolites, were detected. Strong correlations between metabolites of specific sectors of metabolism, including structurally related metabolites, or metabolites from the same biological pathway, were found, as reported previously (Schauer *et al.*, 2006; Lu *et al.*, 2008; Lin *et al.*, 2014). There was a strong correlation between the monosaccharides glucose and fructose (*R*=0.75). Other high correlations were detected: gentiobiose–erythritol (*R*=0.91), raffinose–*myo*-inositol (*R*=0.85), galactinol–*myo*-inositol (*R*=0.59), and raffinose–galactinol (*R*=0.57). Galactinol, raffinose, and *myo*-inositol are involved in the reversible reaction producing stachyose (Tanner and Kandler, 1968; Lehle and Tanner, 1973). High correlations between the citric acid cycle metabolites malic acid and citric acid (*R*=0.79) as well as fumaric acid and malic acid (*R*=0.55) were detected, supporting the finding that metabolites involved in the same pathway(s), in particular, are highly correlated (Sulpice *et al.*, 2010). The BCAAs valine, leucine, and isoleucine were significantly and highly correlated: valine–isoleucine (*R*=0.94), leucine–isoleucine (*R*=0.88), and valine–leucine (*R*=0.81). This might be due to their structural relationship and largely shared biosynthetic and degradation pathways (Binder, 2010; Angelovici *et al.*, 2013). A dominant role for amino acids in metabolite correlations has previously been described in tomato (Toubiana *et al.*, 2012) and soybean (Lin *et al.*, 2014).

Maturing Arabidopsis seeds mainly accumulate lipids in the form of triacylglycerols (TAGs), and seed storage proteins (SSPs) as storage macromolecules substantially contributing to the weight of mature seeds (Baud *et al.*, 2008). Although primary metabolites serve as feedstock for their synthesis, only low correlations between polar primary metabolites and seed weight were found. Erythritol (*R*=0.39) and gentiobiose (*R*=0.36) were the strongest positively correlated, and prephenic acid (*R*= –0.28) and ornithine (*R*= –0.26) the strongest negatively correlated metabolites of known structure. As lipids and lipophilic compounds contribute 34.6–46.0% to the seed dry weight (O’Neill *et al.*, 2003), they might be more important than polar primary metabolites.

In total, 276 significant correlations between metabolites and seed protein content were detected, including 55 metabolites of known chemical structure. However, most of these pairs are only moderately or slightly correlated (only 41 with *R*>0.5). The top four known metabolites correlated with protein content are glutamic acid (*R*=0.57), glucopyranose (*R*=0.57), melibiose (*R*=0.57), and pyroglutamic acid (*R*=0.56). The high correlation of glutamic acid with seed protein content was expected due to its central role in plant nitrogen transport and protein biosynthesis (Glass *et al.*, 2001). A recent study revealed that the glutamate decarboxylase- (GAD) mediated conversion of glutamate to γ-aminobutyric acid (GABA) during seed development plays an important role in balancing carbon and nitrogen metabolism, and storage reserve accumulation in Arabidopsis seeds, and affects seed protein content (Fait *et al.*, 2011). Glutamic acid is a key compound of cellular metabolism involved in the biosynthesis of many other amino acids. It acts as a substrate for glutamate dehydrogenases and various aminotransferases, providing 2-oxoglutarate for respiration (Forde and Lea, 2007). Moreover, it is a major transport form of nitrogen in plants (Xu *et al.*, 2012). Protein content and seed weight were not found to be significantly correlated.

In summary, moderate to high, mostly positive pairwise correlations were detected, especially for structurally or pathway-related metabolites. Unlike our initial hypothesis, only weak pairwise correlations between individual polar primary metabolites and seed weight could be detected. Lipophilic compounds or combinations of particular metabolites might yield higher correlations, as previously described (Meyer *et al.*, 2007).

### Natural variation in primary metabolism and detection of mQTLs and regions associated with seed protein content

**Fig. 1.**
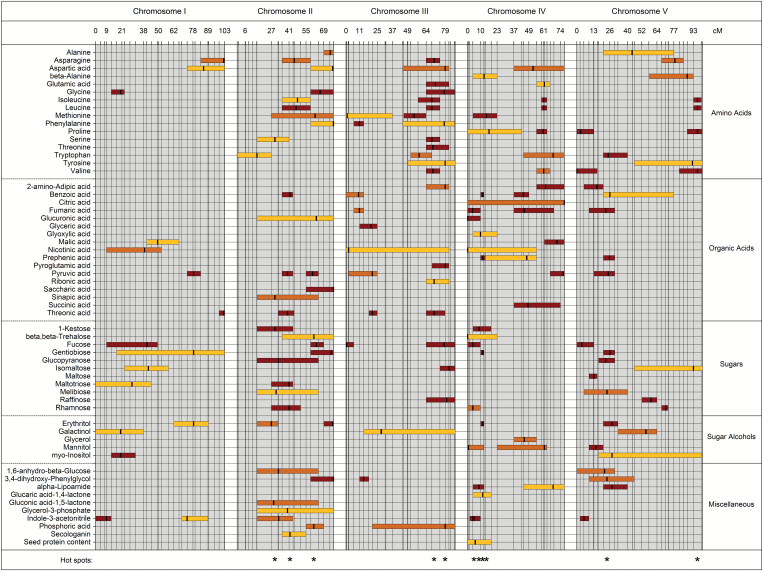
Distribution of mQTLs for metabolites of known chemical structure. Chromosomal locations of significant mQTLs for the 58 metabolites of known chemical structure and the seed protein content are indicated by boxes representing the 1.5-LOD QTL support intervals. Vertical black lines within the boxes indicate the apices of the corresponding LOD curves. The mQTLs are color-coded according to their significance [threshold at alpha of 0.05 (yellow), 0.01 (orange), 0.001 (red)] derived from permutation results of the genome-wide maximum LOD scores. Vertical lines represent marker positions. For a subset, their approximate distance in centiMorgans is indicated. Asterisks at the bottom correspond to the position of identified mQTL hotspots.

A total of 786 mQTLs affecting the metabolism of mature Arabidopsis seeds were identified by our mapping approach (Supplementary Data S8). For 90% of the 311 analyzed compounds, at least one mQTL was identified, reflecting a remarkably high variation emerging from the cross of two accessions. Lisec *et al.* (2008) used the same RILs and similar analytical approaches, but investigated leaf material of young vegetatively grown plants and found mQTLs for only 46% of the detected metabolites. Similarly, Rowe *et al.* (2008) identified mQTLs for 44% of the measured leaf metabolites. In the study presented here, 152 mQTLs were attributed to 58 metabolites of known (Fig. 1) and 634 mQTLs to 222 metabolites of unknown chemical structure. For 72 metabolites, only one QTL was identified. A maximum of seven QTLs was found for unknown mass spectral tags (MSTs) 40, 66, 218, 225, 232, and 245, respectively. Combined main effect QTLs explained up to 88.87% of phenotypic variation of particular metabolites, with explained phenotypic variation ranging from 0.57% to 77.68% for individual QTLs. On average, 7.23% of phenotypic variation is explained by a single QTL, suggesting that for most metabolites multiple unidentified genetic loci may contribute with moderate effects to overall phenotypic variation. In addition, the analysis revealed evidence for 30 epistatic interactions, including four metabolites of known structure: leucine, isoleucine, benzoic acid, and indole-3-acetonitrile (Supplementary Data S9). One QTL associated with seed protein content was detected on chromosome IV with ‘MASC04123’ (4.15 cM) as the nearest marker. The confidence interval for this QTL spanning a region of 18.7 cM on chromosome IV is overlapping with the confidence intervals of 22 QTLs for metabolites of known structure, including proline and methionine, and 146 QTLs for metabolites of unknown structure. However, only a small proportion of phenotypic variation of seed protein content (2.94%) is explained by this QTL.

The RIL population was previously utilized by Lisec *et al.* (2008), who quantified 181 leaf metabolites, including 85 compounds of known chemical nature, and identified 157 mQTLs for 84 metabolites (50 of known chemical structure). Comparing this with our findings, a total of 25 known metabolites with mQTLs, predominantly amino acids and organic acids, were detected in both studies. To compare the detected mQTLs, support intervals were extended to the nearest flanking markers to derive physical map positions. This comparison revealed nine mQTLs for seven metabolites with overlapping confidence intervals (Table 1), including a tyrosine mQTL on chromosome V that contains a tyrosine aminotransferase (At5g53970) involved in tocopherol synthesis in Arabidopsis (Riewe *et al.*, 2012). Untargeted LC-MS-based metabolomic approaches and quantitative genetic analysis enabled broad-spectrum molecular dissection of the Arabidopsis leaf metabolite composition of 160 Cvi×Ler RILs (Keurentjes *et al.*, 2006). Mapping of >2000 mass peaks resulted in the identification of mQTLs for ~75% of all mass signals, which is comparable with the detection rate in seeds in the present study. Another study used GC-MS to analyze 210 Bay×Sha RILs (Rowe *et al.*, 2008). They identified a total of 557 metabolites and used them for QTL mapping, leading to the identification of 438 mQTLs for 243 metabolites (36 known metabolites overlapped with those identified in the present study).

**Table 1.** Comparison of detected mQTLs in seeds and leaf material

Metabolite	Chromosome	Support interval	*R* ^2^ (%)^*a*^	Support interval Lisec *et al.* (2008)	*R* ^2^ (%)^*a*^ Lisec *et al.* (2008)
Glycine	III	17.27–23.28 Mbp	5.42	16.24–17.78 Mbp	8.00
Malic acid	IV	13.69–18.54 Mbp	9.85	10.67–15.39 Mbp	4.20
*myo*-Inositol	I	3.49–9.36 Mbp	5.32	4.12–6.50 Mbp	6.50
Raffinose	III	17.27–23.41 Mbp	4.30	16.24–19.50 Mbp	4.70
Serine	II	5.18–10.43 Mbp	3.30	3.00–5.33 Mbp	5.10
Serine	III	17.27–19.86 Mbp	7.54	15.17–17.78 Mbp	6.90
Tyrosine	III	14.30–23.41 Mbp	3.36	11.77–17.78 Mbp	4.20
Tyrosine	V	18.83–26.92 Mbp	3.50	21.92–22.91 Mbp	9.60

^*a*^ Estimated proportion of the phenotype variance explained by a QTL

By testing all 55 pairwise epistatic interactions between 11 detected metabolite QTL clusters against the average accumulation of 557 metabolites, Rowe *et al.* (2008) identified 240 metabolites with 1–5 significant epistatic interactions, for a total of 328 significant interactions. Lisec *et al.* (2008) detected 38 epistatic interactions involving metabolites of known structure. With only four interactions among mQTLs of known metabolites, much fewer incidences of epistasis were observed in the present study on Arabidopsis seed metabolites.

### Detection of enzyme-encoding mQTL candidate genes

To identify candidates, genes within the mQTL confidence intervals were extracted and queried for direct or indirect association with the particular metabolite using the AraCyc 13.0 database. Genes encoding enzymes catalyzing reactions that involve the metabolite as substrate or product were considered direct candidates, while genes encoding enzymes that catalyze other reactions within pathways that lead toward the formation of the metabolite or which consume the metabolite were considered indirect candidates. A total of 168 direct candidate genes for 27 of the 52 metabolites of known structure (52%) were found. Expanding search criteria to all pathways in which the metabolite is involved, a total of 765 direct and indirect candidate genes for 33 metabolites (~63%) were determined. These numbers differ from those observed by Lisec *et al.* (2008) utilizing the same RIL population, but analyzing leaf material. We found a higher percentage of direct candidate genes, which might be attributed to a less conservative calculation of mQTL confidence intervals. In contrast to the 43% increase found by Lisec *et al.* (2008), the extension of the search criteria to indirect associations increased the percentage of mQTLs with a candidate gene only by 21% in the present study.

### Shared mQTLs among branched-chain amino acids, epistatic interactions, and candidate genes

**Fig. 2.**
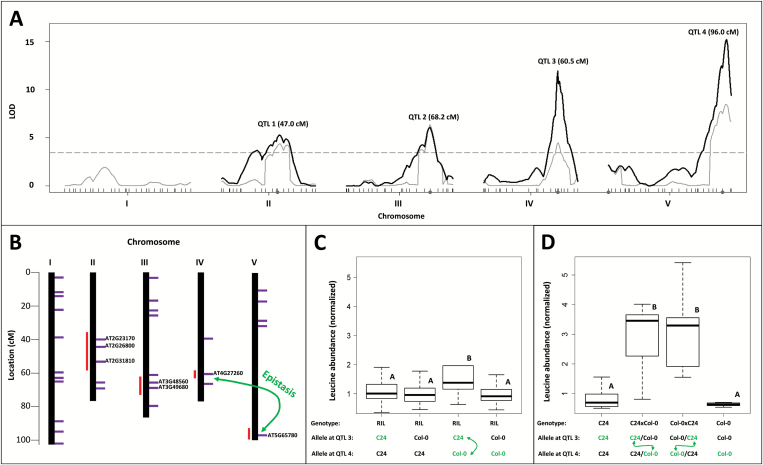
mQTL analysis and candidate gene identification for leucine. (A) LOD profiles were plotted for all five Arabidopsis chromosomes. Gray lines represent LOD profiles calculated with the ‘cim’ function (composite interval mapping). Gray dots indicate selected cofactors. The horizontal dashed gray line corresponds to a CIM alpha threshold of 0.05, estimated by 10 000 permutations. The solid black lines indicate LOD profiles calculated with the ‘stepwiseqtl’ function using a multiple QTL model. The positions of the QTL apices in centiMorgans are given above the curves. (B) A simplified genetic map with known and putative genes involved in leucine biosynthesis and degradation. Purple horizontal lines indicate the locations of genes, directly or indirectly involved in leucine metabolism. Leucine mQTLs were identified on chromosomes II, III, IV, and V. Support intervals are shown as red vertical lines beside the chromosomes. Leucine-related genes, located within the confidence intervals of the mQTLs, are indicated. Identified candidate genes for chromosome II are *AT2G23170* (*GH3.3*), *AT2G26800* (*HML1*), and *AT2G31810*, for chromosome III *AT3G48560* (*AHAS*) and *AT3G49680* (*BCAT3*), for chromosome IV *AT4G27260* (*GH3.5*), and for chromosome V *AT5G65780* (*BCAT5*). (C) Boxplots of normalized and median divided leucine abundances in seeds of RILs. Samples were subdivided into four groups according to the allelic state at the epistatically interacting loci on chromosomes IV and V. Significant differences between the groups are indicated by upper case letters (ANOVA with post-hoc Tukey HSD, *P*_adj_<0.001; number of individuals: *n*_C24/C24_=113, *n*_Col-0/C24_=20, *n*_C24/Col-0_ =82, *n*_Col-0/Col-0_=149). (D) Boxplots of normalized and median divided leucine abundances in seeds of parental and reciprocal F_1_ hybrid plants derived from an independent experiment. Significant differences between the groups are indicated by upper case letters (ANOVA with post-hoc Tukey HSD, *P*_adj_<0.05; number of individuals: *n*_C24_=7, *n*_C24×Col-0_=5, *n*_Col-0×C24_=5, *n*_Col-0_=5).

For leucine and isoleucine, shared loci and epistatic interactions between the mQTLs on chromosomes IV (60.5 cM) and V (96.0 cM) were observed (Supplementary Data S8, S9). Furthermore, high correlations were found between leucine, valine, and isoleucine (Supplementary Data S7). These findings prompted us to look at candidate genes within the leucine mQTLs in more detail. Leucine, valine, and isoleucine are characterized by their branched hydrocarbon residues. They form the small group of BCAAs that are critical for protein synthesis and normal plant growth (Yu *et al.*, 2013) and serve as precursors for secondary metabolites (Binder, 2010; Buchanan *et al.*, 2015). Plants synthesize these essential amino acids *de novo*. Valine and isoleucine are synthesized in two parallel pathways using a single set of four enzymes, whereas the pathway to leucine branches off and requires three additional steps (Binder, 2010). The BCAA biosynthesis in plants occurs in chloroplasts (Diebold, 2002; Binder, 2010), whereas the degradation mostly takes place in mitochondria (Zolman *et al.*, 2001; Beck *et al.*, 2004). Despite the limited mapping resolution provided by RILs, it is possible to identify candidate genes underlying biochemical pathways (Lisec *et al.*, 2008; Brotman *et al.*, 2011). To this end, the four detected mQTLs for leucine (Fig. 2A; Supplementary Data S8) were screened for known and putative pathway genes involved in BCAA metabolism. From three databases, ARAPORT 11 (araport.org), AraCyc 13.0 (PMN, plantcyc.org), and KEGG PATHWAY (www.genome.jp/kegg/pathway.html, last accessed 16 February 2017), genes annotated in leucine biosynthesis and degradation were extracted and their map positions compared with the leucine mQTL confidence intervals. Candidate genes could be identified for all four leucine mQTLs. Three candidate genes: *AT2G23170* (*GH3.3*), *AT2G26800* (*HML1*), and *AT2G31810* (*AHAS*), were associated with the mQTLs on chromosome II. *AT2G23170* (*GH3.3*) encodes an indole-3-acetic acid (IAA)-amido synthetase (Staswick *et al.*, 2005), *AT2G26800* a putative hydroxymethylglutaryl-CoA lyase, presumably involved in leucine degradation, and *AT2G31810* a small regulatory subunit of the acetolactate synthase (Binder, 2010). The acetolactate synthase is the first common enzyme in the biosynthetic pathways of the BCAAs (Chen *et al.*, 2010) and catalyzes the conversion of two molecules of pyruvate into (*S*)-2-acetolactate, or one molecule of pyruvate and one molecule of 2-oxobutanoate into 2-aceto-2-hydroxybutyrate (Singh, 1999; Duggleby *et al.*, 2008). Two candidate genes co-localized with the confidence interval of the leucine mQTLs on chromosome III. *AT3G48560* (*AHAS*) encodes the catalytic subunit of the acetolactate synthase (pyruvate decarboxylase). Mutants defective in this gene exhibit increases in all three BCAAs in mature seeds (Lu *et al.*, 2011). *AT3G49680* (*BCAT3*) encodes a BCAA aminotransferase, which is involved in the biosynthesis and degradation of valine, leucine, and isoleucine (Knill *et al.*, 2008). For the confidence interval on chromosome IV, *AT4G27260* (*GH3.5*) could be identified as a direct candidate gene encoding an IAA-amido synthetase, which conjugates various amino acids, including leucine, to IAA. IAA is the prevalent form of auxin, an important phytohormone, affecting many aspects of plant development and plant response to biotic and abiotic stimuli (Woodward and Bartel, 2005). Some of these amino acid modifications can be reversed by amido hydrolases (Davies *et al.*, 1999), suggesting that IAA–amino acid conjugates, such as IAA–leucine, are storage forms of auxin (Staswick *et al.*, 2005). These compounds can be metabolized to contribute to the pool of free auxin, allowing plants to fine-tune their levels of active auxin (Woodward and Bartel, 2005). The confidence interval for the leucine mQTLs on chromosome V harbors *AT5G65780* (*BCAT5*), encoding another member of the BCAA transaminase gene family. Green fluorescent protein tagging localized the protein to the chloroplast, but recent proteomic studies indicated a mitochondrial localization (Diebold, 2002; Taylor, 2004; Zybailov *et al.*, 2008). Although its localization is still controversial and BCAT5 has not yet been characterized in detail, its role in leucine metabolism has been suggested (Schuster *et al.*, 2006; Binder, 2010). A recent genome-wide association study using a diversity panel of 360 Arabidopsis accessions in conjunction with a QTL analysis of a RIL population derived from accessions Bay-0 and Shahdara revealed the unique, catabolic role of the *AT1G10070* locus (*BCAT2*) in BCAA metabolism in Arabidopsis seeds (Angelovici *et al.*, 2013). However, as these accessions are polymorphic for several SNPs and insertion/deletion events, the causative molecular mechanism(s) underlying the phenotypic differences in Bay-0 and Shahdara *BCAT2* alleles were not determined. In the present study, no significant association with the *BCAT2* locus or any other region on chromosome I was detected. A comparison of the C24 and Col-0 alleles of the *BCAT2* locus revealed several polymorphisms (Supplementary Data S10). Only one polymorphism within the coding sequence leading to a glutamic acid to aspartic acid exchange was detected. Both amino acids have comparable properties and are thus unlikely to cause substantial differences of the BCAT2 protein. This absence of functional relevant differences between the C24 and Col-0 alleles could explain the fact that no significant influence of the *BCAT2* locus was detected in the C24/Col-0 RIL population. A simplified genetic map with genes involved in leucine biosynthesis and degradation is shown in Fig. 2B. Comparing detected mQTLs for all BCAAs, overlapping confidence intervals were detected. Leucine and isoleucine share all detected mQTLs on chromosomes II, III, IV, and V, including a putative epistatic interaction between mQTLs on chromosomes IV (60.5 cM) and V (96.0 cM). For leucine and valine, mQTLs on chromosomes III, IV, and V share overlapping confidence intervals. For valine there is no mQTL on chromosome II, but an additional mQTL on chromosome V that is not shared by leucine and isoleucine. Its confidence interval harbors one candidate gene (*AT5G09300*, *BCKDH*) encoding a putative subunit of the branched-chain keto acid dehydrogenase complex that catalyzes the second step of BCAA degradation (Binder, 2010; Peng *et al.*, 2015).

To investigate further the influence of the mQTL on chromosomes IV and V and their putative interaction, samples were divided into four groups based on their allelic states and the leucine abundances were plotted (Fig. 2C). The C24 allele at the locus on chromosome V has an increasing effect on the leucine abundance, but only if the Col-0 allele is present at the locus on chromosome IV. The presence of the C24 allele at the locus on chromosome IV leads to no substantial difference in leucine abundance, independent of the allele at the locus on chromosome V. This finding is consistent with the detected epistatic interaction between the mQTLs on chromosomes IV and V and higher leucine abundances in seeds of the reciprocal hybrids compared with their parental accessions (Fig. 2D).

### Detection of mQTL hotspots for Arabidopsis seed metabolism

QTL studies in various species identified hotspots (Schauer *et al.*, 2006, 2008; Joosen *et al.*, 2013; Chen *et al.*, 2014; Alseekh *et al.*, 2015; Wen *et al.*, 2015), but their number and position can vary across tissues within a specific population, as found in rice (Gong *et al.*, 2013). There are two potential explanations for these observations. It could be a reflection of an uneven distribution of biosynthetic genes over the genome, or may be a consequence of the occurrence of pleiotropic or regulatory genes of higher hierarchical order controlling multiple metabolic reactions rather than individual metabolic conversions. A study comparing the distribution of metabolic genes in the genome with the mQTL distribution has provided evidence that a large proportion of detected mQTLs, with hitherto unknown metabolic functions, are most probably regulatory genes controlling primary metabolism (Lisec *et al.*, 2008).

The mQTLs detected in this study were not evenly distributed across the Arabidopsis genome. In some regions, mQTLs clustered, whereas other regions were depleted of QTLs. Since 311 metabolic traits and 138 markers were taken into account, stochastic co-localizations of mQTLs are to be expected. The threshold for detection of significant enrichment of mQTLs in certain positions was determined using 10 000 permutations. Markers associated with at least 15 mQTLs were regarded as significant hotspots. In the present study, we found evidence for several mQTL hotspots on chromosomes II, III, IV, and V (Table 2; Supplementary Fig. S3). The smallest number of mQTLs (*n*=90) and no hotspot were found for chromosome I. On average, substantially fewer mQTLs per marker were detected on chromosome I than expected for a random distribution. For chromosome II, a total of 148 mQTLs and three hotspots were identified. On chromosome III, 143 mQTLs were detected, with evidence for two large hotspots. Chromosome IV shows evidence for 220 mQTLs, and at least four markers were identified as hotspots. Three of them are localized on the short arm of the chromosome and might be regarded as a single hotspot due to their proximity. Their combined median confidence interval is delimited by the markers ‘MASC04123’ and ‘MASC04685’ and extends beyond the short arm of chromosome IV. It spans a region of ~5 Mbp and contains 567 genes. For chromosome V, a total number of 185 mQTLs and the largest hotspot with 94 mQTLs, associated with the marker ‘MASC09209’, were detected. Calculating the median confidence interval over all mQTLs sharing this marker, the hot spot is delimited by the markers ‘MASC09208’ and ‘nga139’, spanning a region of ~6.5 Mbp, and containing 487 genes.

**Table 2.** Summary of mQTL hotspots

Chromosome	Marker	Position (kbp)	Position (cM)	Number of mQTLs
II	M2_4269	8410.151	32.48	16
II	MASC02644	10 428.938	41.29	20
II	MASC09222	14 375.406	58.38	34
III	MASC09224	18 501.466	68.17	44
III	MASC02788	20 744.711	78.77	32
IV	MASC04123	301.329	4.15	27
IV	MASC04725	1092.491	10.21	35
IV	MASC05042	2188.362	12.90	44
IV	MASC04685	5230.768	14.01	16
V	MASC09209	7717.922	26.27	94
V	MASC09211	25 579.812	92.79	15

Hotspots of mQTLs have been previously reported in Arabidopsis (Keurentjes *et al.*, 2006; Fu *et al.*, 2009; Chan *et al.*, 2010; Joseph *et al.*, 2013). Lisec *et al.* (2008) identified two mQTL hotspots, on the short arm of chromosome IV and at the bottom of chromosome V, with 16 and 12 mQTLs, respectively. A biomass QTL and multiple mQTLs (Lisec *et al.*, 2008), as well as an early stage biomass heterosis QTL (Meyer *et al.*, 2010) were found in similar positions on the short arm of chromosome IV as the hotspot in the present study. The studies of Rowe *et al.* (2008) and Joosen *et al.* (2013) on the Bay×Sha RIL population revealed 11 and 8 mQTL hotspots, respectively. Two major hotspots (*AOP* on chromosome IV and *Elong* on chromosome V) may correspond to here reported hotspots on chromosomes IV (10.21 cM and 12.9 cM) and V (26.27 cM). These loci co-localize with known QTLs that determine the transcript accumulation of aliphatic glucosinolate biosynthetic genes and the accumulation and structure of aliphatic glucosinolates (Wentzell *et al.*, 2007). However, another study indicated that expression QTL (eQTL) hotspots may not overlap in different populations (Cubillos *et al.*, 2012).

### A region on the short arm of chromosome IV is responsible for the major proportion of metabolic variation

**Fig. 3.**
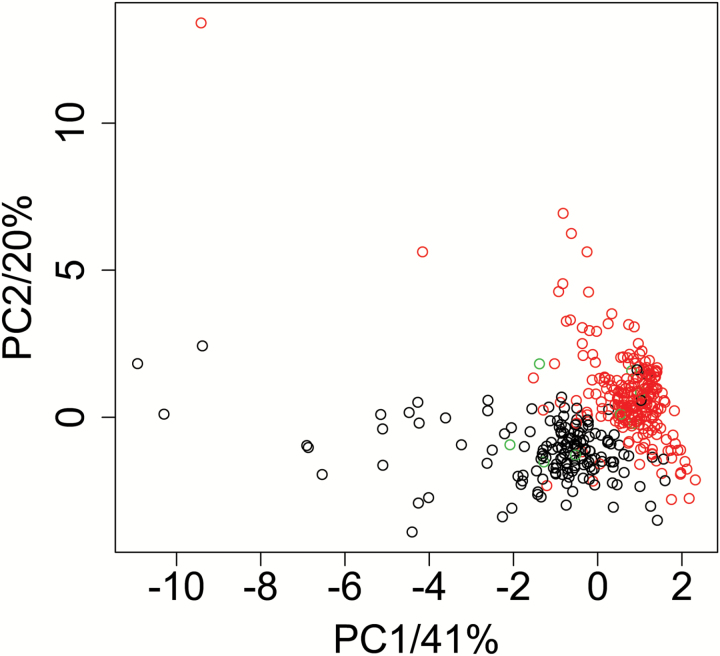
Principal component analysis of metabolite data. Score plot of the first two principal components PC1 and PC2 explaining 41% and 20% of variance of the data set, respectively. Samples were colored according to the genotype information on chromosome IV/marker: ‘MASC05042’ (12.90 cM). Black, red, and green circles correspond to Col-0, C24, and heterozygous alleles, respectively. Data were normalized, Pareto scaled, and mean centered prior to the calculation of the principal components.

To identify major effects on metabolism, a PCA was performed. The first four principal components explained 41, 20, 7.4, and 5.6% of the variance, respectively. The top loading metabolites of PC1 are unknown MSTs 124, 40, 222, and 105, gentiobiose, and galactinol, and for PC2 unknown MSTs 90, 74, 155, 40, and 85 (Supplementary Data S11). The first two principle components separated the samples into two narrow clusters. Labeling the samples according to extraction batches, measurement time point, or the two RIL subsets did not explain the clustering (Supplementary Data S12). Hence, samples were labeled according to the genotype information sequentially for all 138 markers (Supplementary Data S12). The allelic distribution at marker ‘MASC05042’ on the short arm of chromosome IV (12.90 cM) closely matches the clustering (Fig. 3). All markers in a region of ~23.38 cM, ranging from ‘MASC02820’ to ‘MASC02668’, display a similar pattern, indicating that this region contributes to a large proportion of the overall metabolic variation. To investigate this effect further, PCA scores were included in the QTL analysis to identify genomic regions of high importance affecting multiple correlated metabolites, yielding 13 loci significantly associated with the first four principal components (Supplementary Data S8). Three genomic regions were associated with PC1 responsible for the largest proportion of variance (41%). One QTL was identified on chromosome IV, spanning a region of 25 cM. Another QTL of 14 cM was located on chromosome V. The most prominent QTL for PC1 was identified on the short arm of chromosome IV (12 cM) with ‘MASC05042’ as the nearest marker to the LOD peak. Its confidence interval spans 2.7 cM and includes several mQTLs for PC1 loadings. The PC1 QTL is localized within the mQTL hotspot region on the short arm of chromosome IV that is responsible for the major clustering in the PCA (Table 2; Supplementary Data S12). In previous studies, mQTL hotspots were found at similar positions (Lisec *et al.*, 2008; Rowe *et al.*, 2008; Joosen *et al.*, 2013). The *FRIGIDA* (*FRI*; *AT4G00650*) locus, which encodes a major determinant of Arabidopsis flowering time (Johanson *et al.*, 2000), is located ~8.8 cM (1.9 Mbp) distal to ‘MASC05042’ (marker ‘F6N23ID’ detects a polymorphism within *FRIGIDA*). The PC1 QTL displays a very sharp peak with a steeply declining LOD profile and the *FRIGIDA* locus is not included within its confidence interval (Supplementary Fig. S4). Moreover, the top five loadings of PC1 share similar LOD profiles. Considering these facts, it is rather unlikely that allelic variation of *FRIGIDA* is causal for the observed complex metabolic variation conditioned by the QTLs on the short arm of chromosome IV. In contrast, these findings indicate that another, as yet unknown, master regulatory gene of seed metabolism or a major effector of seed development that has profound consequences on metabolite composition (e.g. through differences in size of certain tissues) is located in the identified region of chromosome IV and is responsible for major parts of the observed phenotypic variation.

As an entry into the identification of promising candidate regulatory genes for further analyses, all 567 genes within the confidence interval of the hotspot on chromosome IV were analyzed for gene ontology (agriGO, bioinfo.cau.edu.cn/agriGO/; Du *et al.*, 2010) and matched against the plant transcription factor database (PlnTFDB 3.0, plntfdb.bio.uni-potsdam.de; Riaño-Pachón *et al.*, 2007). Several genes were annotated with kinase or phosphatase activity, but annotations gave no direct hints of seed metabolic processes. A total of 38 annotated transcription factors are located within the hotspot (Supplementary Data S13). According to the data accessible in the Arabidopsis eFP Browser 2.0 (www.bar.utoronto.ca/efp2/Arabidopsis/Arabidopsis_eFPBrowser2.html, last accessed 16 February 2017 , Winter *et al.*, 2007), 12 of them display a high relative expression in mature and/or developing Arabidopsis seeds (Table 3). Three of them, *AT4G01120*, *AT4G01280*, and *AT4G02640*, are highly or predominantly expressed in mature (and developing) seeds. A particularly interesting candidate is *AT4G02640*, which encodes a basic leucine zipper transcription factor, (bZIP10). bZIP10 has been shown to interact with ABI3 (*AT3G24650*), a central transcriptional regulator of seed maturation (MAT) genes in Arabidopsis, and to activate seed storage protein gene expression synergistically (Lara *et al.*, 2003). Heterodimerization of bZIP53 with bZIP10 significantly enhances DNA binding activity and produces a synergistic increase in target gene activation (Alonso *et al.*, 2009). Furthermore, these bZIP heterodimers interact with ABI3, which further increases MAT gene activation. Comparing the C24 and Col-0 alleles of *bZIP10*, several polymorphisms (synonymous substitutions) and more interestingly a 7 bp insertion in the putative promotor region of C24 were detected (Supplementary Data S10).

**Table 3.** Selection of transcription factor (TF) genes within the mQTL hotspot on chromosome IV expressed in seeds

AGI locus identifier	TF family	General expression profile^*a*^	Seed-specific expression profile^*a*^
*AT4G00730*	HB	Ubiquitous	Intermediate development
*AT4G01120*	bZIP	Seed specific	Late development and mature seeds
*AT4G01250*	WRKY	Preferentially in seeds	Late development
*AT4G01280*	MYB-related	Seeds and other organs	Mature seeds
*AT4G01460*	bHLH	Uubiquitous	Intermediate development
*AT4G01500*	ABI3VP1	Ubiquitous	Early and intermediate development
*AT4G01580*	ABI3VP1	Seeds and other organs	Intermediate development
*AT4G02020*	SET	Ubiquitous	Early development
***AT4G02640***	**bZIP**	**ubiquitous**	**Late development and mature seeds**
*AT4G02670*	C2H2	Seeds and other organs	Early development
*AT4G03170*	ABI3VP1	Seeds and other organs	Late development
*AT4G04890*	PDF2	Ubiquitous	Intermediate development

^*a*^ According to Arabidopsis eFP Browser 2.0

In conclusion, the results provided by this study substantially enhance our current knowledge about Arabidopsis seed metabolism and natural variation of complex traits. It provides a broad and solid basis for further studies towards broadening the knowledge of factors mediating or regulating plant seed metabolism: detailed investigations can immediately be initiated on the enzyme-encoding candidate genes identified here; metabolic factors, such as transporters or regulators, may be selected via further database searches. Furthermore, upon confirmation and fine mapping of detected mQTLs, for example by the use of introgression lines (ILs), novel factors of hitherto unknown function can be identified. Finally, the evidence provided of a master regulatory locus of seed metabolism on the short arm of chromosome IV and the hints towards a corresponding candidate transcription factor gene are of particular interest as they provide a direct entry into further unraveling of important processes of seed development and maturation.

## Supplementary data

Supplementary data are available at *JXB* online.

Fig. S1. Histogram of metabolite correlations.

Fig. S2. Correlation matrix.

Fig. S3. Overview of mQTL hotspots.

Fig. S4. LOD profile of PC1 (chromosome IV).

Data S1. Summary of phenotypic data.

Data S2. Additional set of markers.

Data S3. Genetic map.

Data S4. mQTL candidate genes.

Data S5. Summary of the two-way ANOVA.

Data S6. Modified cross-validation of leucine mQTLs.

Data S7. Correlation analysis.

Data S8. Summary of QTL analysis.

Data S9. Summary of epistatic interactions.

Data S10. Comparison of C24 and Col-0 alleles for *BCAT2* and *bZIP10*.

Data S11. Principal component loadings.

Data S12. Principal component plots.

Data S13. Transcription factors within the hotspot on chromosome IV

## Supplementary Material

Supplementary_Dataset_S1_S5_S7_S9_S11_S13

Supplementary_Dataset_S6

Supplementary_Dataset_S10

Supplementary_Dataset_S12

Supplementary_Figures_S1_S4
