# Supplementary material for: Genetic dissection of metabolite variation in Arabidopsis seeds: evidence for mQTL hotspots and a master regulatory locus of seed metabolism
Source: J Exp Bot. 2017 Mar 6;68(7):1655–67. doi: 10.1093/jxb/erx049 (PMC5444479; doi:10.1093/jxb/erx049)
Supplement: Supplementary_Dataset_S6 [file erx049_suppl_Supplementary_Dataset_S6.pdf]

Data S6. Modified cross-validation of Leucine mQTL

To exclude maternal effects a modified cross-validation approach was conducted, subdividing the RILs into the two reciprocal RIL subsets: Col-0xC24 (n=202) and C24xCol-0 (n=191). Grey lines represent LOD profiles calculated with the 'cim' function (composite interval mapping). Black dots indicate selected cofactors. The horizontal dashed grey line corresponds to an alpha threshold of 0.05, estimated by 10,000 permutations for the composite interval mapping. The solid black lines indicate LOD profiles calculated with the 'stepwiseqtl' function using a multiple QTL model. The positions of the QTL apices in cM are given above the curves. The reduction of the population size by dividing the RILs into the two reciprocal subsets lead to a drastic reduction in detection power, indicated by the low LOD scores. Three of the four previously identified leucine mQTL were also detected in the split population (subsets 1 & 2). Randomly subdividing a population may also result in different subsets of detected QTL due to sampling effects. Therefore, ten randomly selected subsets of the RIL population, each containing 100 C24xCol-0 and 100 Col-0xC24 RILs, were also included in the analysis (random subset 1 to 10). As expected, different combinations of QTL were detected in the subsets. Overall, the QTL found in the random subsets correspond to the previously identified QTL for leucine in the whole RIL population.

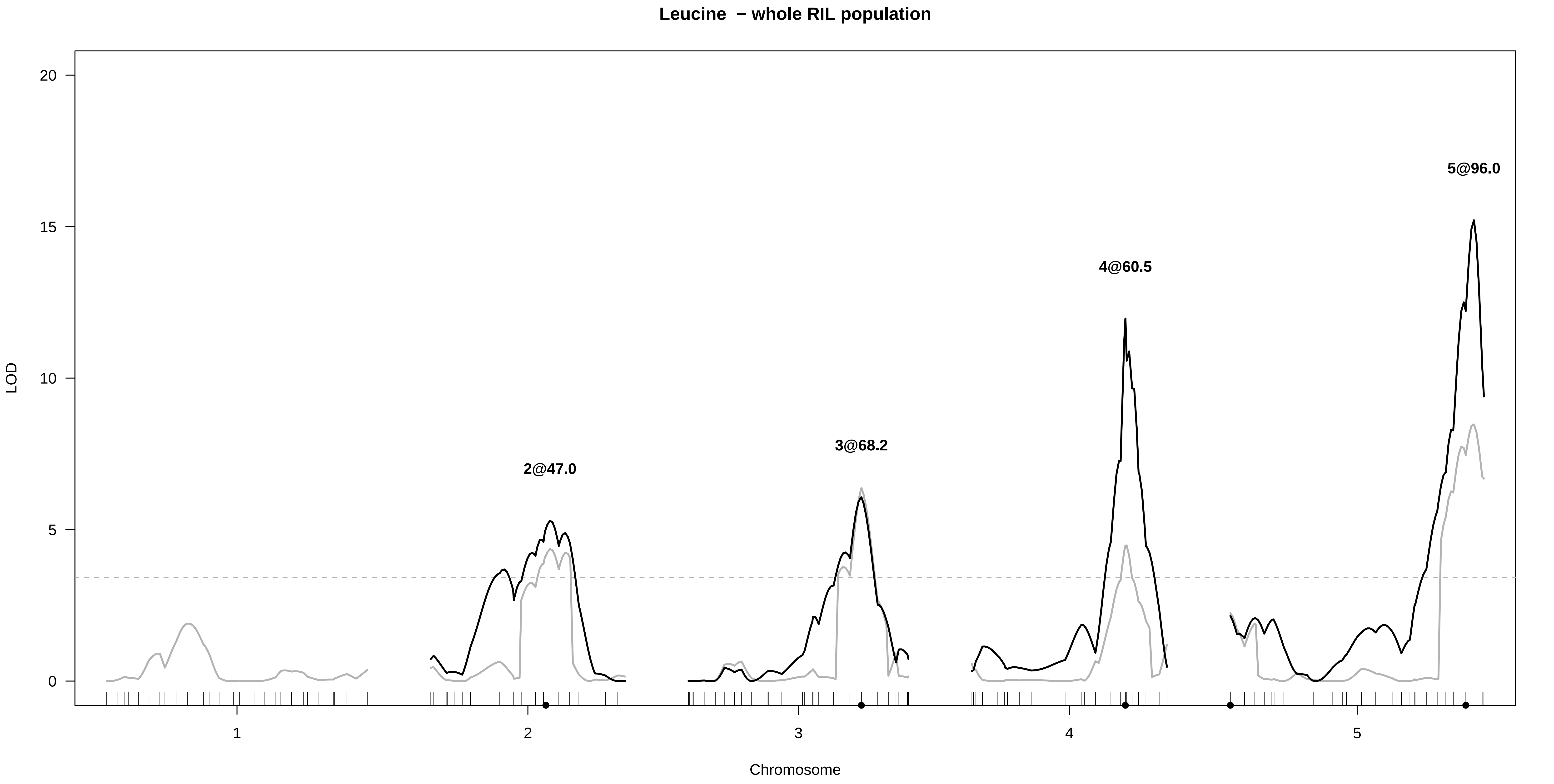
Leucine − whole RIL population
LOD
0
5
10
15
20
2@47.0
3@68.2
4@60.5
5@96.0
1
2
3
4
5
Chromosome

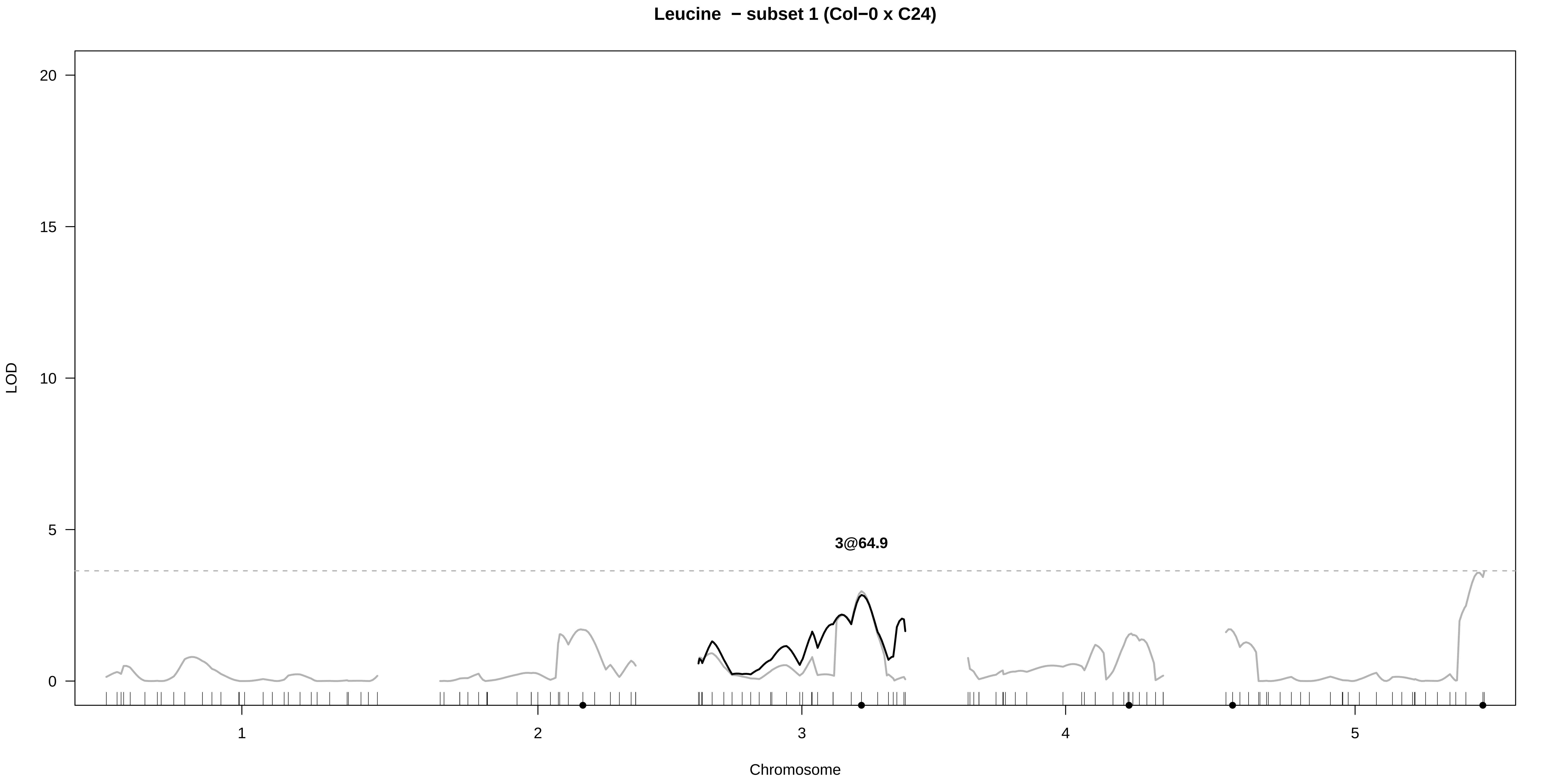
Leucine − subset 1 (Col−0 x C24)
LOD
20
15
10
5
0
3@64.9
1
2
3
4
5
Chromosome

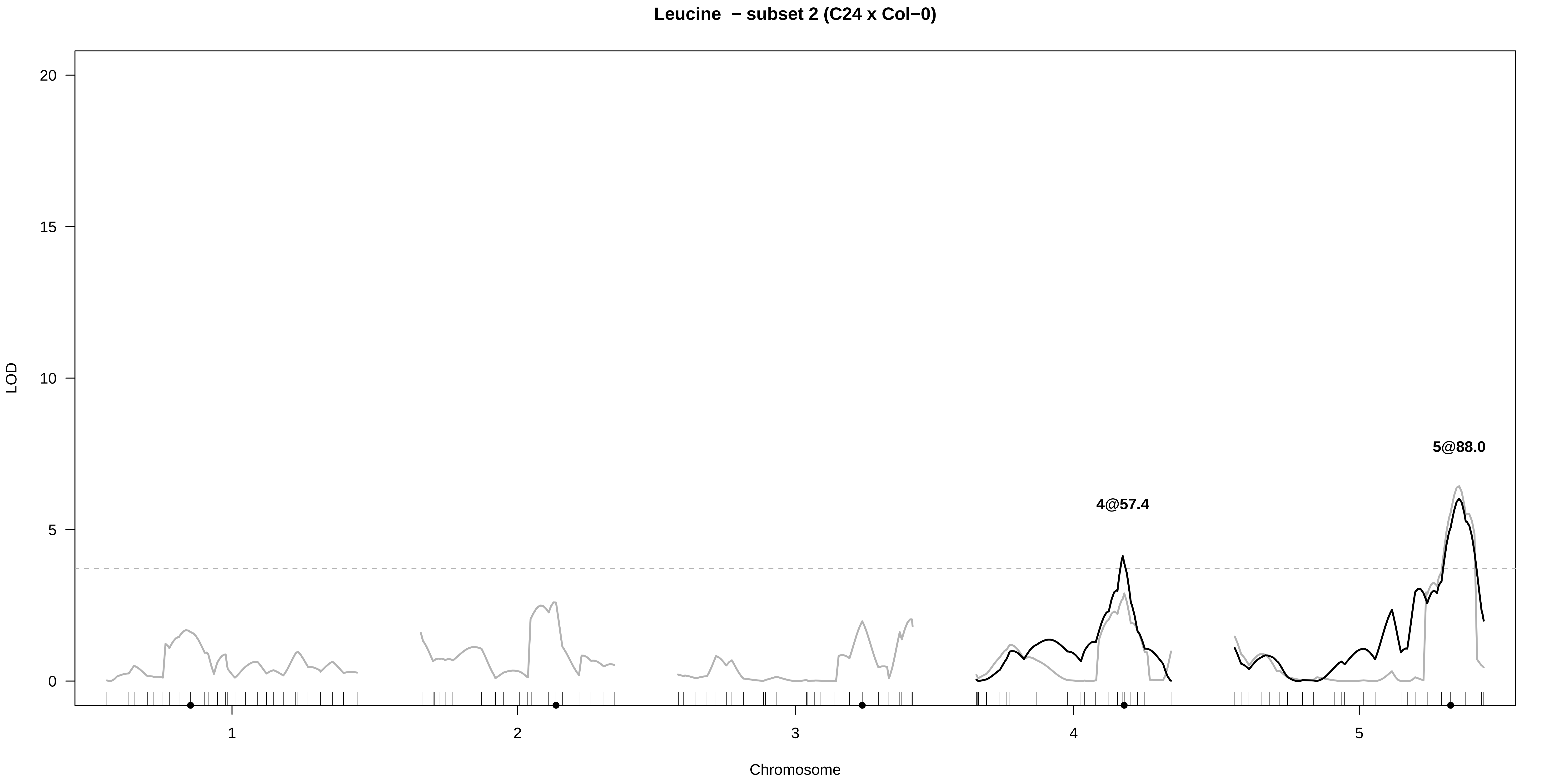

Leucine − subset 2 (C24 x Col−0)
LOD
20
15
10
5
0
4@57.4
5@88.0
1
2
3
4
5
Chromosome

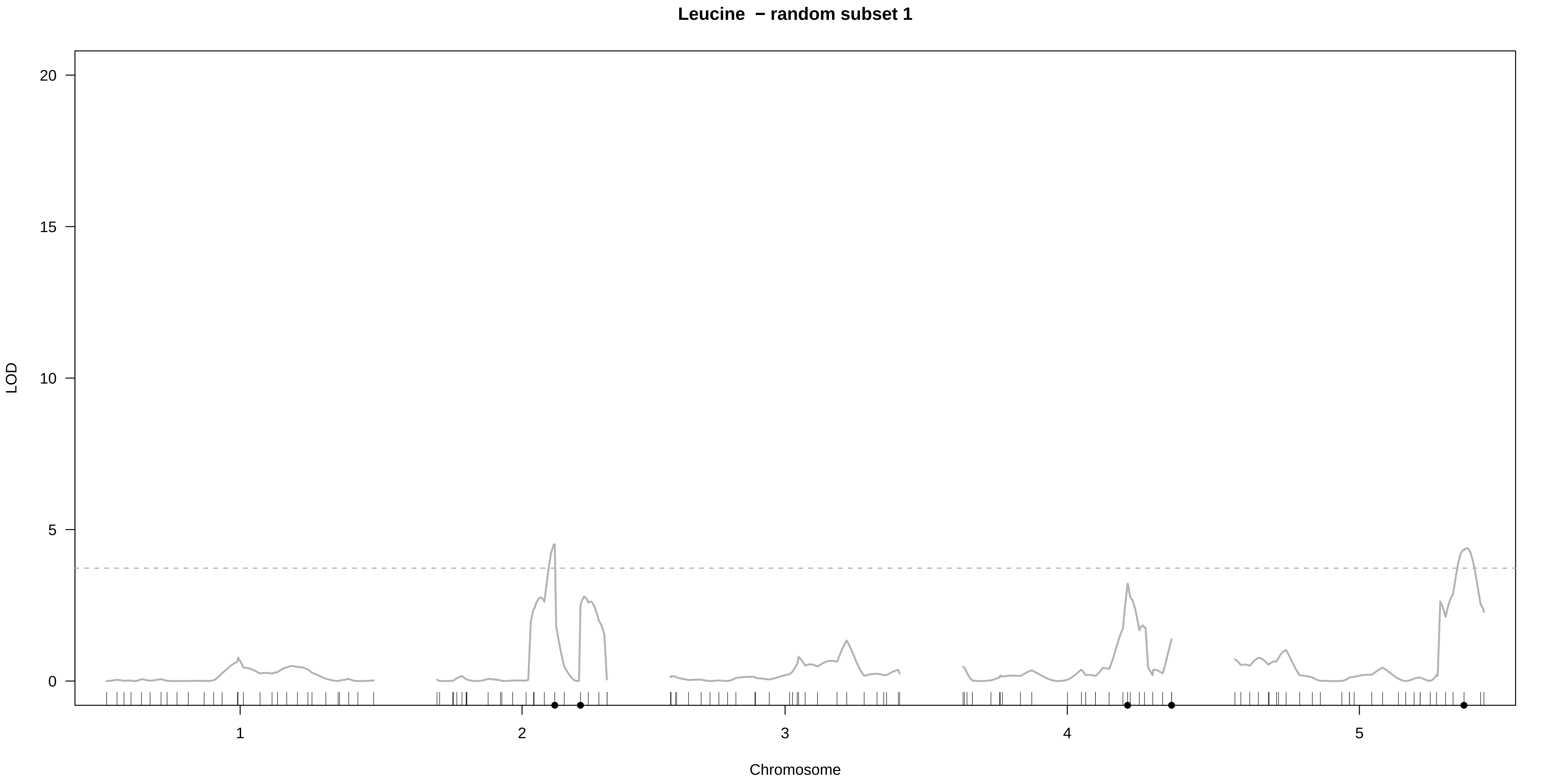
Leucine − random subset 1
LOD
20
15
10
5
0
Chromosome
1
2
3
4
5

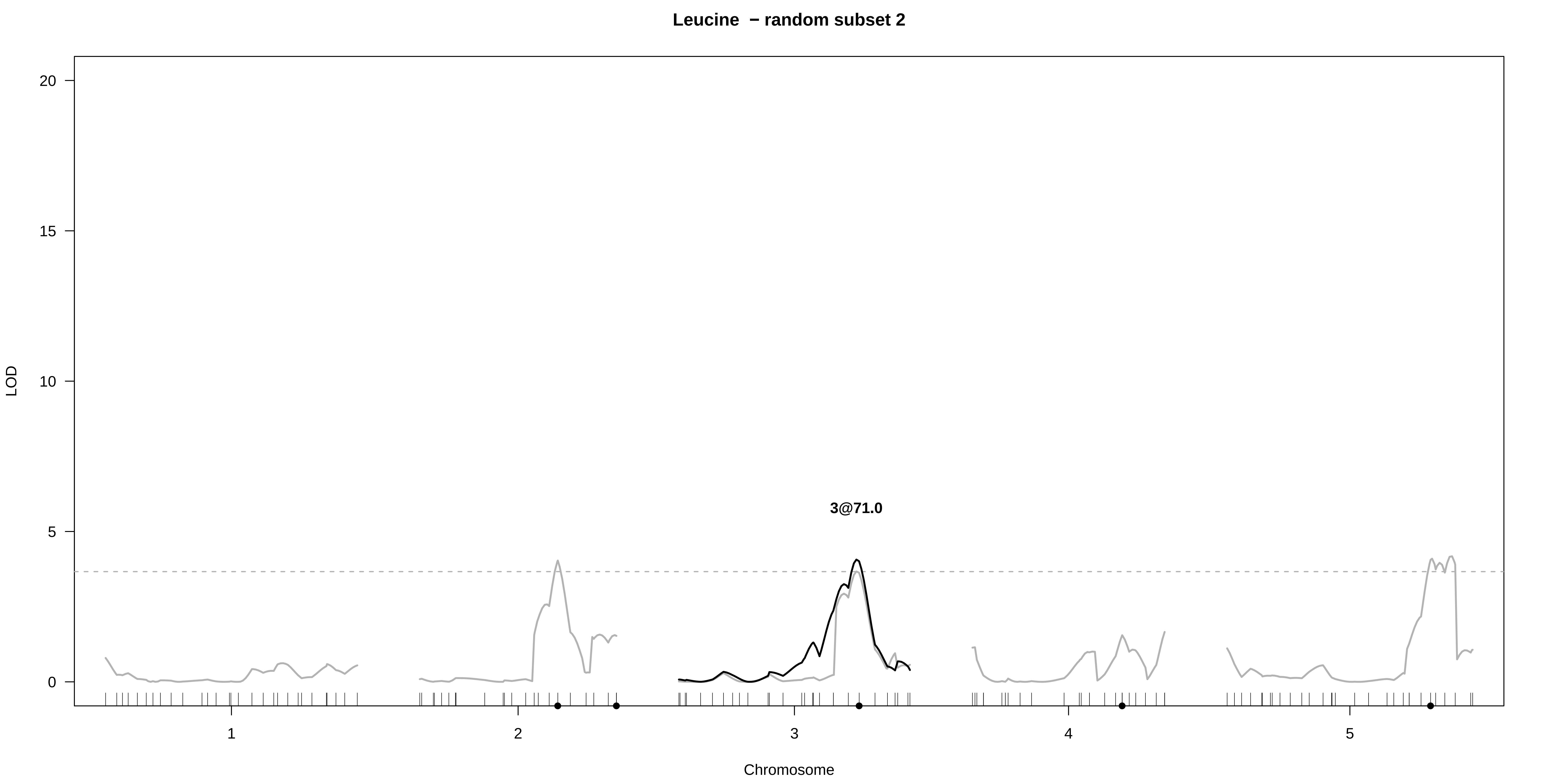

Leucine − random subset 2
LOD
20
15
10
5
0
3@71.0
1
2
3
4
5
Chromosome

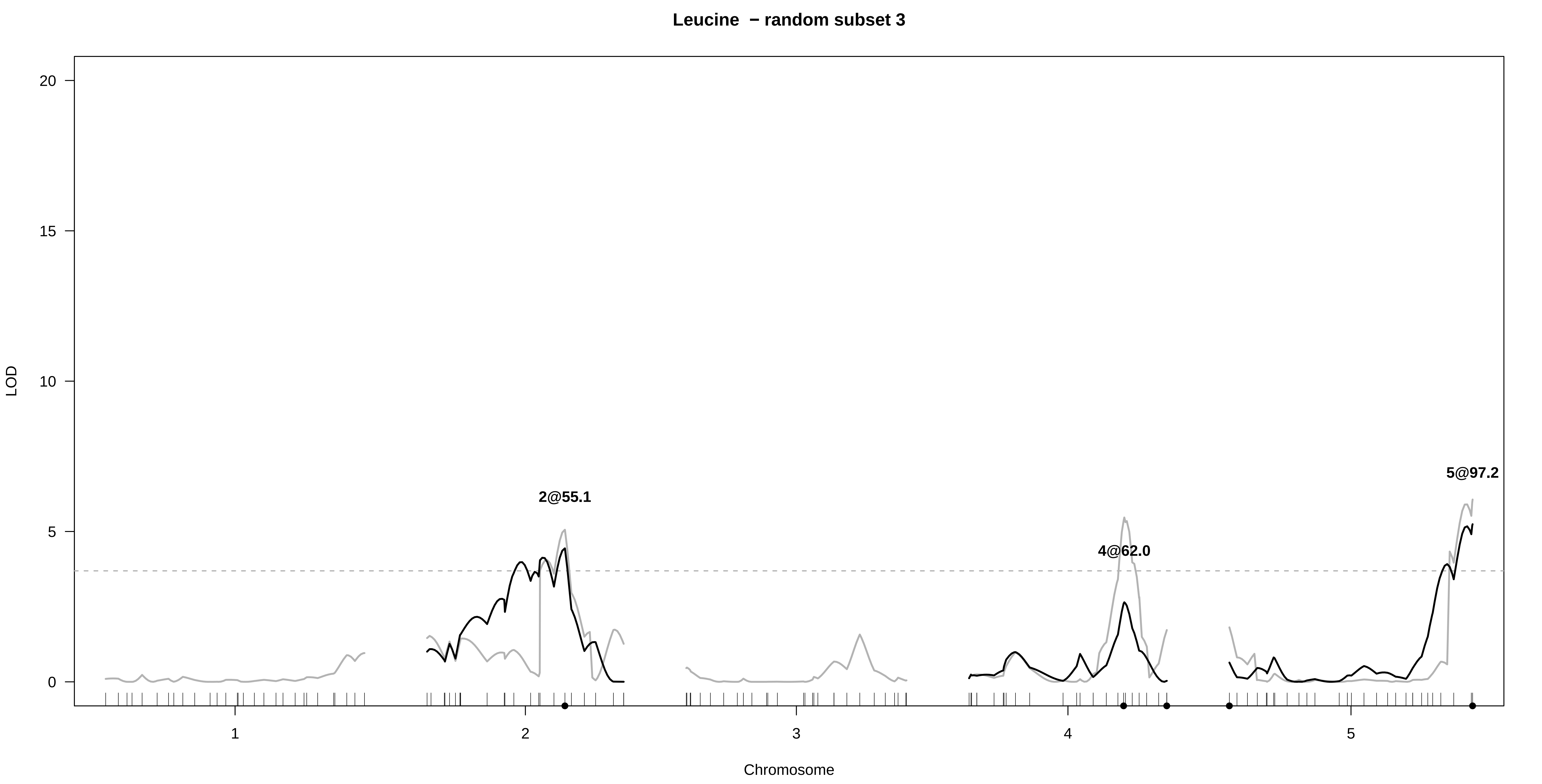

Leucine − random subset 3
LOD
0
5
10
15
20
2@55.1
4@62.0
5@97.2
1
2
3
4
5
Chromosome

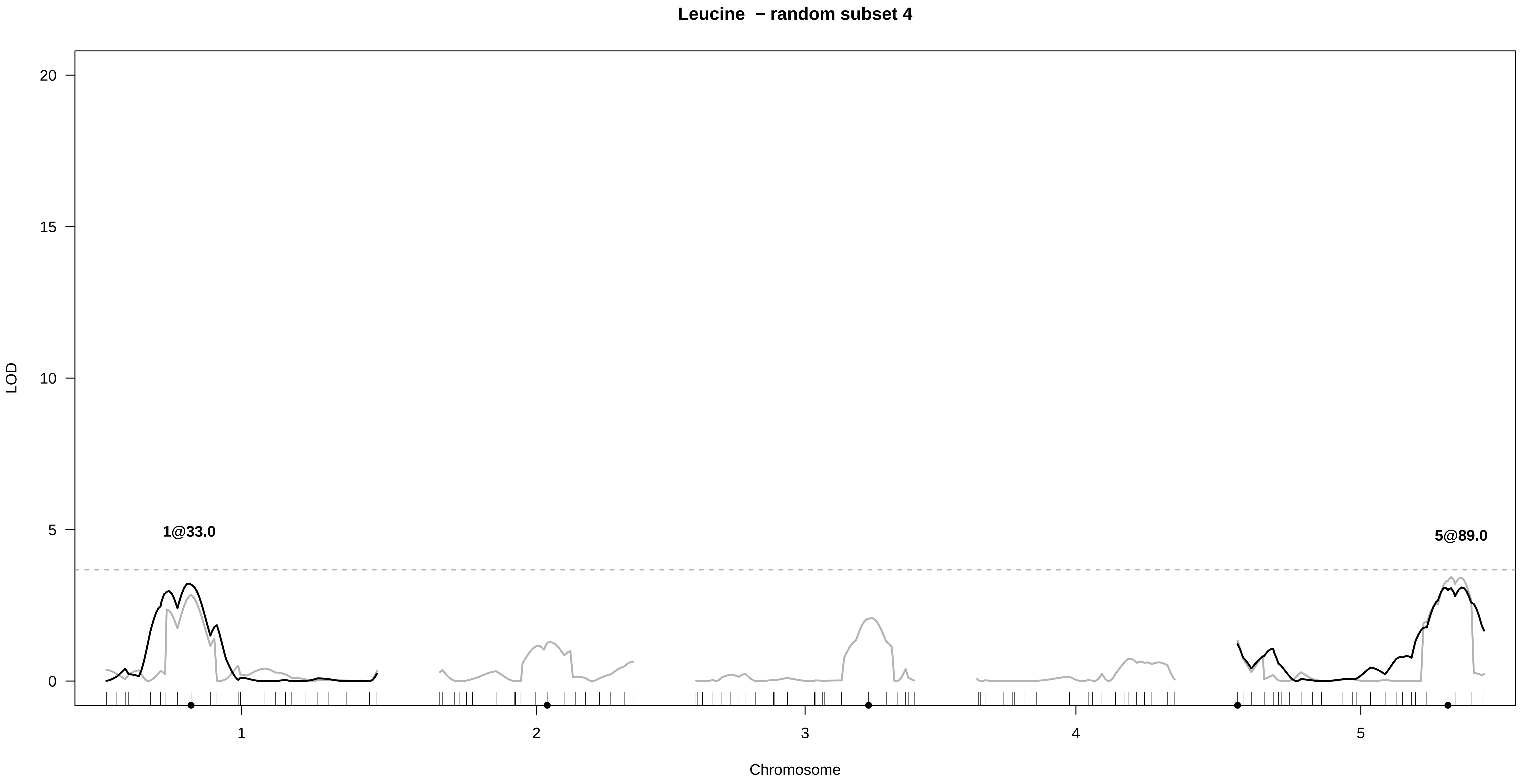

Leucine − random subset 4
LOD
20
15
10
5
0
1@33.0
5@89.0
1
2
3
4
5
Chromosome

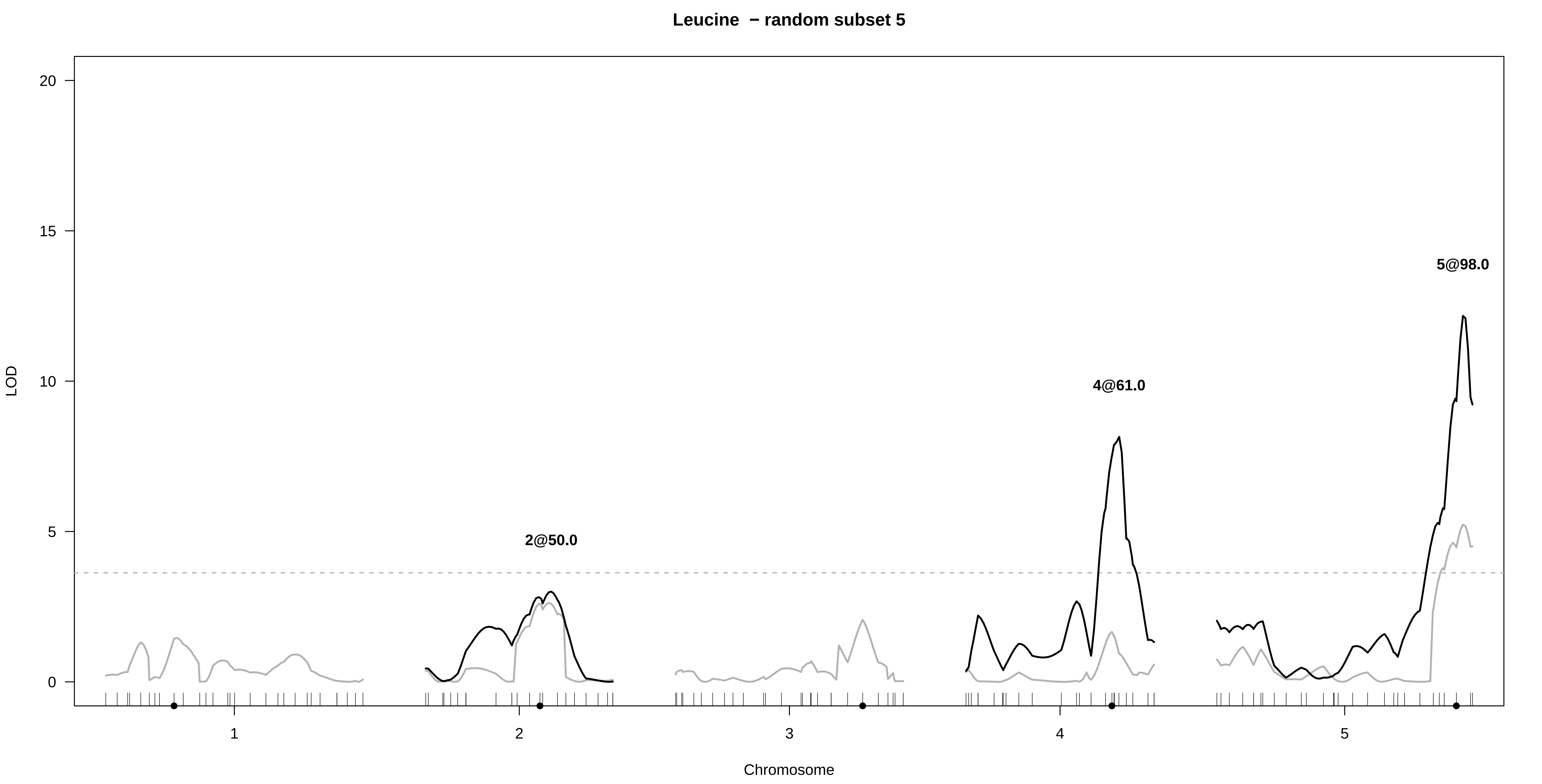

Leucine − random subset 5
LOD
20
15
10
5
0
2@50.0
4@61.0
5@98.0
1
2
3
4
5
Chromosome

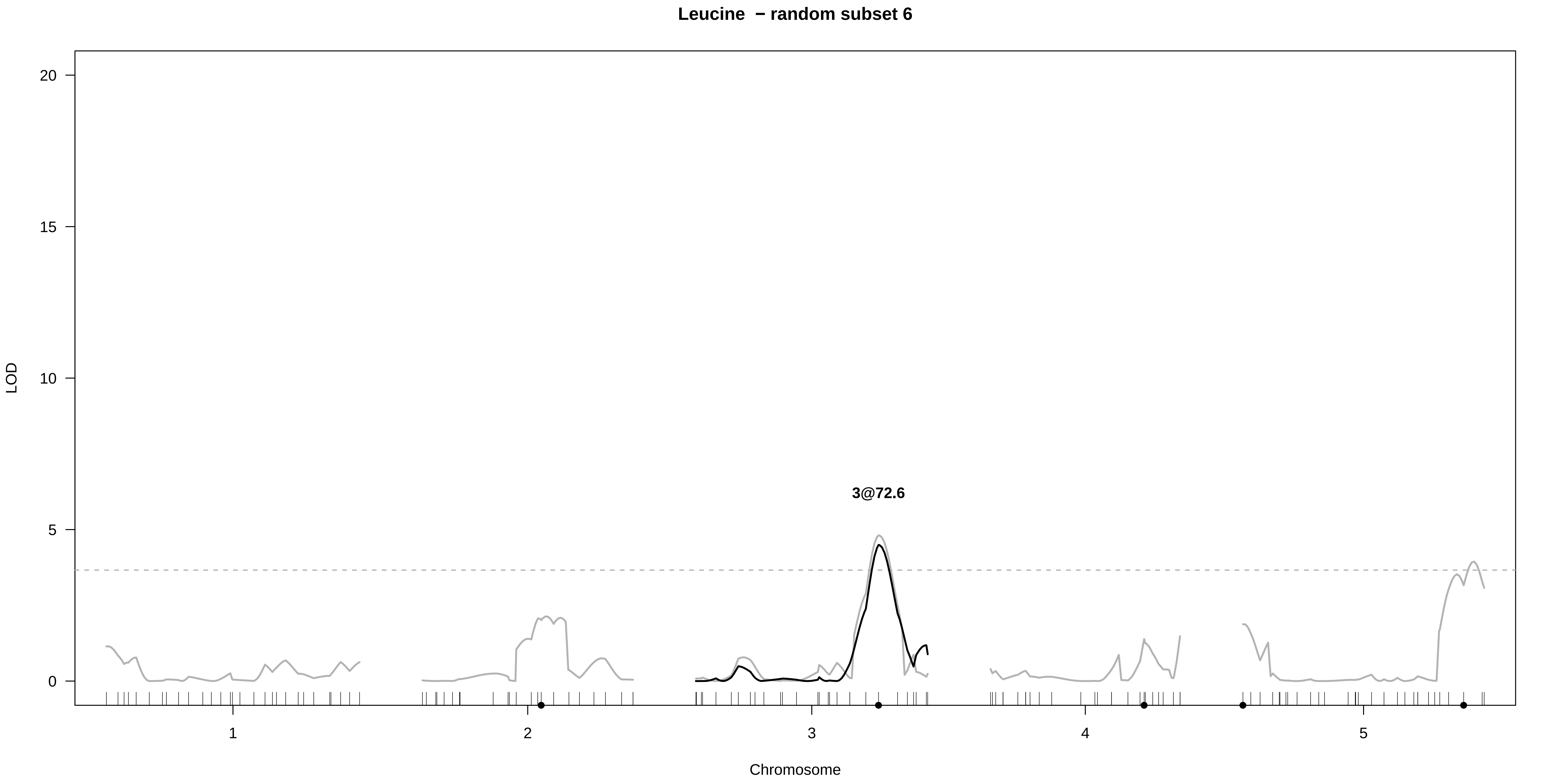

Leucine − random subset 6
LOD
20
15
10
5
0
3@72.6
1
2
3
4
5
Chromosome

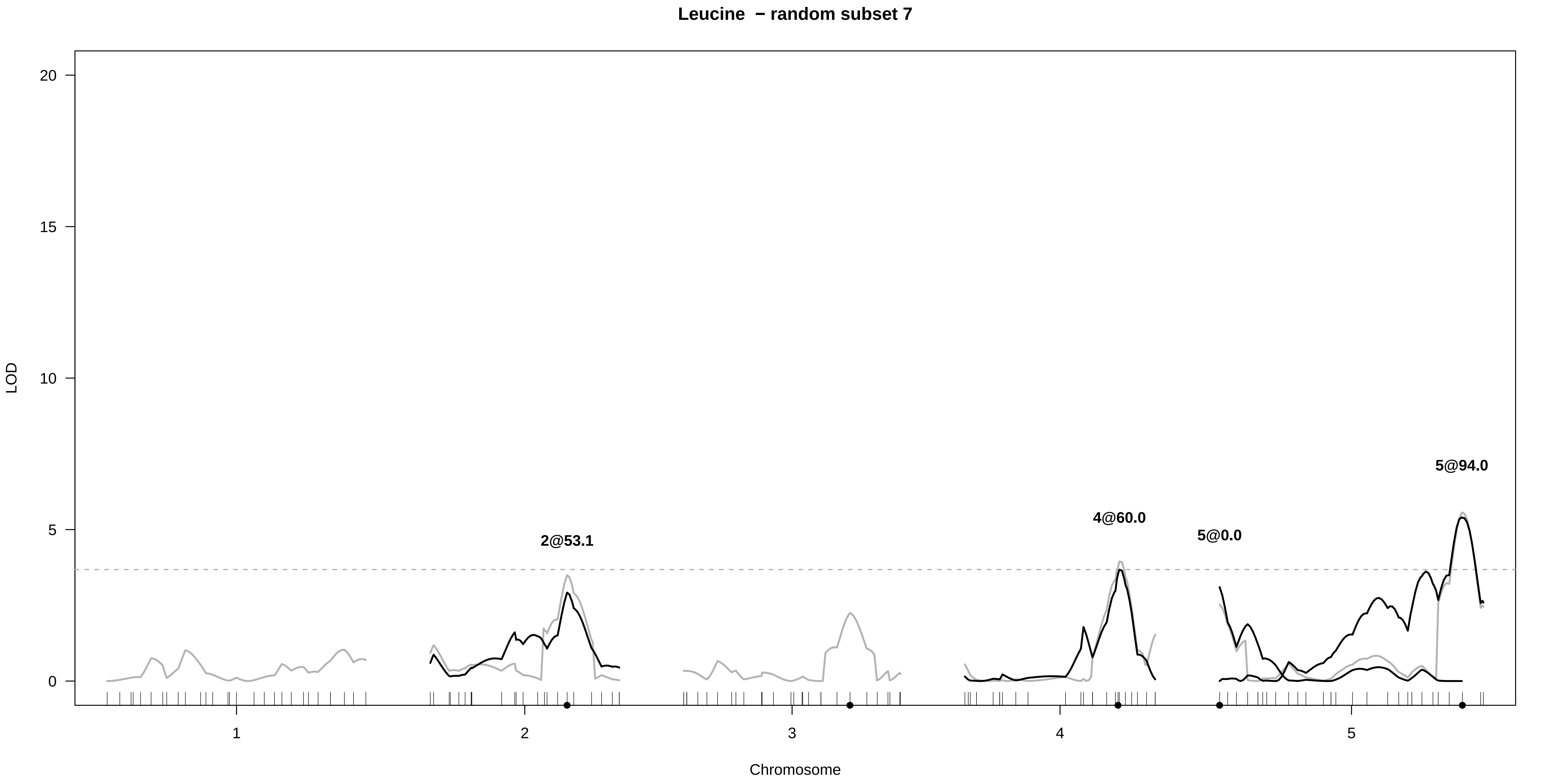

Leucine − random subset 7
LOD
20
15
10
5
0
Chromosome
1
2
3
4
5
2@53.1
4@60.0
5@0.0
5@94.0

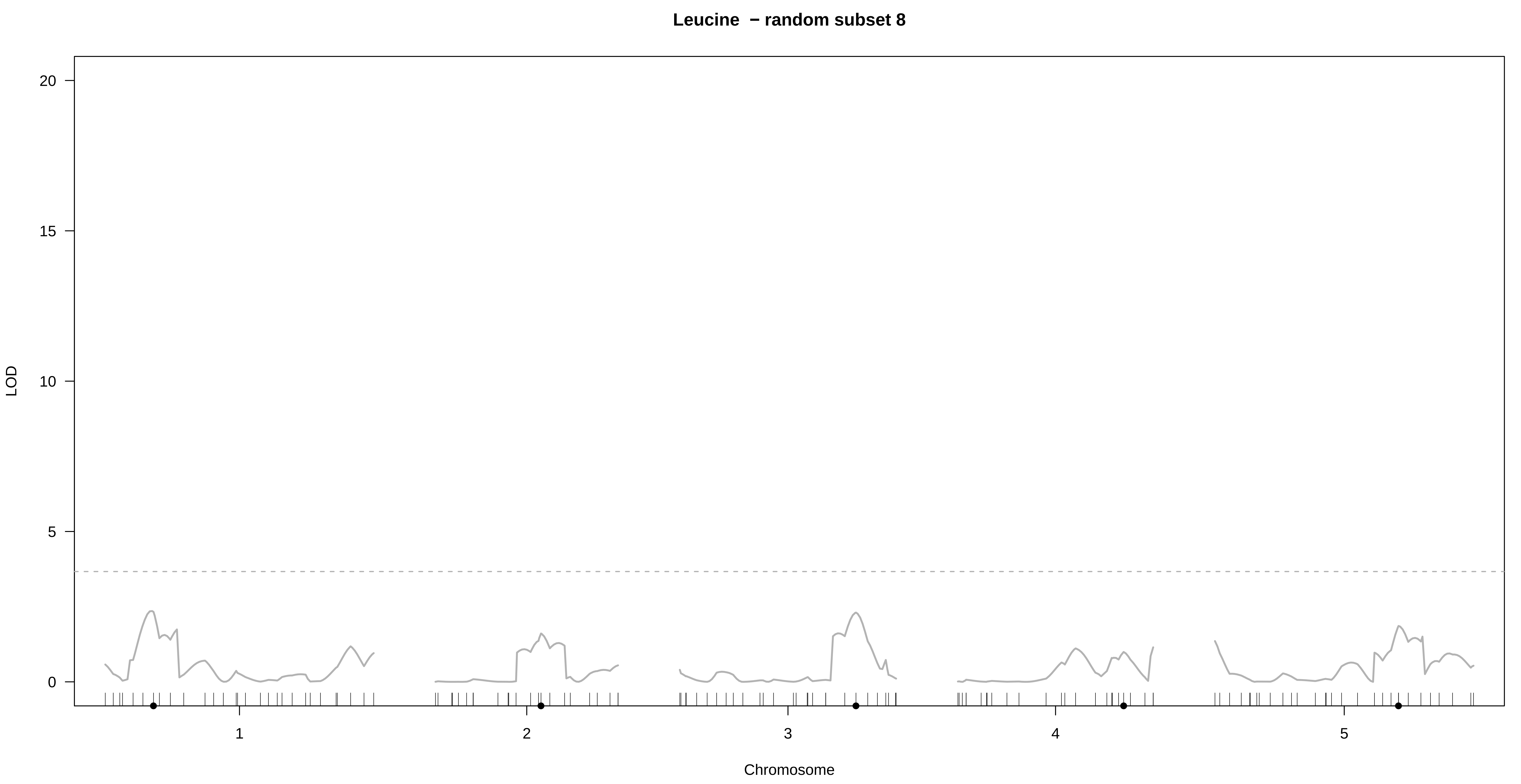
Leucine − random subset 8
LOD
20
15
10
5
0
Chromosome
1
2
3
4
5

Leucine − random subset 9

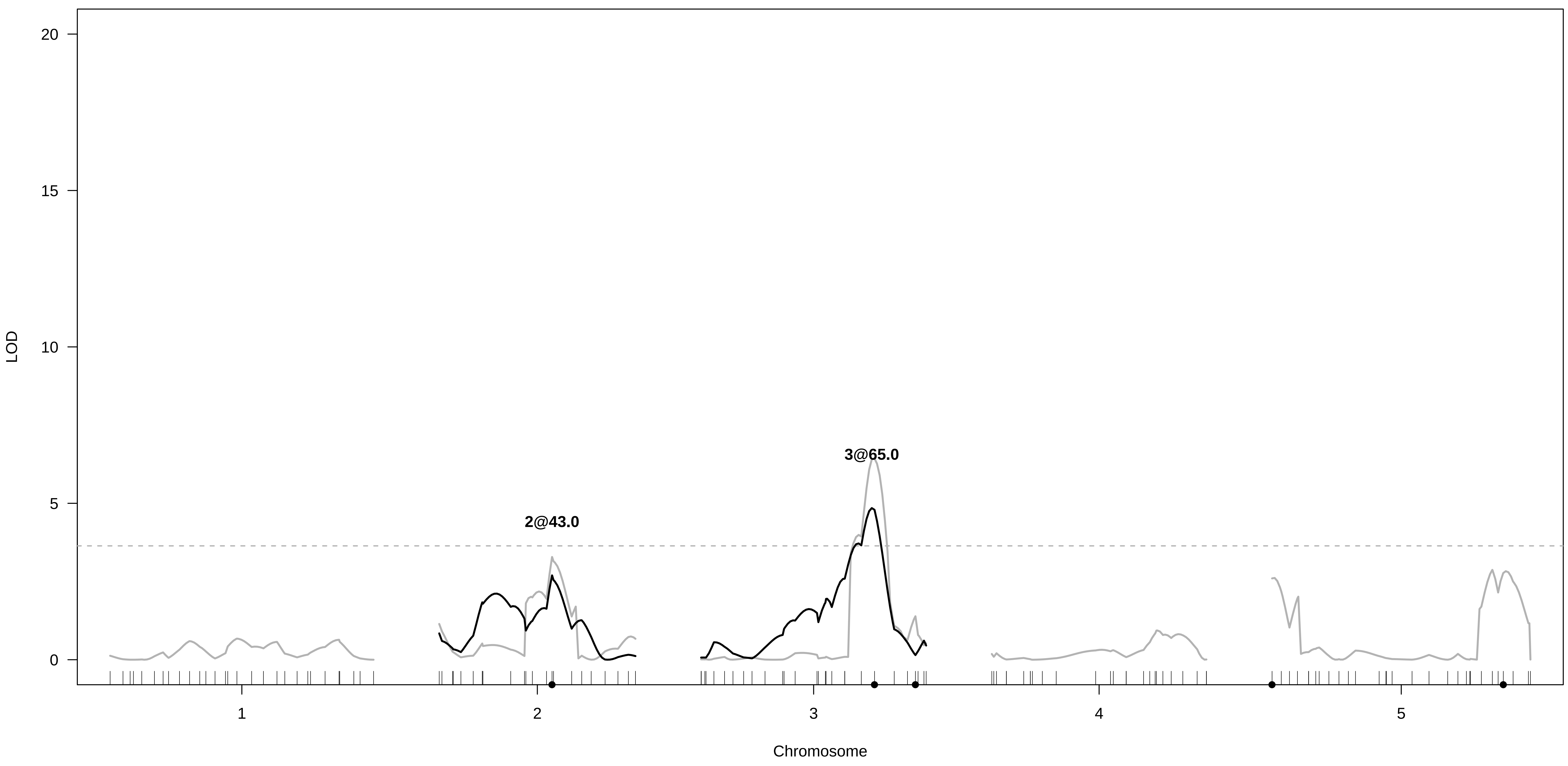

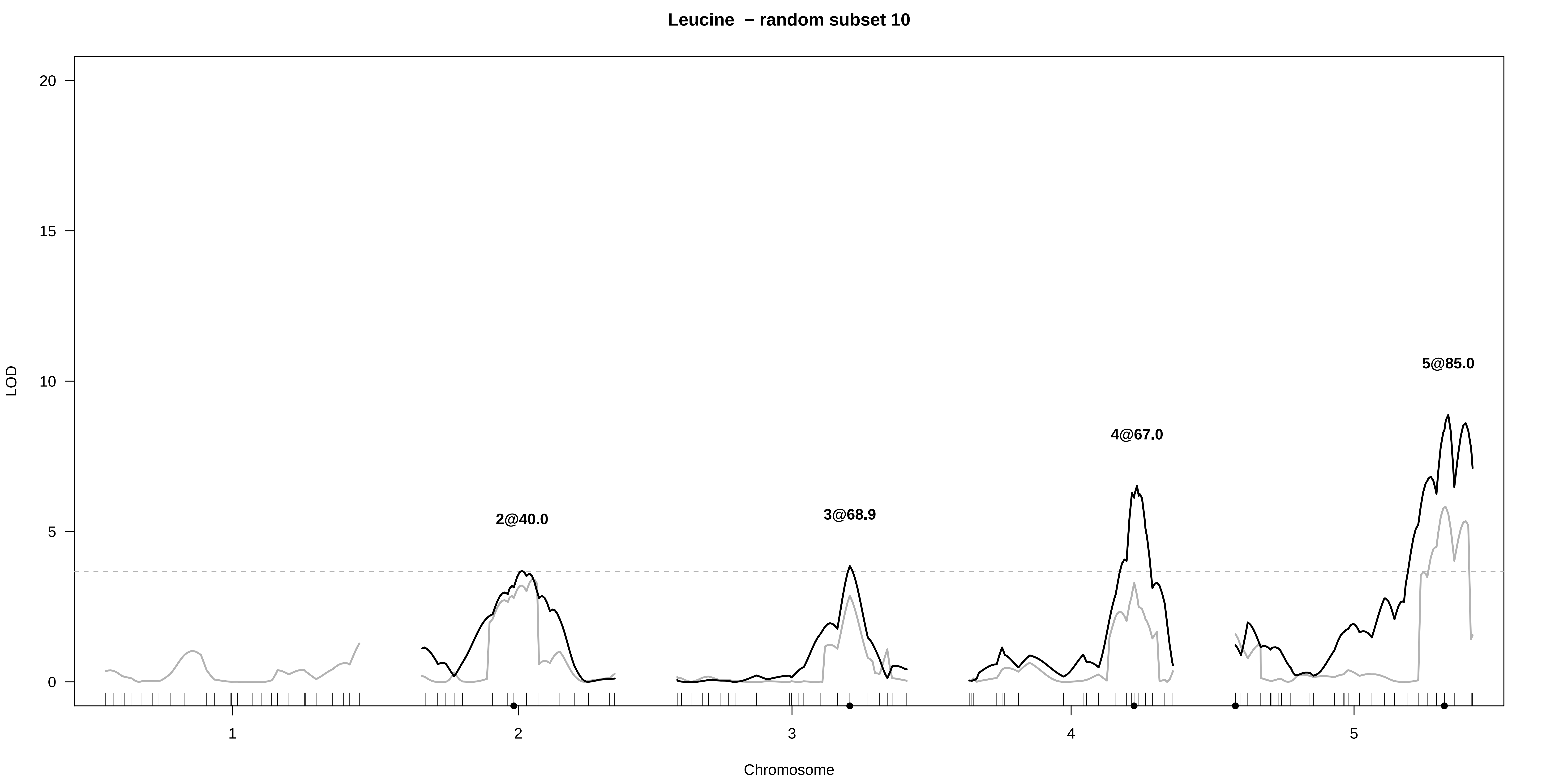

Leucine − random subset 10
LOD
20
15
10
5
0
2@40.0
3@68.9
4@67.0
5@85.0
1
2
3
4
5
Chromosome
