# Supplementary material for: Genetic dissection of metabolite variation in Arabidopsis seeds: evidence for mQTL hotspots and a master regulatory locus of seed metabolism
Source: J Exp Bot. 2017 Mar 6;68(7):1655–67. doi: 10.1093/jxb/erx049 (PMC5444479; doi:10.1093/jxb/erx049)
Supplement: Supplementary_Dataset_S10 [file erx049_suppl_Supplementary_Dataset_S10.pdf]

> *AT1G10070* (*BCAT2*) | Col-0 (TAIR10) vs. C24 (contig_chr1L_95)

ttactttcatatcctataaataatgttgcactctcttgatcacccatcaaaagcttttgaaatagtttttggtaaatt
tgtgacataaatttgtgatatatcaccacatcgtattagtattaaacatttgtttcactcttccaaagagacaaaagc
aaaactaaagaccttcaacttcaaggtcaggatatgtaatctaatggtttgtctttgtggcattttgccaccattggt
tcaaaataccattgctgtatgatcagcctcctcagattctatttgccagactctacggcggtatattcgcttttagaa
aaaaaacaatttgtcgaaacttttttttttttgtaaatattgtagattccacacacaagactgtctaagattctttg
ataagacacgacaaatgcgatttaagattttcaaagaaatgaggttcgttgattgttccactaggtttggtatattt
ataatttgaattttttttt**[tt;t]**aaagatatttataagaaaacgaaggagtgtgctcgcgtcaagtgtgtactaat
agtcgatgacactagtgatacgacgatactggaaa**[g;a]**ggacatcaatattcattagtaagccatcttagcaaaaa
cgttgttatcagataagaaaagttctcagactgacgtggctgtcaatctccacaacgtgtttatctcccatttgggta
gttacggacaaagacgttgaaaagacaacaatatggtctaaaagattgacatggaaagaaaattgtttatccgacaaa
gacctcttggtccaacgtggctttcacatcactagtttactatttcacatctcatggaaaattactgaaattagtact
tgtctgtattttgtttttcgtcaagtatttgtttgtatactttaggtaattgcgtgccgaagaaacattgctaaatt
gatatttataccaacttacaatagtttacagttcaaaaatagaaatctccatttttgaagatacaccactaattttctt
catttatttaccccataagagaacgagaaaagaac**[a;t]**atagtccgttgatttcgttaaaatctagtttcagaaag
acacgtctagatctgtctgatcagggcagatagacacaagtgacgaagcaaaagaaacacaaaaataagataagaact
cttaccactaaaagataaaaataagaaaaaaaaataaaacaaattcaacgatttgccaagataaaagcagacactgtac
ataagctccgca*ttcacttacgtgttggatacttaatttgacactcttctttgtgttttctcttccaccttcttttc*
ggtatatcattcaacaaaattctctcttcatttctttatttgttgttttttttattattattgaaatcaagagaatt
*aaaacagaggctctgcaaaagaag*ATGATCAAAACAATCACATCTCTACGCAAAACTCTGGTTCTACCTCTTCATTTA
CATATTCGTACGgtataaactggattcttacaatctaatgatgattacttacttagagacataactcggatctcagat
acaaacatgttttatgttctgtttttttgtcctgatgaaatagCTACAAACTTTCGCCAAGTACAACGCACAAGCTG
CATCGGCTTTGCGAGAAGAGCGTAAGAAACCTCTTTATCAgtaagttctctttttcatgatccagccaggtcatatag
atatttgttaccattggaaaaagctctgtttcaaatttgttgtaattgtatcatatagAAATGGAGATGATGTATAT
GCGGATTTGGATTGGGATAATCTCGGGTTTGGTCTAAATCCAGCTGATTACATGTATGTCATGAAATGCTCAAAAGAC
GGCGAATTCACTCAAGGAGAACTTAGTCCCTATGGGAATATTCAGCTAAGTCCTTCTGCTGGAGTCTTAAACTATGGA
CAGgcaaggatctcgtaacccccaaatgtttcttgatttgtttctcaactgtatgatcaatagaggttttgggattct
ttgcagGCGATATACGAAGGTACAAAAGCATACAGGAAAGAAAATGGGAAGCTTCTTTTGTTTCGTCCGGATCACAAC
GCTATCCGGATGAAGCTTGGCGCTGAACGGATGCTCATGCCTTCTCCTTCGGTTGATCAGTTTGTTAATGCAGTTAAA
CAAACCGCTCTTGCAAACAAACGTTGGgtaaaaacgctttcttgtgacgtatatttcttctgaagaggtttgtttt**[t**
;a]gggatctgat**[a;g]**agtgatgatgtgtttgttcagGTTCCTCCTGCAGGGAAAGGGACTTTGTACATTAGGCCT
TTGTTGATGGGAAGTGGTCCAATACTTGGTTTAGGTCCTGCACCTGAATATACATTCATTGTCTATGCATCTCCAGTT
GGTAACTACTTCAAGgtatatacaatactcttgcatgtccatgctttttggcgatggaacgattagcaaatgttttgt
tcactgatttgtagGAAGGGATGGCTGCTCTTAACCTCTATGTTGAGGAAGAATATGTCCGAGCGGCTCCTGGTGGAG
CTGGAGGCGTCAAGAGCATCACAAATTATGCGCCAgtaagtgttgaaaccaacttagtttctcaaagaccagagtcag
aaaaagagttttgataaaccacgtgtttgtagGTTTTGAAAGCACTGAGCAGAGCCAAGAGTCGGGGGTTTTCAGACG
TTCTTTATCTCGACTCTGTCAAGAAGAAGTACTTAGAGGAGGCTTCTTCTTGCAACGTCTTTGTTGTCAAGgtaagat
atattgtggtgtctccatgtctctttgttgcatcctaattgtatcattgattcagGGTCGGACAATCTCAACTCCTGC
AACTAATGGAACAATTCTTGAAGGGATTACGCGGAAAAGTGTGATGGAGATCGCAAGTGATCAAGGTTATCAGgtaac
acagataagattga**[a;t]**tcactctaaacacccaaaaactaaactcagctaagtttcacacctgagttatgatcagG
TAGTAGAGAAGGCAGTTCATGTGGATGAAGTAATGGATGCAGATGAAGTTTTTTGCACCGGAACTGCTGTAGTAGTTG
CTCCCGTGGGCACTATCACATATCAGGA**[A;C]**AAAAGgtaaatctcaatgcctgaatcaagttttactcaagcctta
agcaaagaaatggactgatgtaatctttctgaaacattcagAGTAGAGTATAAAACCGGGGATGAATCTGTCTGCCAG
AAACTGCGTTCAGTCCTCGTAGGTATCCAGACAGGATTGATTGAAGATAACAAGGGATGGGTCACAGATATCAACTGA
agaataacaatcttcaatatttgccacaaaggagattactctgcattttaagctgtgtatacacgacaatgaagaac
actataaacatatatatgagatagaaacagaaagctcttttgctcatttctatatcgtaaagtcaatgtctttcacaa
aactatgacaaagaaaagaatcgtctattgtacttgtagacaagtgtttccaaggatagtttcatttcctcctgcagt
aactccaaatttacatttcattttaggaggcaaaactgtatatacgtttcaatggctttccccacttcttactttcat
*cacccataagggtttcaactacta*aaattgtaactcaactgcaaaaacaccttttcatctgcagagttataaagtata
cattgtaactcaagtat

> *AT4G02640* (*bZIP10*) | Col-0 (TAIR10) vs. C24 (contig_chr4L_77)

attcttttggttcgttctctattgtaatatagcctacatagtggtgatttgtggtattggtgacactatgttaaggtc
aaaaagaaatagagagtaaattccatatatatgaaaattgaaaatgactgtttaaatttacatatggcgcgaaaaaat
gatctagttcgttaattatttttagaagctttttgt**[g,agattatt]**gtaaatttagattttt**[a,t]**aaaaactcc
atat**[at,tg]**ttttttttt**[a,t]**taatttaggcttgtctattgtaaatttgtaatatagcttagtgatttatttgt
ttagcttctggtgacattttataaatttttaatgaccagtataatatagtatttt**[t,g]**ttataaatta**[a,g]**aat
ataatctaatgttttatgtcaacttcgttgttcattgaataaatcaaactttgaagaataattaaataacaatgtttg
caaaaactgttacgtttttttcatgtaaacattagtggacgttatacaatagatatttaaattataaaaactattcaaa
gaatattgtaaagaaataaaacagaaatgtgtaagaaaaaatgtttattttattgtacgtaataaaaaaagagaatat
acttgtgtgggtggacctaaatgcaaataggcaaagtaccgacaaaaa*aagaagaatcagaaagtgaaaaagagagcg*
*agcg*ATGAACAGTATCTTCTCCATTGACGATTTCTCCGATCCTTTCTGGGAAACTCCTCCGATTCCTCTCAATCC**[C,**
T]GACTCTTCTAAGCCTGTTACGGCGGATGAAGTTAGCCAGAGTCAACCGGAATGGACTTTCGAGATGTTTCTCGAAG
AGATTTCTTCGTCGGCGGTGAGCTCTGAGCCACTTGGTAACAACAACAACGCGATCGTCGGTGTTTCTTCGGCGCAAT
CTCTTCCTTCTGTTTCCGGACAGAATGATTTCGAGGATGATAGTCGATTTCGTGATCGCGATTCGGGAAATTTGGATT
GTGCTGCTCCCATGACGACGAAGACGGTGATTGTTGATTCCGATGATTATCGTCGTGTTCTTAAGAACAAGCTTGAGA
CTGAGTGCGCTACTGTTGTTTCTCTTCGGgtgtgttgttaatcttctatttttcttatctatatgtgtaatttgtgcg
ttttgatctaaatctgaagctctgatgttctcttcttagGTTGGGTCTGTGAAGCCTGAAGATTCGACTAGTTCTCCA
GAAACTCAACTTCAACCAGTTCAATCCAGTCCTCTTACTCAAGgtaagag**[t,c]**ctcttcttagtttcaccaggata
gatgagcgatgataaacctgcagattcgtttctctgttctgtgtttgttaatttagatgcctaggttaaggaattaga
aatagaaaatggtacttttgttggatcaagcgcctagttttatccgtcaagtttcaatctttttgtgactgactttct
cattgaaattaaactgctttctggaagaacaataagcttgggattgagatgagttctggtggttagttcaatatatgg
gaaatcattatttcagcgctcgtctttctctttcagatatctcttctgagtcttagacatagtaagttttaatcgtgg
tagGTTCTTTGATGACCCCAGGAGAACTTGGTGTTACTTCTTCCTTACCAGCTGAGGTGAAAAAAACTGGTGTATCAA
TGAAGCAGGTTACTAGTGGATCGTCGAGAGAATATTCTGATGACGAGGACCTTGATGAAGAGAATGAAACCACCGGTT
CCTTGAAGCCAGAGGACGTTAAAAAATCTAGAAGgtaatcattactttttctccaatttcttgtcgtttgaagattac
atttatagagccttgttcttccttttaatatttgtagGATGCTGTCAAATCGTGAGTCAGCTAGGCGATCTAGAAGGA
GAAAGCAGGAGCAAACAAGTGACCTCGAAACACAGgtttgtgttgtatcctctcaacacctcattttgtgttgaag
gatattaatgagacctaatttgtattttgatagGTTAATGATCTAAAAGGTGAGCATTCATCACTTCTTAAACAACTG
AGCAACATGAATCACAAGTATGACGAGGCTGCTGTTGGCAATAGAATACTAAAGGCTGACATTGAGACATTAAGAGCT
AAGgtaagaagacttaagtattatcctcctctcctttttctgaactacccttttttttgtatacagttttagtaactcc
catgttatgttaagGTGAAAATGGCGGAAGAAACCGTGAAGAGAGTAACAGGAATGAATCCGATGCTTCTCGGAAGAT
CAAGTGGACATAACAACAACAACAGAATGCCAATAACTGGTAACAACAGGATGGATTCTTCTAGCATTATTCCAGCTT
ATCAACCACACTCAAACCTAAACCA**[T,C]**ATGTCAAACCAAAACATCGGGATCCCAACCATTCTACCTCCAAGACTC
GGAAACAATTTCGCTGCTCCTCCATCCCAAACCAGCTCTCCCTTGCAGAGAATTAGAAATGGGCAAAATCACCATGTT
ACTCCAAGCGCCAACCCGTATGGCTGGAATACCGAACCTCAGAACGATTCAGCATGgtaagctaaaacatggctcttt
cttttaagaccattgaacaatttaaaaccatttgaagtactatgatccctgtttatacatatcagattcctgtctccc
ctttttttgctcattttacagGCCGAAAAAATGCGTGGACTGA*tcaaacaagaagcgggtttcgcactatattaatgt*
ctatgcatctgtaatttgtaagtgttattaagttacgaatcatgagaaaacatcttgtgaaaatacagtctcatggct
[ta,]*tatatatatataagctctgtcttataacattacaagattcttatttgagaatcgtctttctatttatagctaa*
*taacagagtgtatatctgtttttatccaattttaaaaaagata****[g,t]****ctc*acatggctttactaatttcccctatca
ttaaattccgacaacctcggcgatttccatggtagatcgatcaagcattcacattttctcgtcagaaaacac**[a,]**aa
aaaaaaaaacagaggagaaaaagagacgacgacaaacaagtgaaagaaaaaagtcgtcgttcttccaatttttttctc
ctctctcttcctctgtttaacggcgtacgtaagtcaaa

Legend:

START to STOP codon in grey
Exons in capital letters
UTRs in italic
Polymorphism in square brackets and red: [Col-0;C24]
