# Supplementary material for: Genetic dissection of metabolite variation in Arabidopsis seeds: evidence for mQTL hotspots and a master regulatory locus of seed metabolism
Source: J Exp Bot. 2017 Mar 6;68(7):1655–67. doi: 10.1093/jxb/erx049 (PMC5444479; doi:10.1093/jxb/erx049)
Supplement: Supplementary_Dataset_S12 [file erx049_suppl_Supplementary_Dataset_S12.pdf]

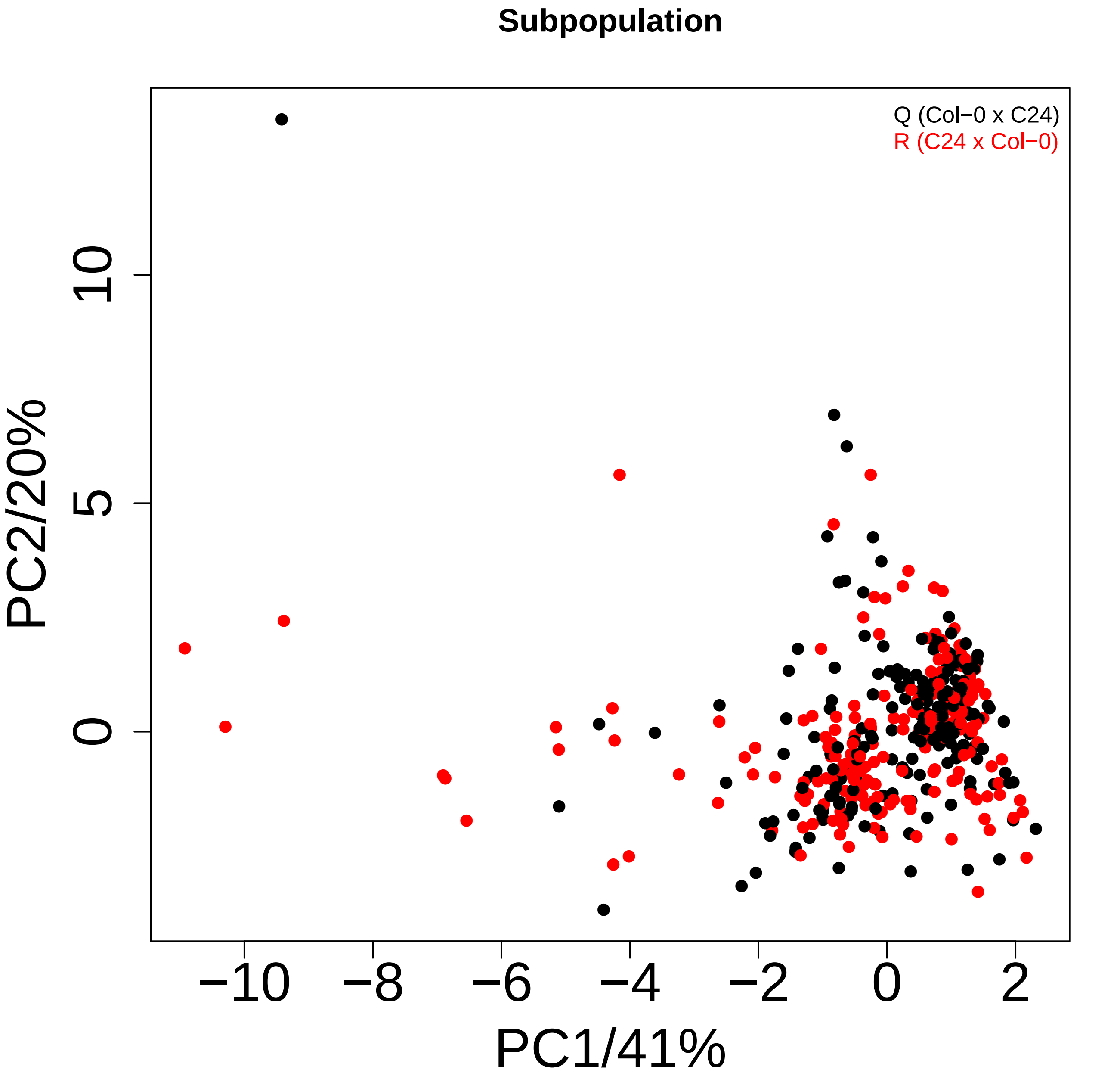
Subpopulation
Q (Col−0 x C24)
R (C24 x Col−0)
PC2/20%
10
5
0
−10
−8
−6
−4
−2
0
2
PC1/41%

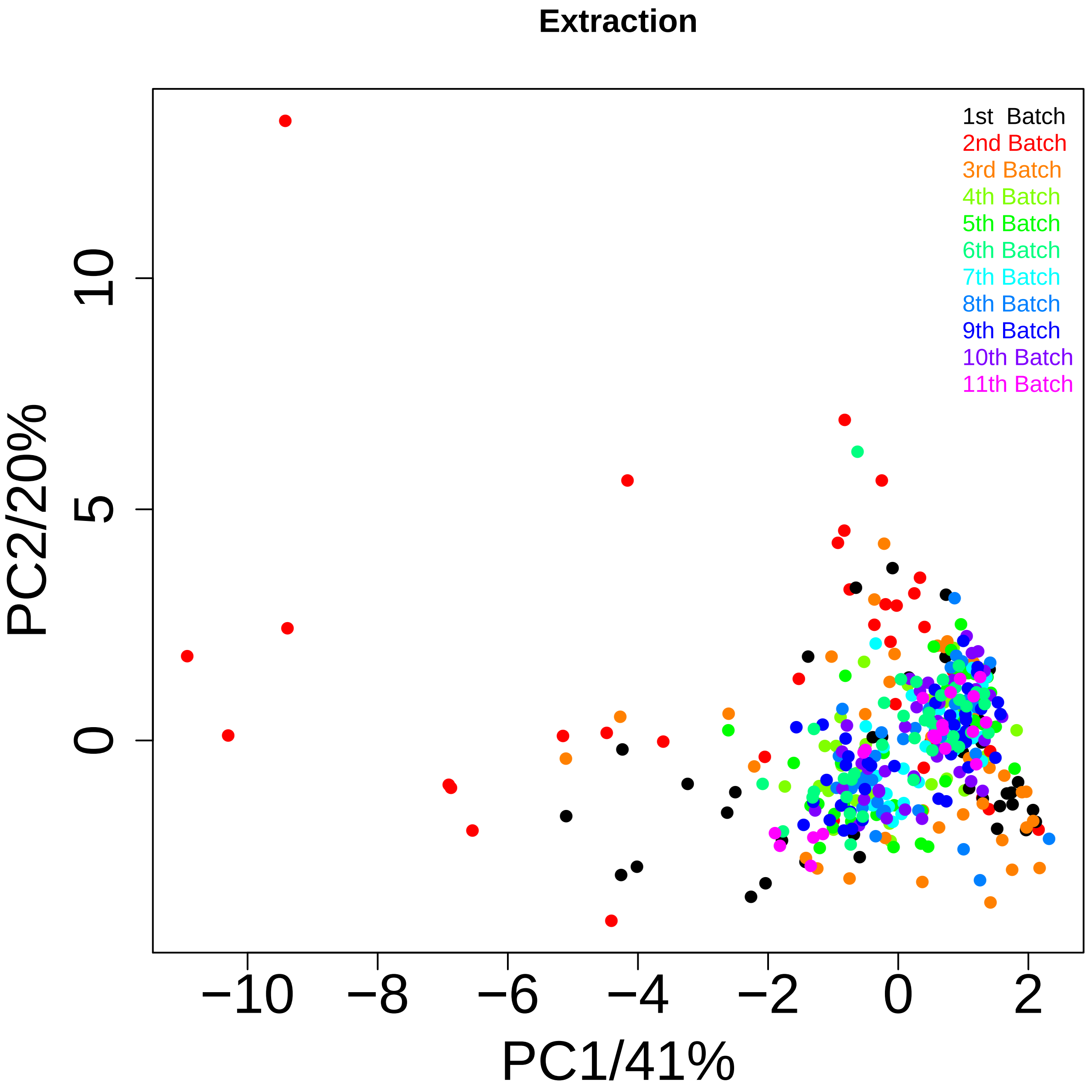

Extraction
1st Batch
2nd Batch
3rd Batch
4th Batch
5th Batch
6th Batch
7th Batch
8th Batch
9th Batch
10th Batch
11th Batch
PC2/20%
PC1/41%
10
5
0
−10
−8
−6
−4
−2
0
2

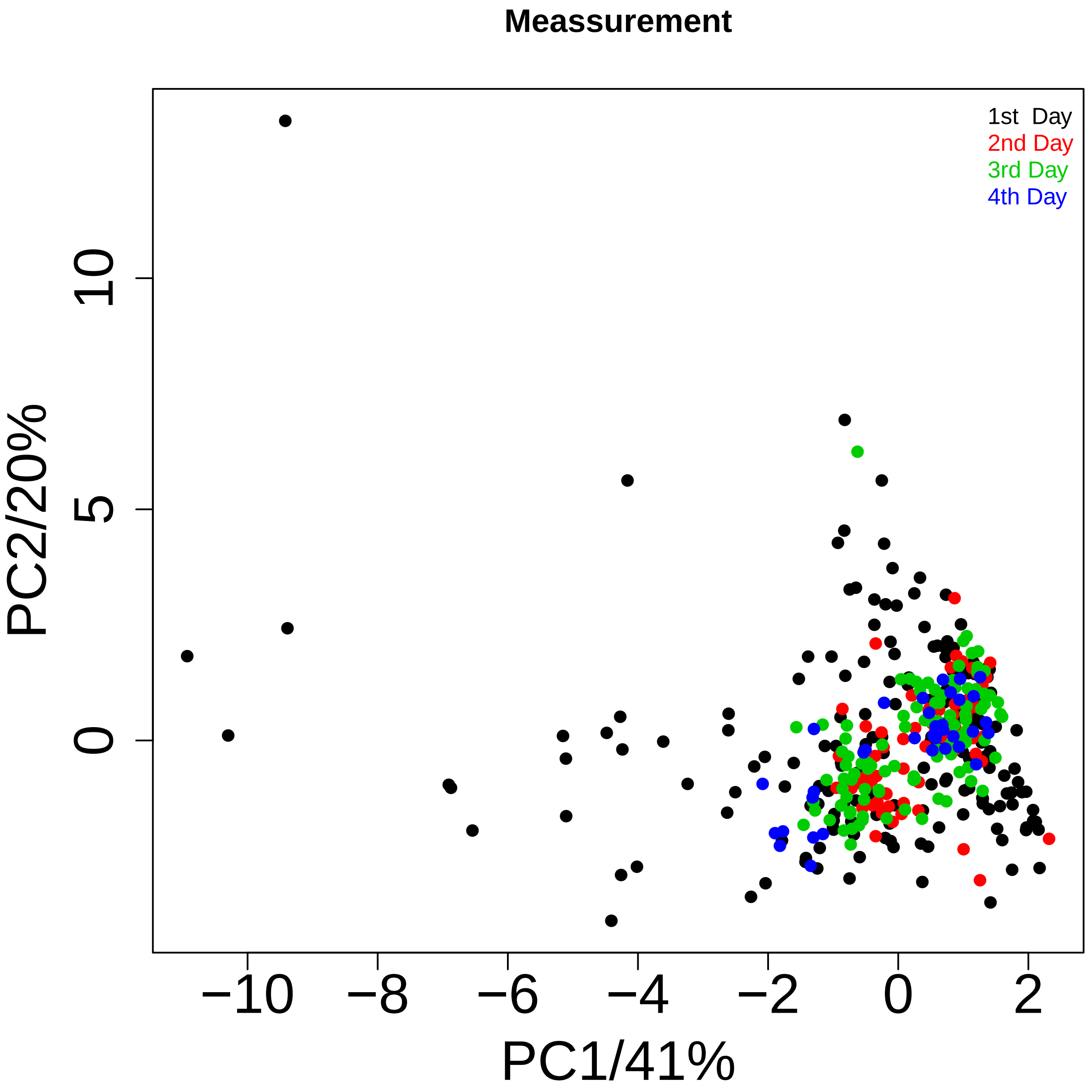
Meassurement
1st Day
2nd Day
3rd Day
4th Day
PC2/20%
PC1/41%
10
5
0
−10
−8
−6
−4
−2
0
2

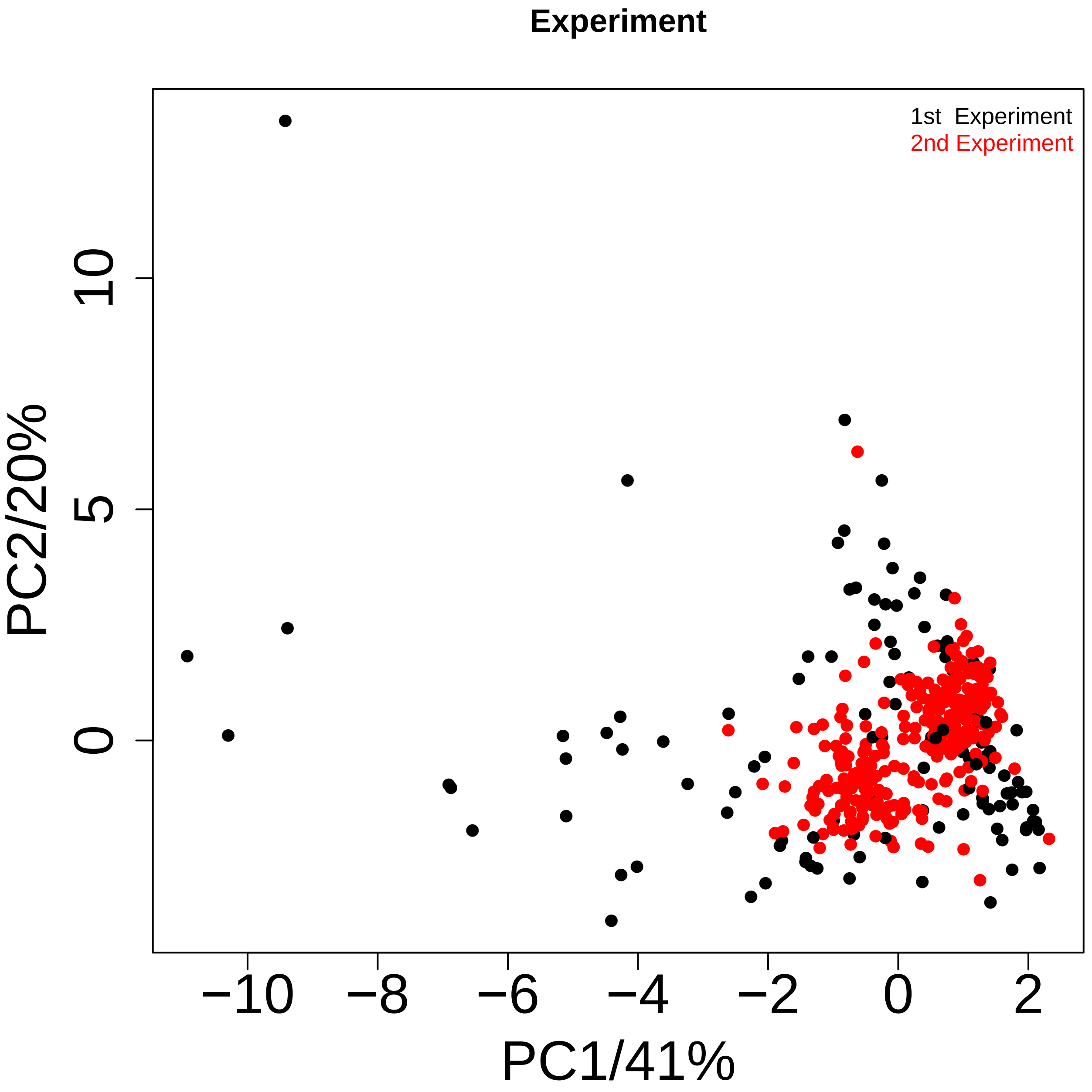
Experiment
1st Experiment
2nd Experiment
PC1/41%
PC2/20%
−10
−8
−6
−4
−2
0
2
0
5
10

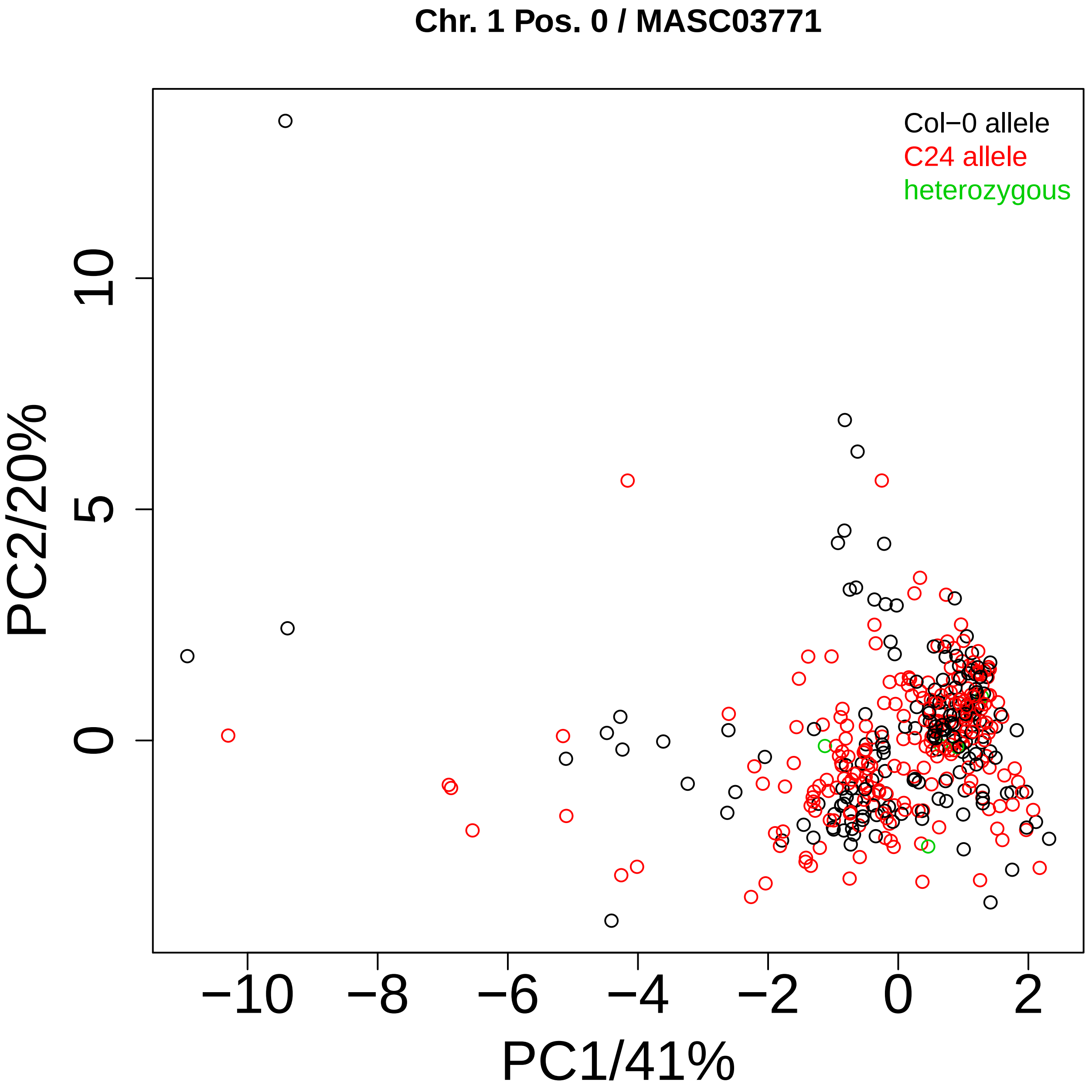

Chr. 1 Pos. 0 / MASC03771
Col−0 allele
C24 allele
heterozygous
PC1/41%
PC2/20%
−10
−8
−6
−4
−2
0
2
0
5
10

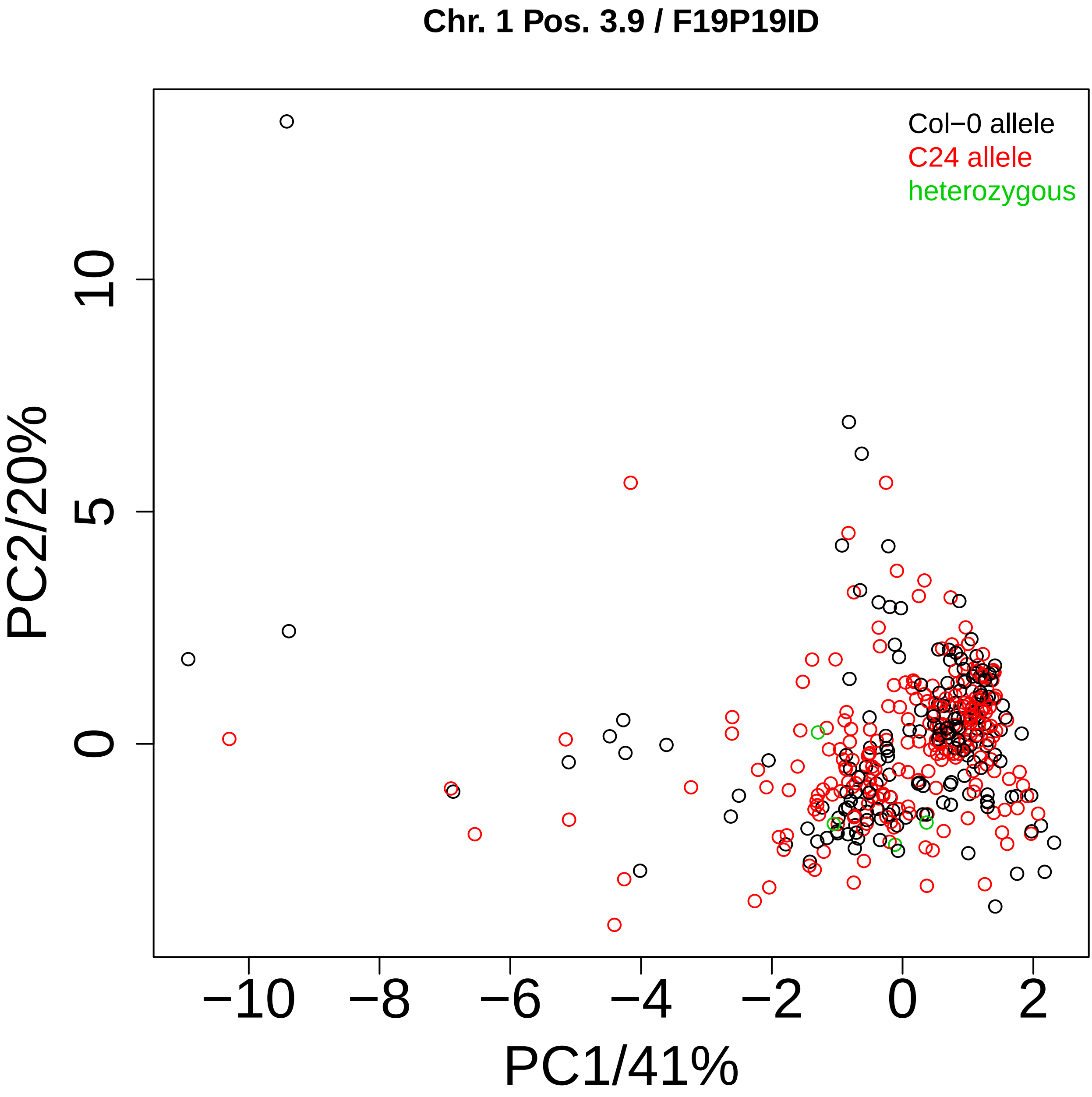
Chr. 1 Pos. 3.9 / F19P19ID
Col−0 allele
C24 allele
heterozygous
PC2/20%
0
5
10
PC1/41%
−10
−8
−6
−4
−2
0
2

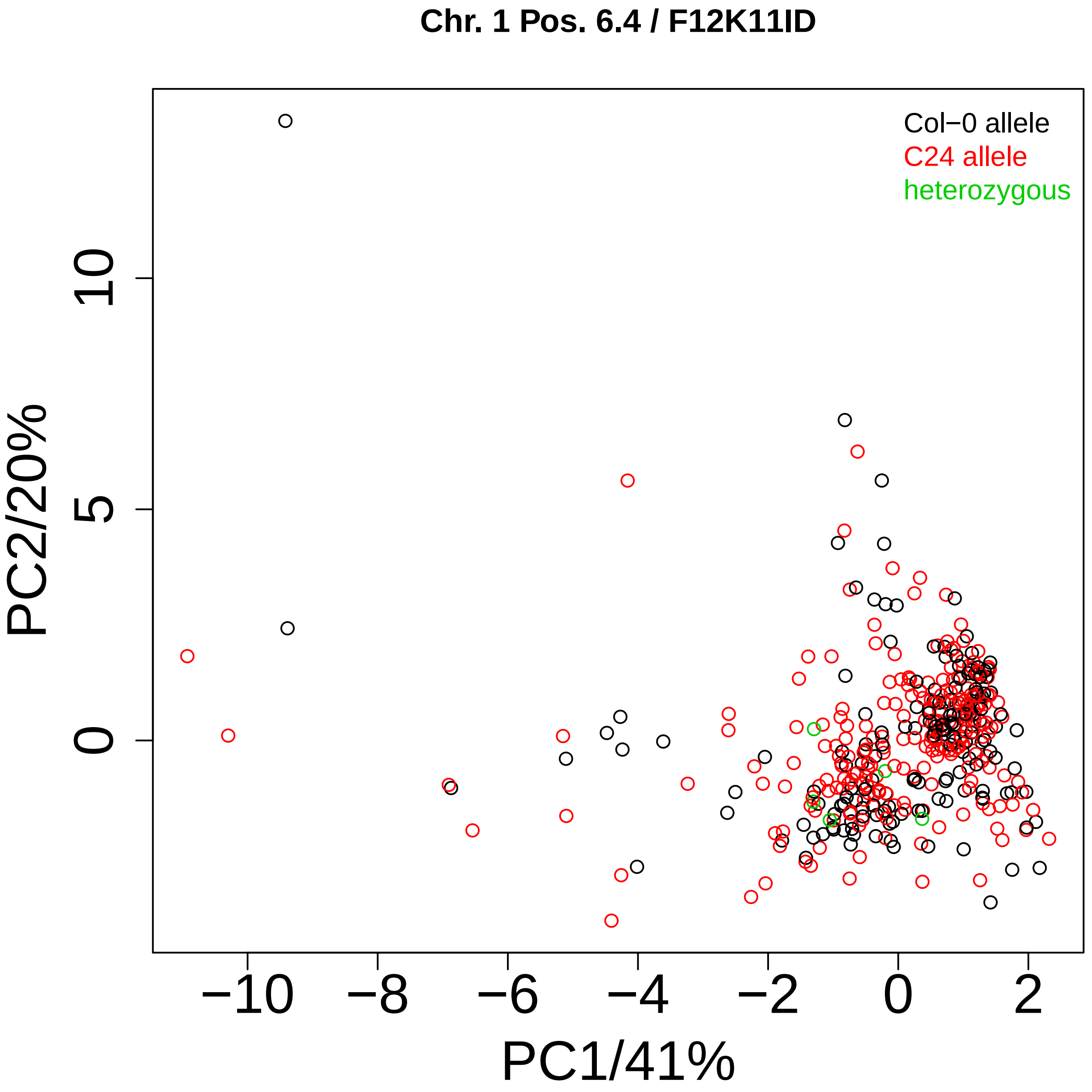

Chr. 1 Pos. 6.4 / F12K11ID
Col−0 allele
C24 allele
heterozygous
PC2/20%
PC1/41%
10
5
0
−10
−8
−6
−4
−2
0
2

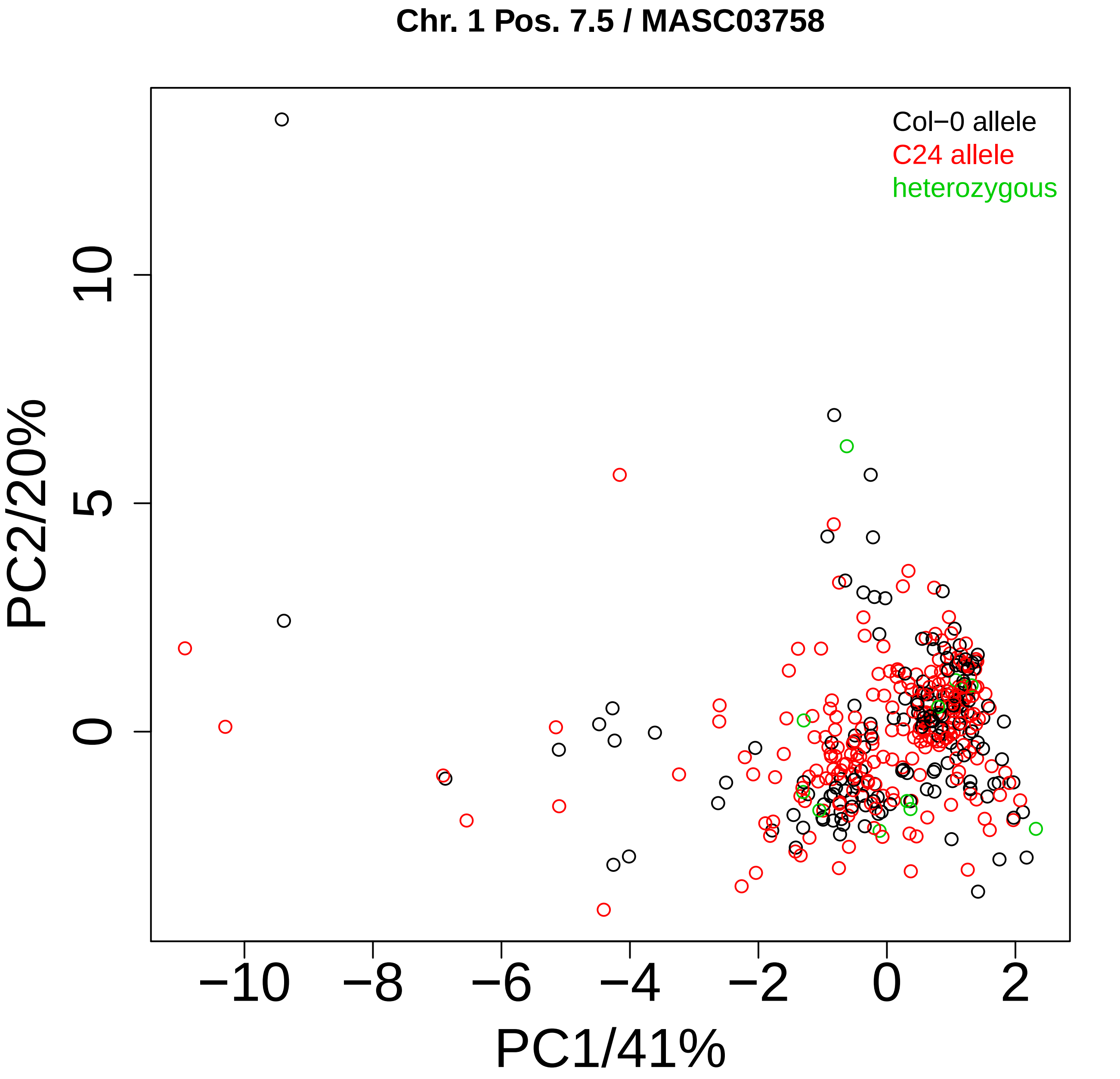
Chr. 1 Pos. 7.5 / MASC03758
Col−0 allele
C24 allele
heterozygous
PC2/20%
PC1/41%
10
5
0
−10
−8
−6
−4
−2
0
2

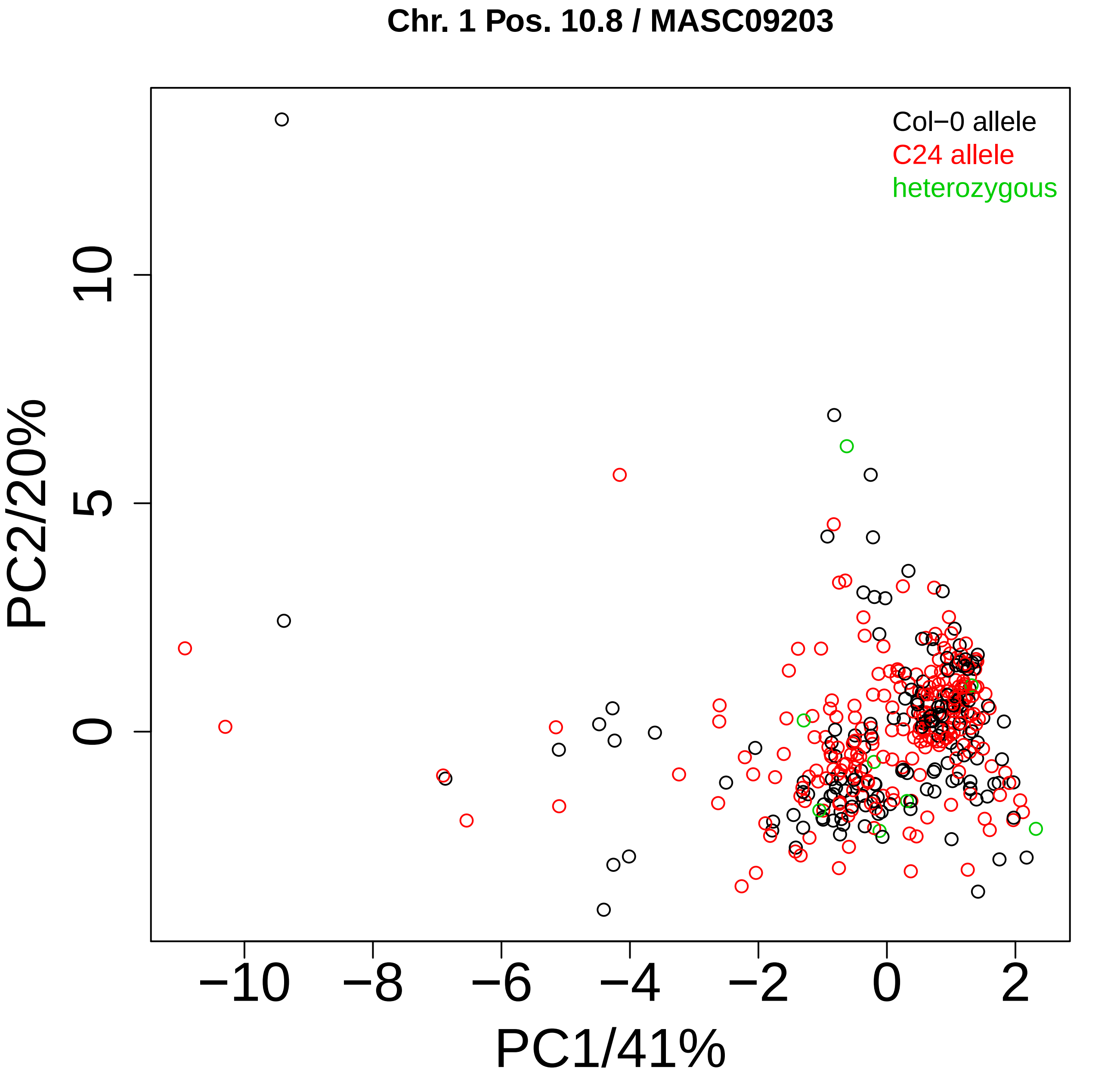
Chr. 1 Pos. 10.8 / MASC09203
Col−0 allele
C24 allele
heterozygous
PC2/20%
PC1/41%
10
5
0
−10
−8
−6
−4
−2
0
2

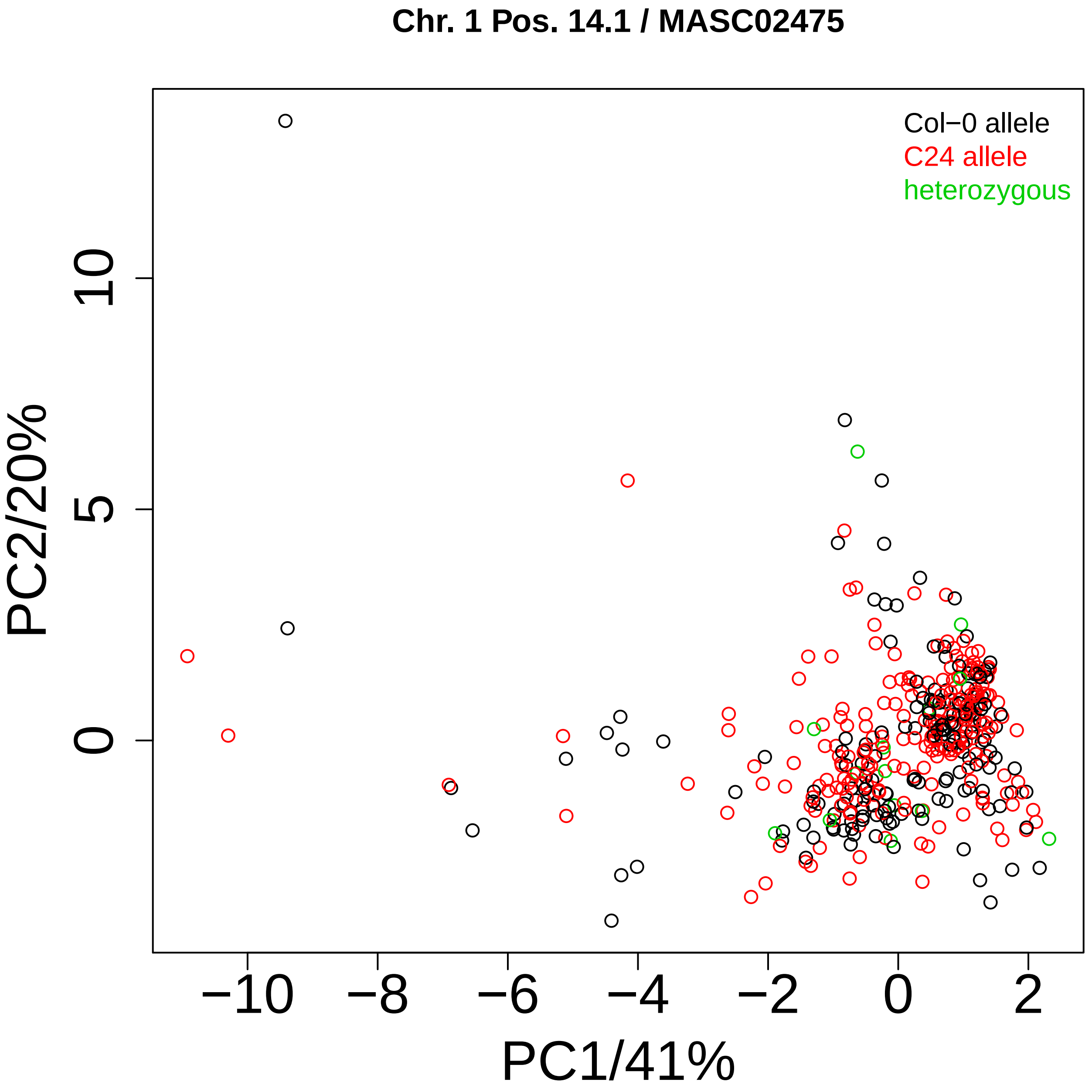
Chr. 1 Pos. 14.1 / MASC02475
Col−0 allele
C24 allele
heterozygous
PC2/20%
PC1/41%
10
5
0
−10
−8
−6
−4
−2
0
2

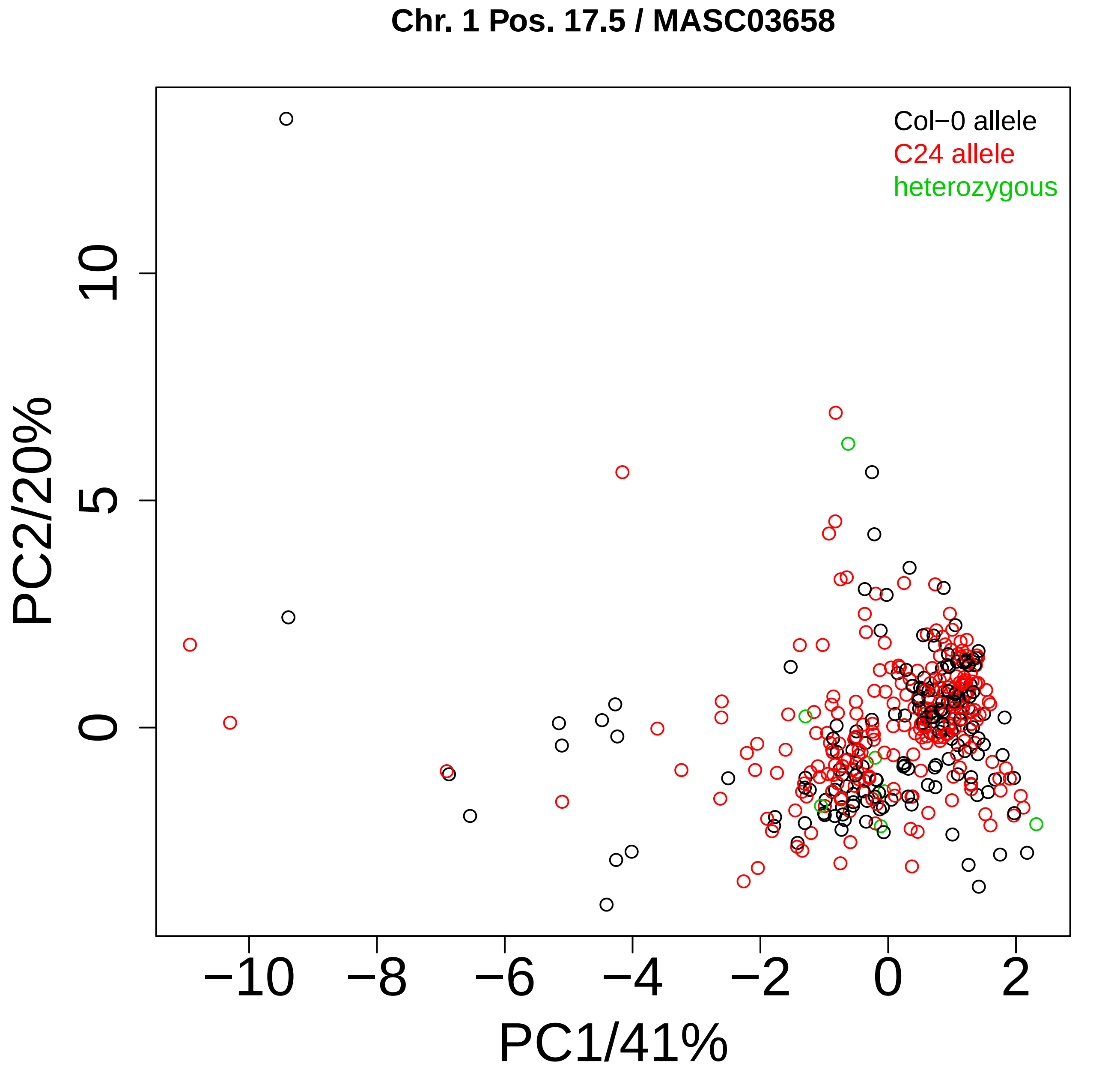

Chr. 1 Pos. 17.5 / MASC03658
Col−0 allele
C24 allele
heterozygous
PC2/20%
0
5
10
PC1/41%
−10
−8
−6
−4
−2
0
2

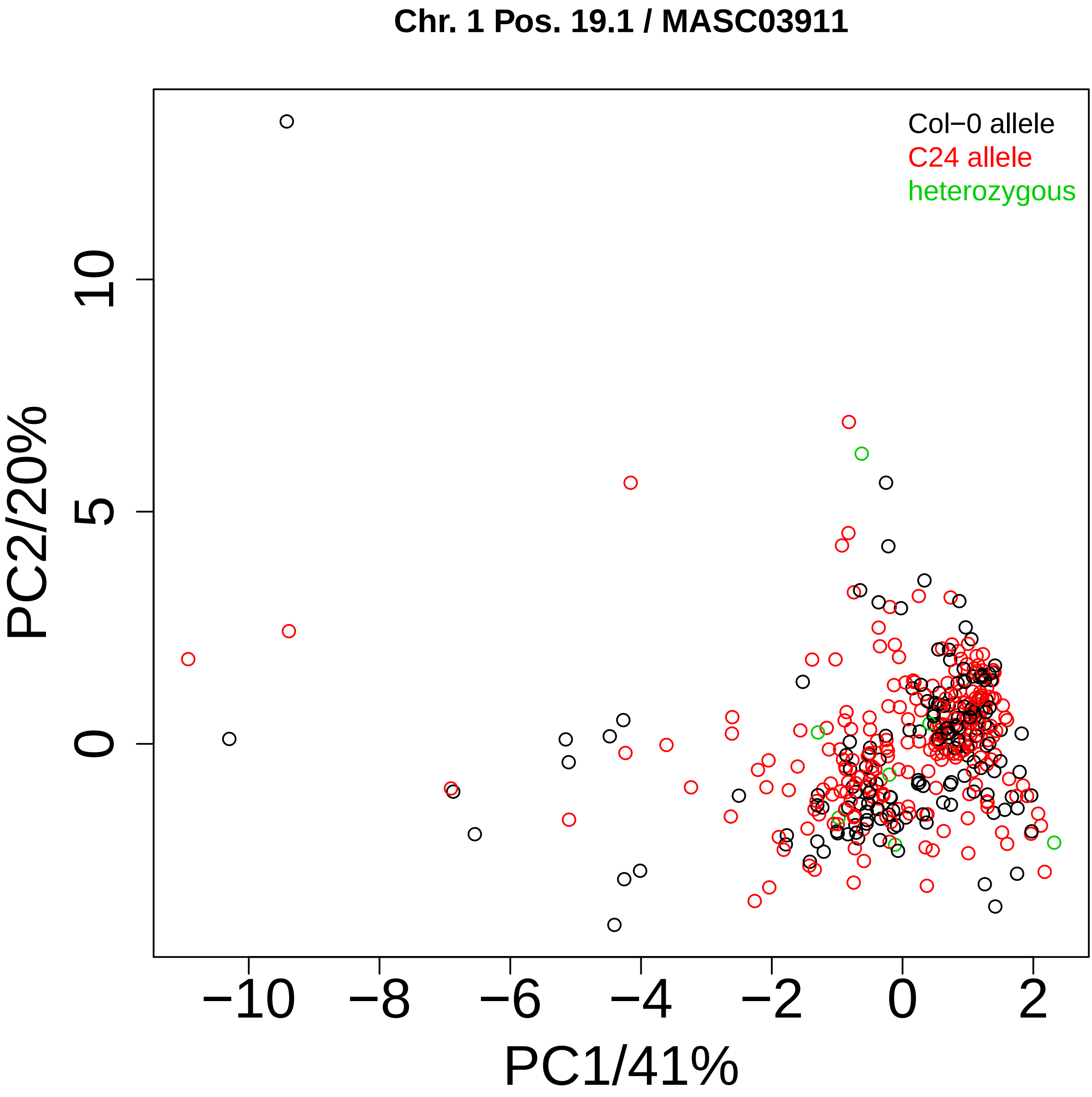
Chr. 1 Pos. 19.1 / MASC03911
Col−0 allele
C24 allele
heterozygous
PC2/20%
PC1/41%
10
5
0
−10
−8
−6
−4
−2
0
2

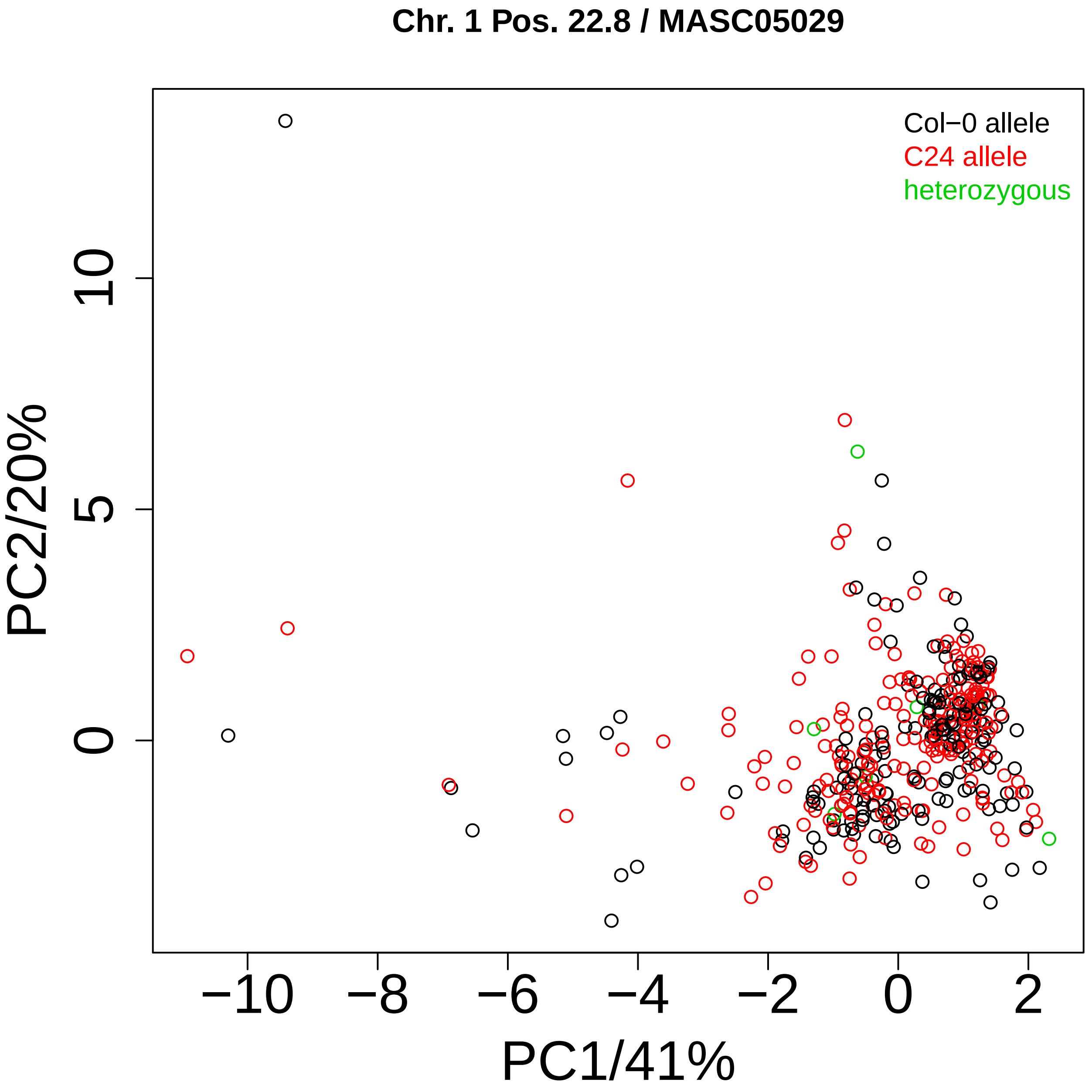
Chr. 1 Pos. 22.8 / MASC05029
Col−0 allele
C24 allele
heterozygous
PC2/20%
10
5
0
PC1/41%
−10
−8
−6
−4
−2
0
2

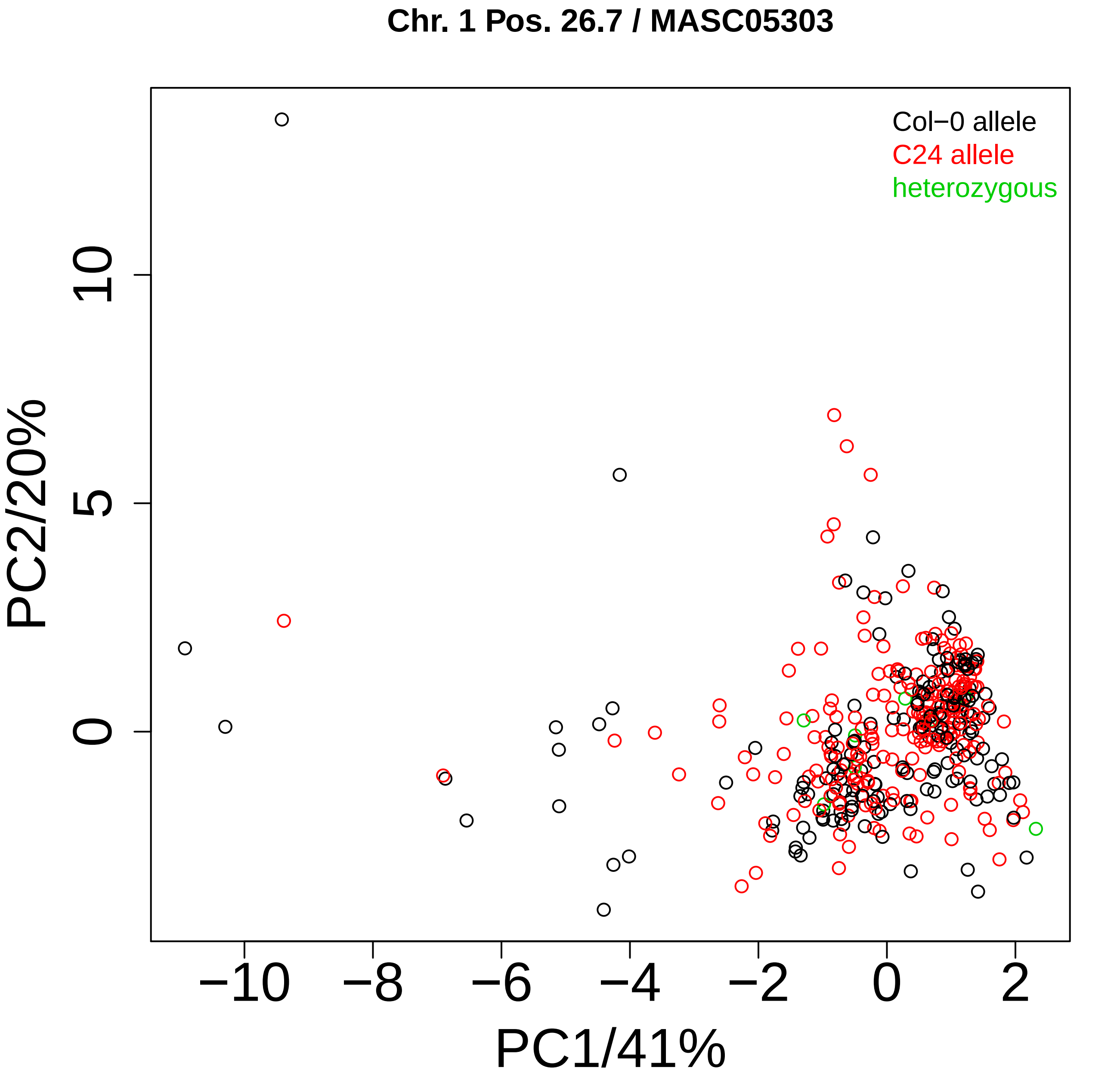

Chr. 1 Pos. 26.7 / MASC05303
Col−0 allele
C24 allele
heterozygous
10
5
0
PC2/20%
−10
−8
−6
−4
−2
0
2
PC1/41%

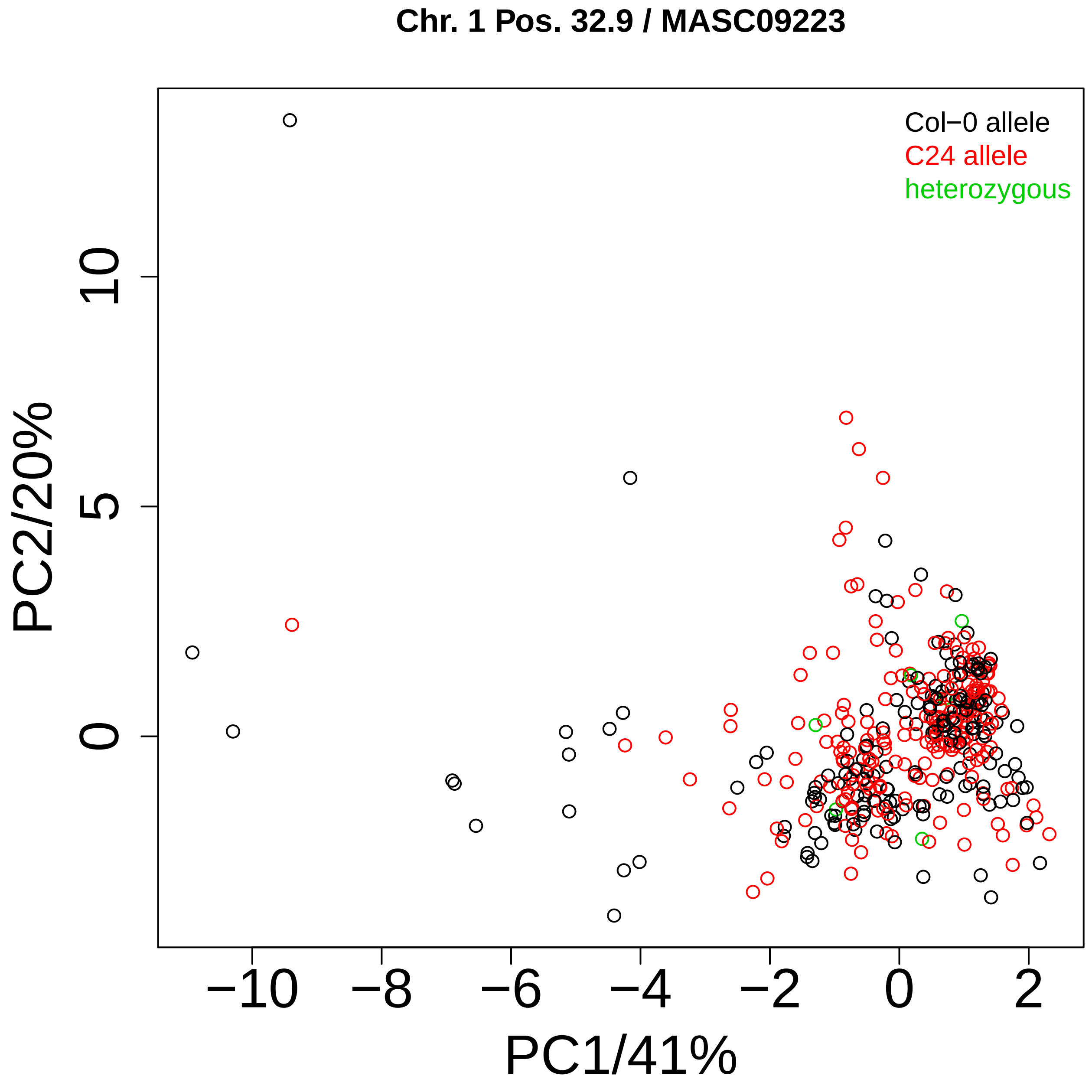
Chr. 1 Pos. 32.9 / MASC09223
Col−0 allele
C24 allele
heterozygous
PC2/20%
0
5
10
PC1/41%
−10
−8
−6
−4
−2
0
2

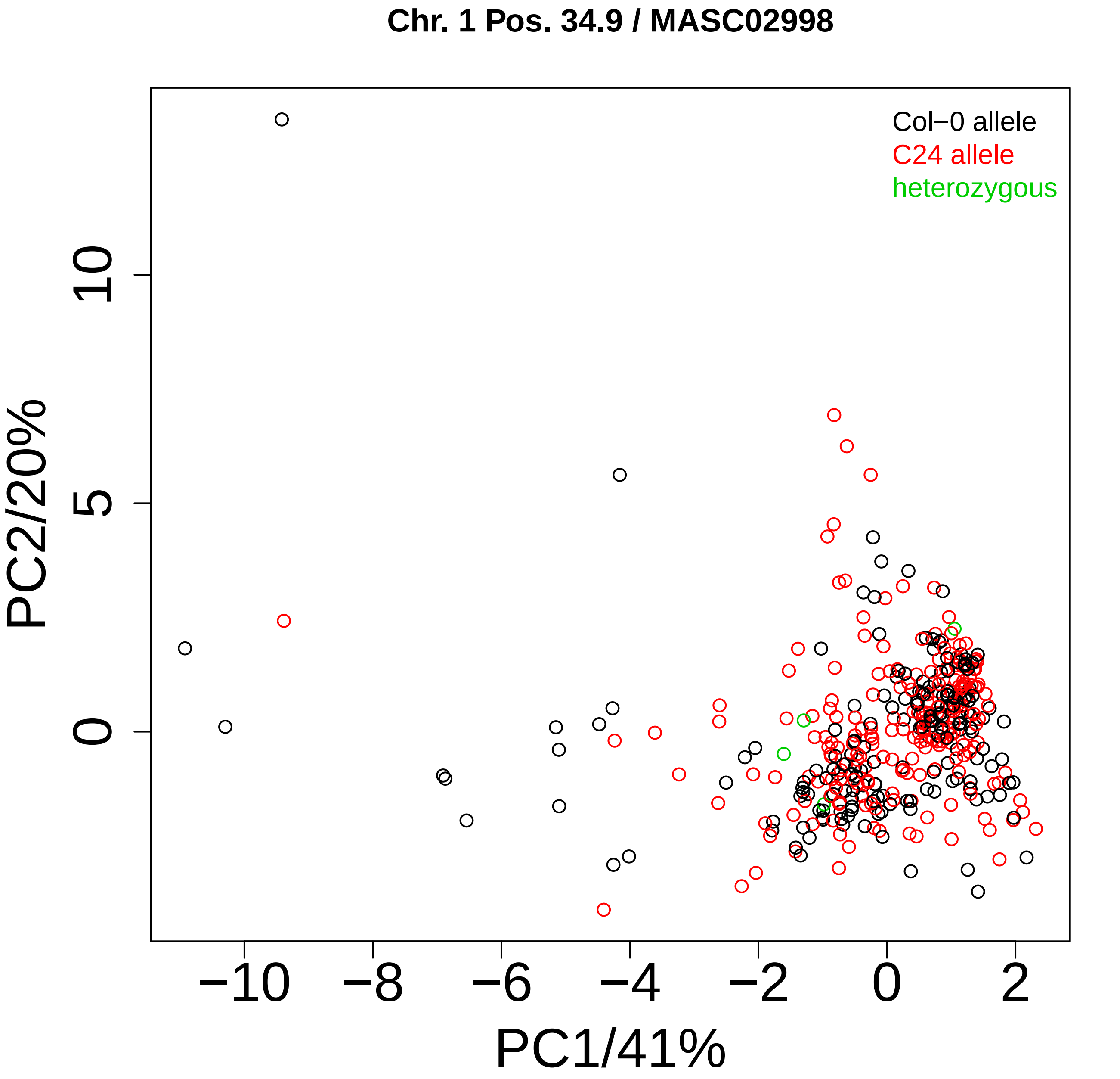

Chr. 1 Pos. 34.9 / MASC02998
Col−0 allele
C24 allele
heterozygous
PC2/20%
PC1/41%
10
5
0
−10
−8
−6
−4
−2
0
2

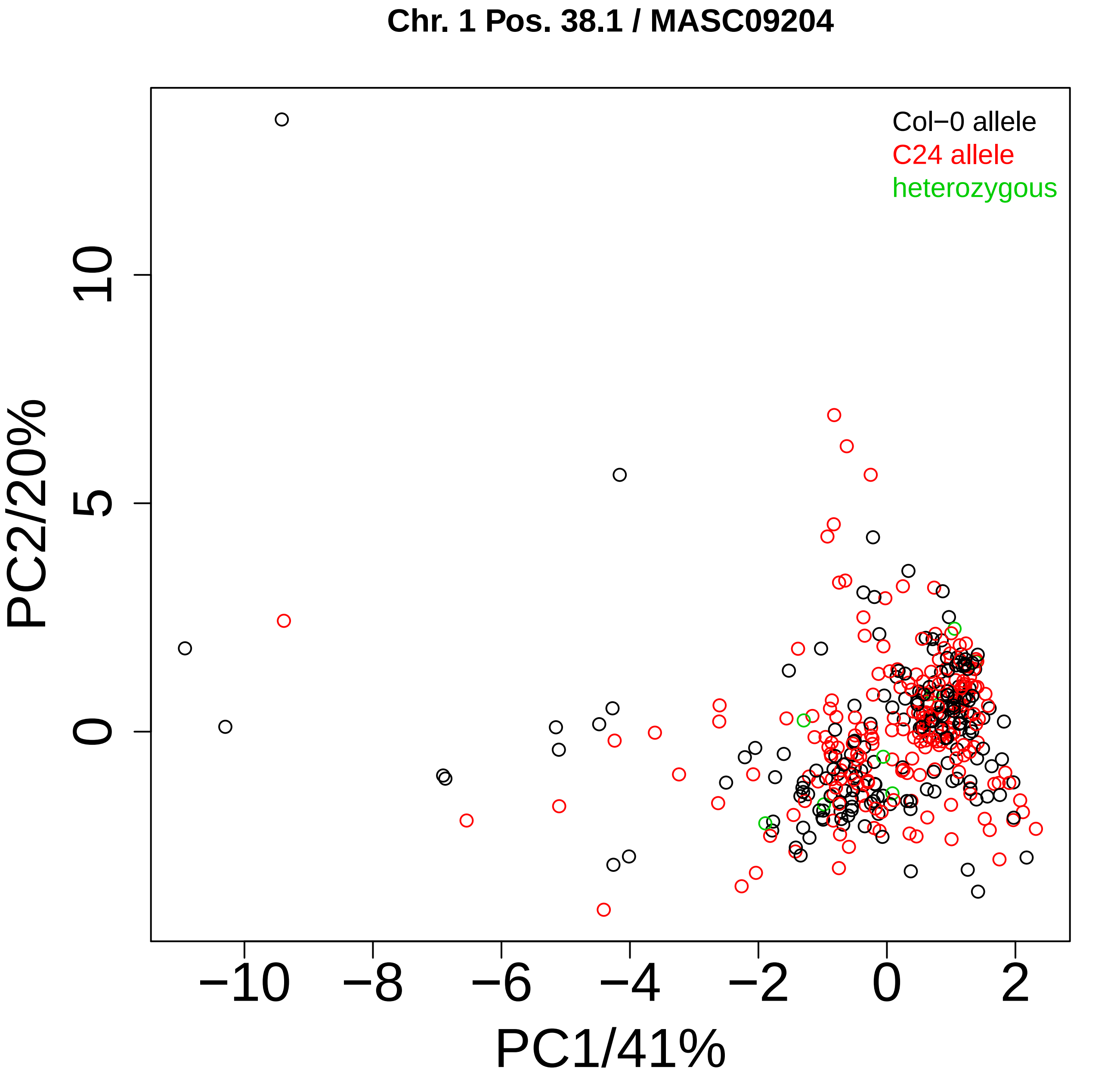

Chr. 1 Pos. 38.1 / MASC09204
Col−0 allele
C24 allele
heterozygous
PC2/20%
PC1/41%
10
5
0
−10
−8
−6
−4
−2
0
2

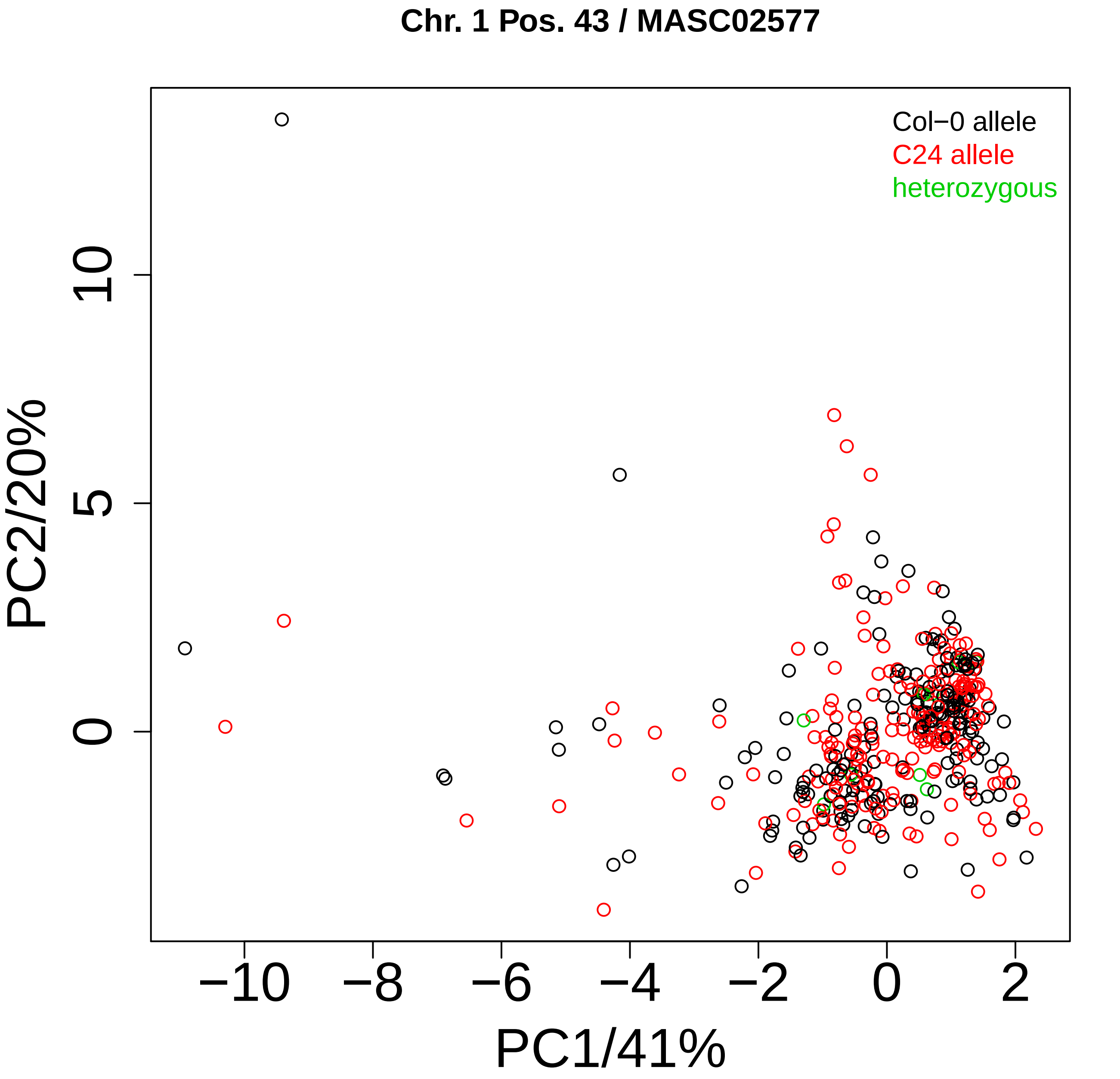
Chr. 1 Pos. 43 / MASC02577
Col−0 allele
C24 allele
heterozygous
PC2/20%
PC1/41%
0
5
10
−10
−8
−6
−4
−2
0
2

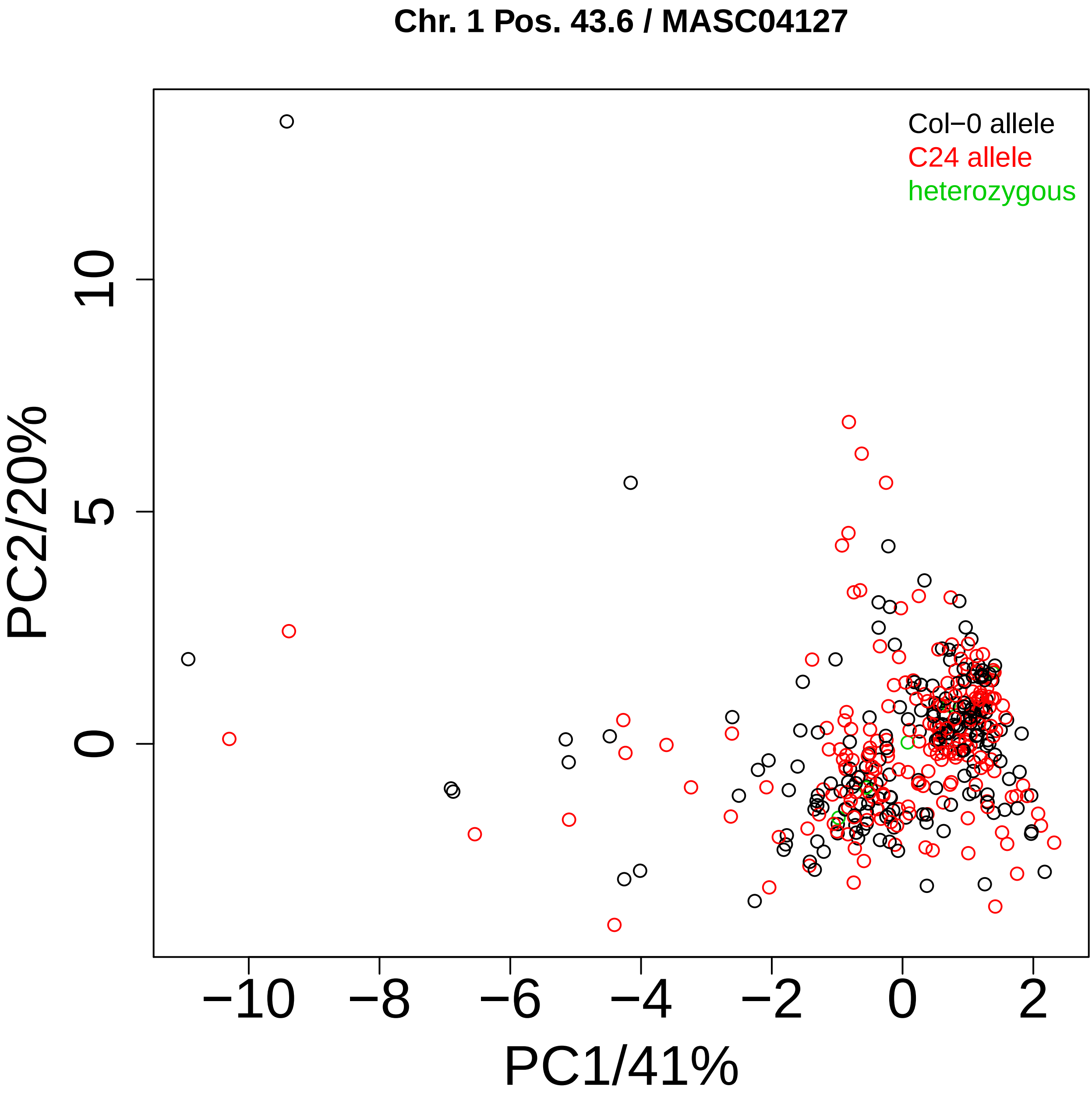
Chr. 1 Pos. 43.6 / MASC04127
Col−0 allele
C24 allele
heterozygous
PC2/20%
0
5
10
PC1/41%
−10
−8
−6
−4
−2
0
2

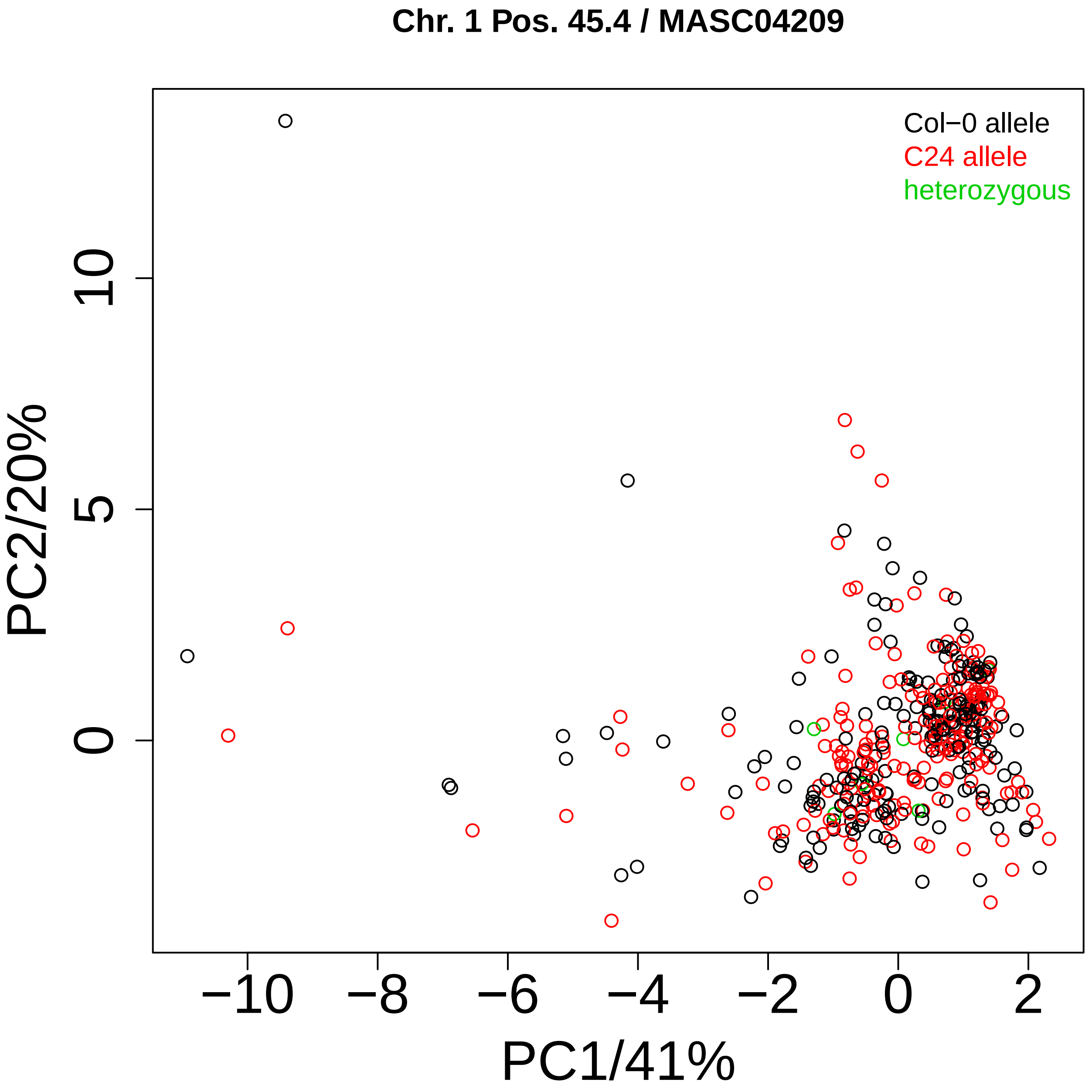
Chr. 1 Pos. 45.4 / MASC04209
Col−0 allele
C24 allele
heterozygous
PC2/20%
PC1/41%
0
5
10
−10
−8
−6
−4
−2
0
2

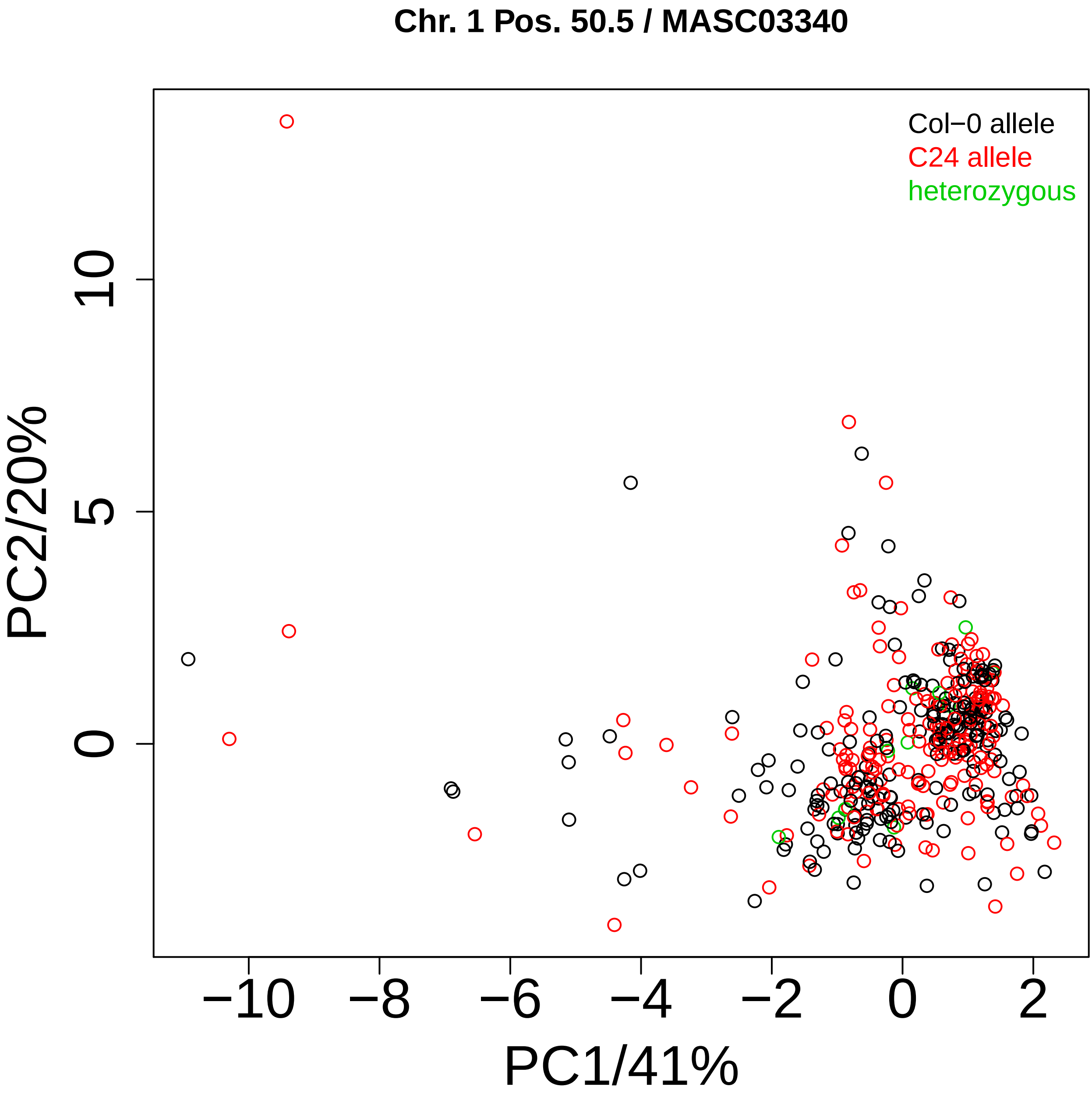
Chr. 1 Pos. 50.5 / MASC03340
Col−0 allele
C24 allele
heterozygous
PC2/20%
0
5
10
PC1/41%
−10
−8
−6
−4
−2
0
2

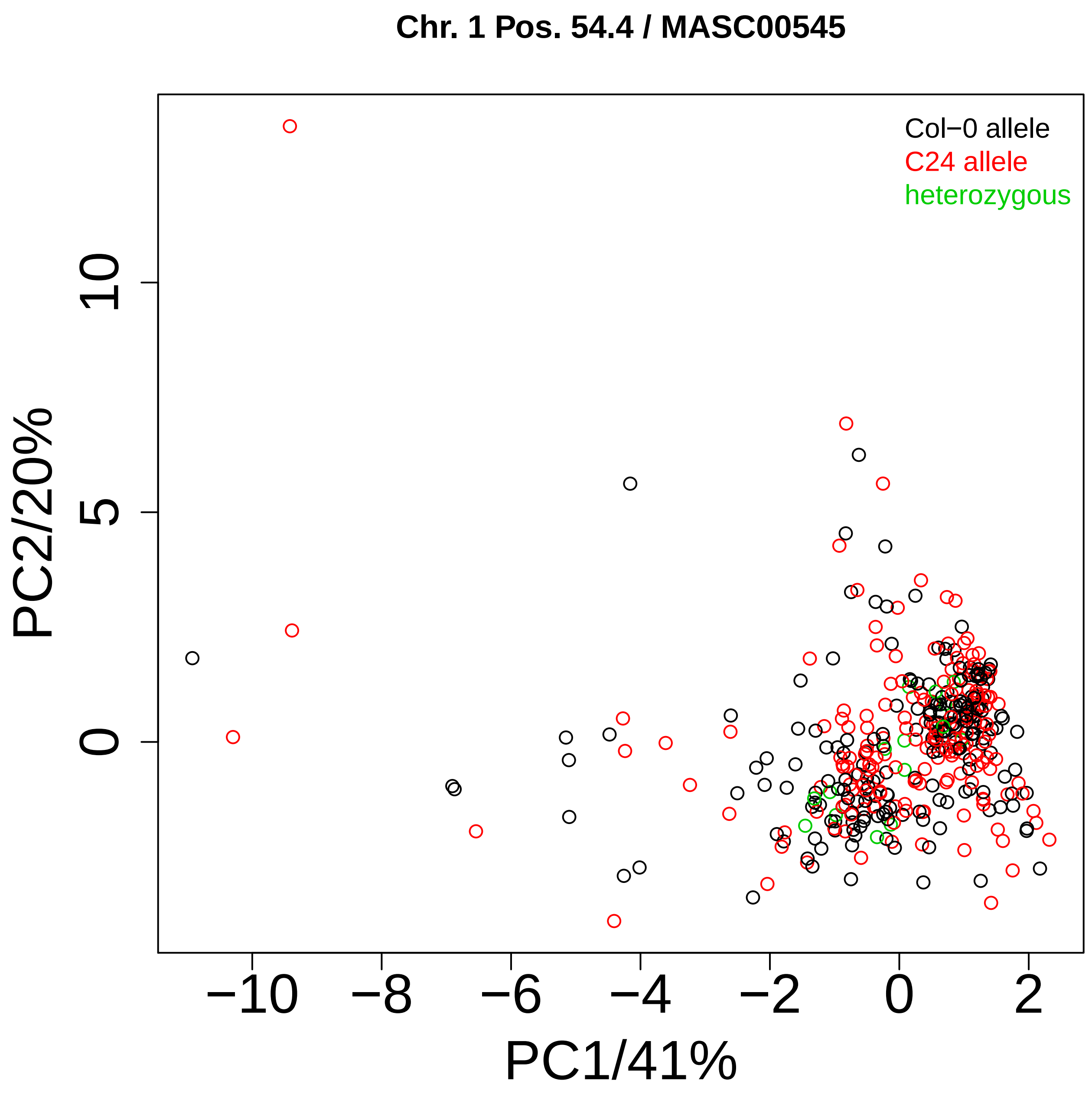

Chr. 1 Pos. 54.4 / MASC00545
Col−0 allele
C24 allele
heterozygous
10
5
0
PC2/20%
−10
−8
−6
−4
−2
0
2
PC1/41%

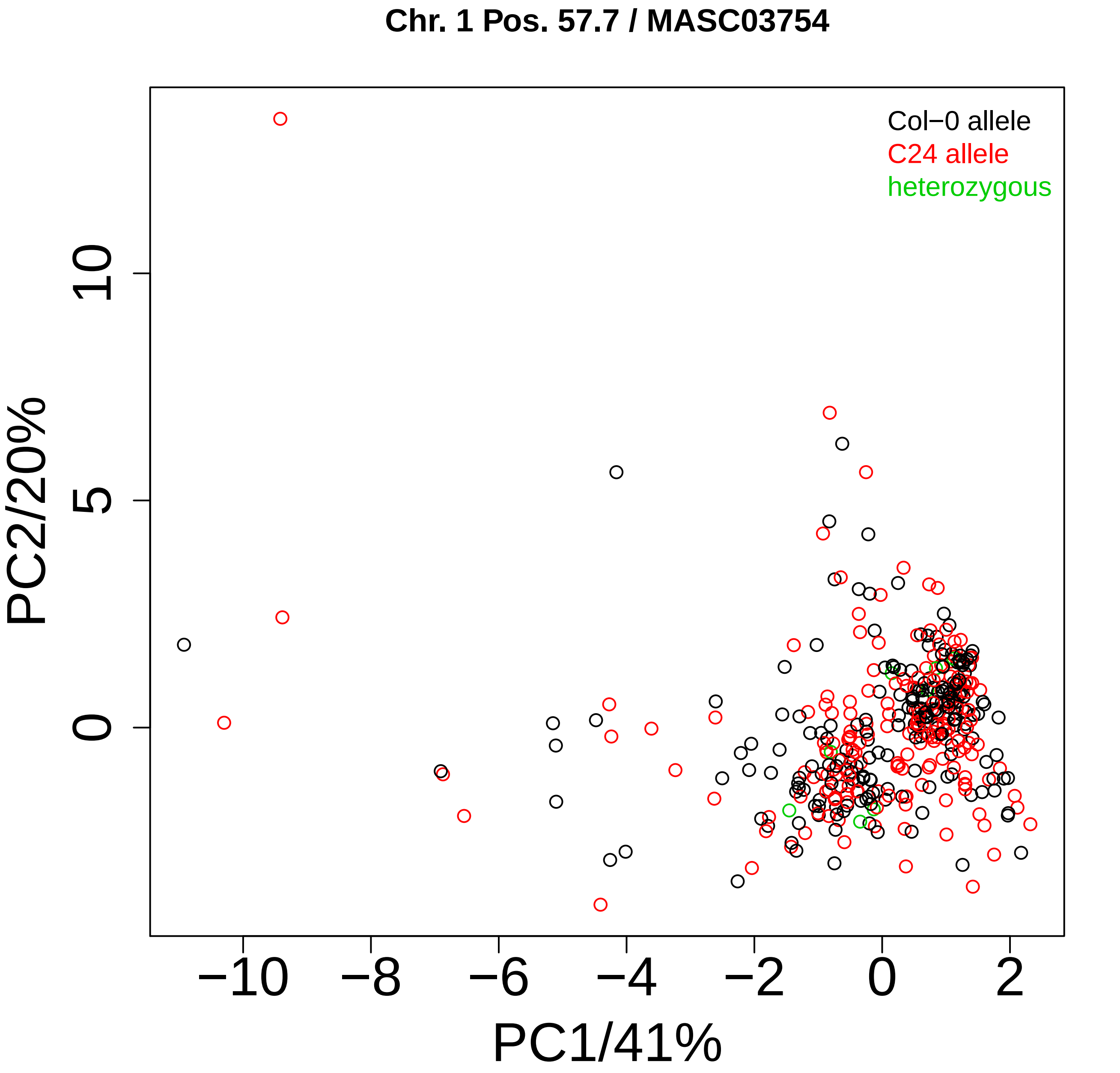
Chr. 1 Pos. 57.7 / MASC03754
Col−0 allele
C24 allele
heterozygous
PC2/20%
0
5
10
PC1/41%
−10
−8
−6
−4
−2
0
2

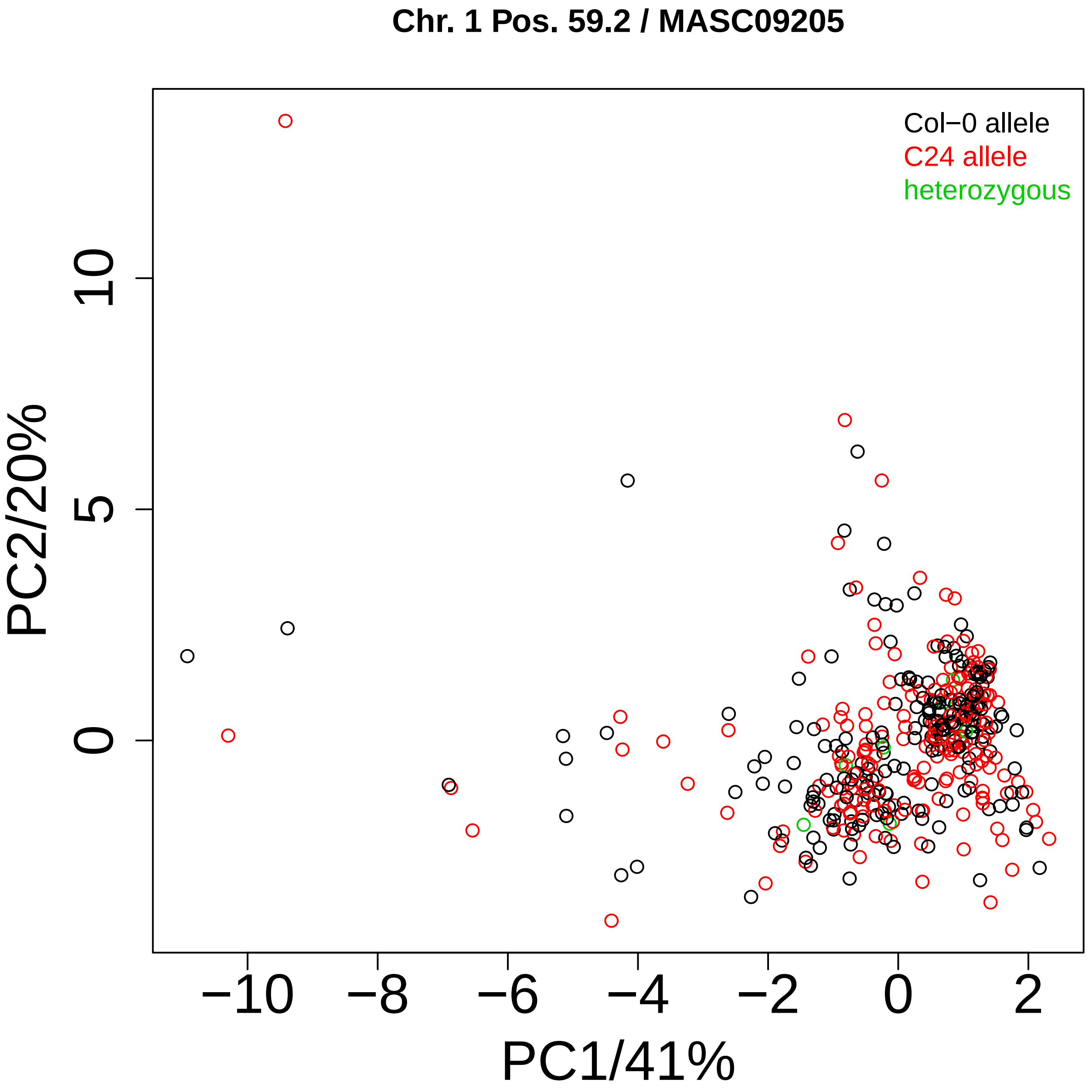
Chr. 1 Pos. 59.2 / MASC09205
Col−0 allele
C24 allele
heterozygous
PC2/20%
10
5
0
PC1/41%
−10
−8
−6
−4
−2
0
2

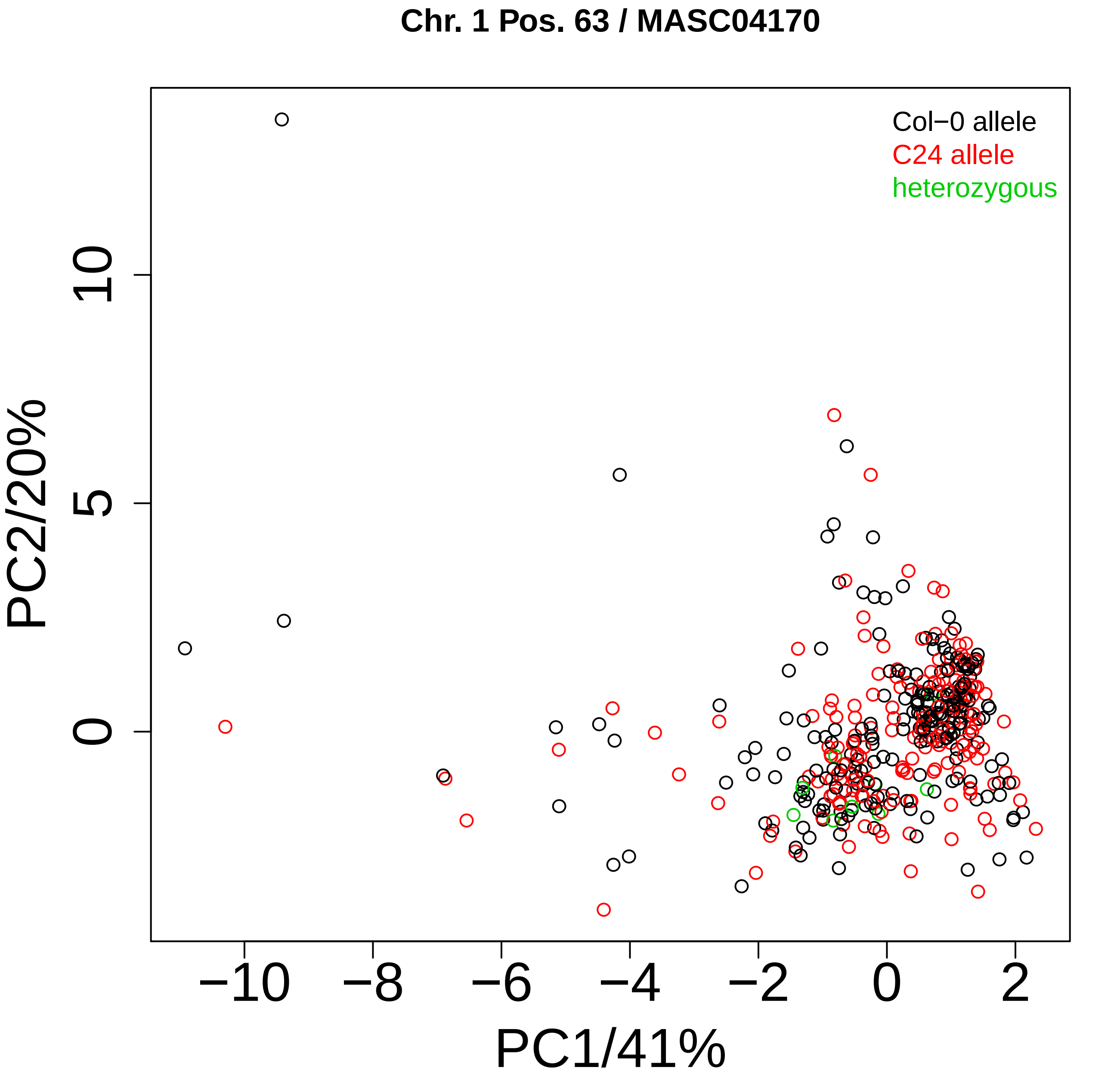
Chr. 1 Pos. 63 / MASC04170
Col−0 allele
C24 allele
heterozygous
PC2/20%
PC1/41%
0
5
10
−10
−8
−6
−4
−2
0
2

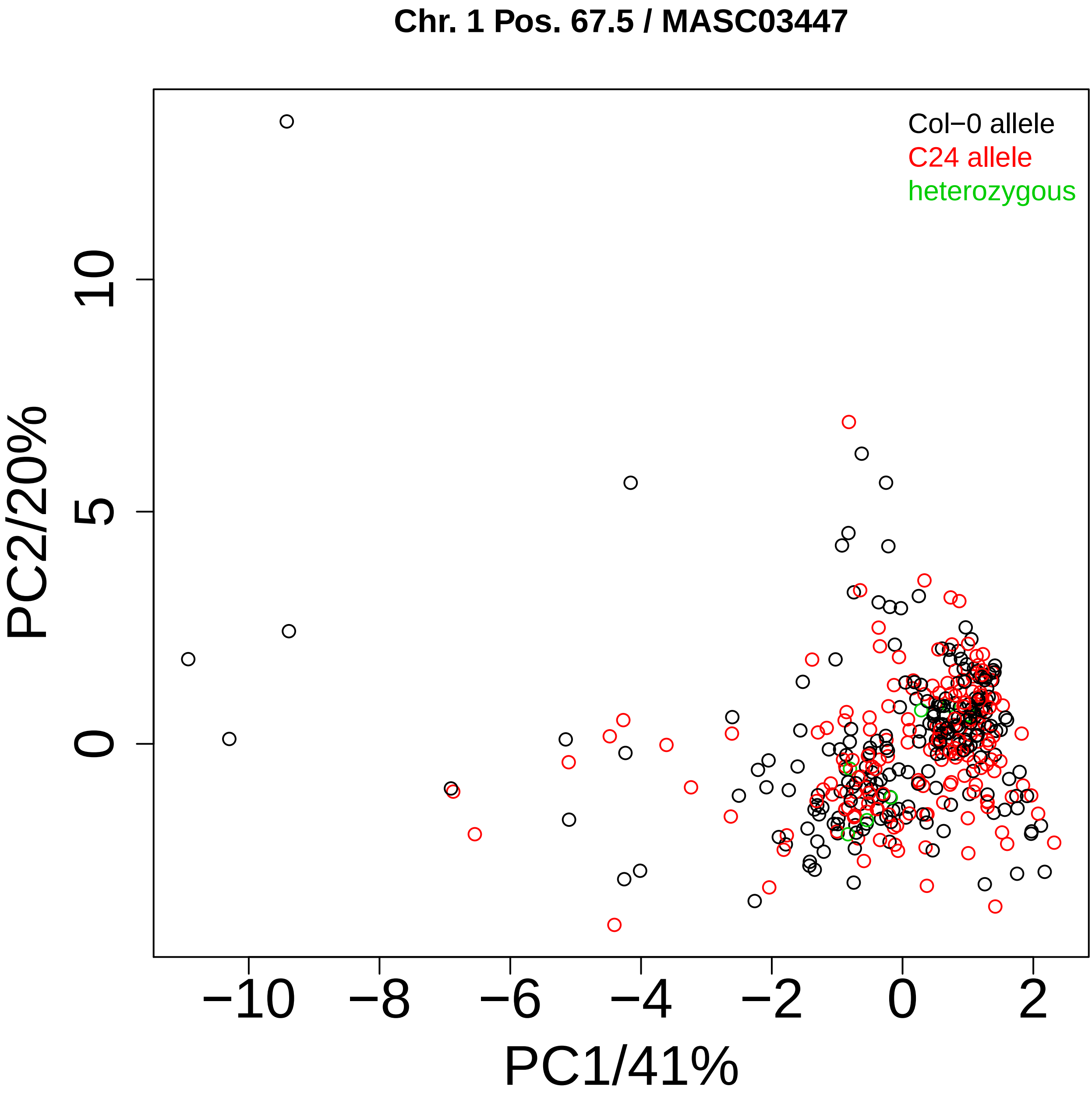
Chr. 1 Pos. 67.5 / MASC03447
Col−0 allele
C24 allele
heterozygous
PC2/20%
0
5
10
PC1/41%
−10
−8
−6
−4
−2
0
2

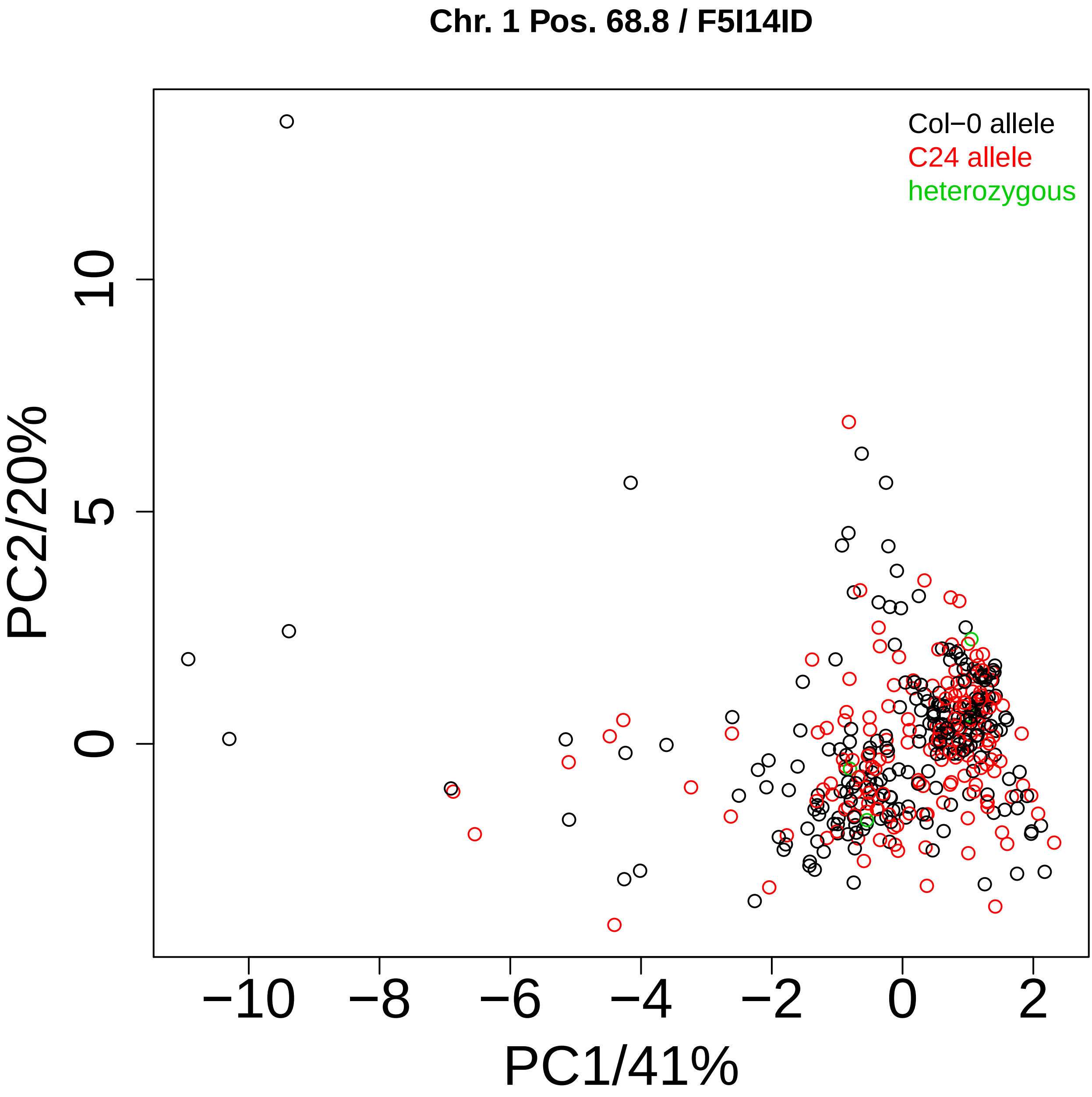

Chr. 1 Pos. 68.8 / F5I14ID
Col−0 allele
C24 allele
heterozygous
PC2/20%
0
5
10
PC1/41%
−10
−8
−6
−4
−2
0
2

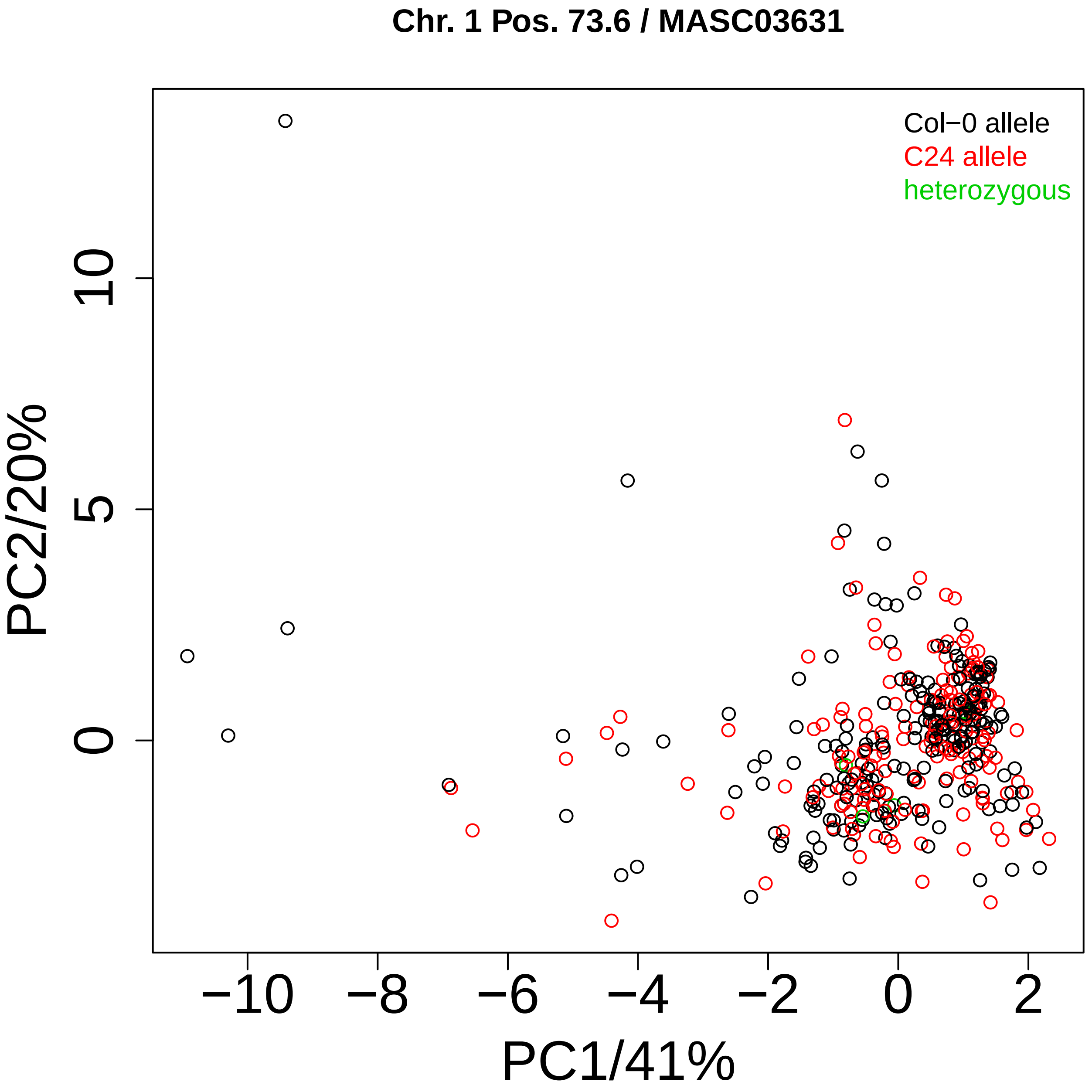
Chr. 1 Pos. 73.6 / MASC03631
Col−0 allele
C24 allele
heterozygous
PC2/20%
0
5
10
PC1/41%
−10
−8
−6
−4
−2
0
2

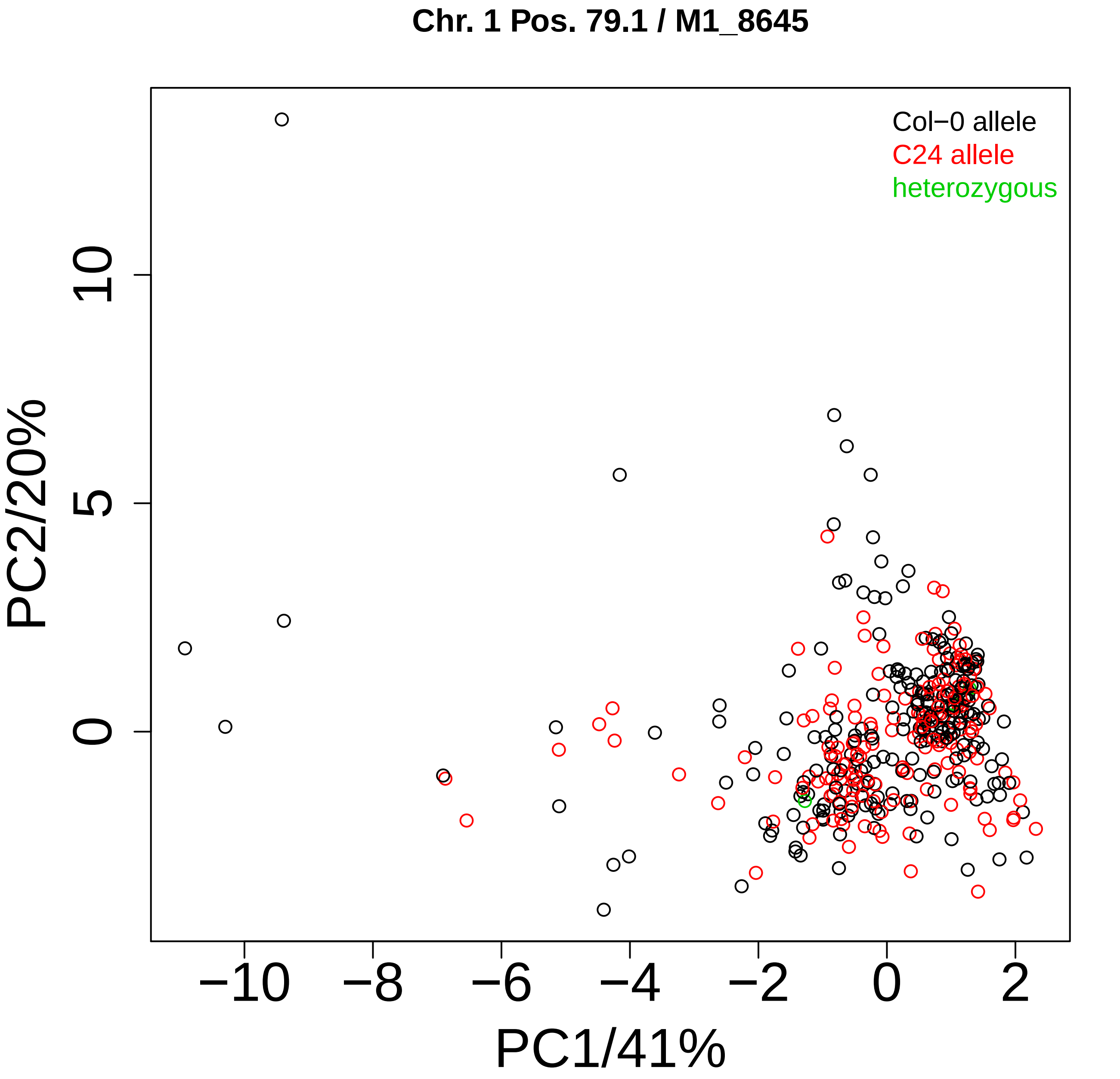
Chr. 1 Pos. 79.1 / M1_8645
Col−0 allele
C24 allele
heterozygous
PC2/20%
0
5
10
PC1/41%
−10
−8
−6
−4
−2
0
2

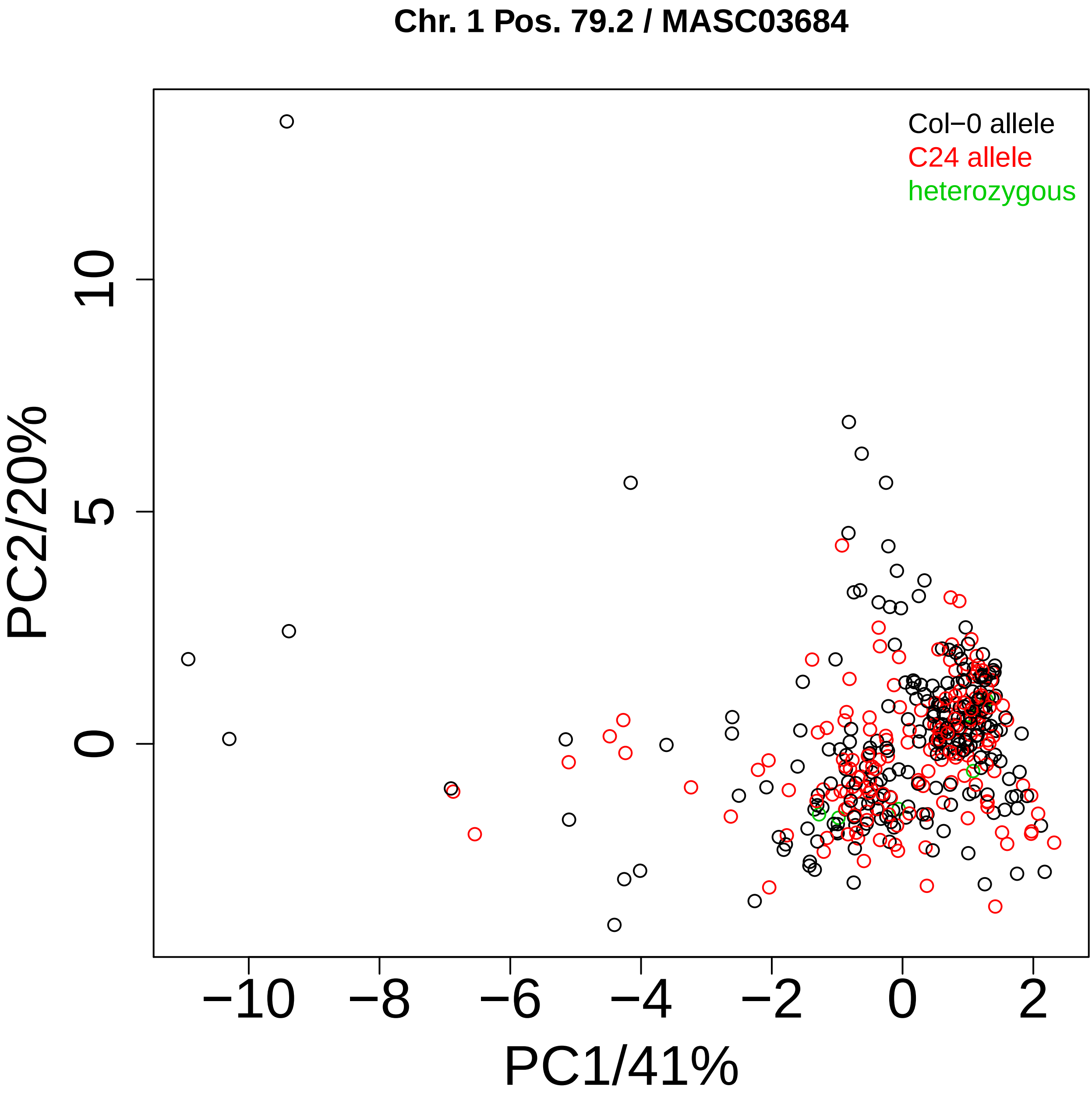

Chr. 1 Pos. 79.2 / MASC03684
Col−0 allele
C24 allele
heterozygous
PC2/20%
PC1/41%
10
5
0
−10
−8
−6
−4
−2
0
2

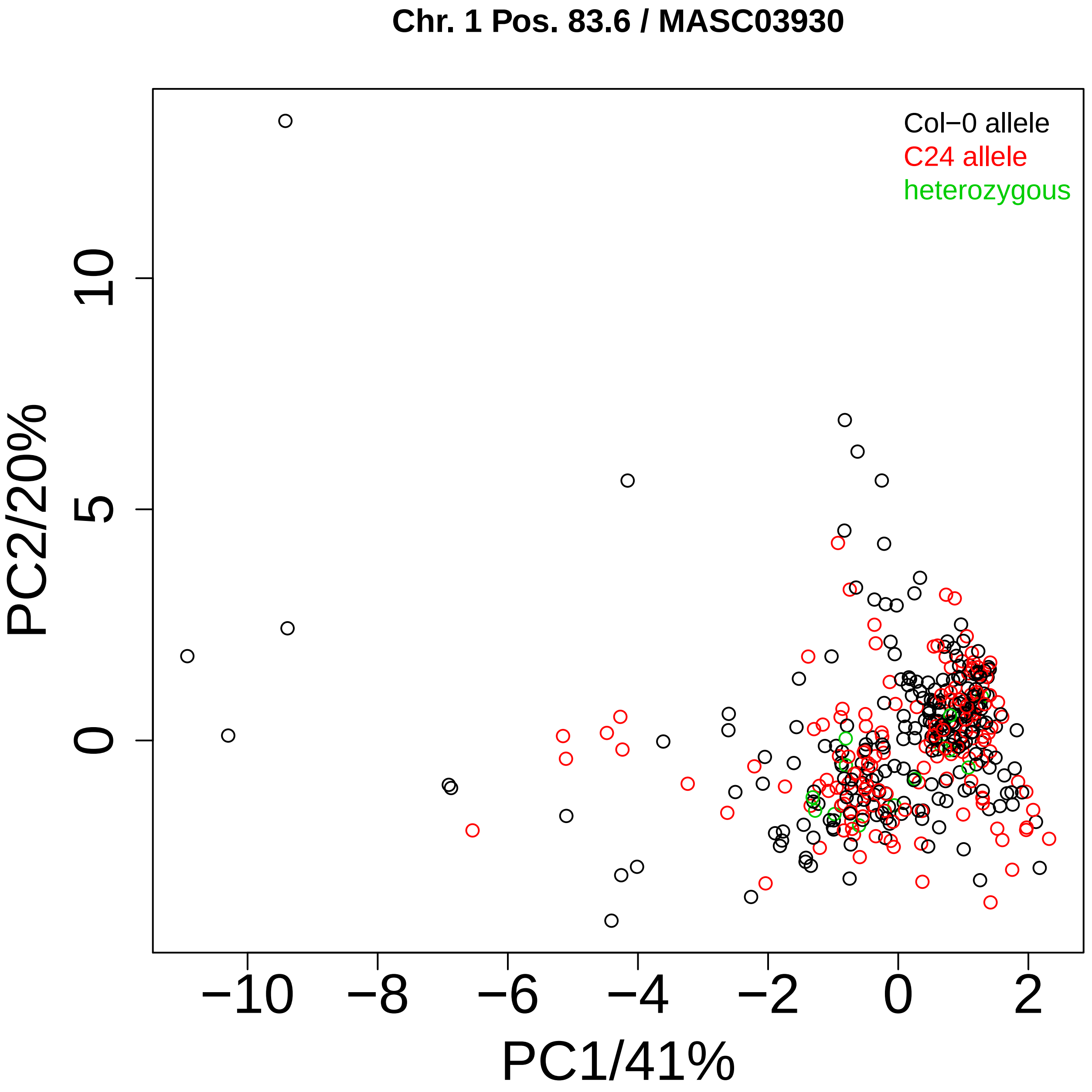
Chr. 1 Pos. 83.6 / MASC03930
Col−0 allele
C24 allele
heterozygous
PC2/20%
0
5
10
PC1/41%
−10
−8
−6
−4
−2
0
2

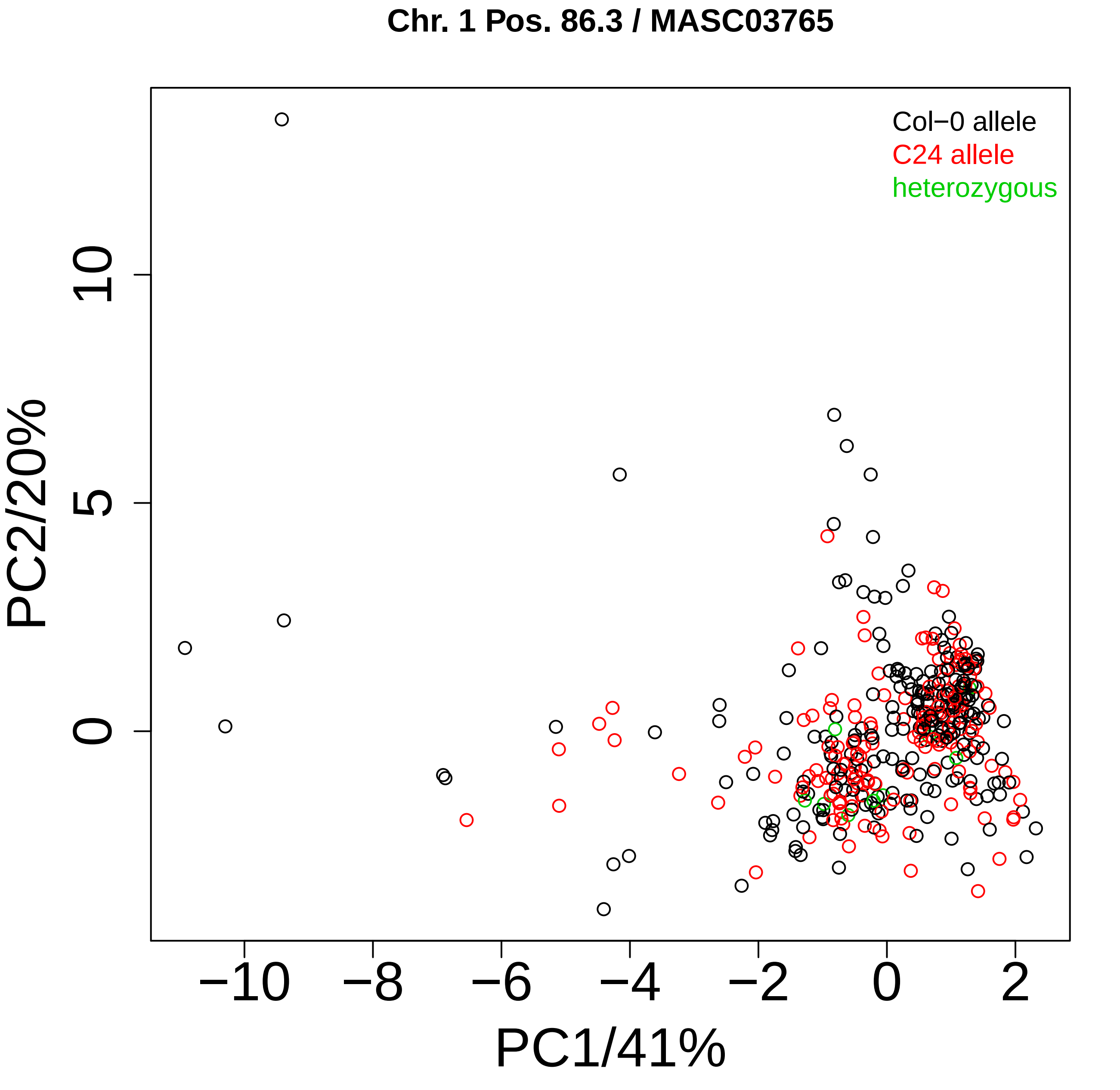
Chr. 1 Pos. 86.3 / MASC03765
Col−0 allele
C24 allele
heterozygous
PC2/20%
0
5
10
PC1/41%
−10
−8
−6
−4
−2
0
2

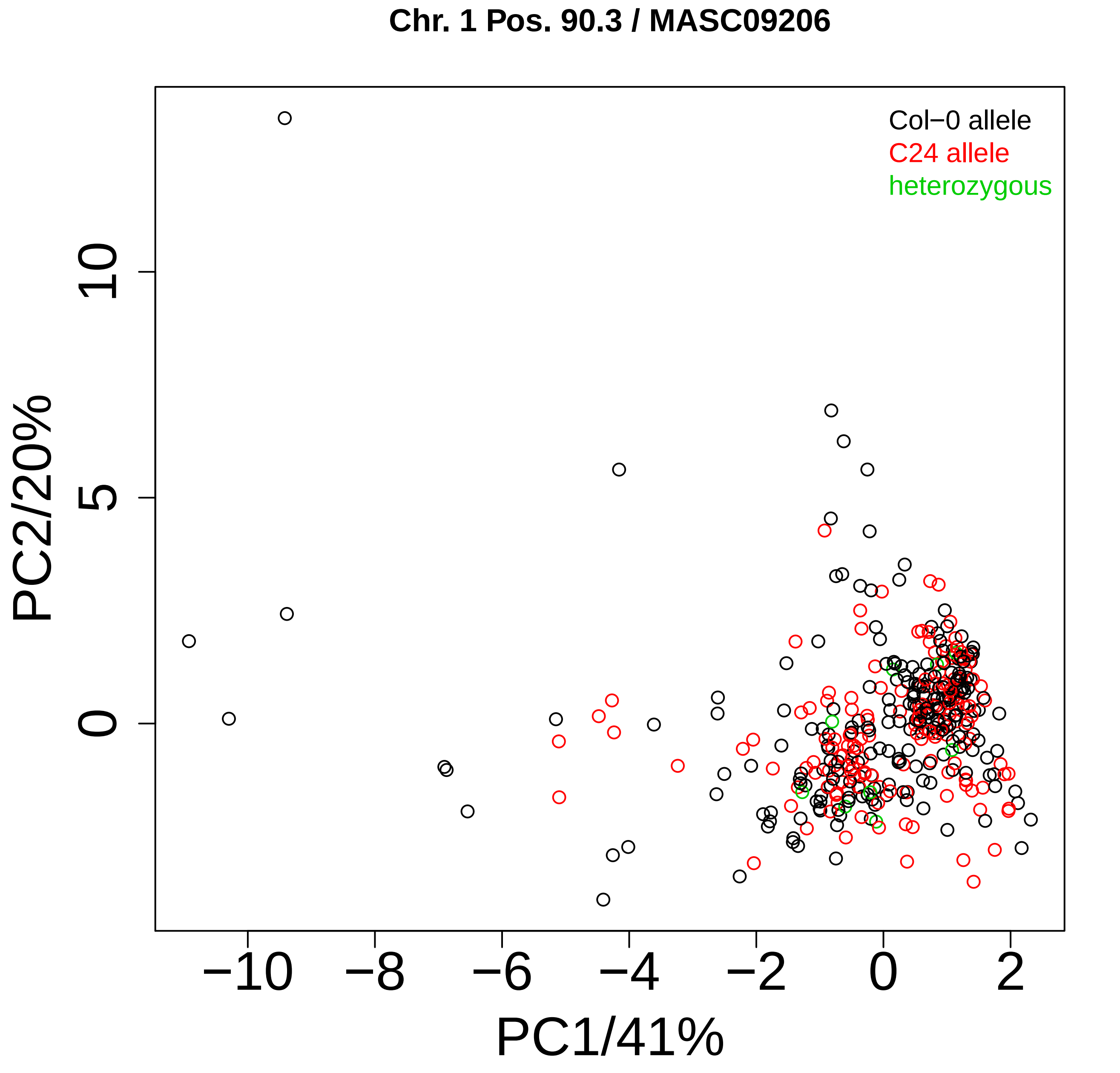
Chr. 1 Pos. 90.3 / MASC09206
Col−0 allele
C24 allele
heterozygous
PC2/20%
0
5
10
PC1/41%
−10
−8
−6
−4
−2
0
2

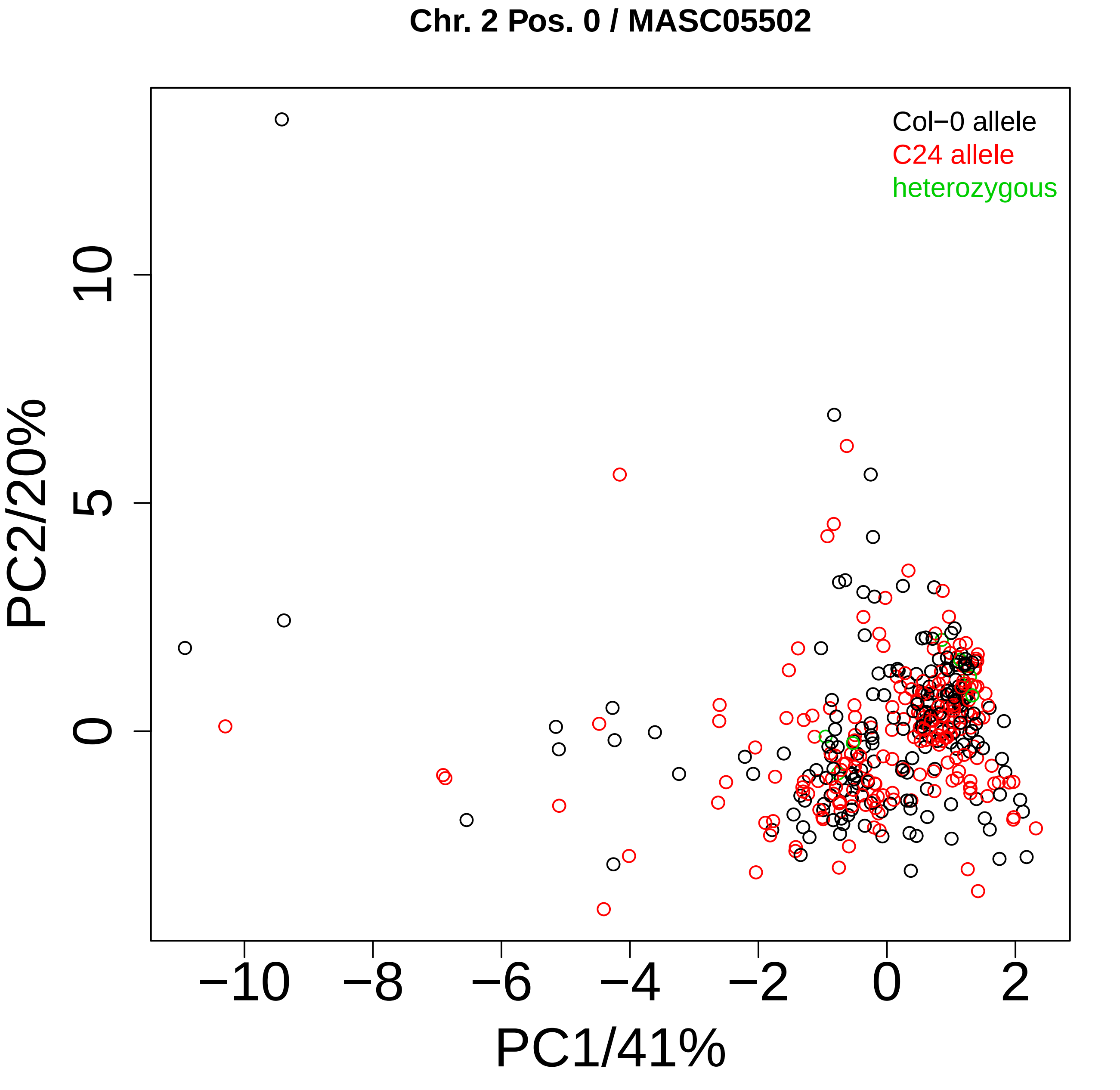
Chr. 2 Pos. 0 / MASC05502
Col−0 allele
C24 allele
heterozygous
PC2/20%
10
5
0
−10
−8
−6
−4
−2
0
2
PC1/41%

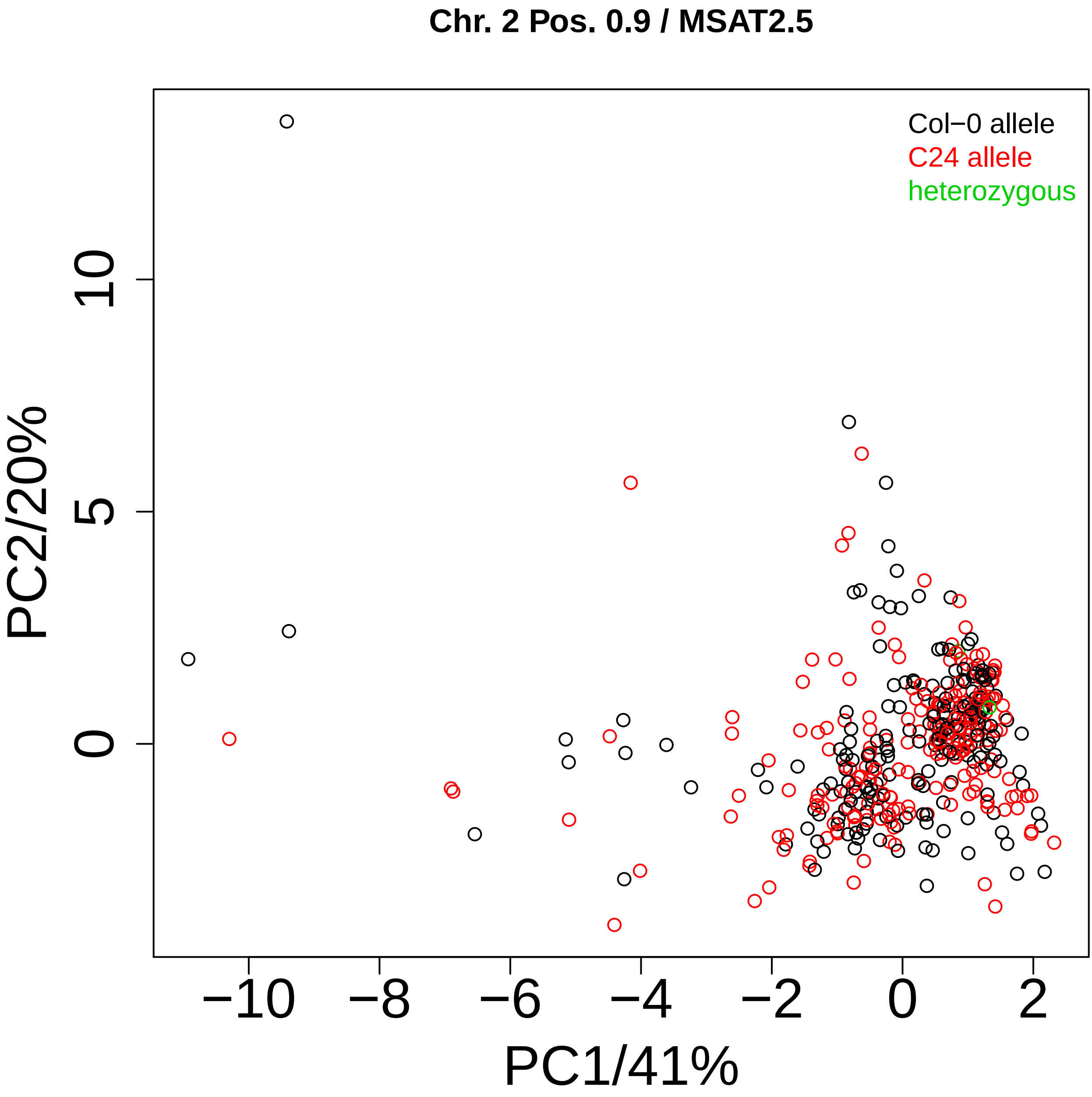
Chr. 2 Pos. 0.9 / MSAT2.5
Col−0 allele
C24 allele
heterozygous
PC1/41%
PC2/20%
−10
−8
−6
−4
−2
0
2
0
5
10

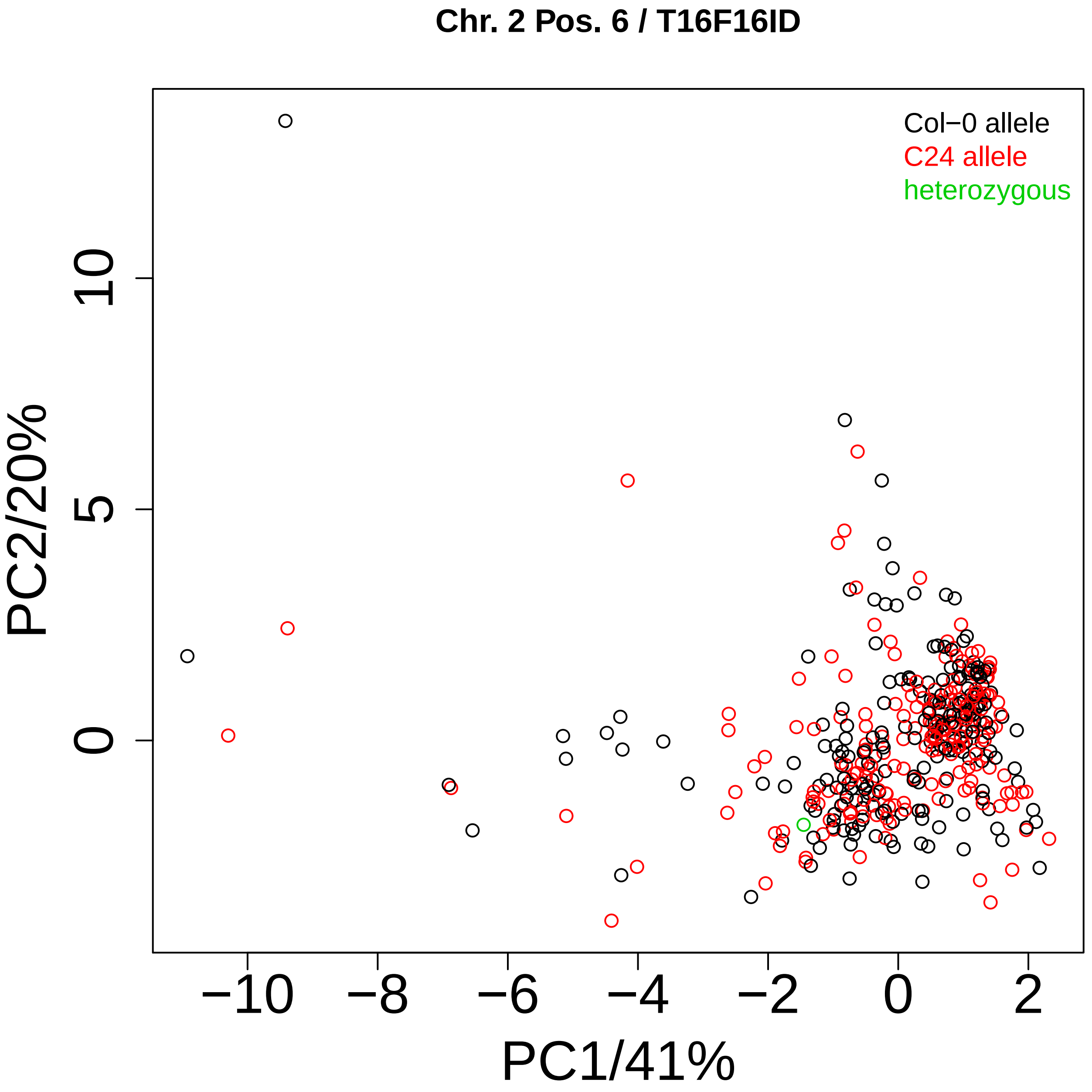

Chr. 2 Pos. 6 / T16F16ID
Col−0 allele
C24 allele
heterozygous
PC1/41%
PC2/20%
−10
−8
−6
−4
−2
0
2
0
5
10

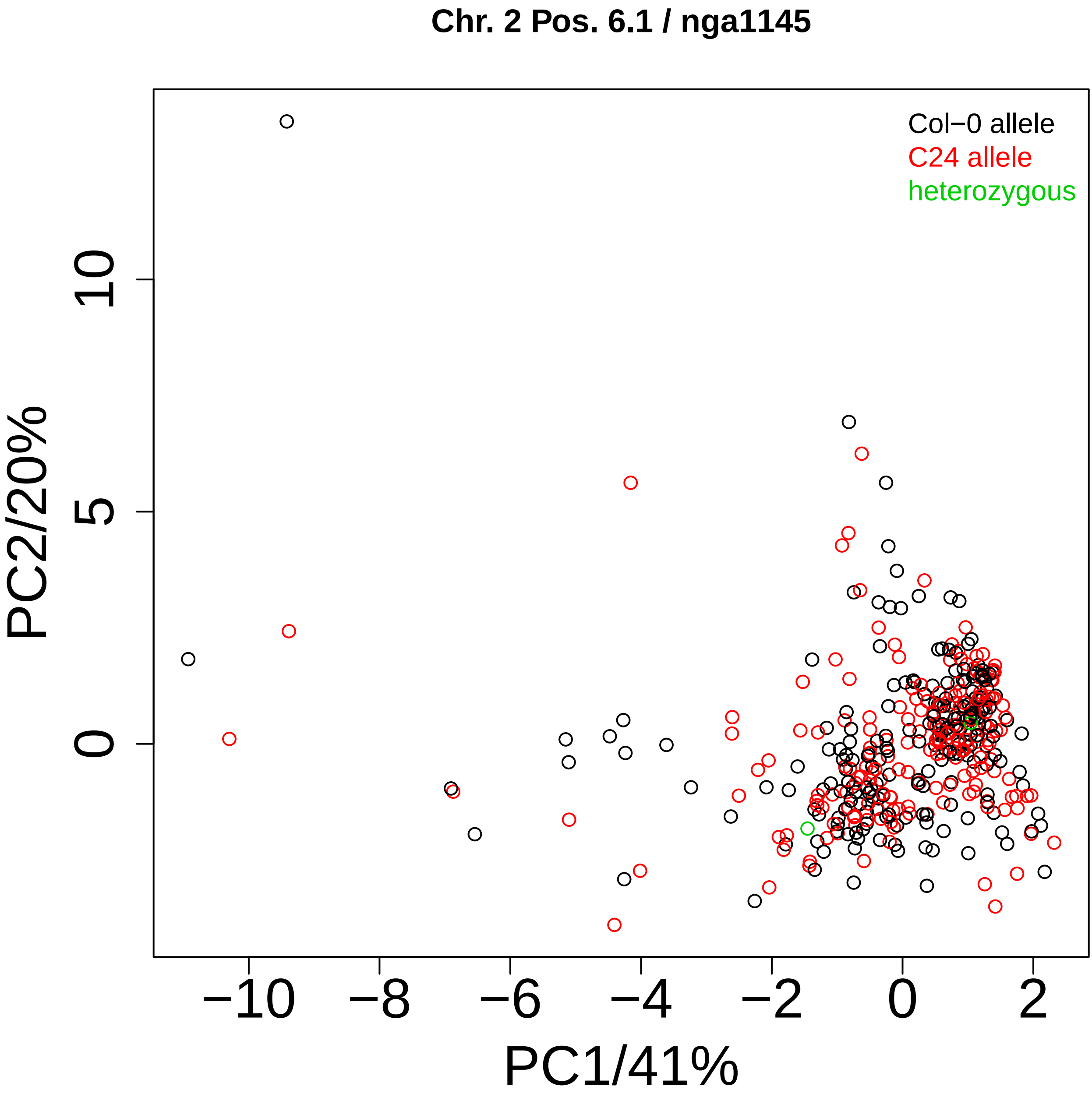

Chr. 2 Pos. 6.1 / nga1145
Col−0 allele
C24 allele
heterozygous
PC1/41%
PC2/20%
−10
−8
−6
−4
−2
0
2
0
5
10

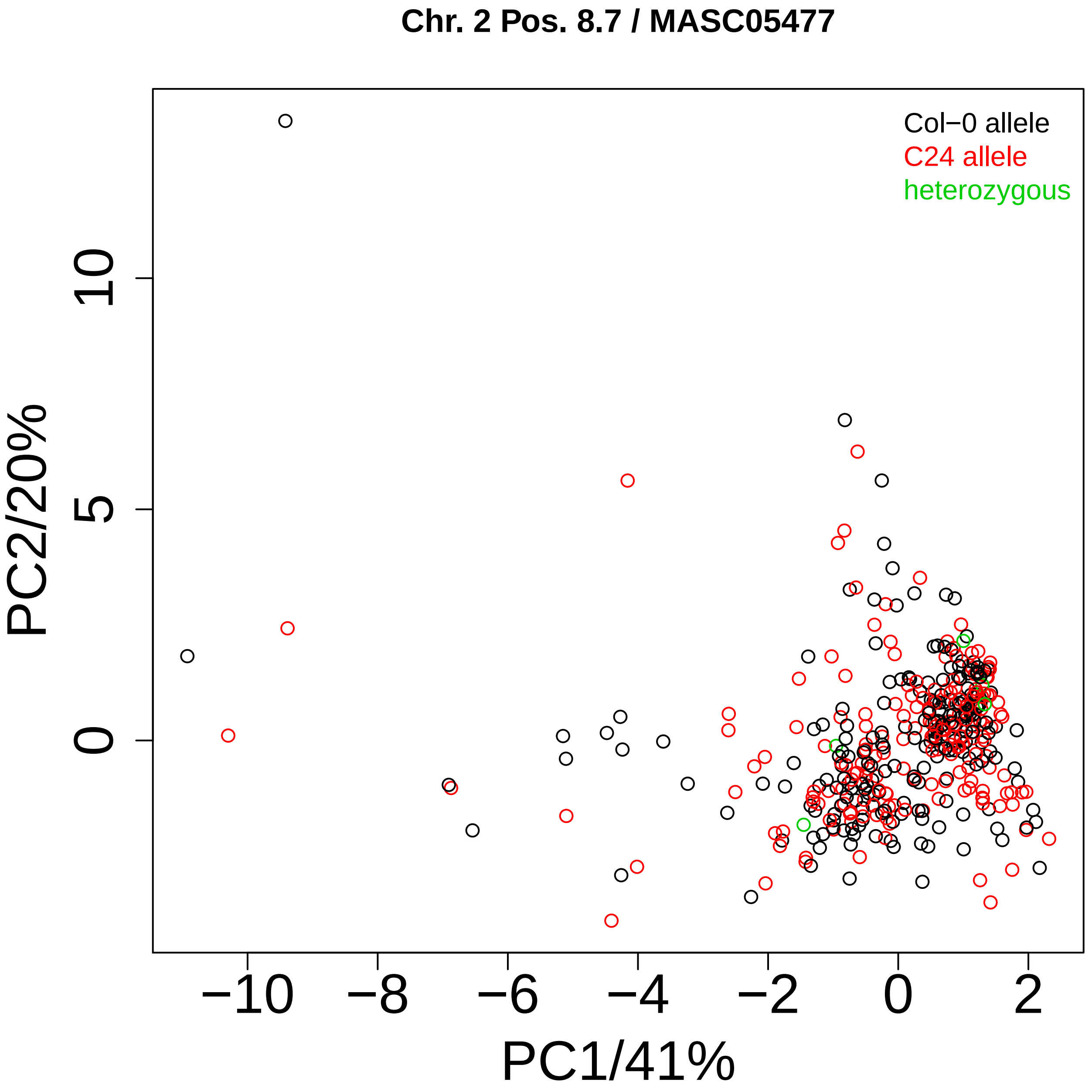

Chr. 2 Pos. 8.7 / MASC05477
Col−0 allele
C24 allele
heterozygous
PC2/20%
10
5
0
−10
−8
−6
−4
−2
0
2
PC1/41%

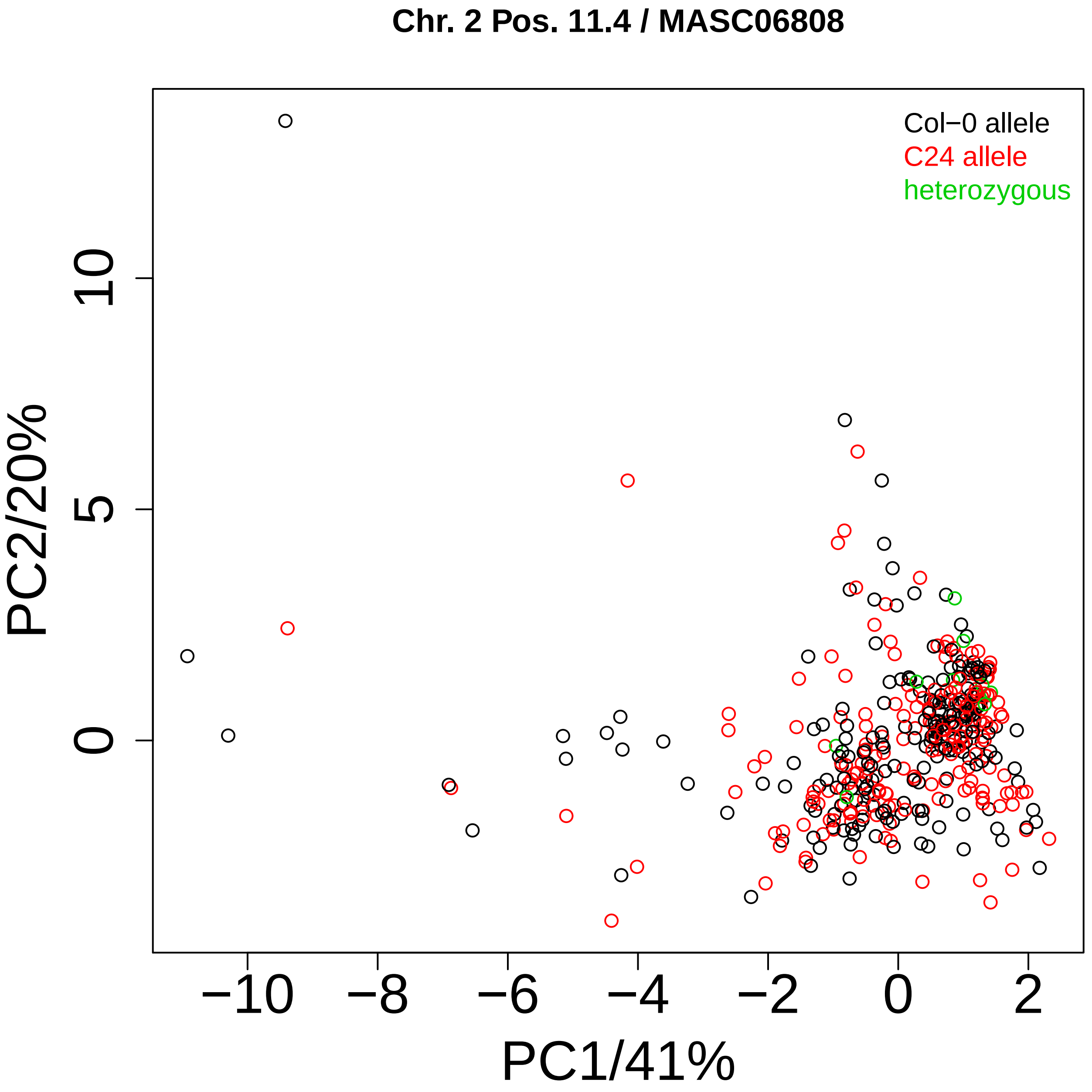
Chr. 2 Pos. 11.4 / MASC06808
Col−0 allele
C24 allele
heterozygous
PC2/20%
PC1/41%
0
5
10
−10
−8
−6
−4
−2
0
2

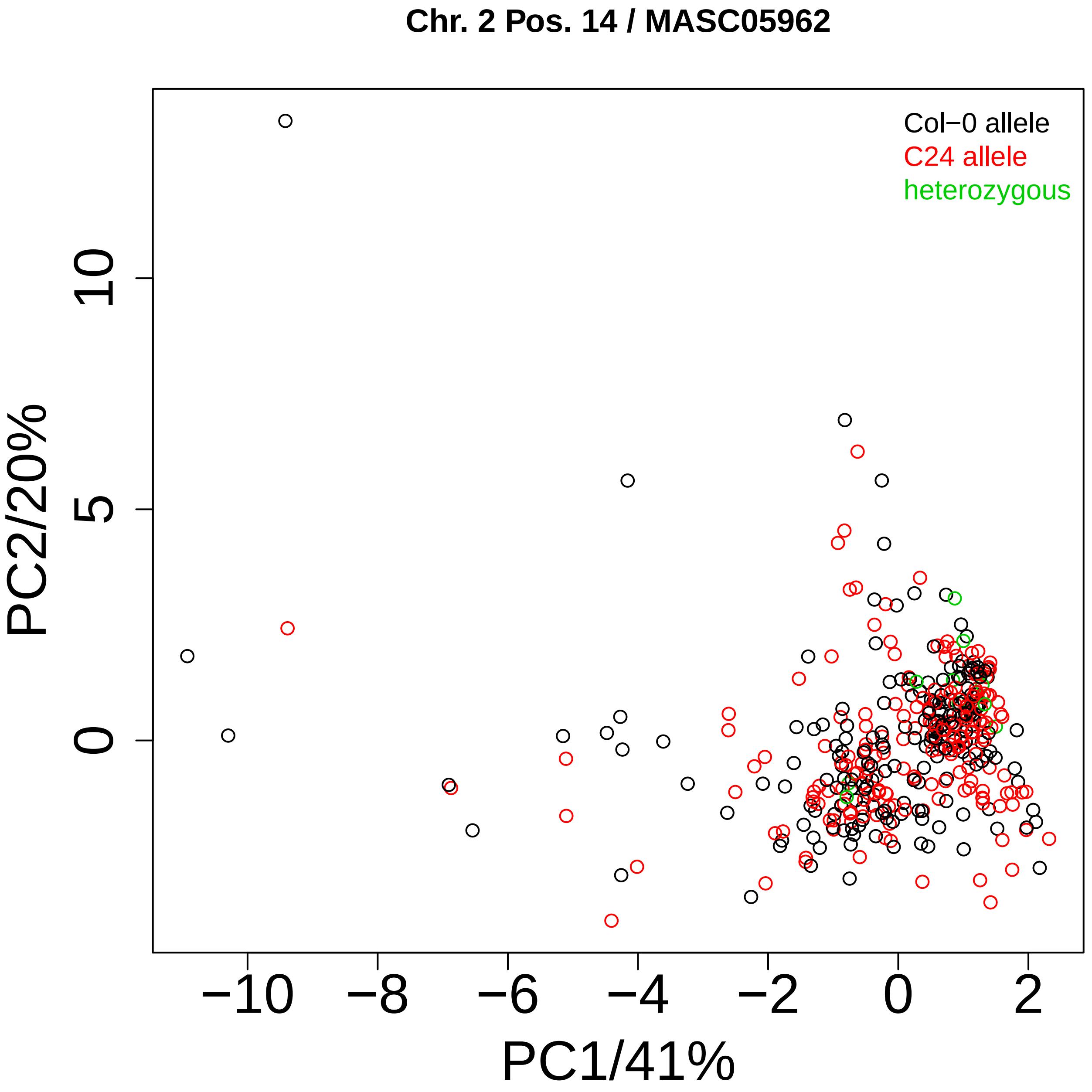
Chr. 2 Pos. 14 / MASC05962
Col−0 allele
C24 allele
heterozygous
PC2/20%
0
5
10
PC1/41%
−10
−8
−6
−4
−2
0
2

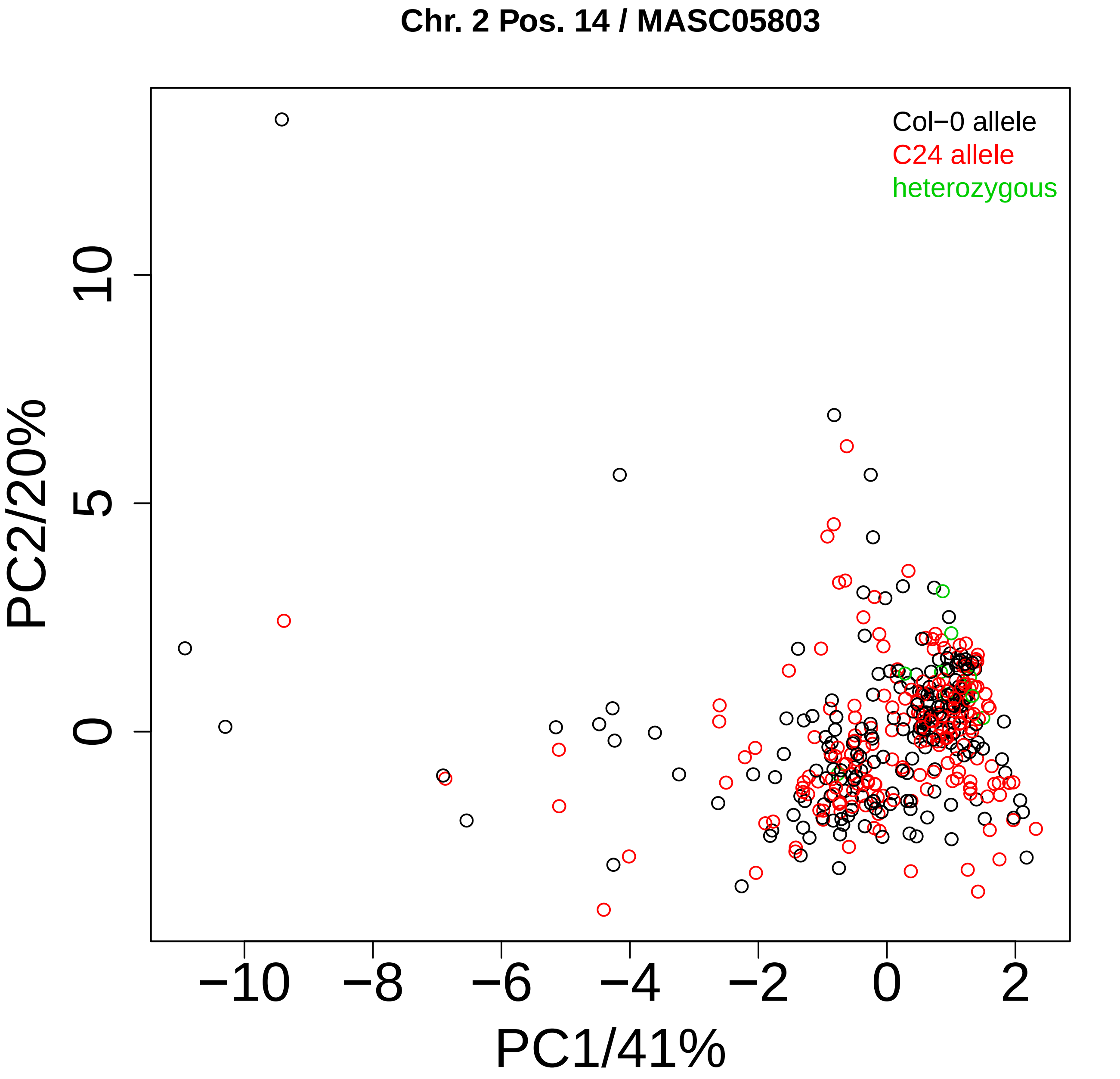
Chr. 2 Pos. 14 / MASC05803
Col−0 allele
C24 allele
heterozygous
PC2/20%
PC1/41%
0
5
10
−10
−8
−6
−4
−2
0
2

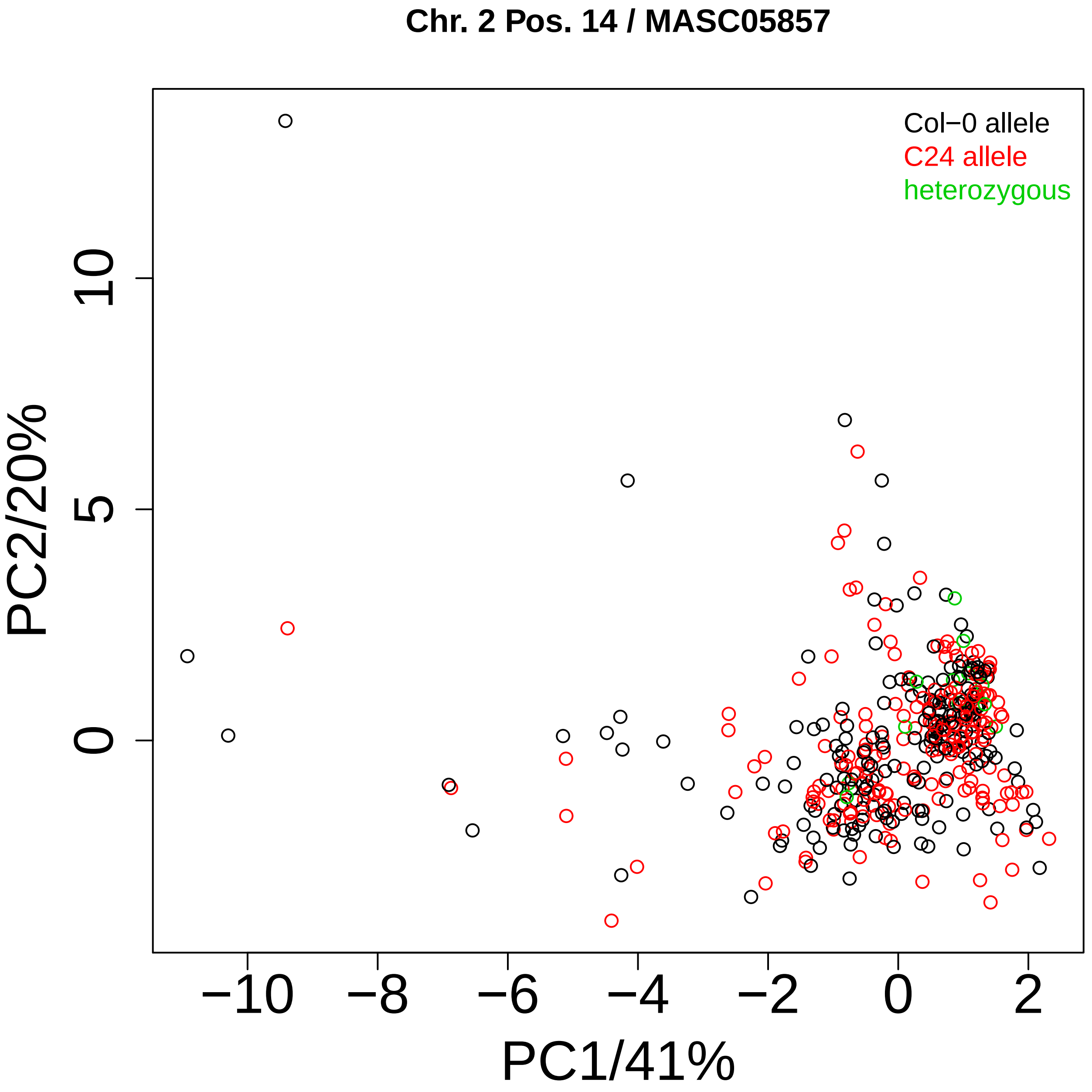
Chr. 2 Pos. 14 / MASC05857
Col−0 allele
C24 allele
heterozygous
PC2/20%
0
5
10
PC1/41%
−10
−8
−6
−4
−2
0
2

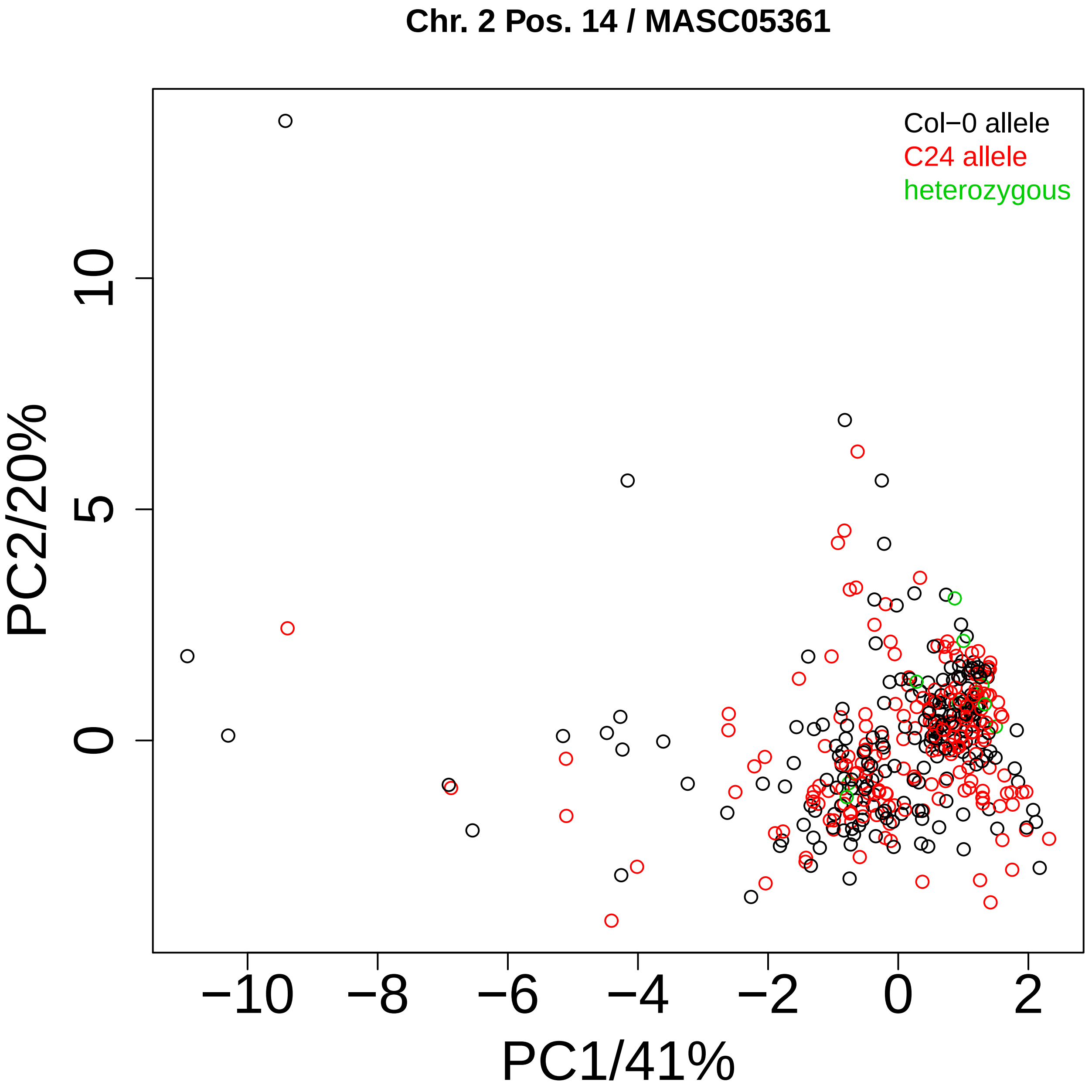

Chr. 2 Pos. 14 / MASC05361
Col−0 allele
C24 allele
heterozygous
PC2/20%
PC1/41%
0
5
10
−10
−8
−6
−4
−2
0
2

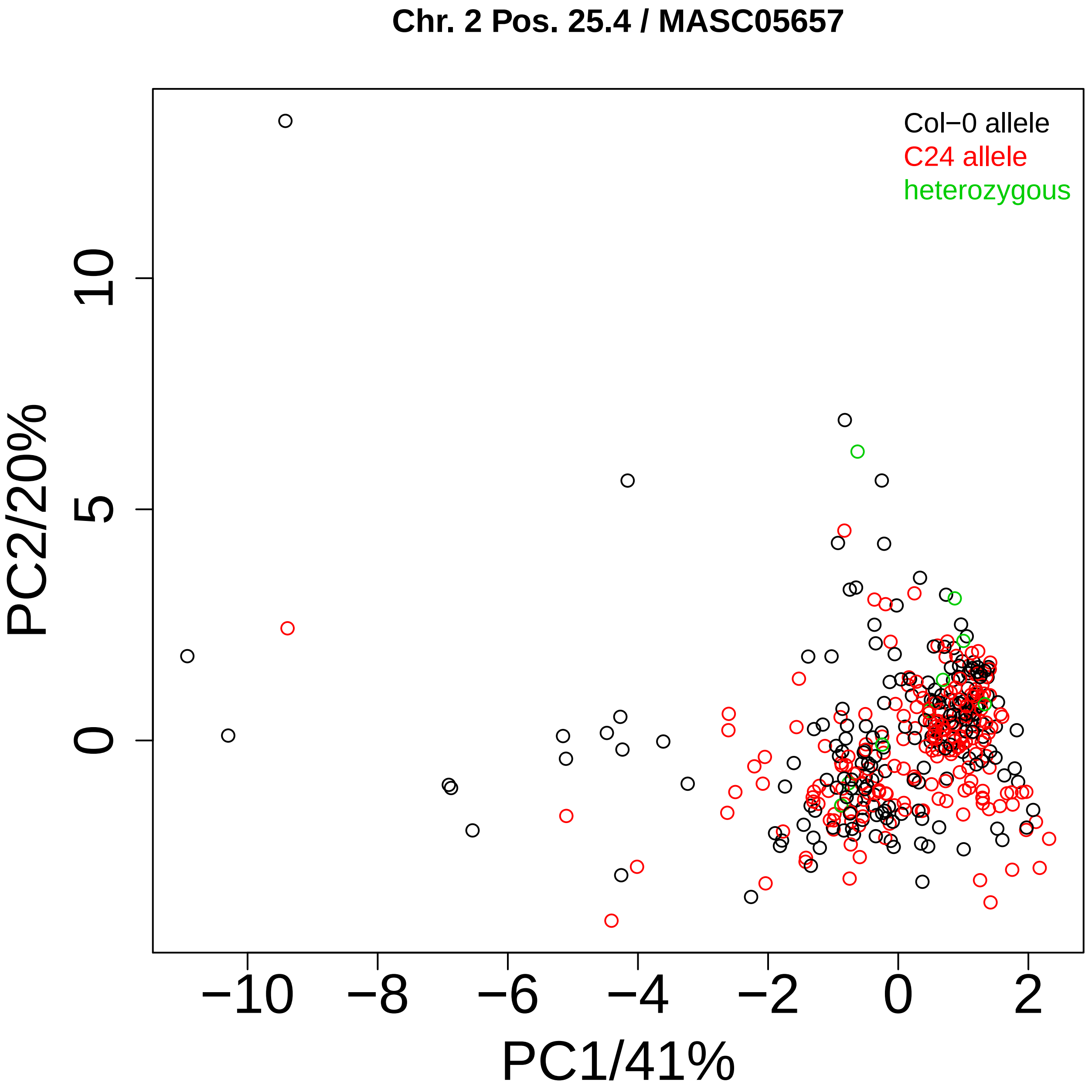
Chr. 2 Pos. 25.4 / MASC05657
Col−0 allele
C24 allele
heterozygous
PC2/20%
0
5
10
PC1/41%
−10
−8
−6
−4
−2
0
2

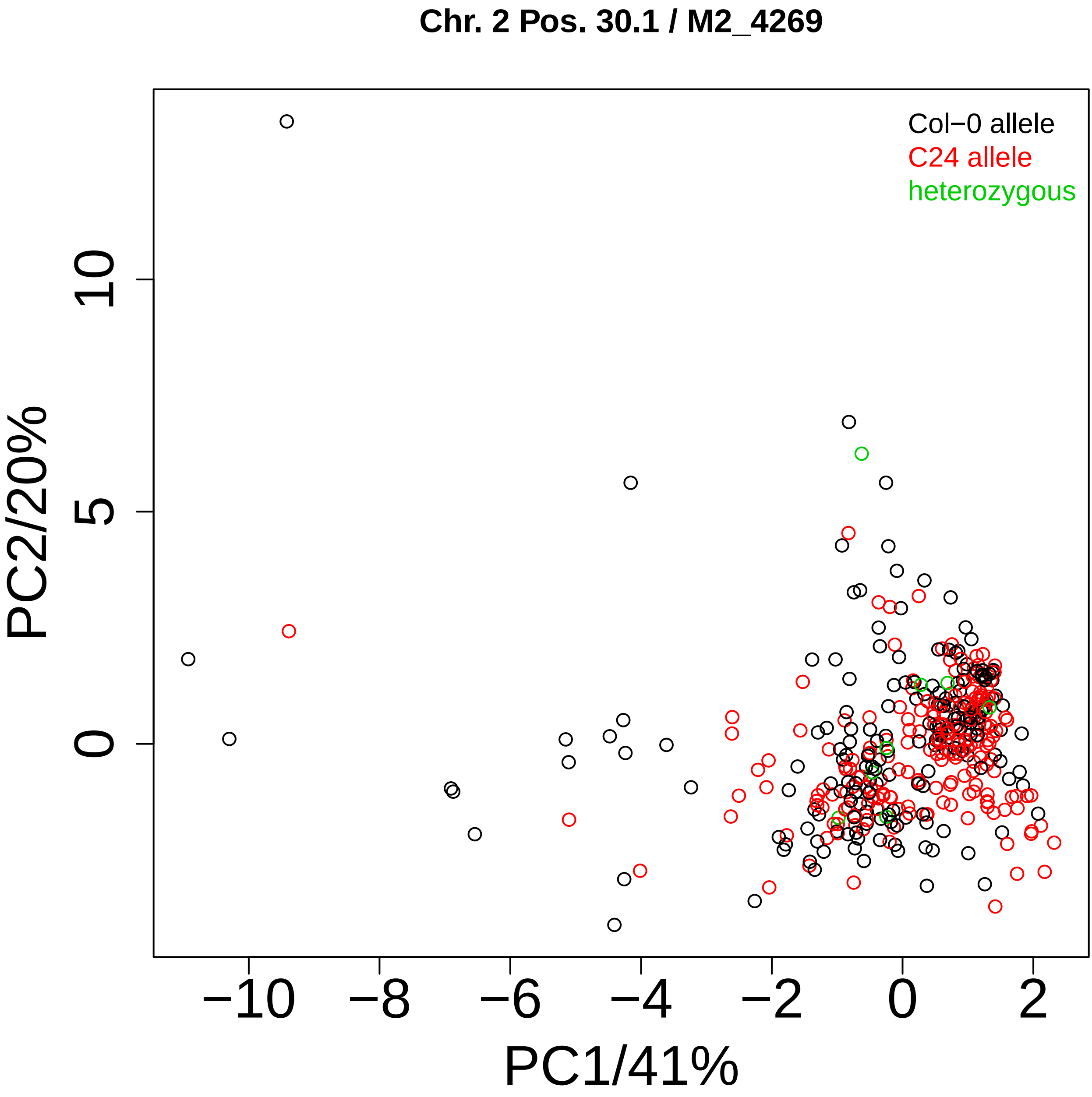
Chr. 2 Pos. 30.1 / M2_4269
Col−0 allele
C24 allele
heterozygous
PC1/41%
PC2/20%
−10
−8
−6
−4
−2
0
2
0
5
10

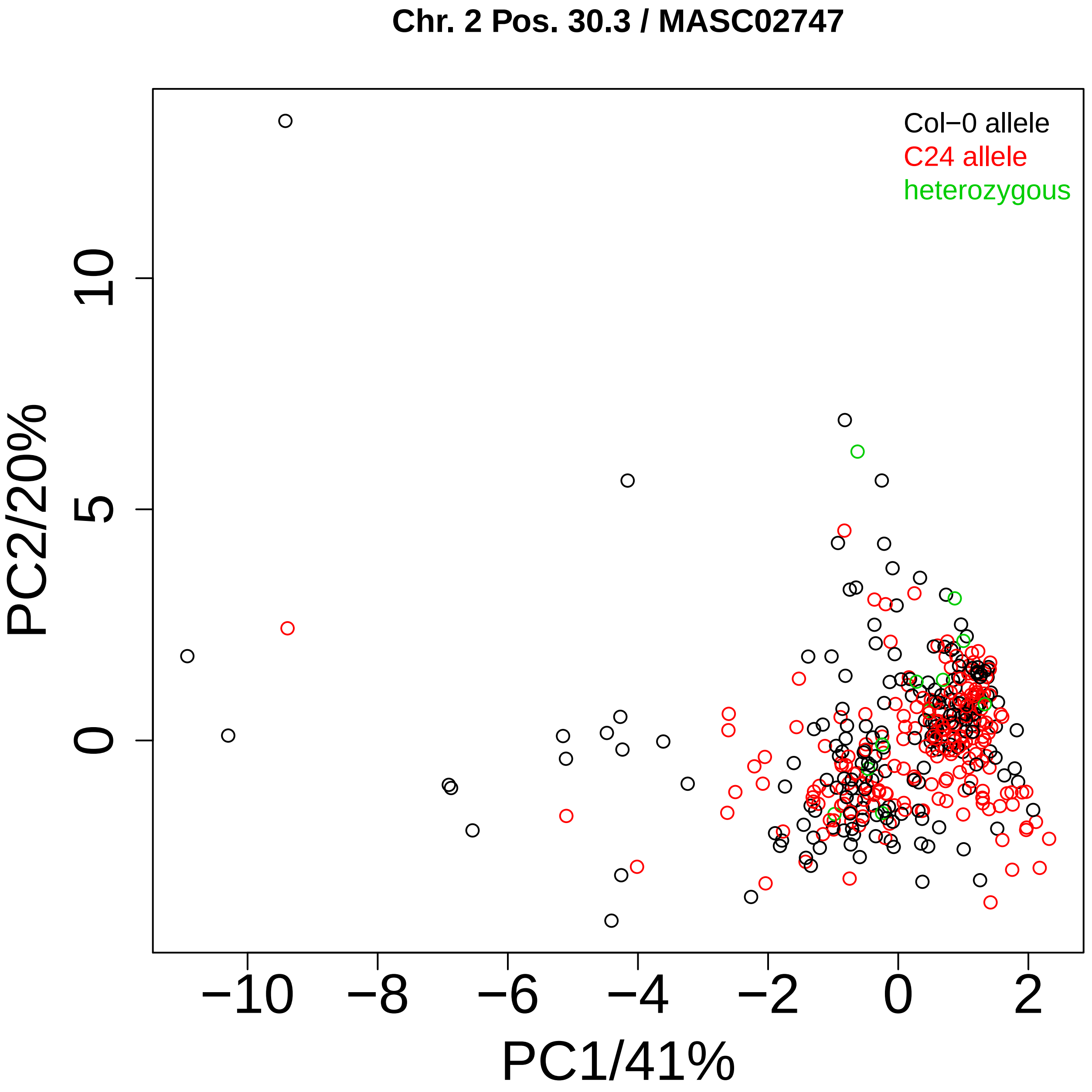
Chr. 2 Pos. 30.3 / MASC02747
Col−0 allele
C24 allele
heterozygous
PC2/20%
PC1/41%
0
5
10
−10
−8
−6
−4
−2
0
2

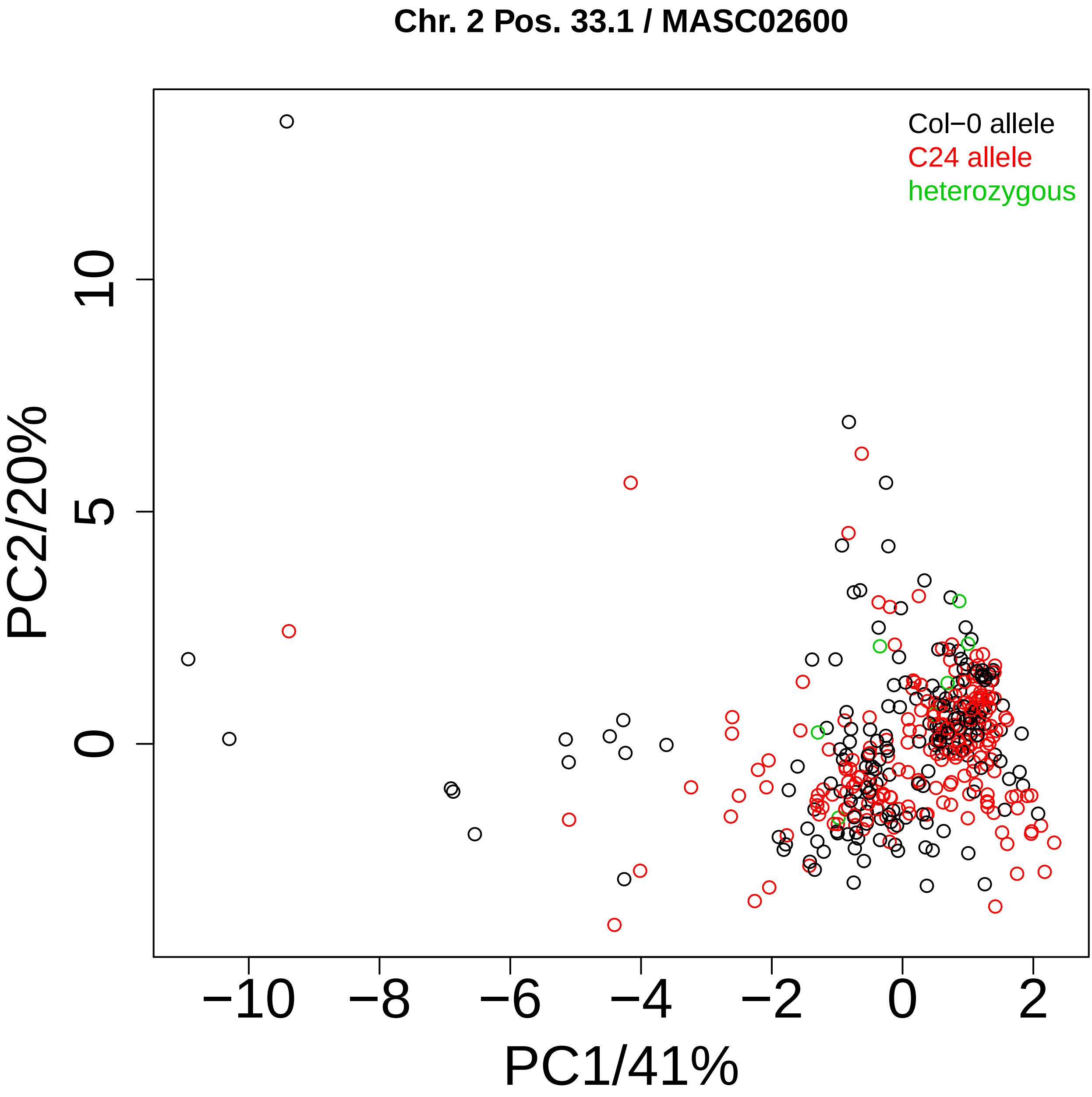
Chr. 2 Pos. 33.1 / MASC02600
Col−0 allele
C24 allele
heterozygous
PC2/20%
PC1/41%
10
5
0
−10
−8
−6
−4
−2
0
2

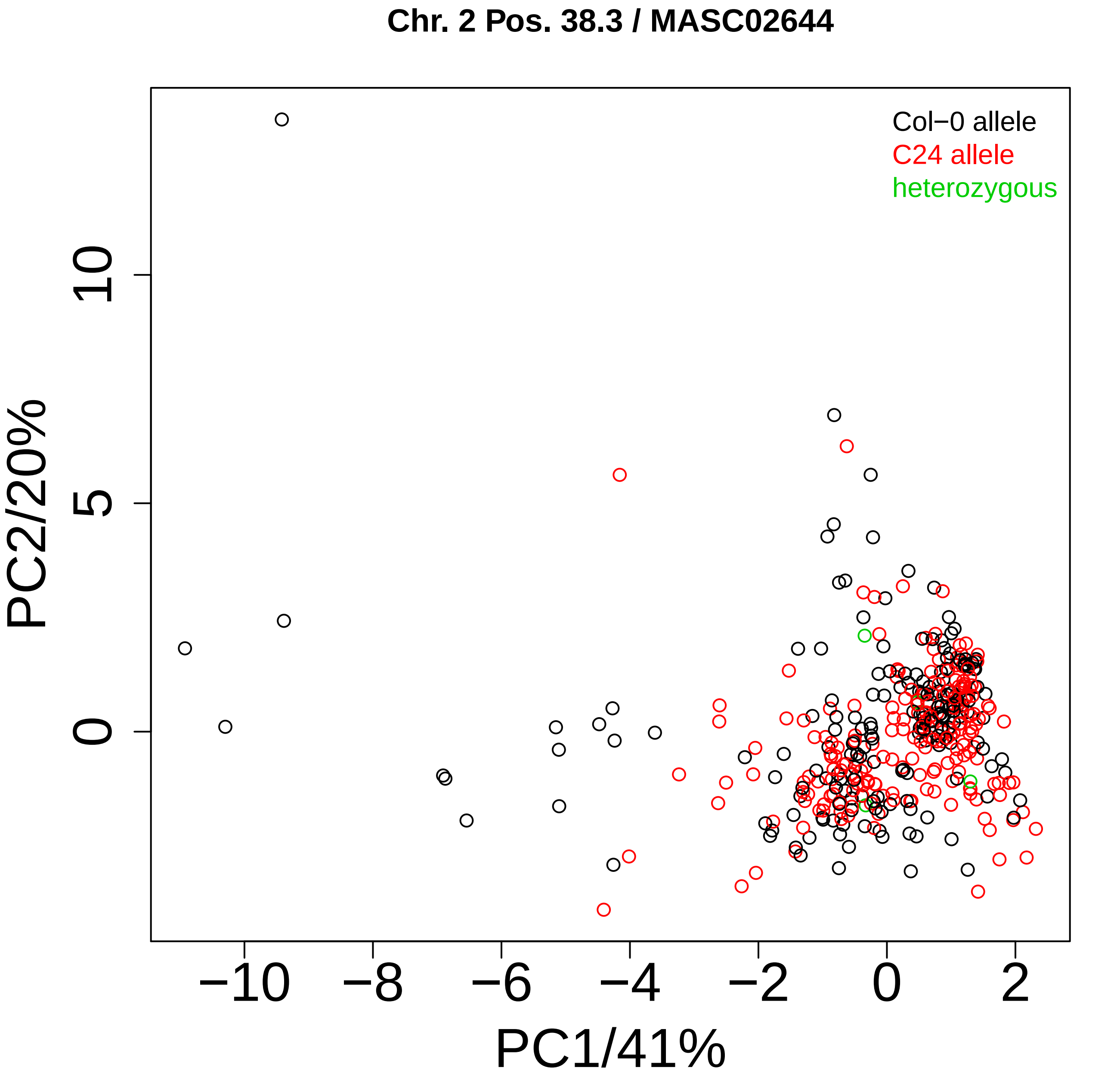
Chr. 2 Pos. 38.3 / MASC02644
Col−0 allele
C24 allele
heterozygous
PC2/20%
PC1/41%
10
5
0
−10
−8
−6
−4
−2
0
2

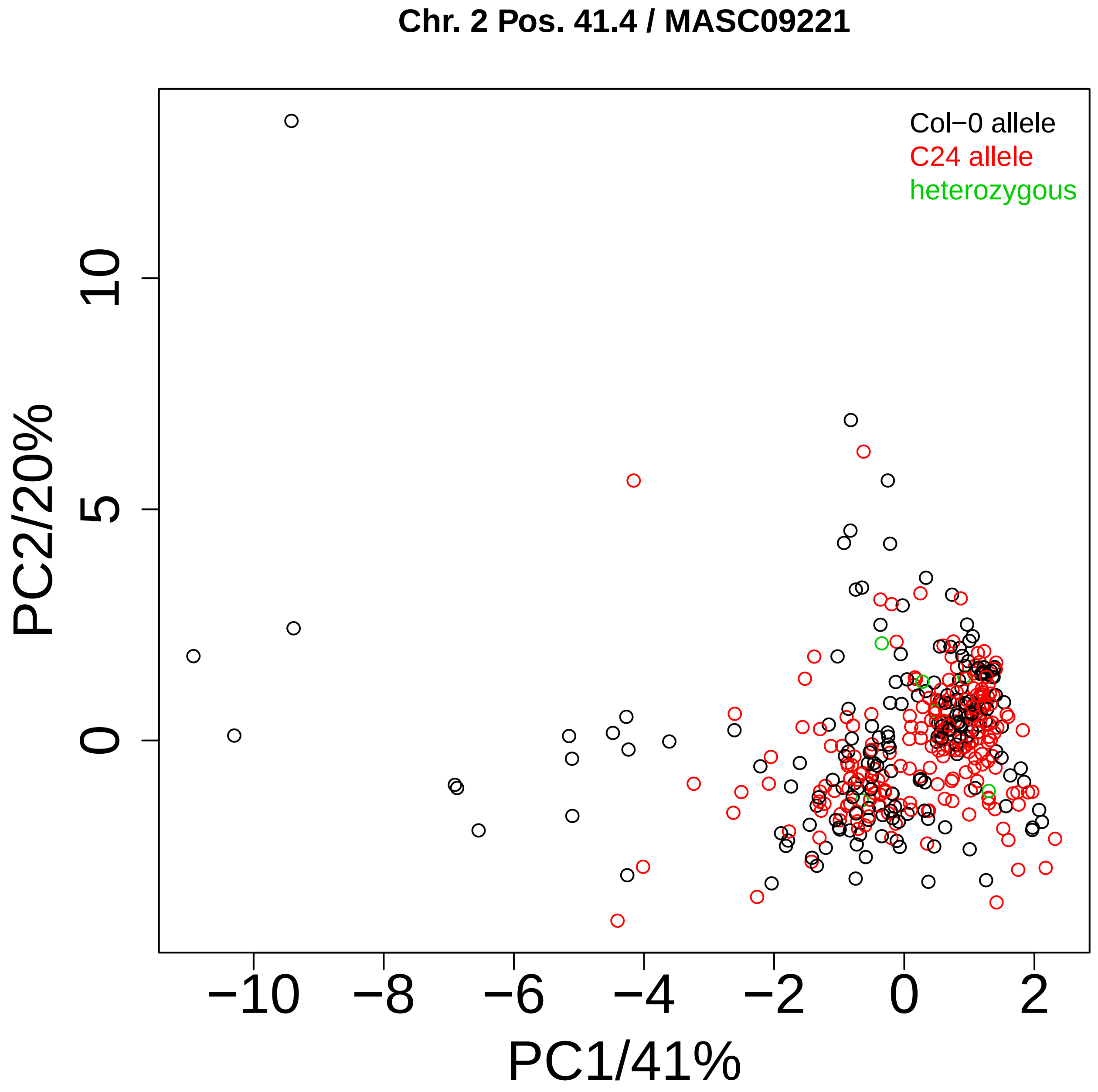
Chr. 2 Pos. 41.4 / MASC09221
Col−0 allele
C24 allele
heterozygous
PC2/20%
0
5
10
PC1/41%
−10
−8
−6
−4
−2
0
2

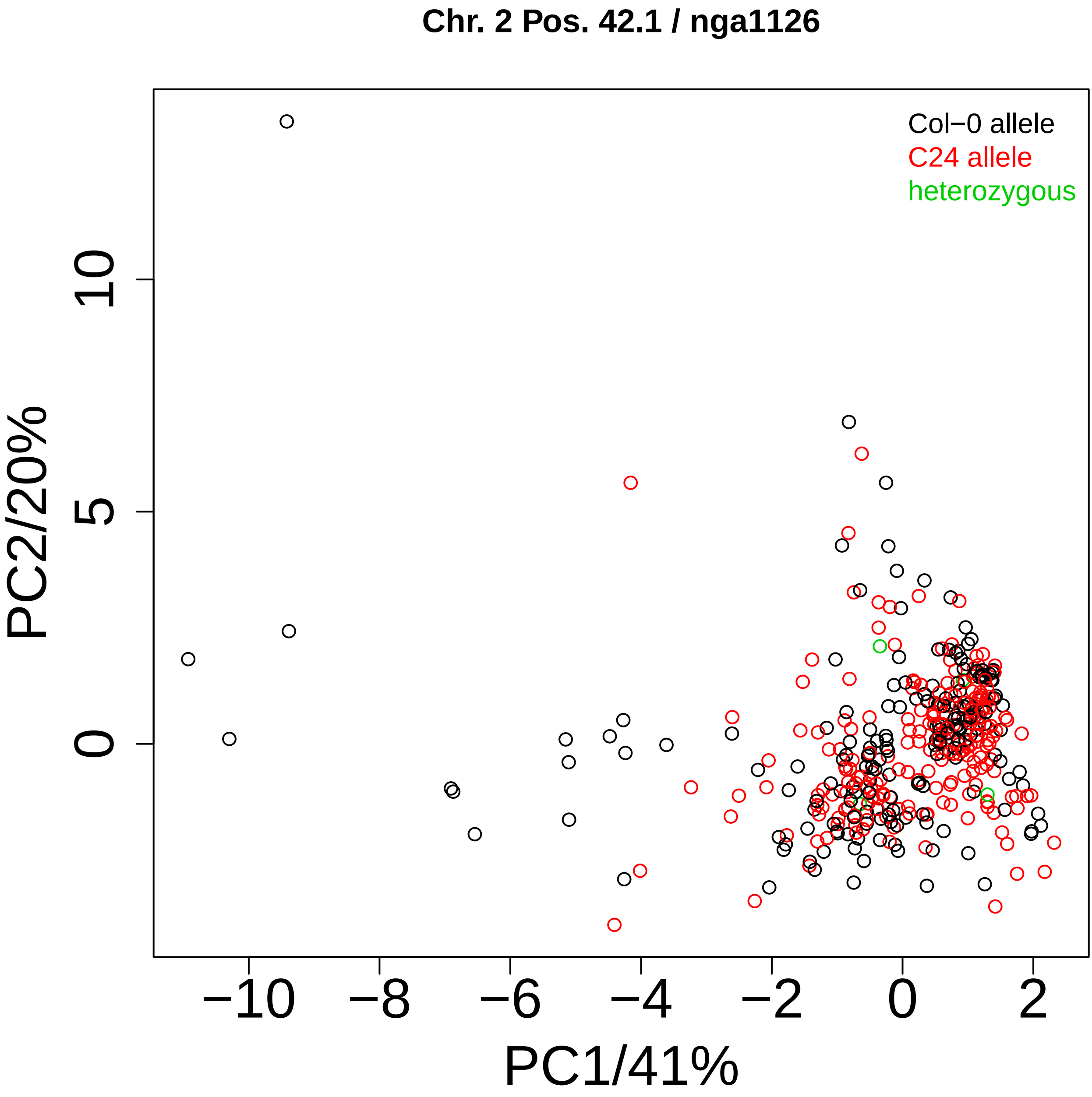

Chr. 2 Pos. 42.1 / nga1126
Col−0 allele
C24 allele
heterozygous
PC2/20%
PC1/41%
10
5
0
−10
−8
−6
−4
−2
0
2

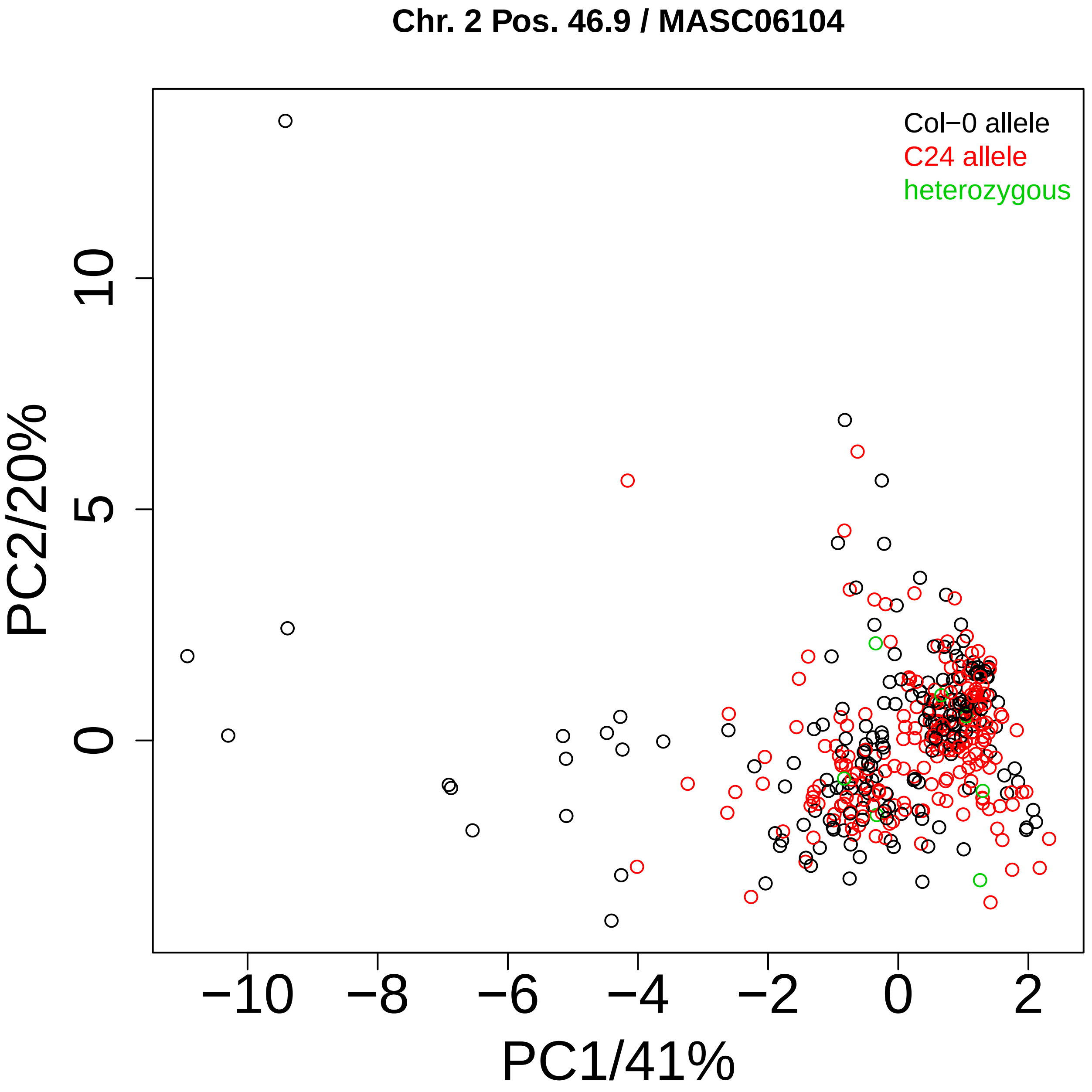

Chr. 2 Pos. 46.9 / MASC06104
Col−0 allele
C24 allele
heterozygous
PC2/20%
0
5
10
PC1/41%
−10
−8
−6
−4
−2
0
2

Chr. 2 Pos. 50.6 / MASC05434

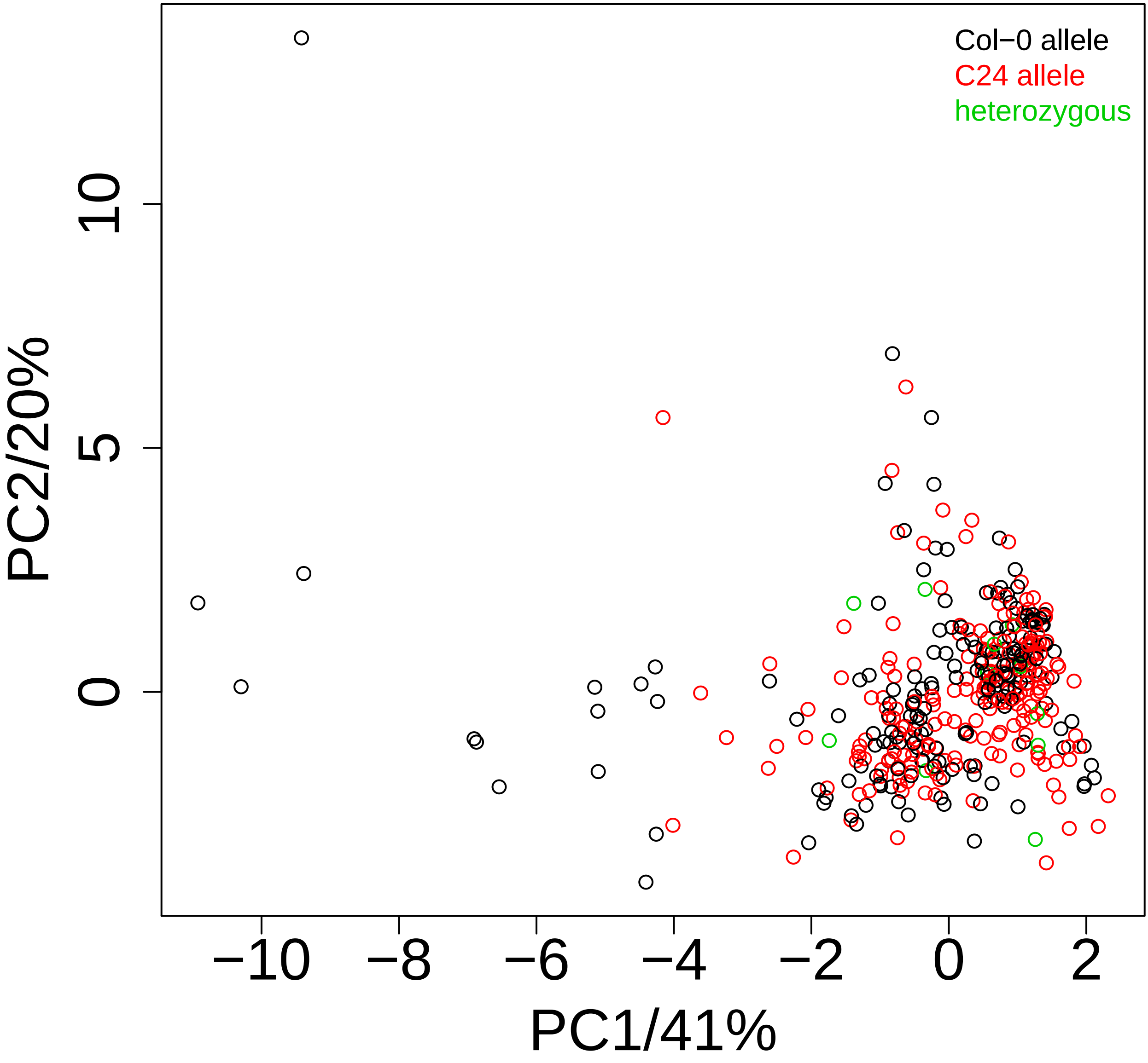

Chr. 2 Pos. 53.1 / MASC09222

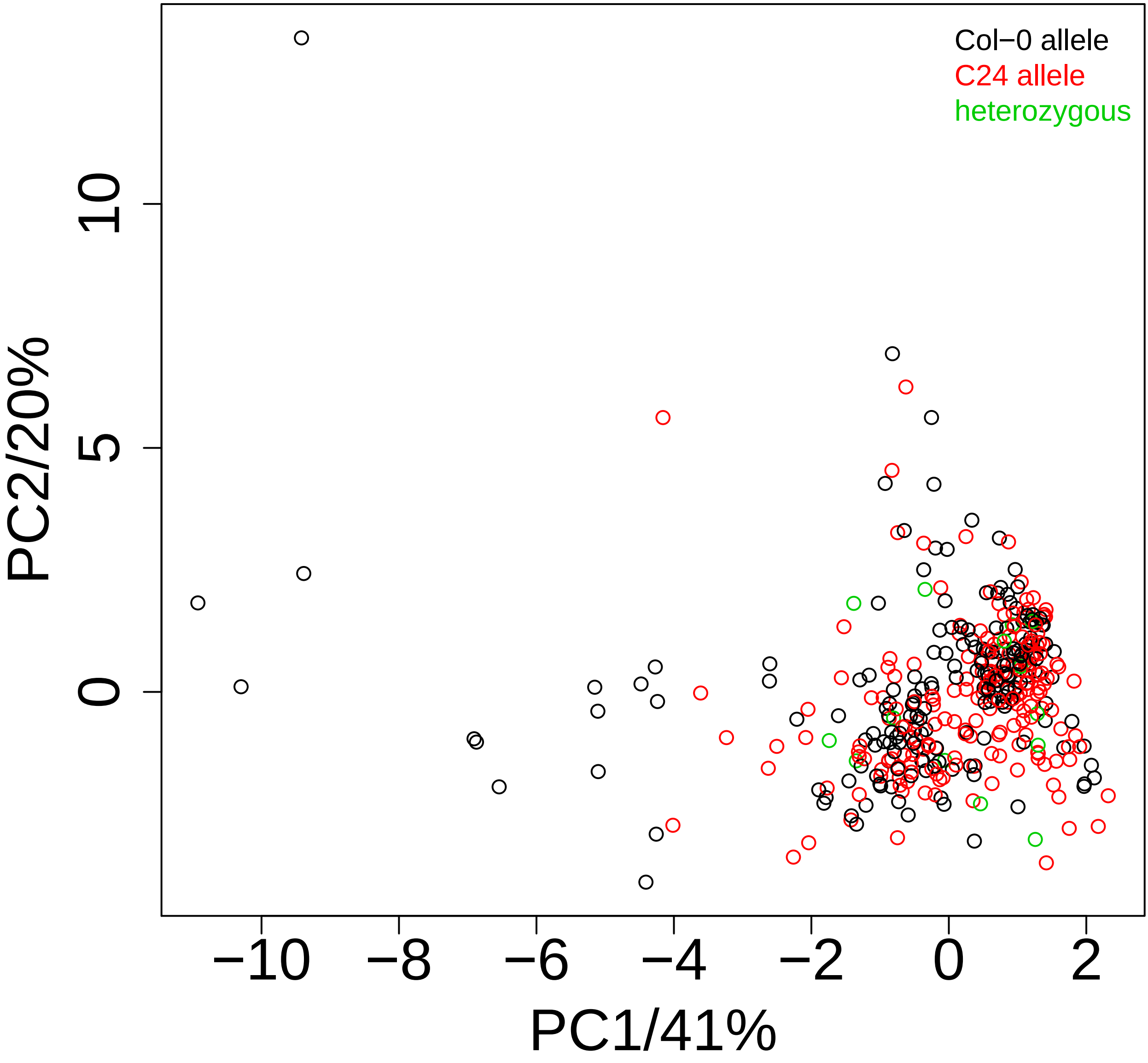

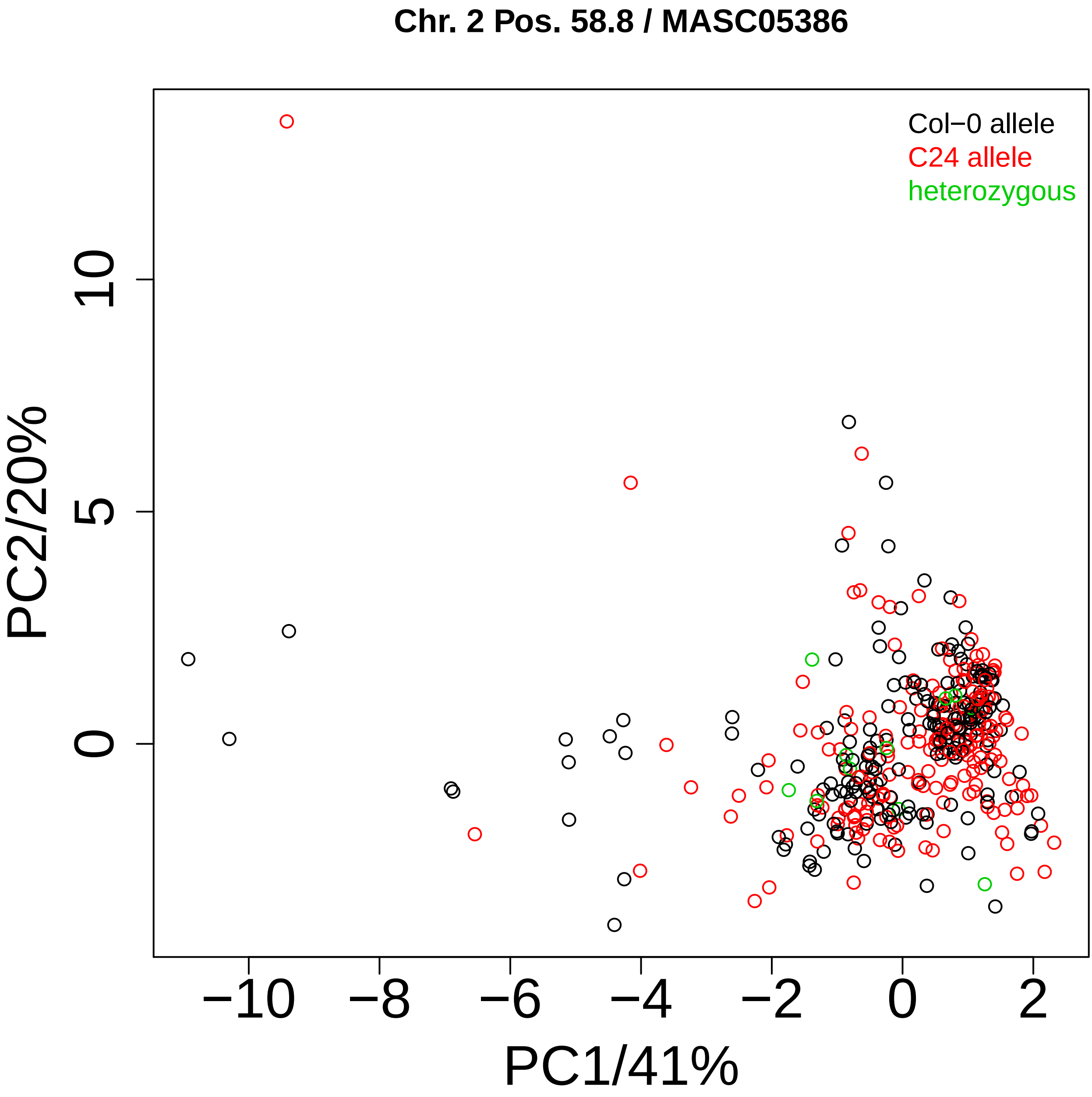

Chr. 2 Pos. 58.8 / MASC05386
Col−0 allele
C24 allele
heterozygous
PC2/20%
0
5
10
PC1/41%
−10
−8
−6
−4
−2
0
2

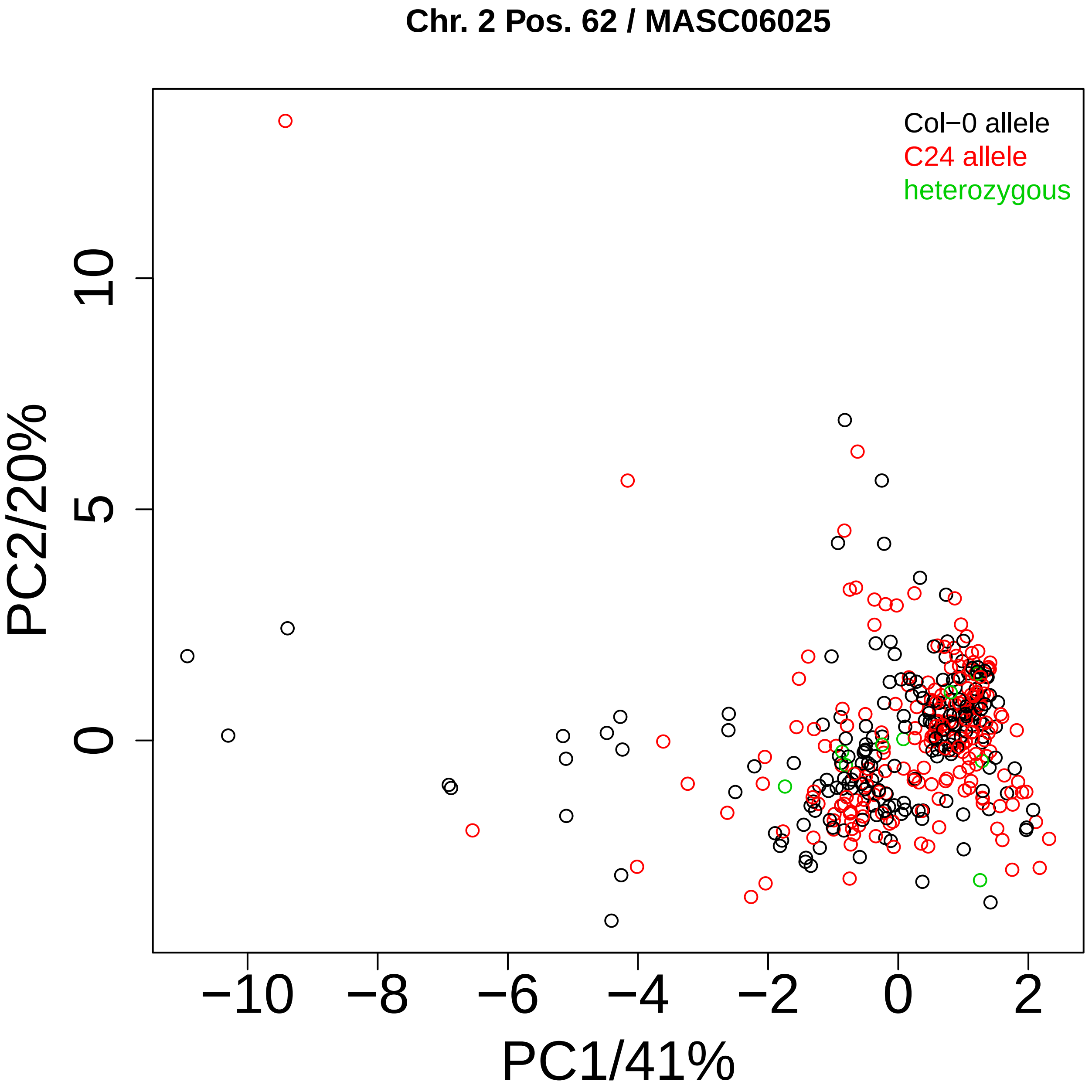
Chr. 2 Pos. 62 / MASC06025
Col−0 allele
C24 allele
heterozygous
PC2/20%
0
5
10
PC1/41%
−10
−8
−6
−4
−2
0
2

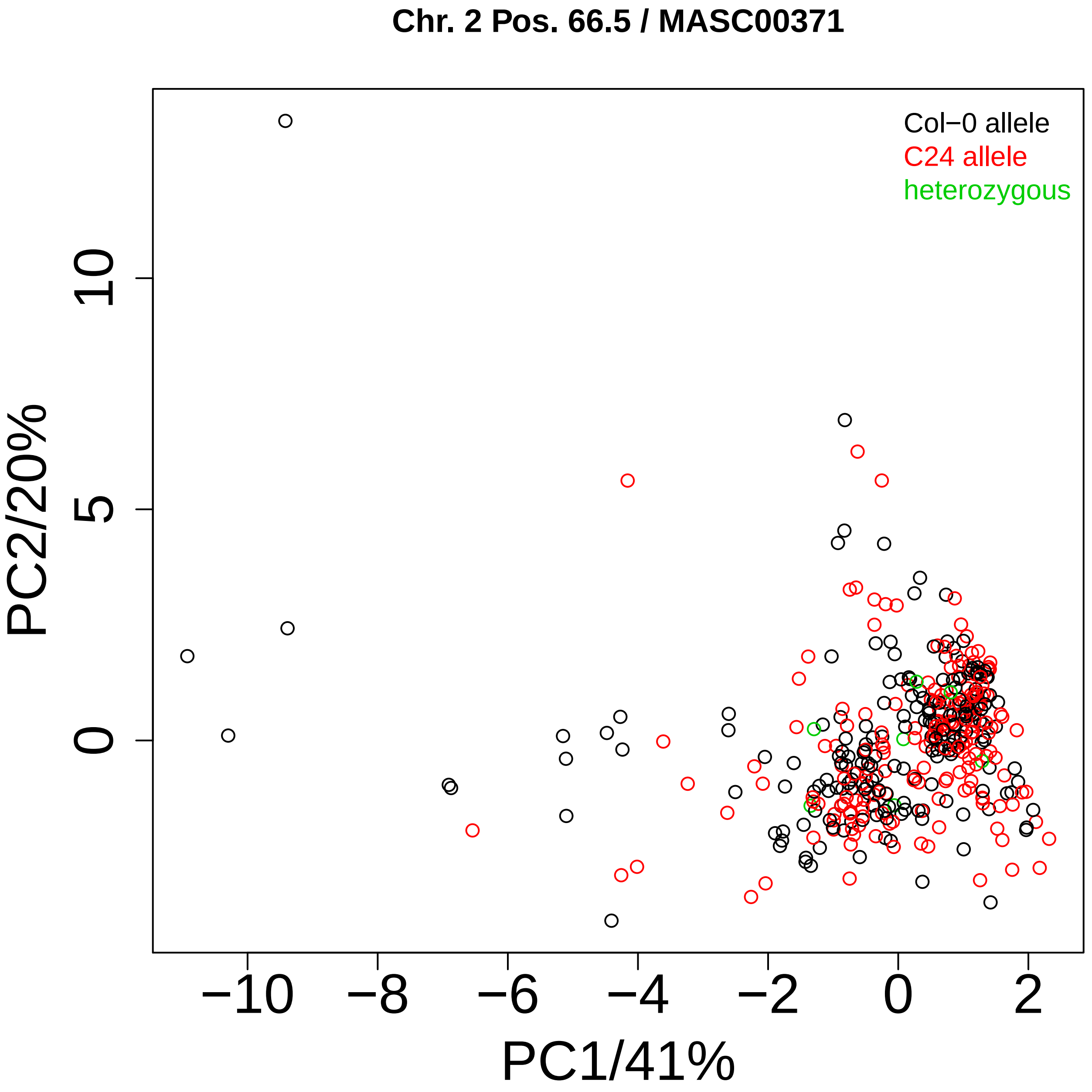
Chr. 2 Pos. 66.5 / MASC00371
Col−0 allele
C24 allele
heterozygous
PC2/20%
PC1/41%
10
5
0
−10
−8
−6
−4
−2
0
2

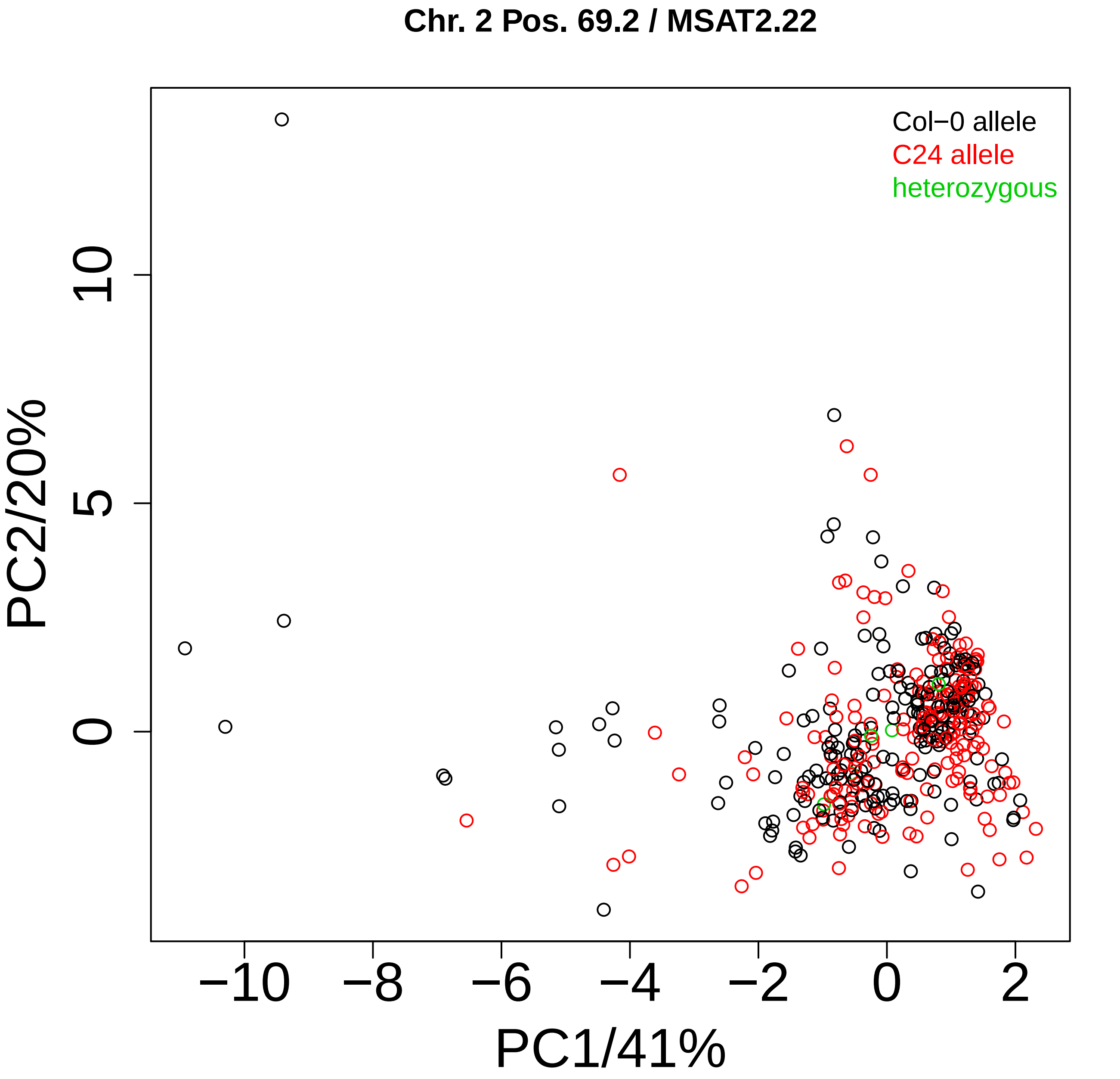
Chr. 2 Pos. 69.2 / MSAT2.22
Col−0 allele
C24 allele
heterozygous
PC2/20%
PC1/41%
10
5
0
−10
−8
−6
−4
−2
0
2

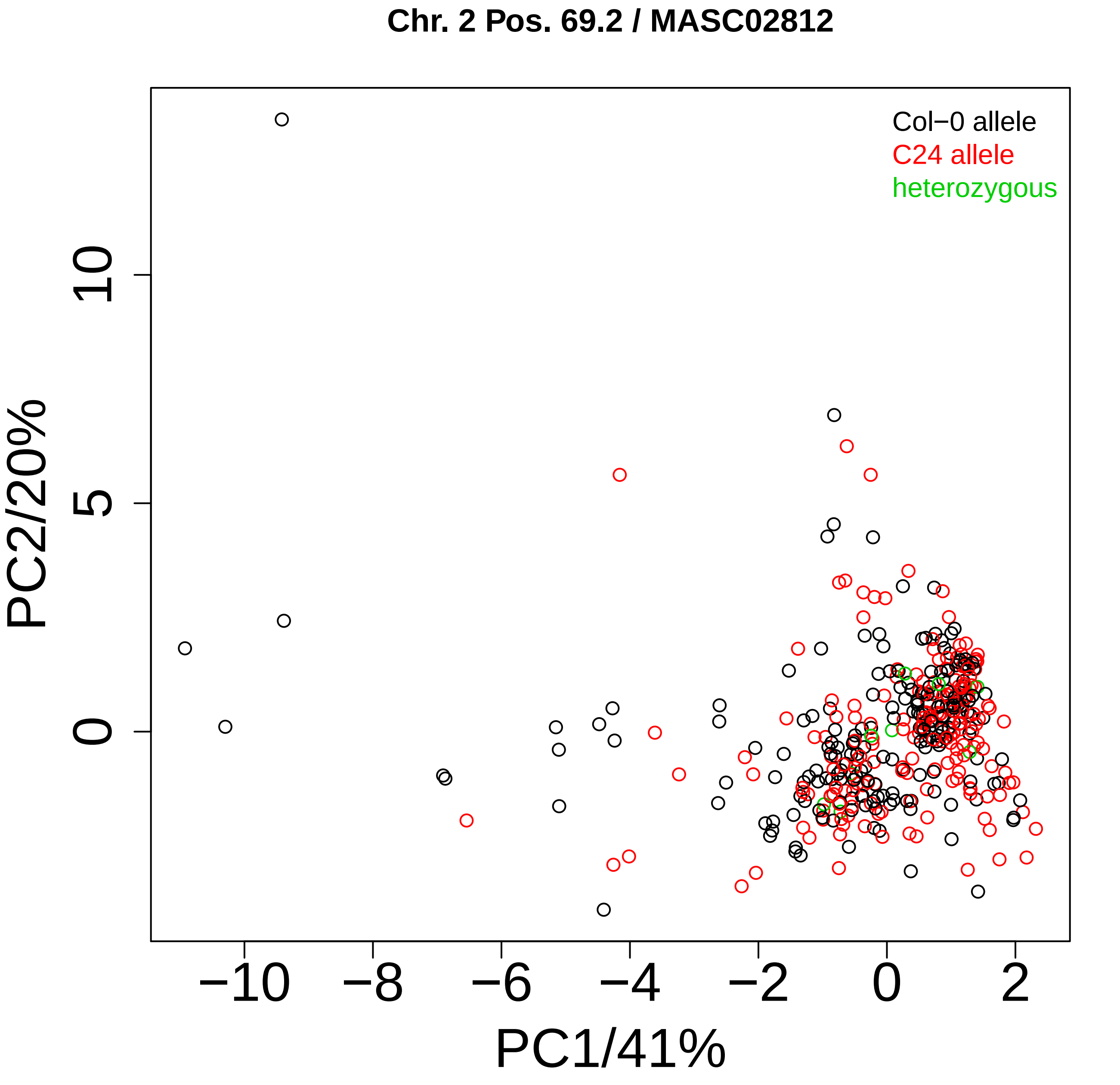

Chr. 2 Pos. 69.2 / MASC02812
Col−0 allele
C24 allele
heterozygous
PC2/20%
10
5
0
−10
−8
−6
−4
−2
0
2
PC1/41%

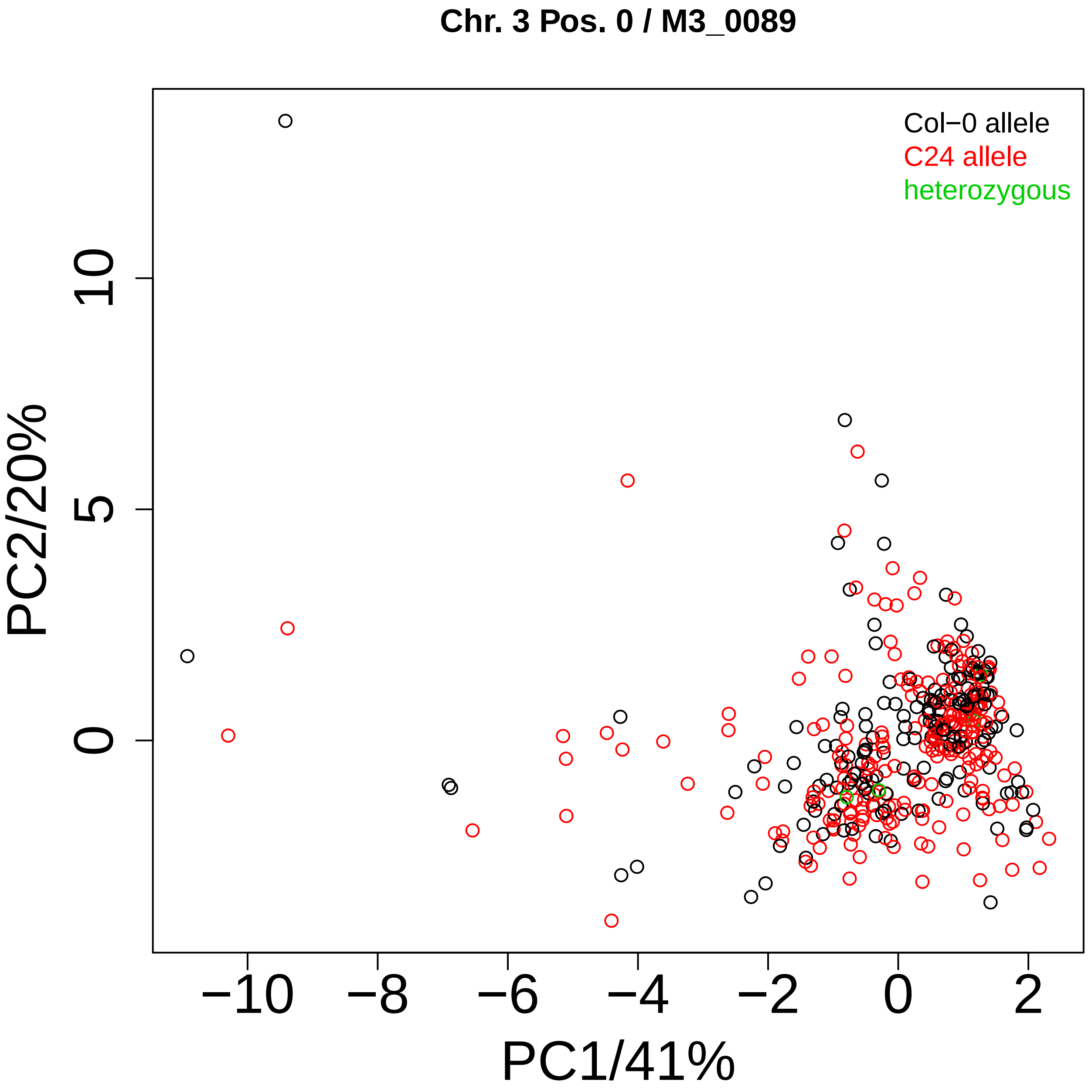

Chr. 3 Pos. 0 / M3_0089
Col−0 allele
C24 allele
heterozygous
PC2/20%
10
5
0
PC1/41%
−10
−8
−6
−4
−2
0
2

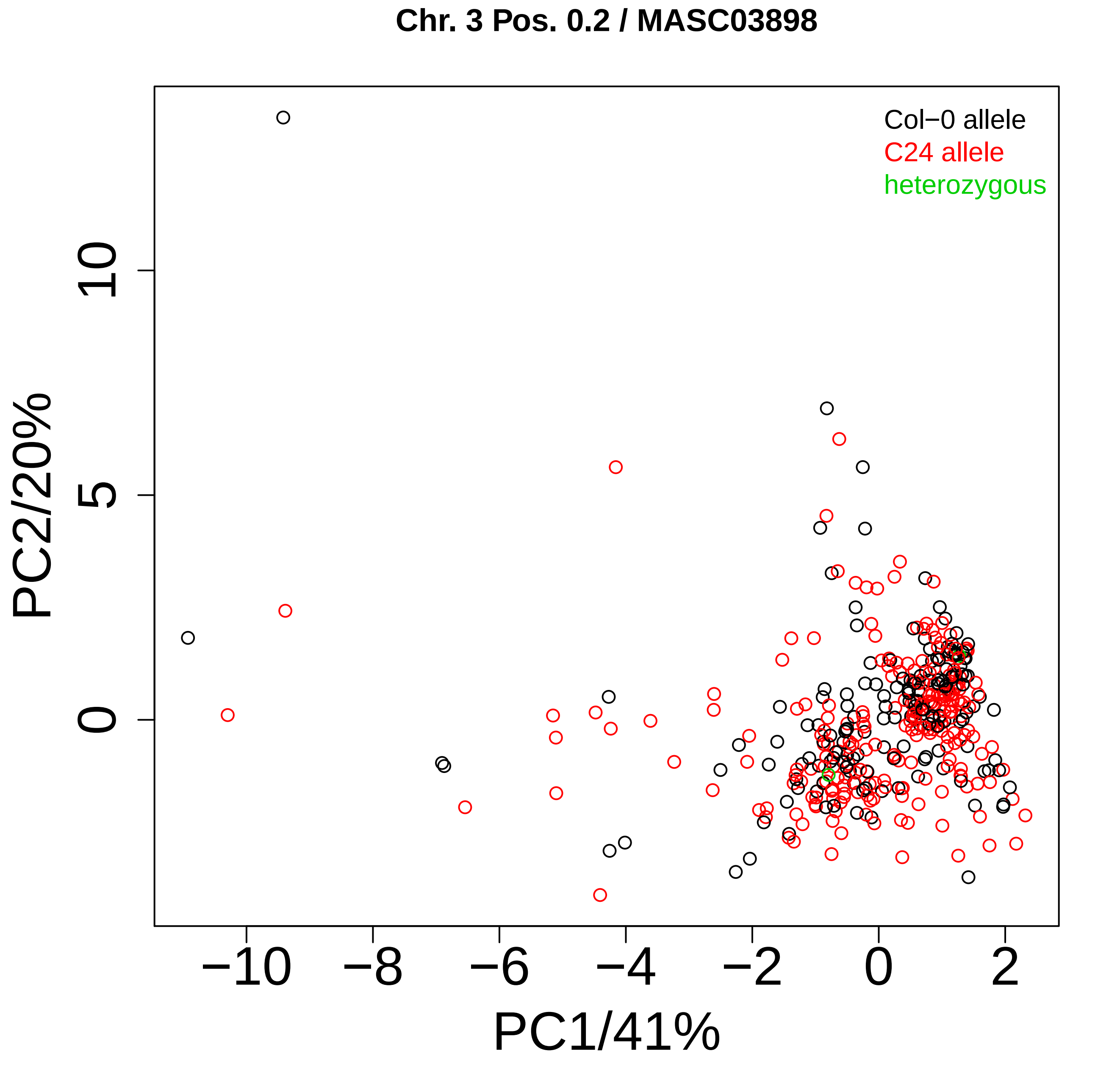

Chr. 3 Pos. 0.2 / MASC03898
Col−0 allele
C24 allele
heterozygous
PC2/20%
0
5
10
PC1/41%
−10
−8
−6
−4
−2
0
2

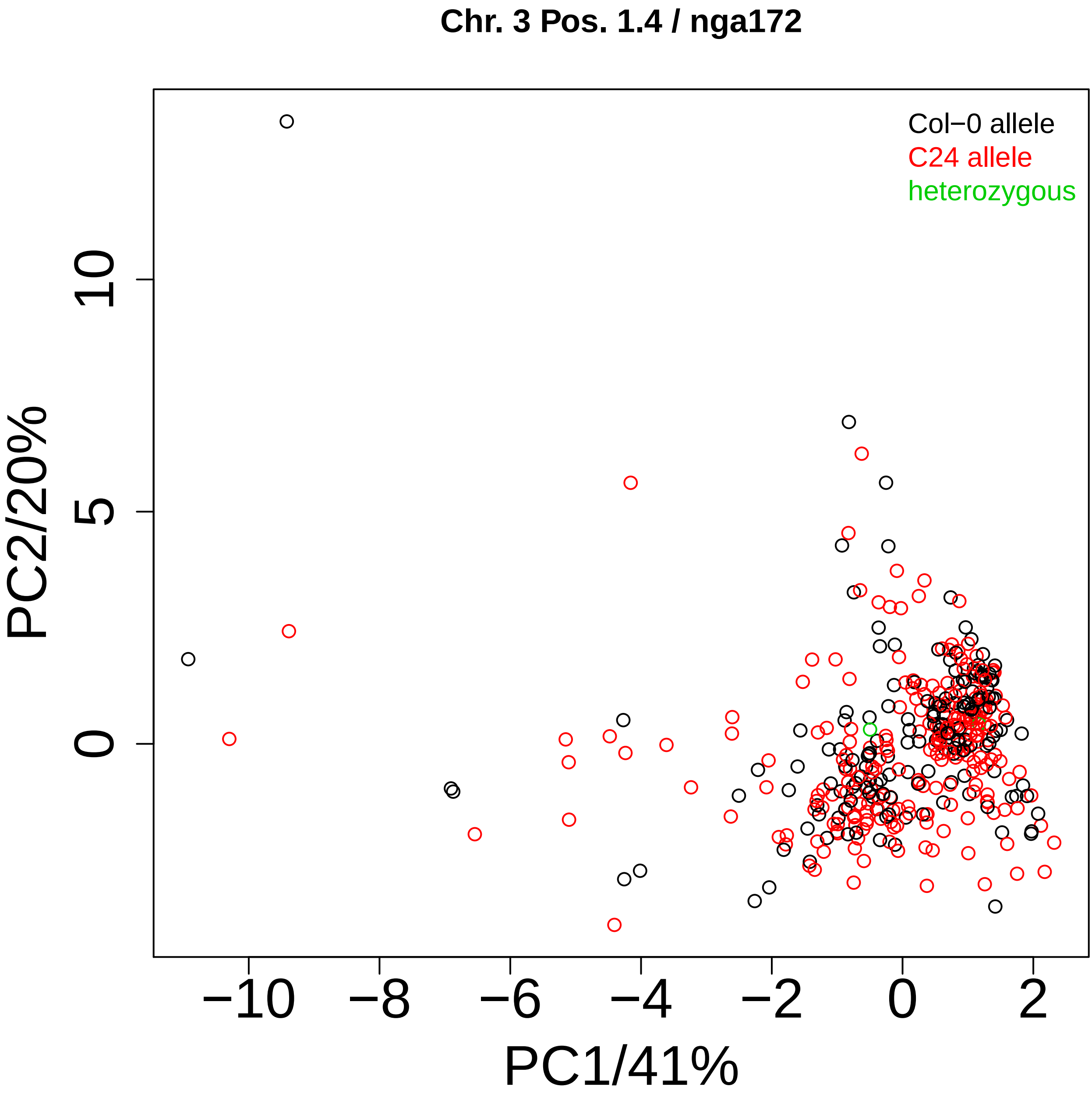
Chr. 3 Pos. 1.4 / nga172
Col−0 allele
C24 allele
heterozygous
PC2/20%
10
5
0
PC1/41%
−10
−8
−6
−4
−2
0
2

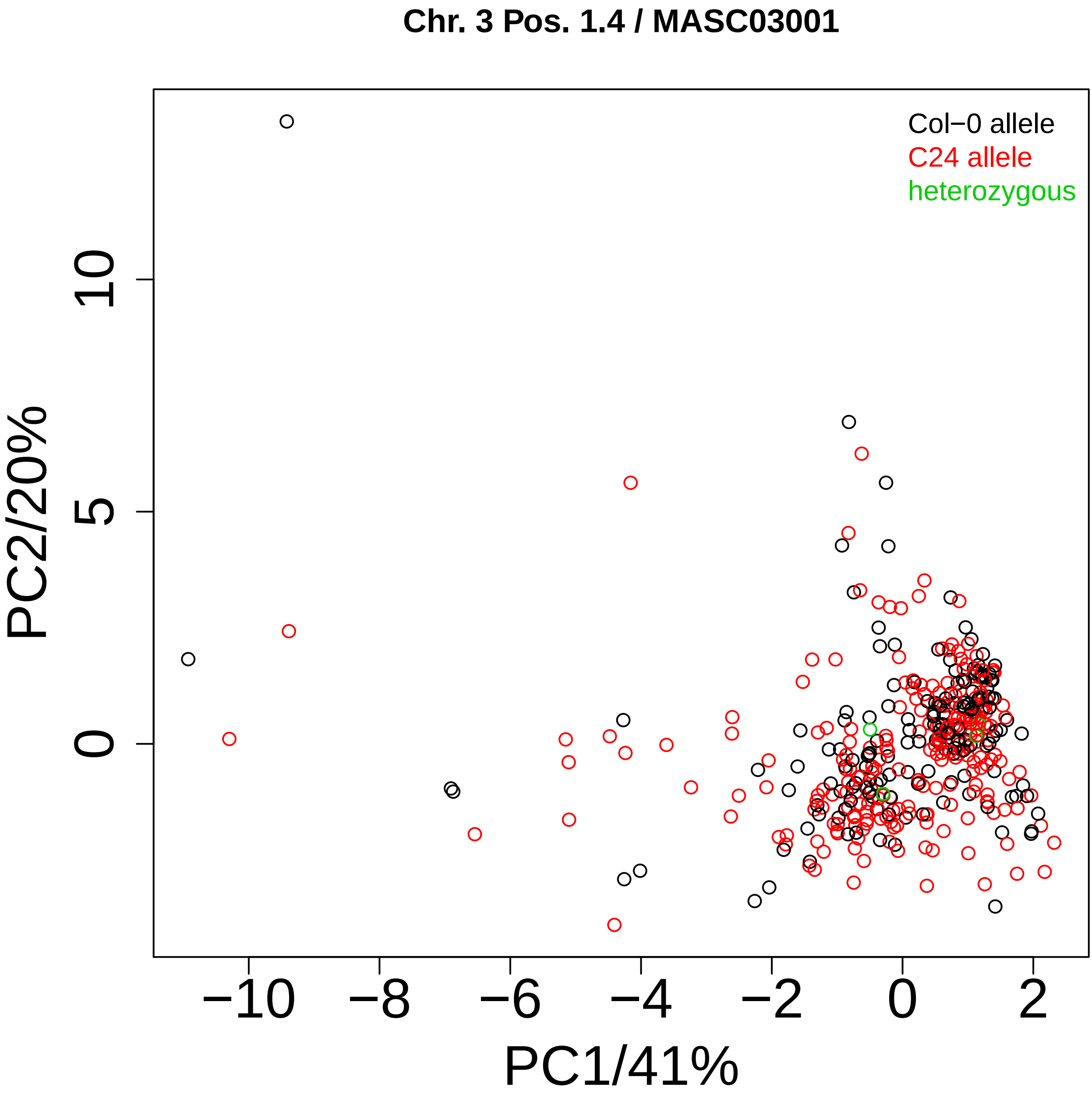

Chr. 3 Pos. 1.4 / MASC03001
Col−0 allele
C24 allele
heterozygous
PC2/20%
0
5
10
PC1/41%
−10
−8
−6
−4
−2
0
2

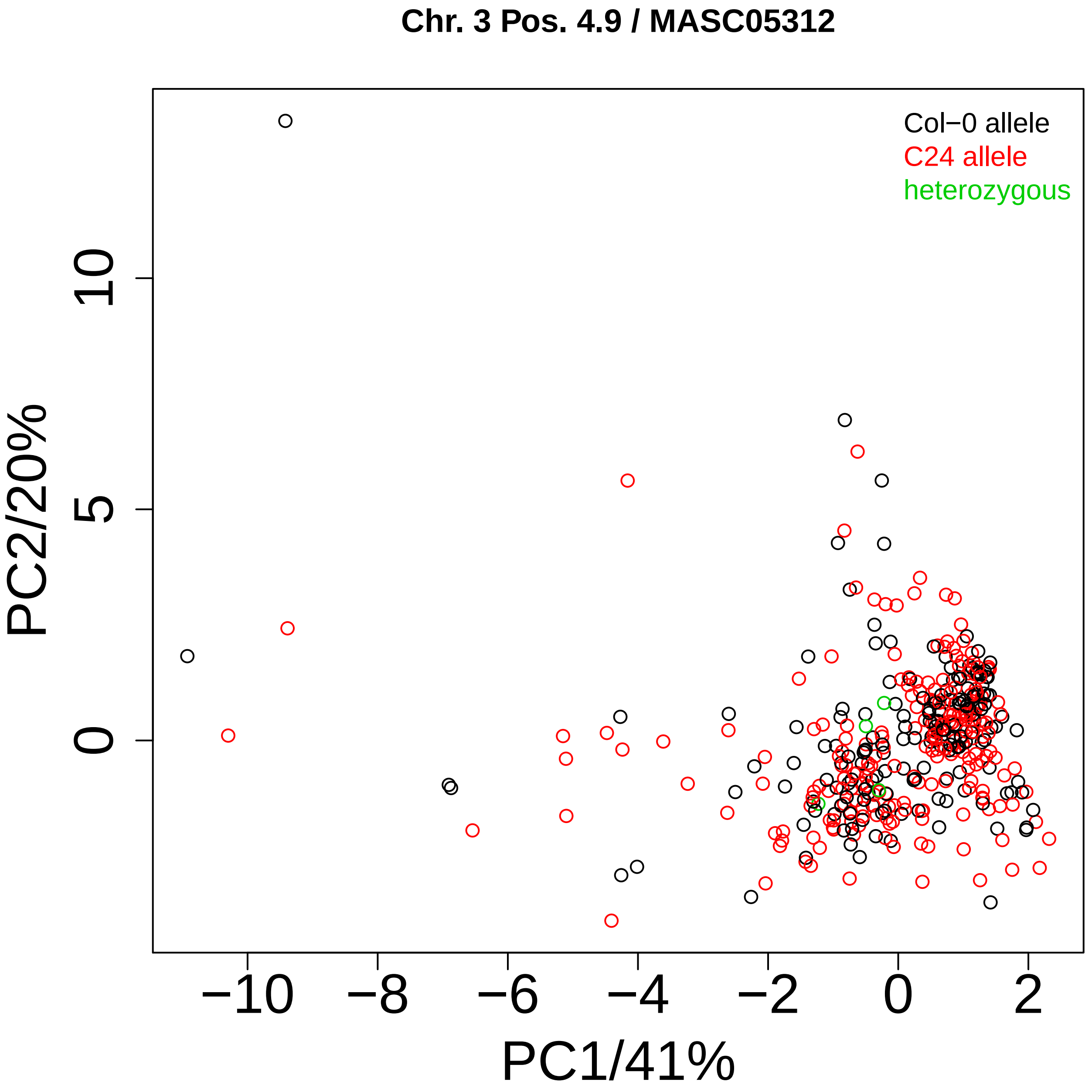

Chr. 3 Pos. 4.9 / MASC05312
Col−0 allele
C24 allele
heterozygous
10
5
0
PC2/20%
−10
−8
−6
−4
−2
0
2
PC1/41%

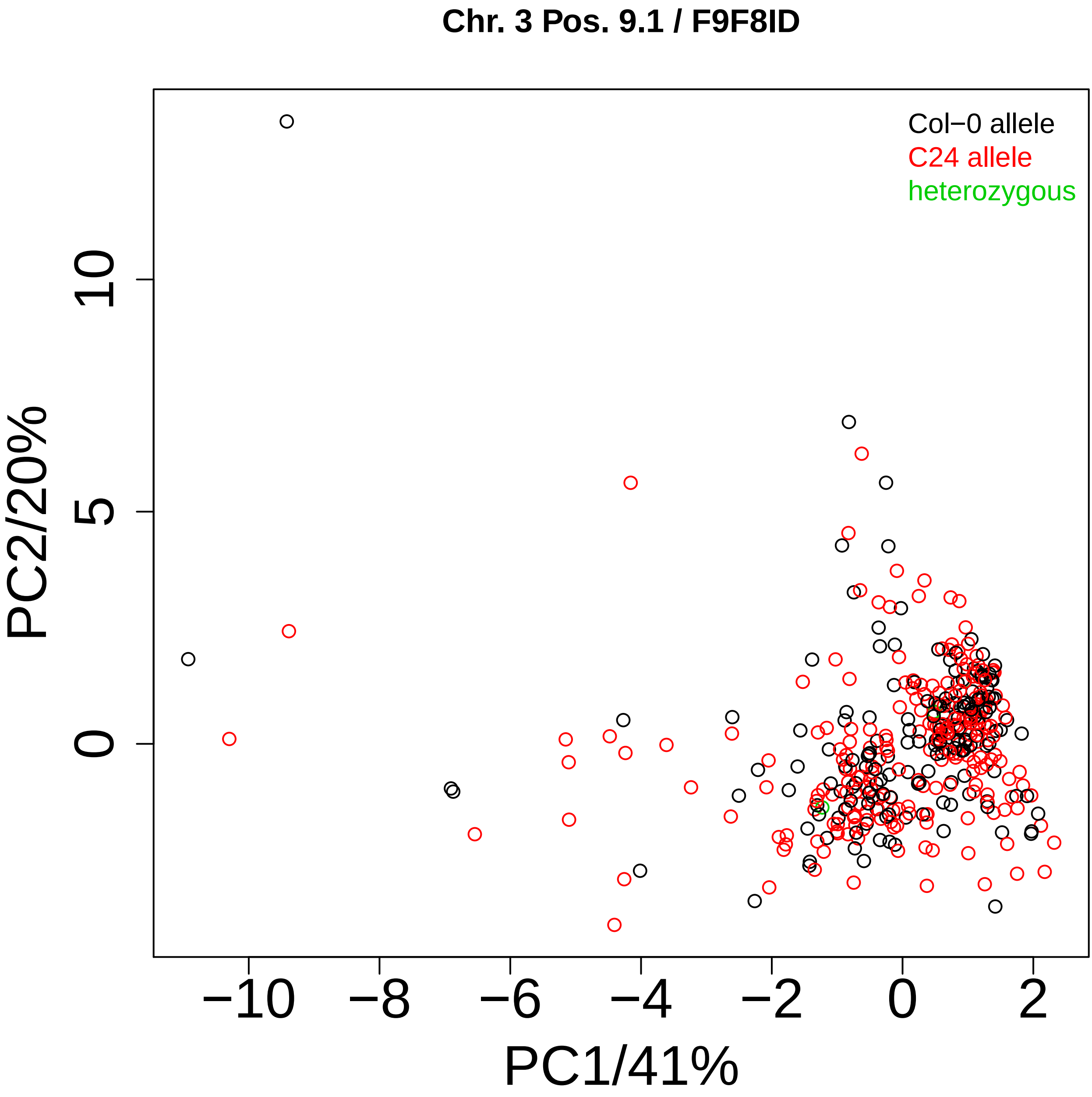

Chr. 3 Pos. 9.1 / F9F8ID
Col−0 allele
C24 allele
heterozygous
10
5
0
PC2/20%
−10
−8
−6
−4
−2
0
2
PC1/41%

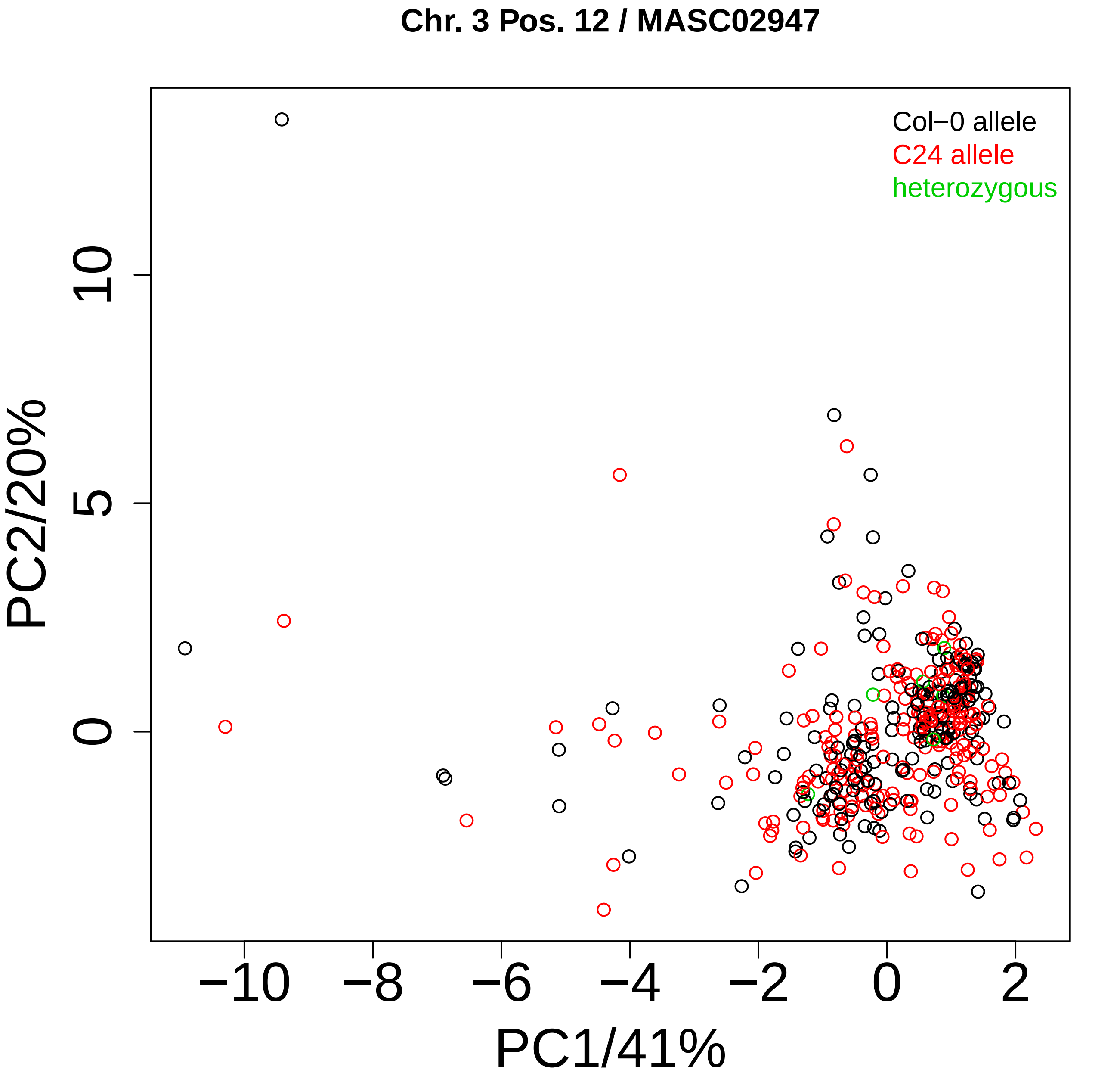
Chr. 3 Pos. 12 / MASC02947
Col−0 allele
C24 allele
heterozygous
PC2/20%
0
5
10
PC1/41%
−10
−8
−6
−4
−2
0
2

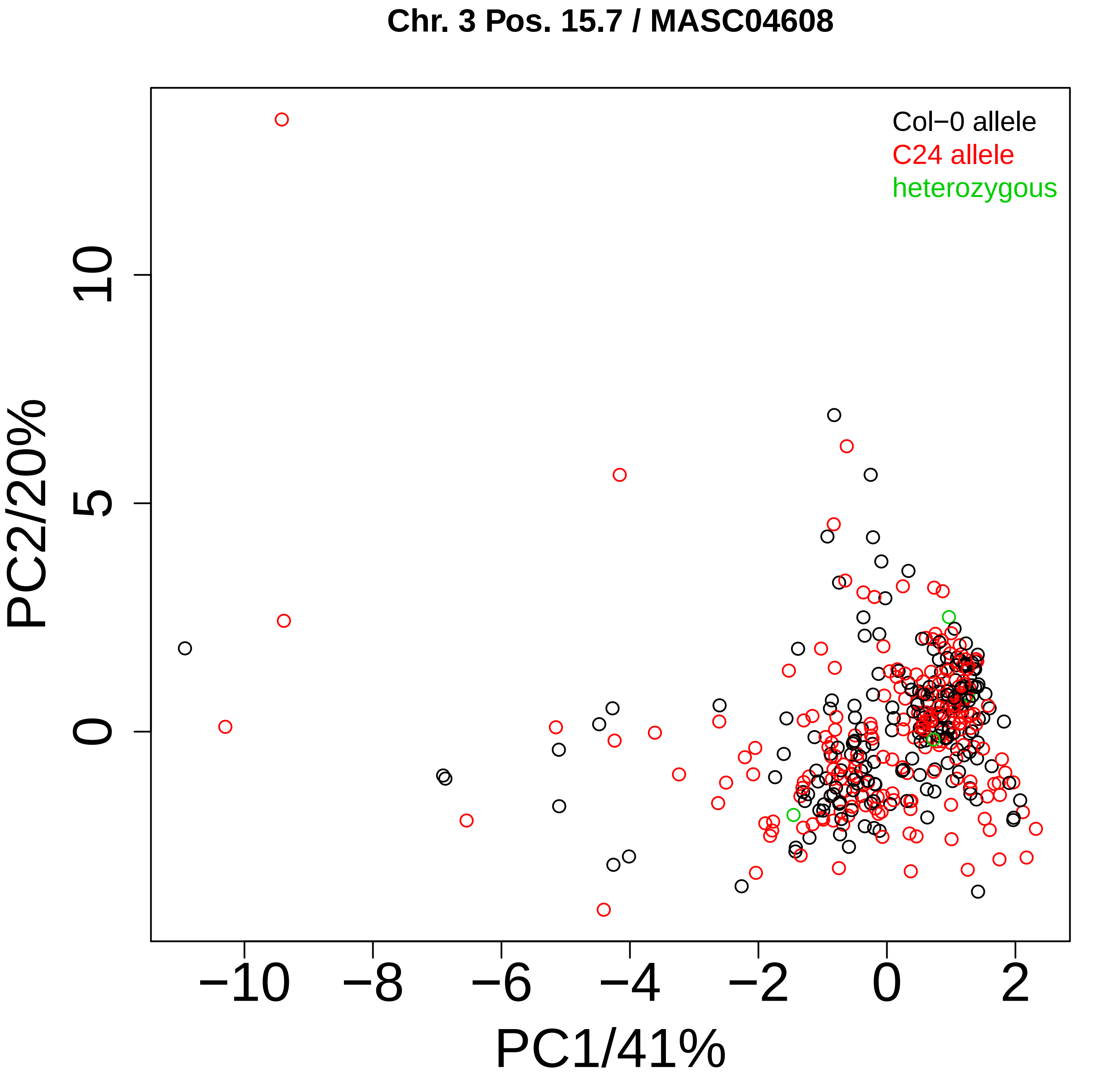
Chr. 3 Pos. 15.7 / MASC04608
Col−0 allele
C24 allele
heterozygous
PC2/20%
PC1/41%
10
5
0
−10
−8
−6
−4
−2
0
2

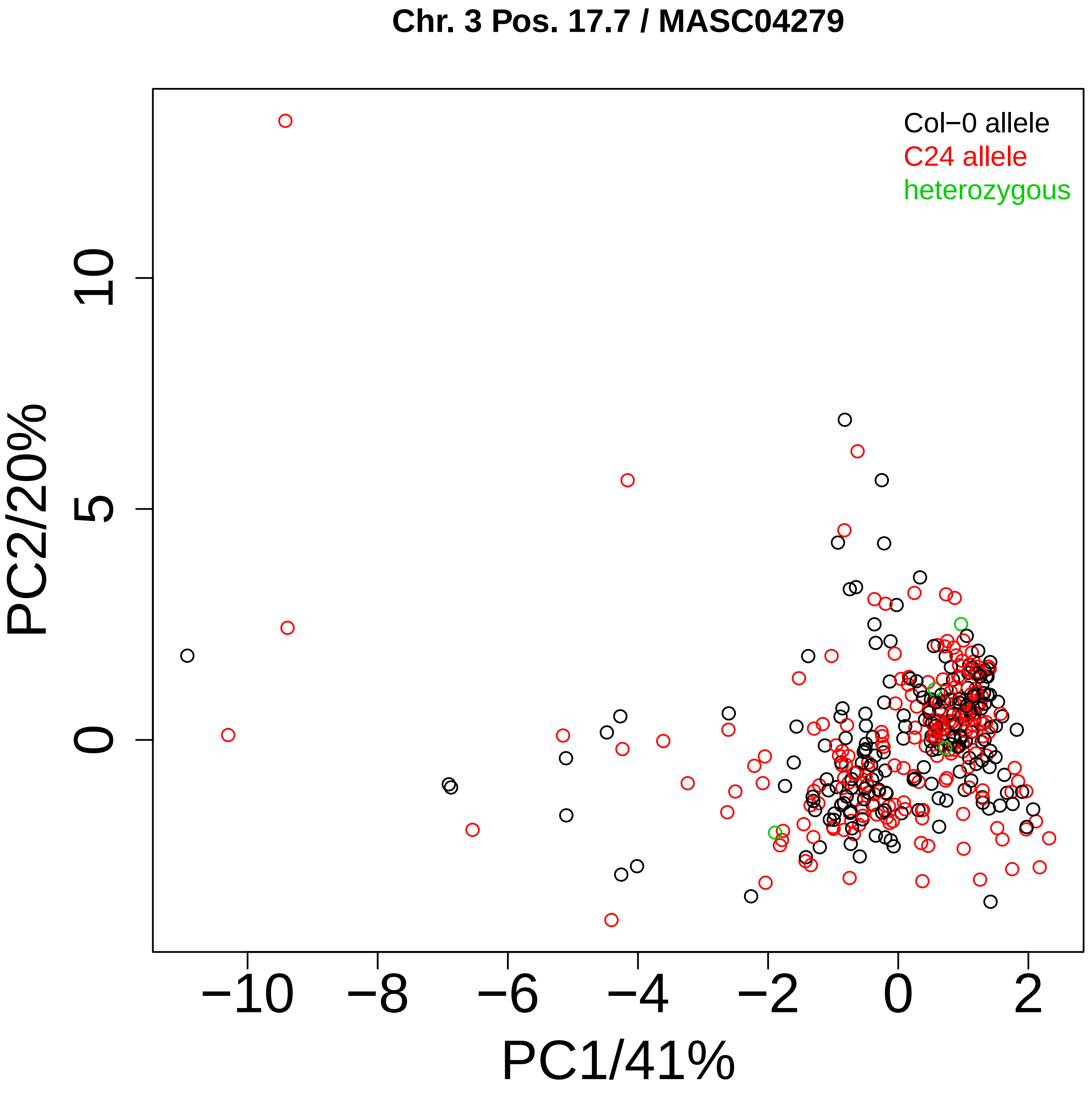
Chr. 3 Pos. 17.7 / MASC04279
Col−0 allele
C24 allele
heterozygous
PC2/20%
PC1/41%
10
5
0
−10
−8
−6
−4
−2
0
2

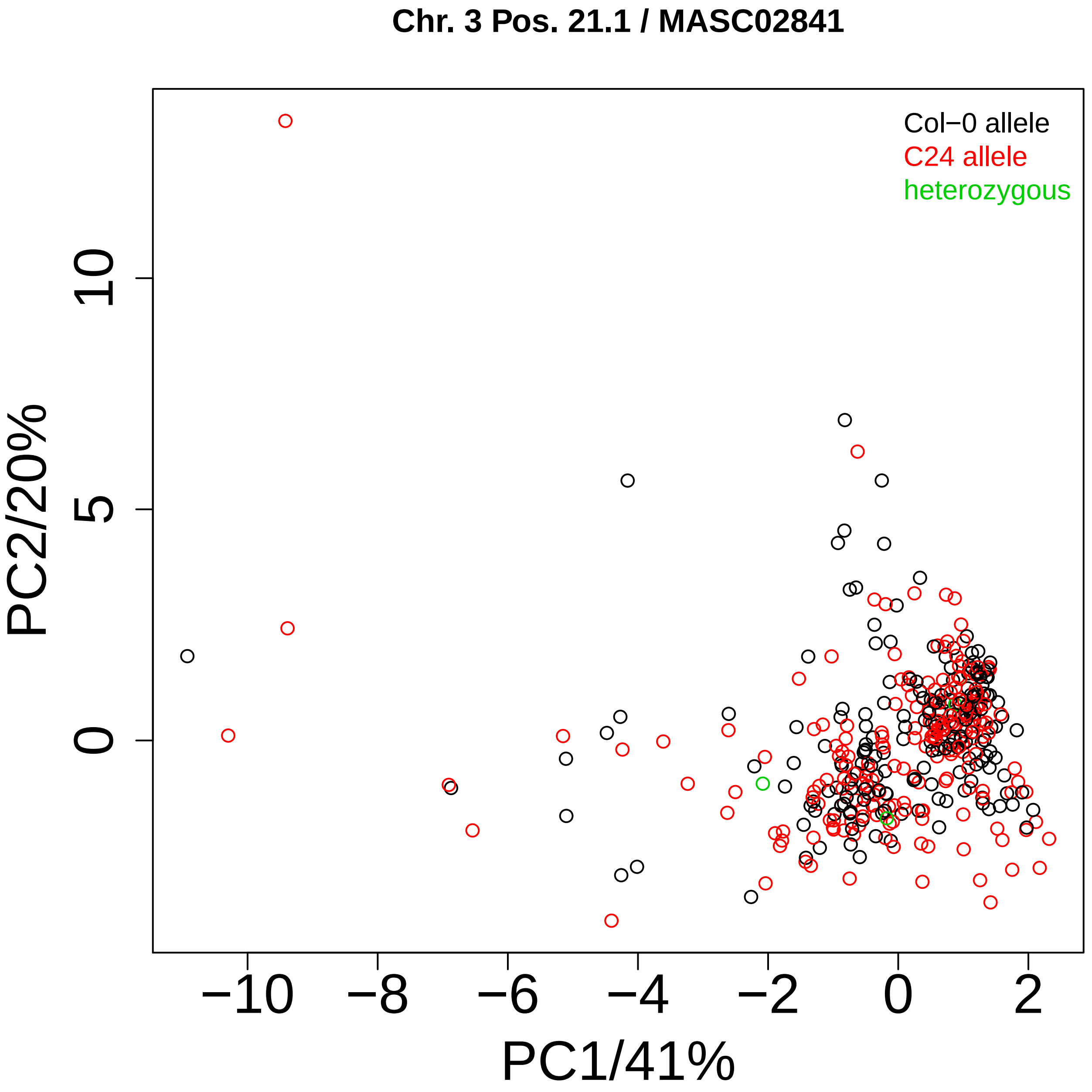
Chr. 3 Pos. 21.1 / MASC02841
Col−0 allele
C24 allele
heterozygous
PC2/20%
0
5
10
PC1/41%
−10
−8
−6
−4
−2
0
2

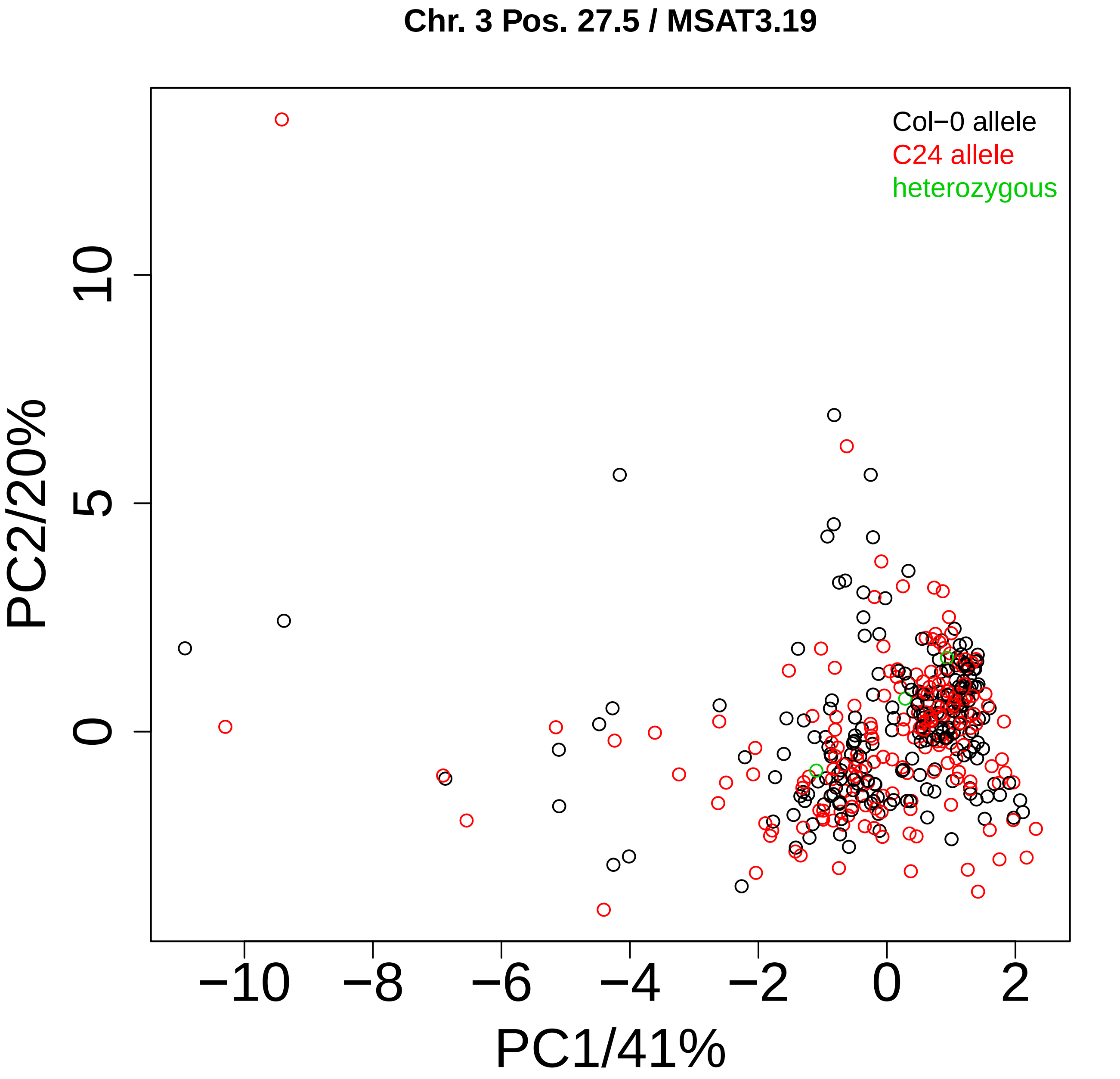

Chr. 3 Pos. 27.5 / MSAT3.19
Col−0 allele
C24 allele
heterozygous
PC2/20%
0
5
10
PC1/41%
−10
−8
−6
−4
−2
0
2

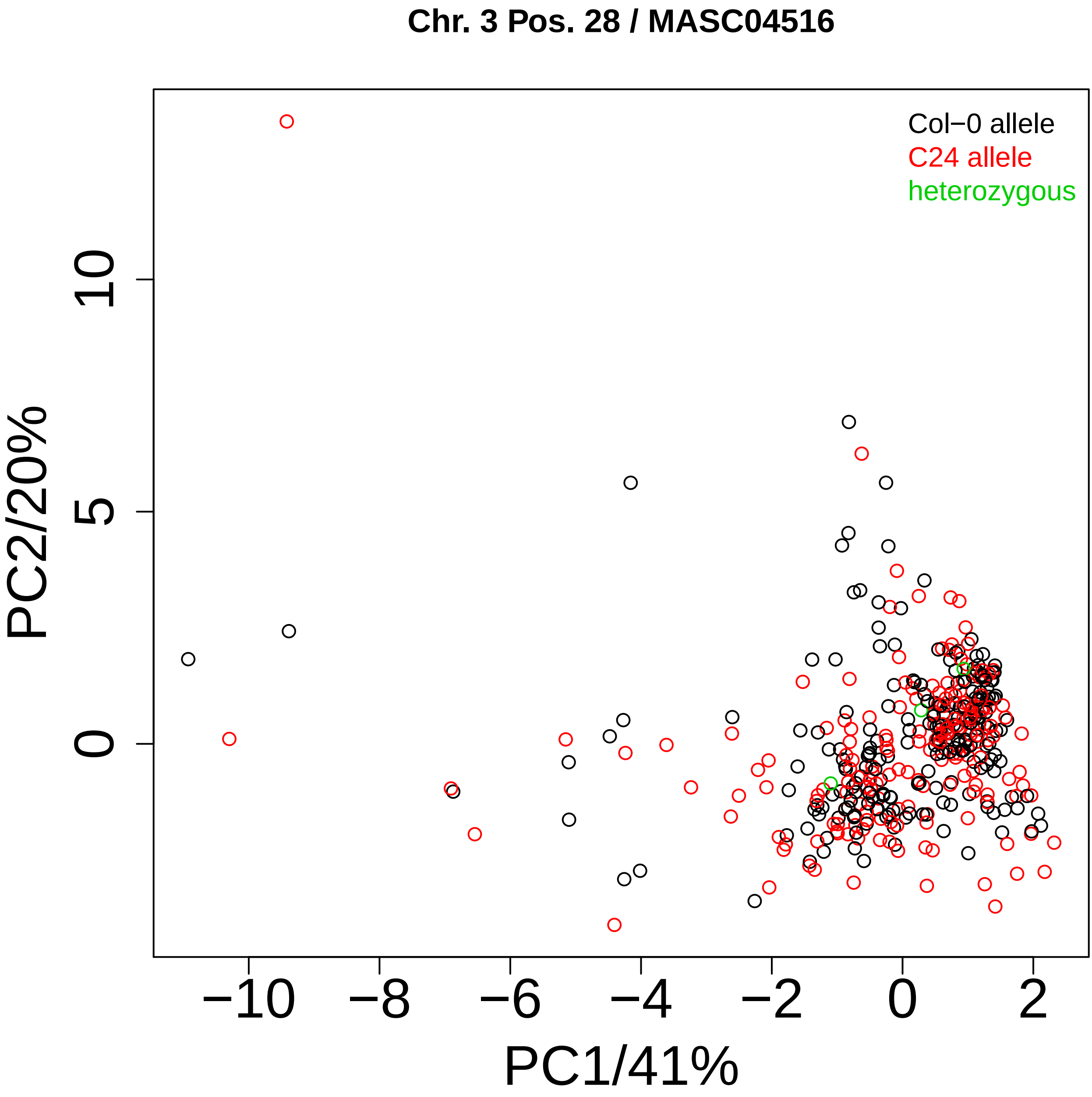
Chr. 3 Pos. 28 / MASC04516
Col−0 allele
C24 allele
heterozygous
PC2/20%
0
5
10
PC1/41%
−10
−8
−6
−4
−2
0
2

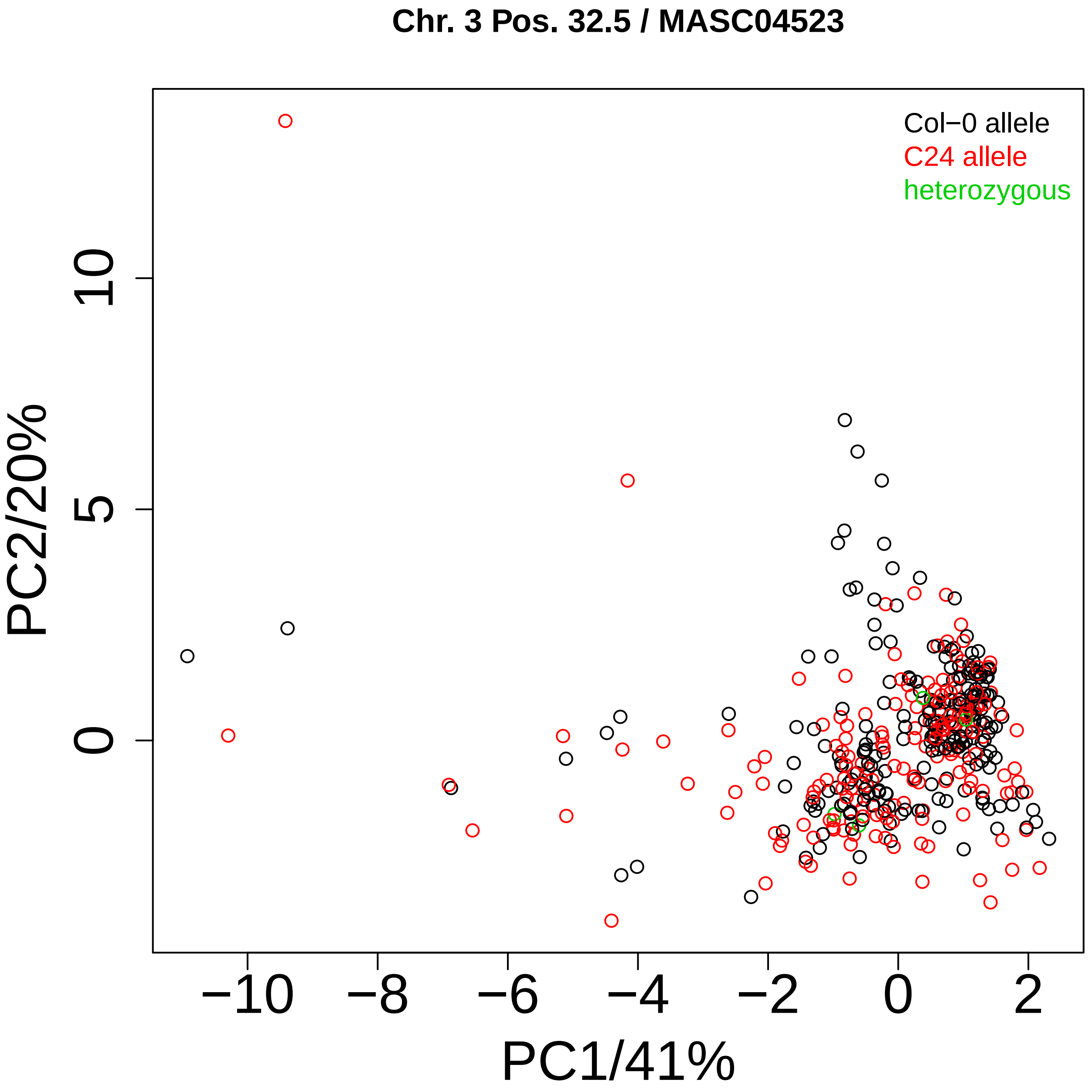

Chr. 3 Pos. 32.5 / MASC04523
Col−0 allele
C24 allele
heterozygous
PC1/41%
PC2/20%
−10
−8
−6
−4
−2
0
2
0
5
10

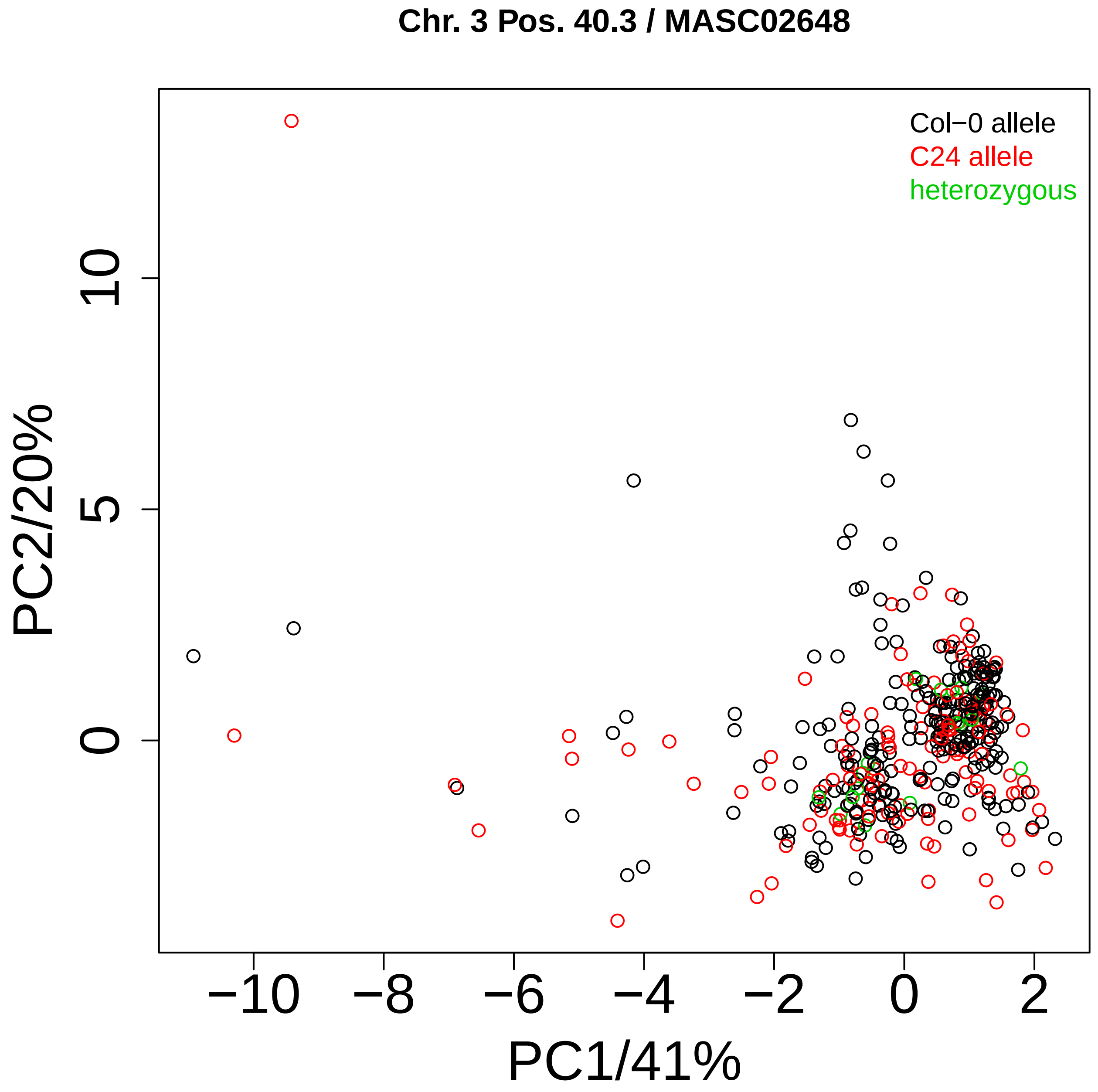

Chr. 3 Pos. 40.3 / MASC02648
Col−0 allele
C24 allele
heterozygous
PC2/20%
0
5
10
PC1/41%
−10
−8
−6
−4
−2
0
2

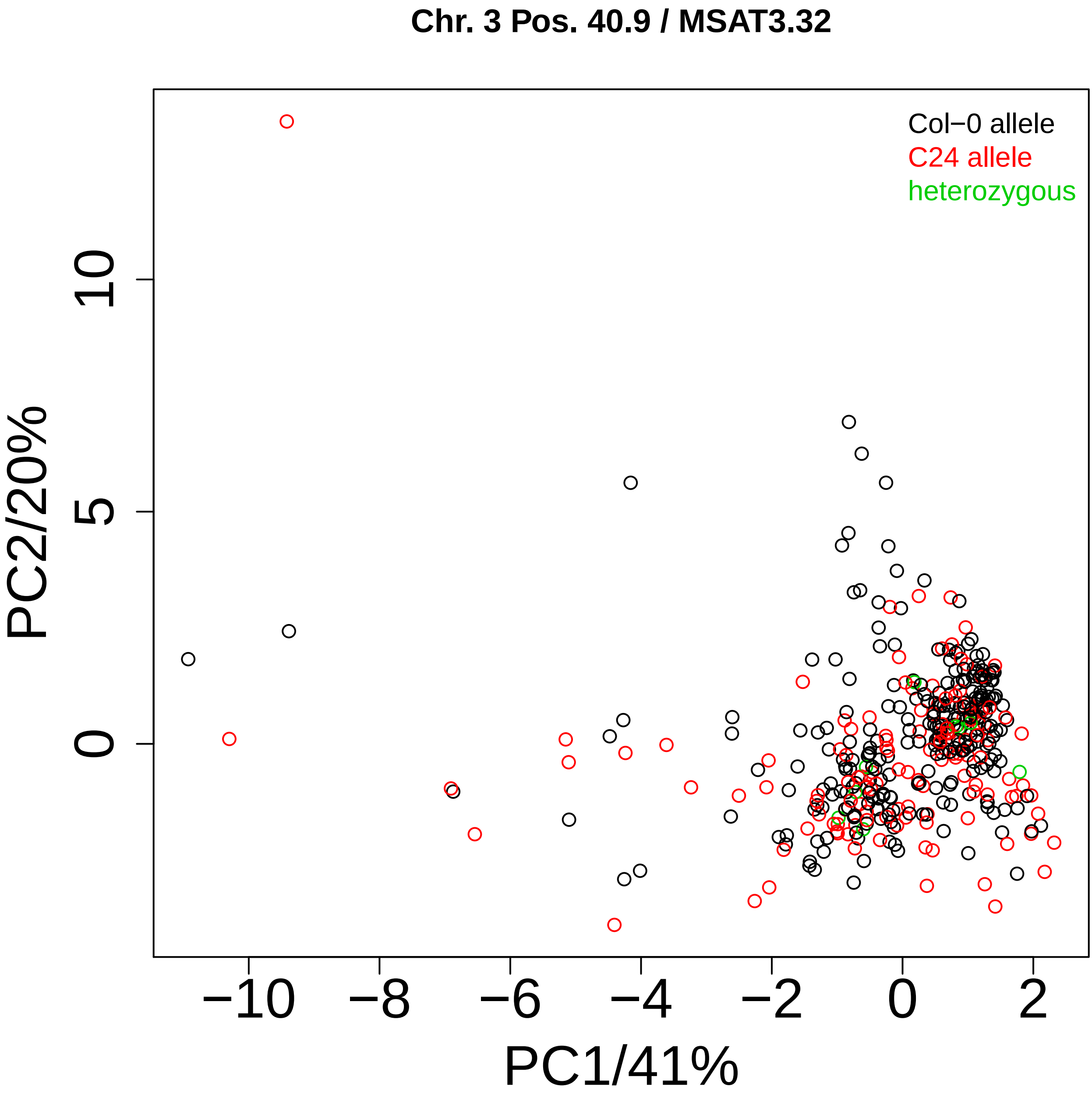
Chr. 3 Pos. 40.9 / MSAT3.32
Col−0 allele
C24 allele
heterozygous
PC2/20%
0
5
10
PC1/41%
−10
−8
−6
−4
−2
0
2

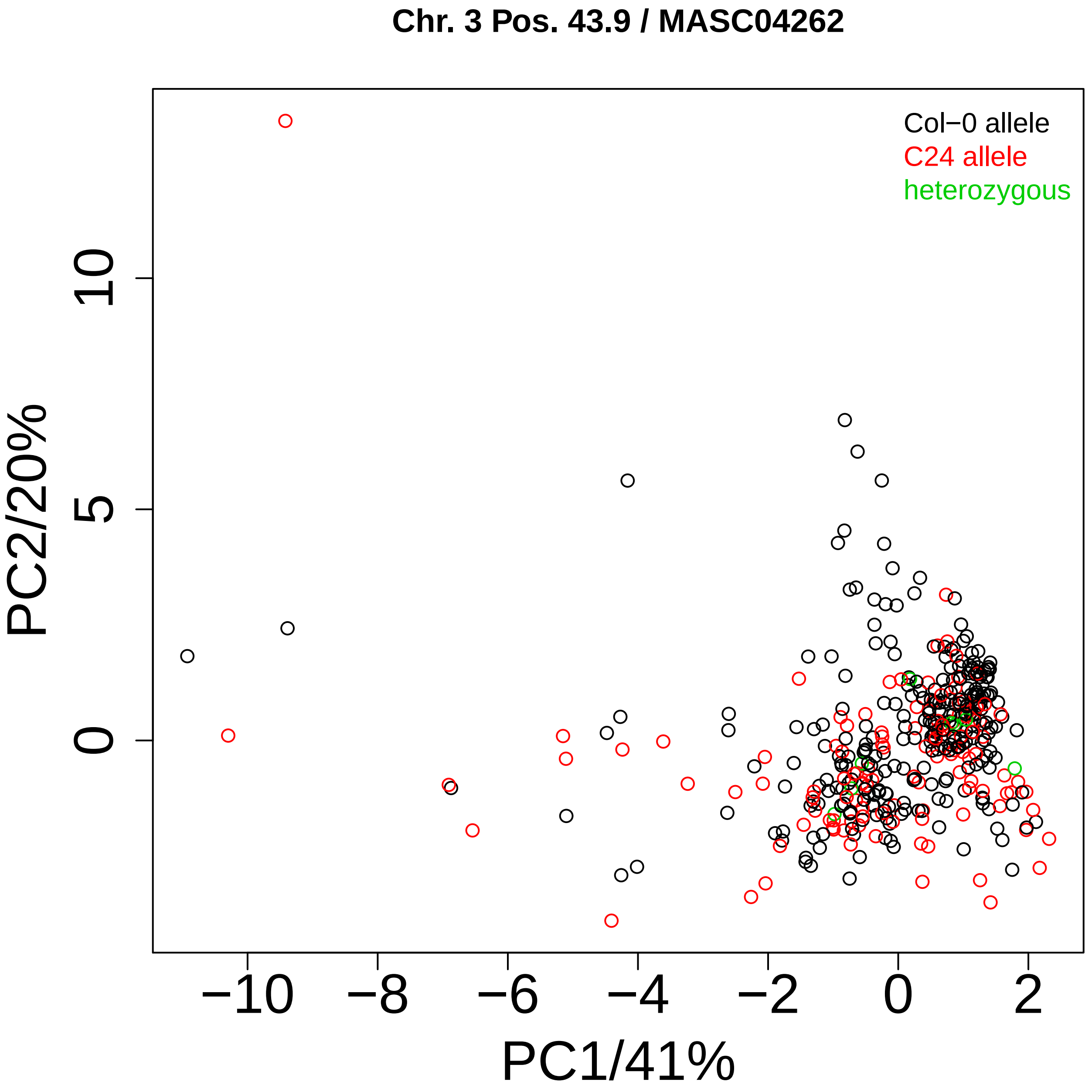
Chr. 3 Pos. 43.9 / MASC04262
Col−0 allele
C24 allele
heterozygous
PC2/20%
PC1/41%
10
5
0
−10
−8
−6
−4
−2
0
2

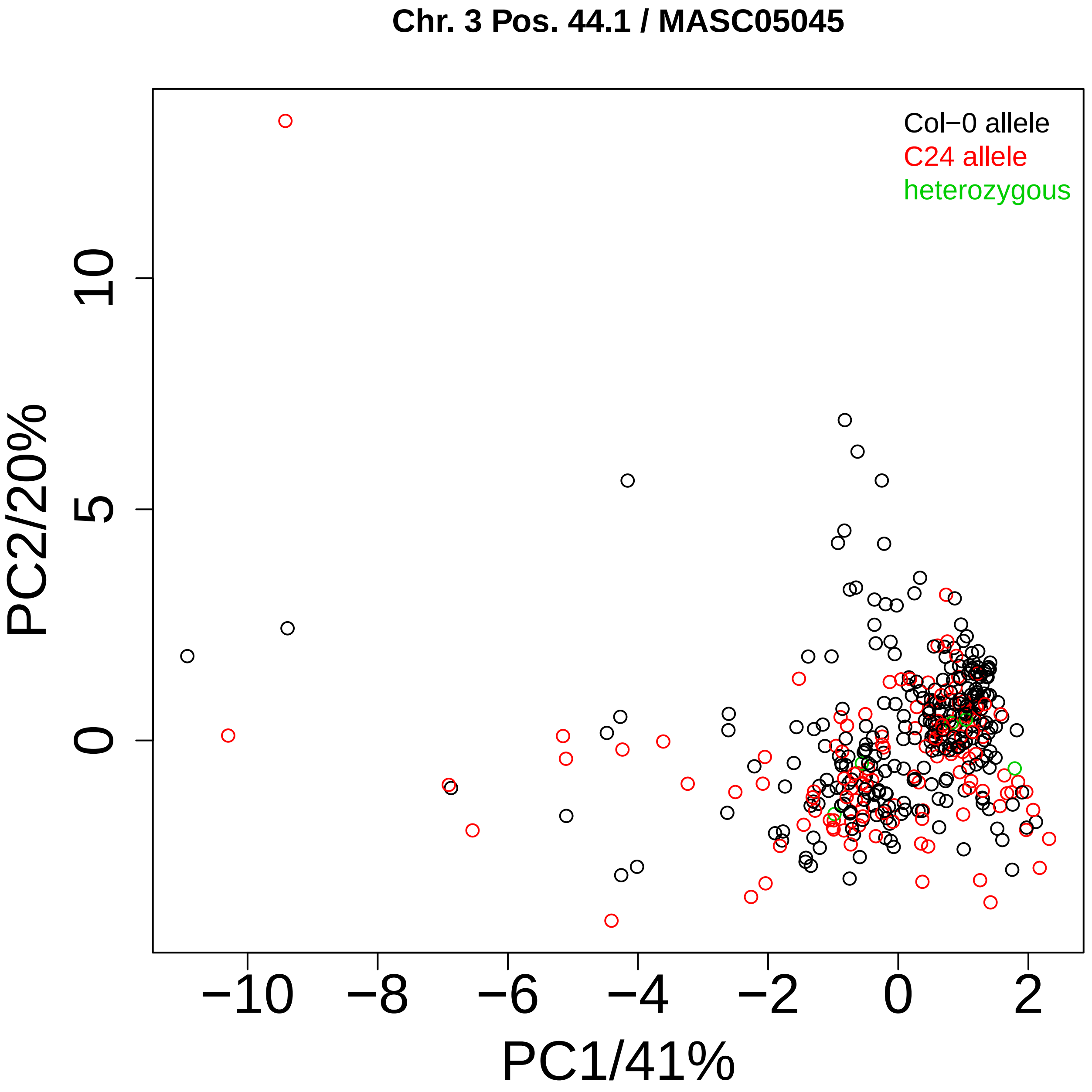
Chr. 3 Pos. 44.1 / MASC05045
Col−0 allele
C24 allele
heterozygous
PC2/20%
PC1/41%
0
5
10
−10
−8
−6
−4
−2
0
2

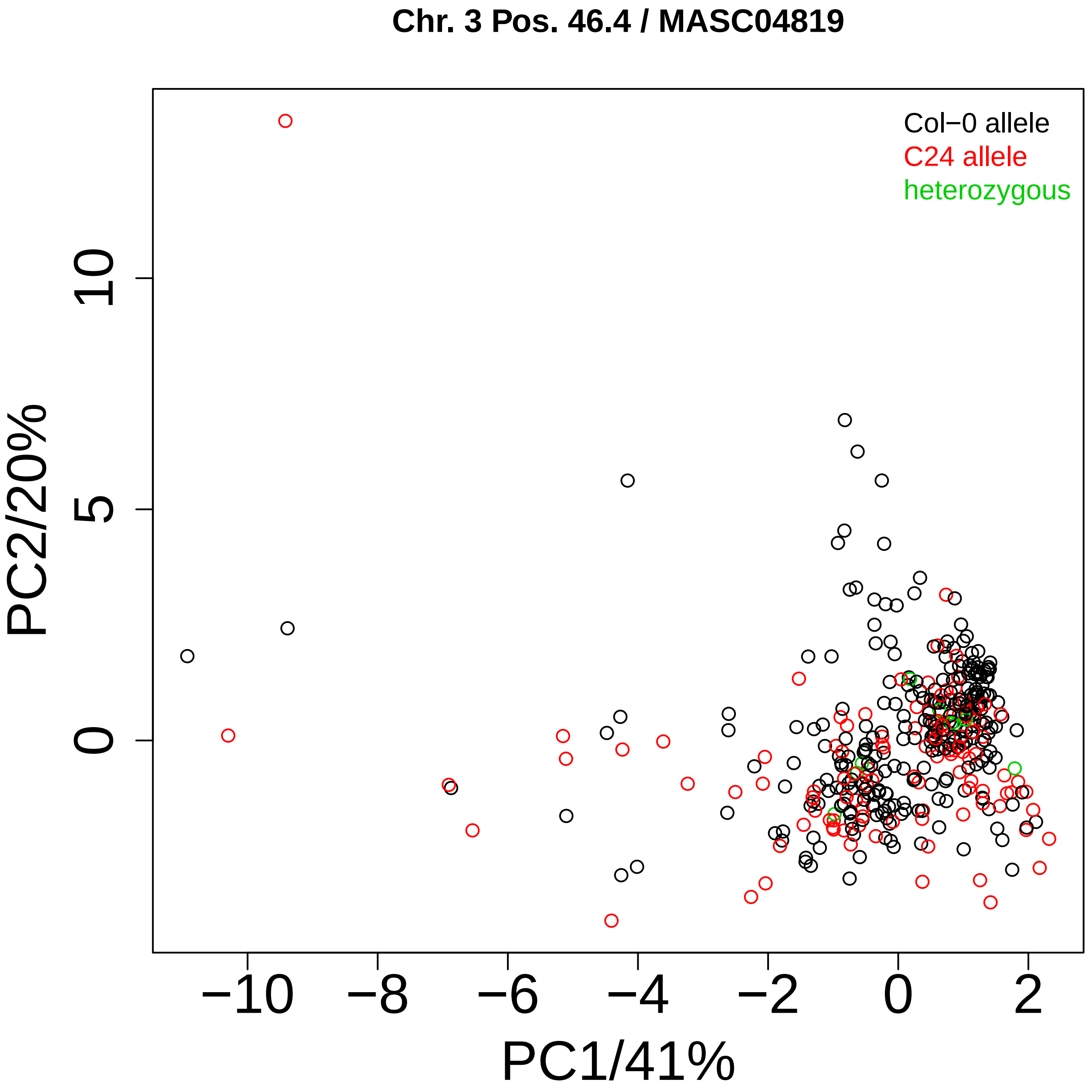

Chr. 3 Pos. 46.4 / MASC04819
Col−0 allele
C24 allele
heterozygous
PC2/20%
PC1/41%
10
5
0
−10
−8
−6
−4
−2
0
2

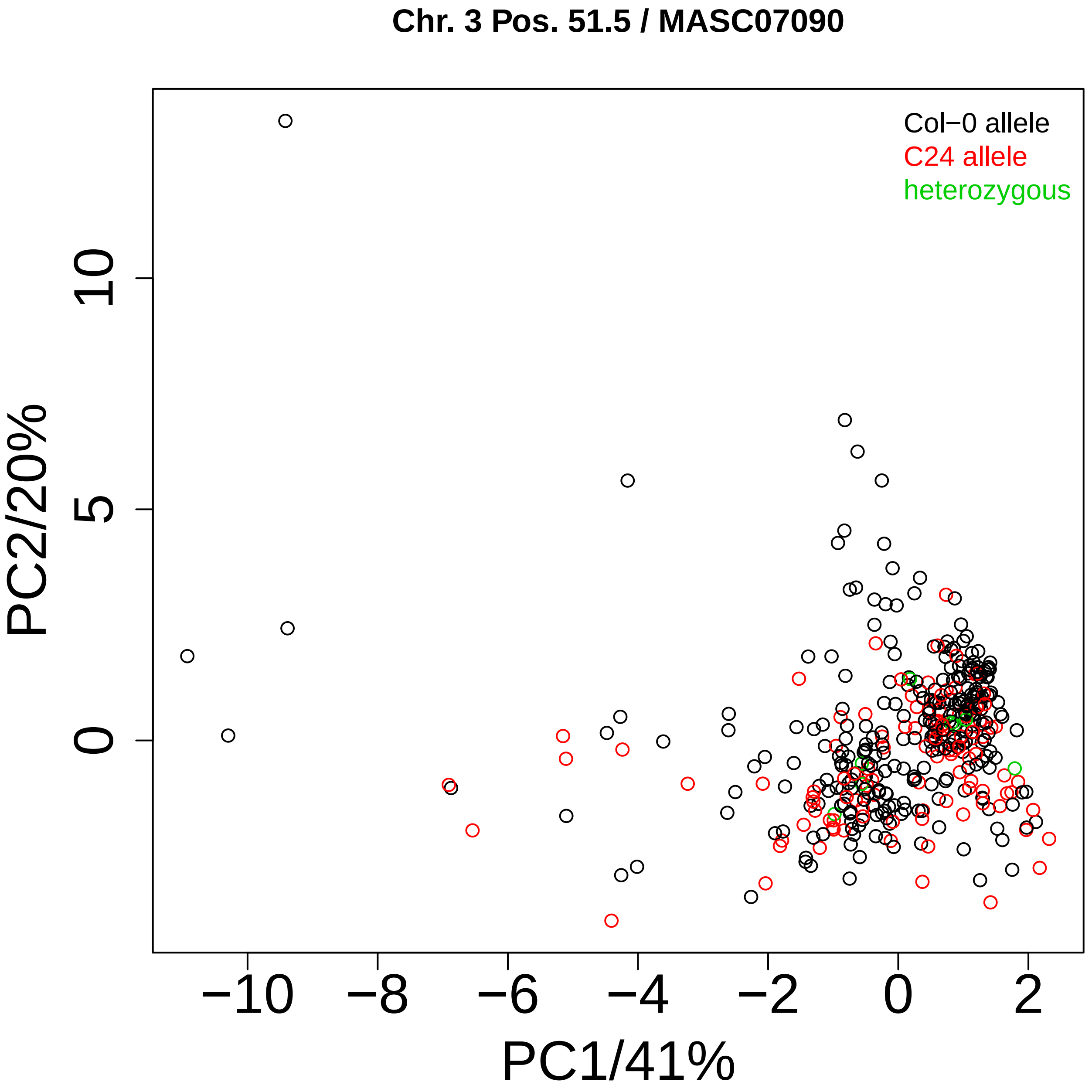
Chr. 3 Pos. 51.5 / MASC07090
Col−0 allele
C24 allele
heterozygous
10
5
0
PC2/20%
−10
−8
−6
−4
−2
0
2
PC1/41%

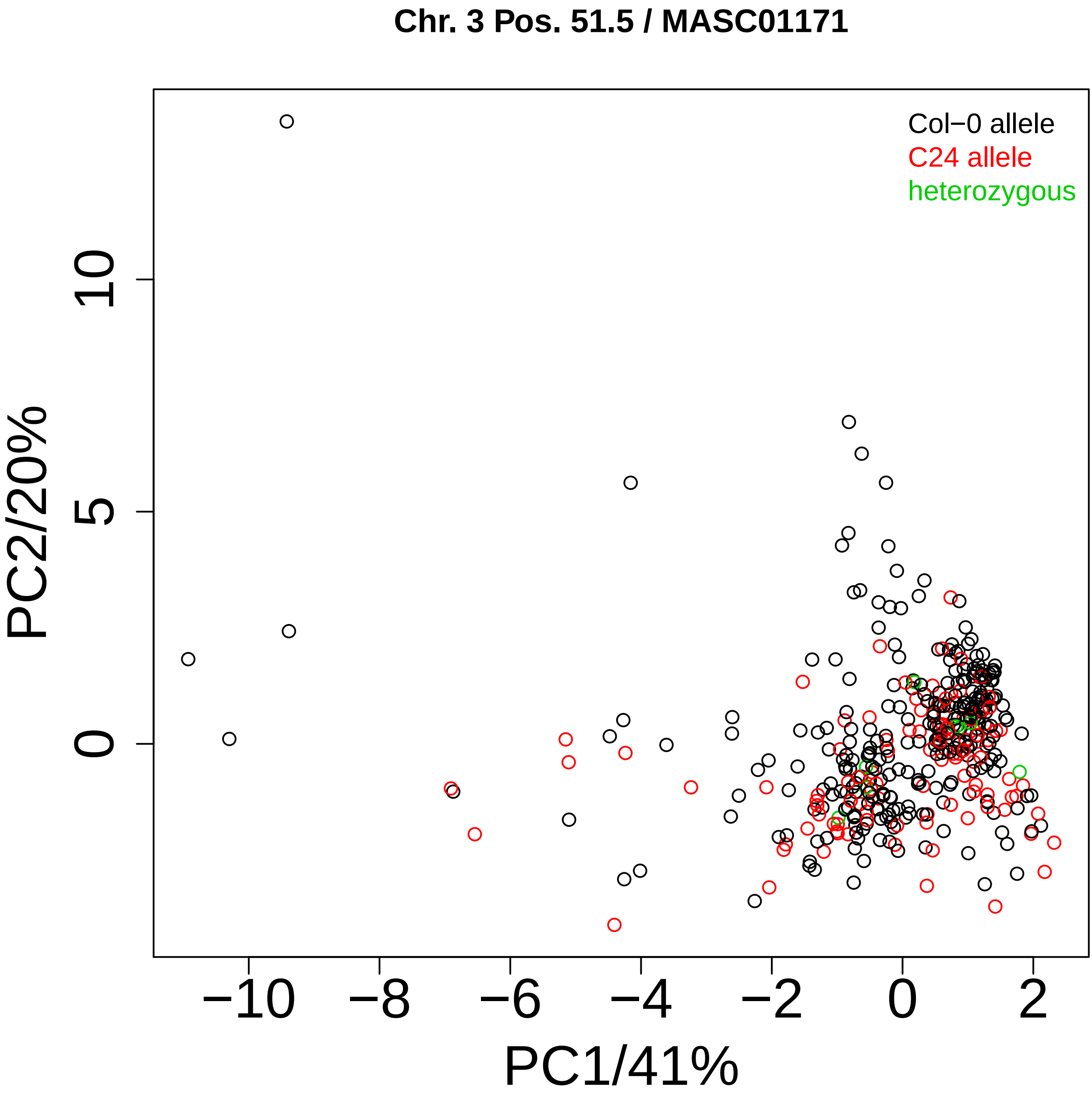

Chr. 3 Pos. 51.5 / MASC01171
Col−0 allele
C24 allele
heterozygous
PC2/20%
PC1/41%
10
5
0
−10
−8
−6
−4
−2
0
2

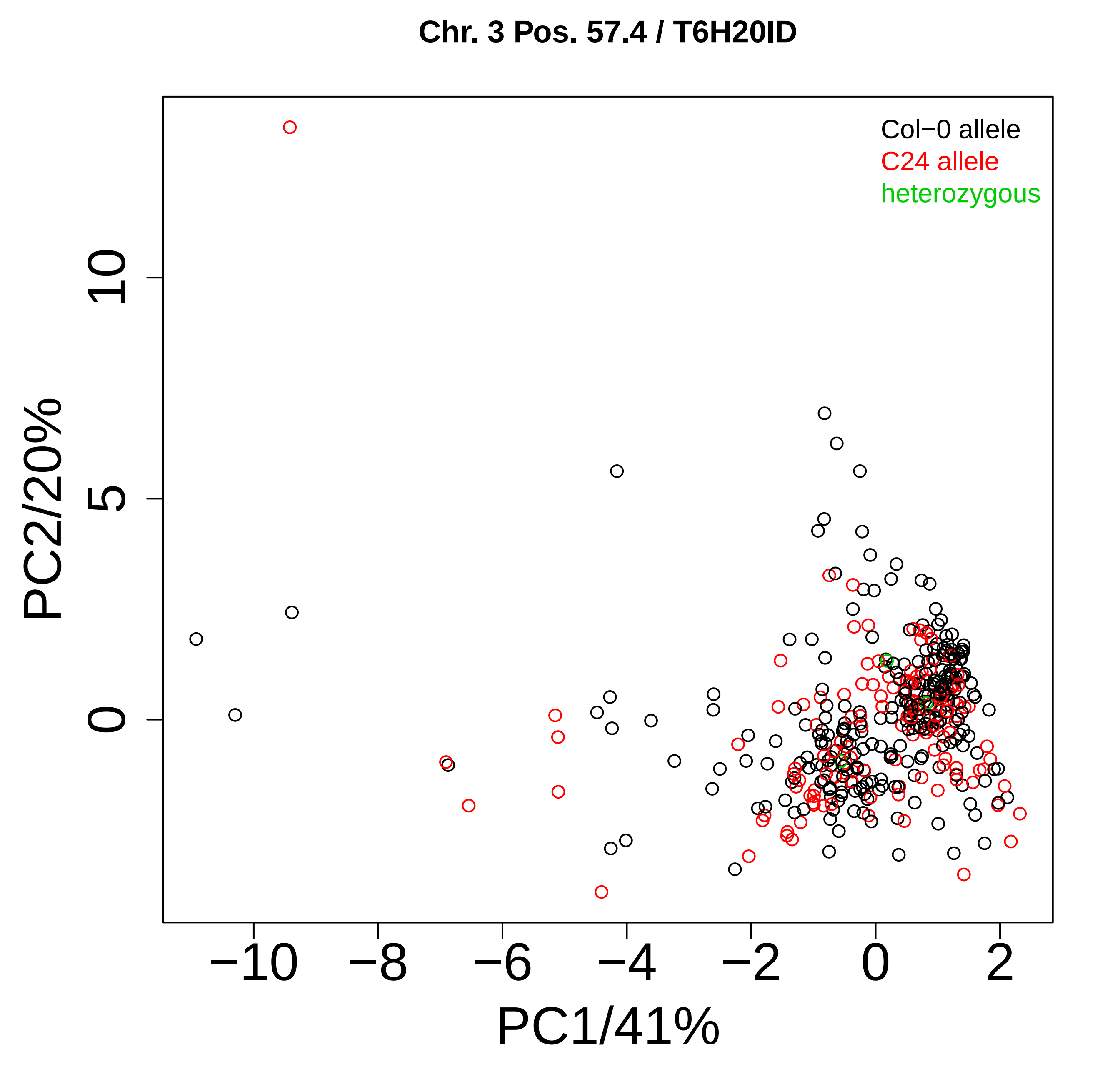
Chr. 3 Pos. 57.4 / T6H20ID
Col−0 allele
C24 allele
heterozygous
PC2/20%
0
5
10
−10
−8
−6
−4
−2
0
2
PC1/41%

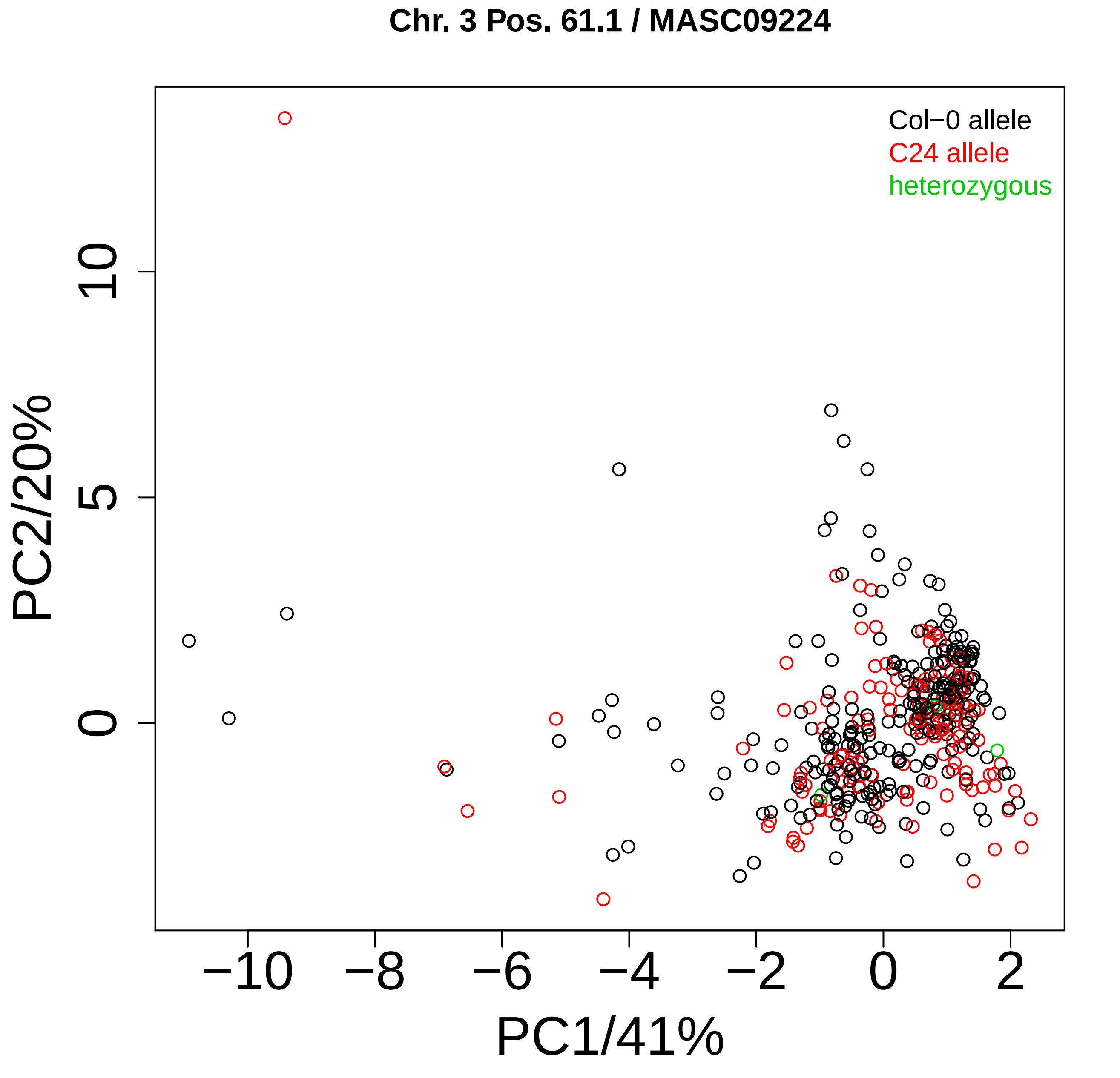
Chr. 3 Pos. 61.1 / MASC09224
Col−0 allele
C24 allele
heterozygous
PC2/20%
0
5
10
PC1/41%
−10
−8
−6
−4
−2
0
2

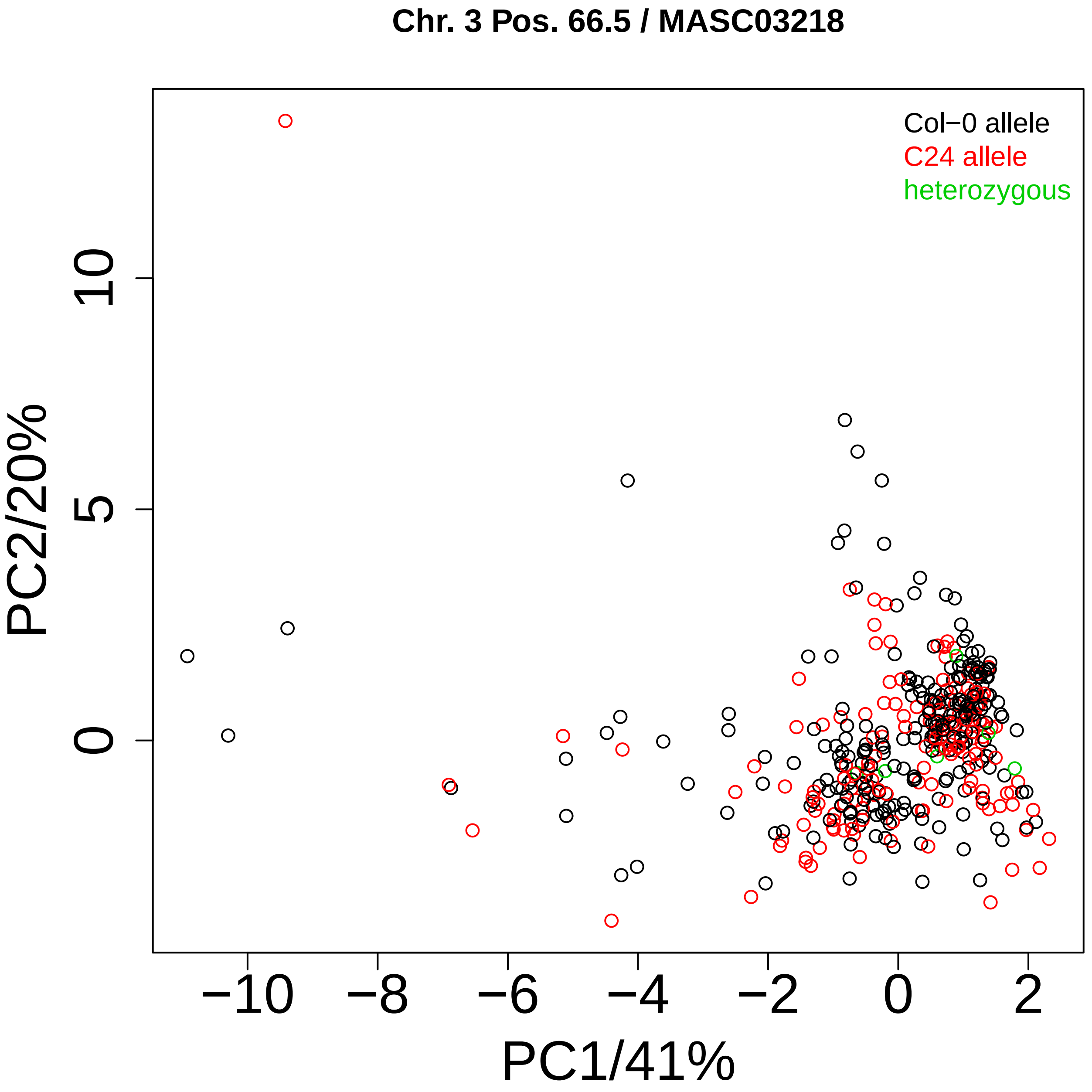

Chr. 3 Pos. 66.5 / MASC03218
Col−0 allele
C24 allele
heterozygous
PC2/20%
PC1/41%
0
5
10
−10
−8
−6
−4
−2
0
2

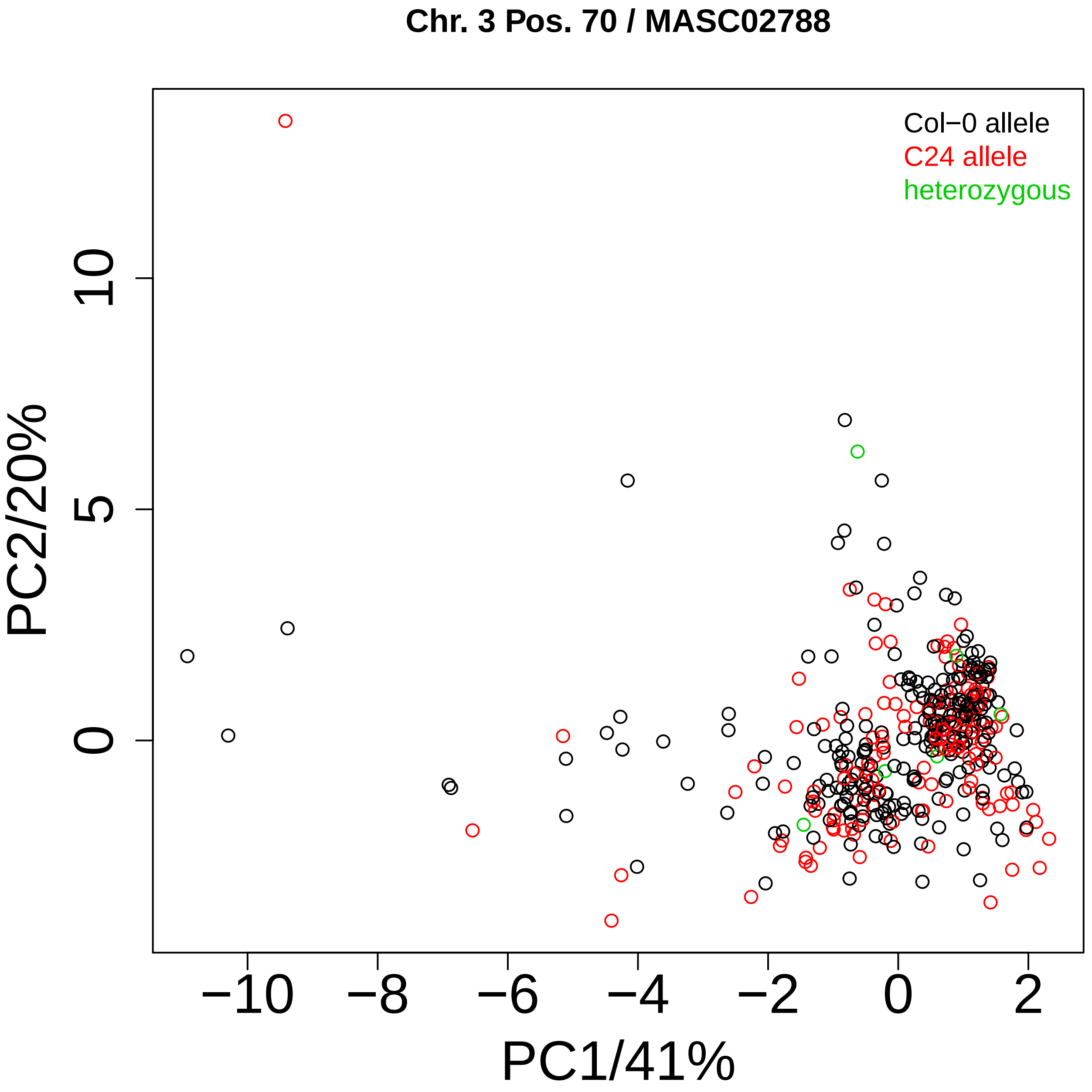
Chr. 3 Pos. 70 / MASC02788
Col−0 allele
C24 allele
heterozygous
PC2/20%
10
5
0
PC1/41%
−10
−8
−6
−4
−2
0
2

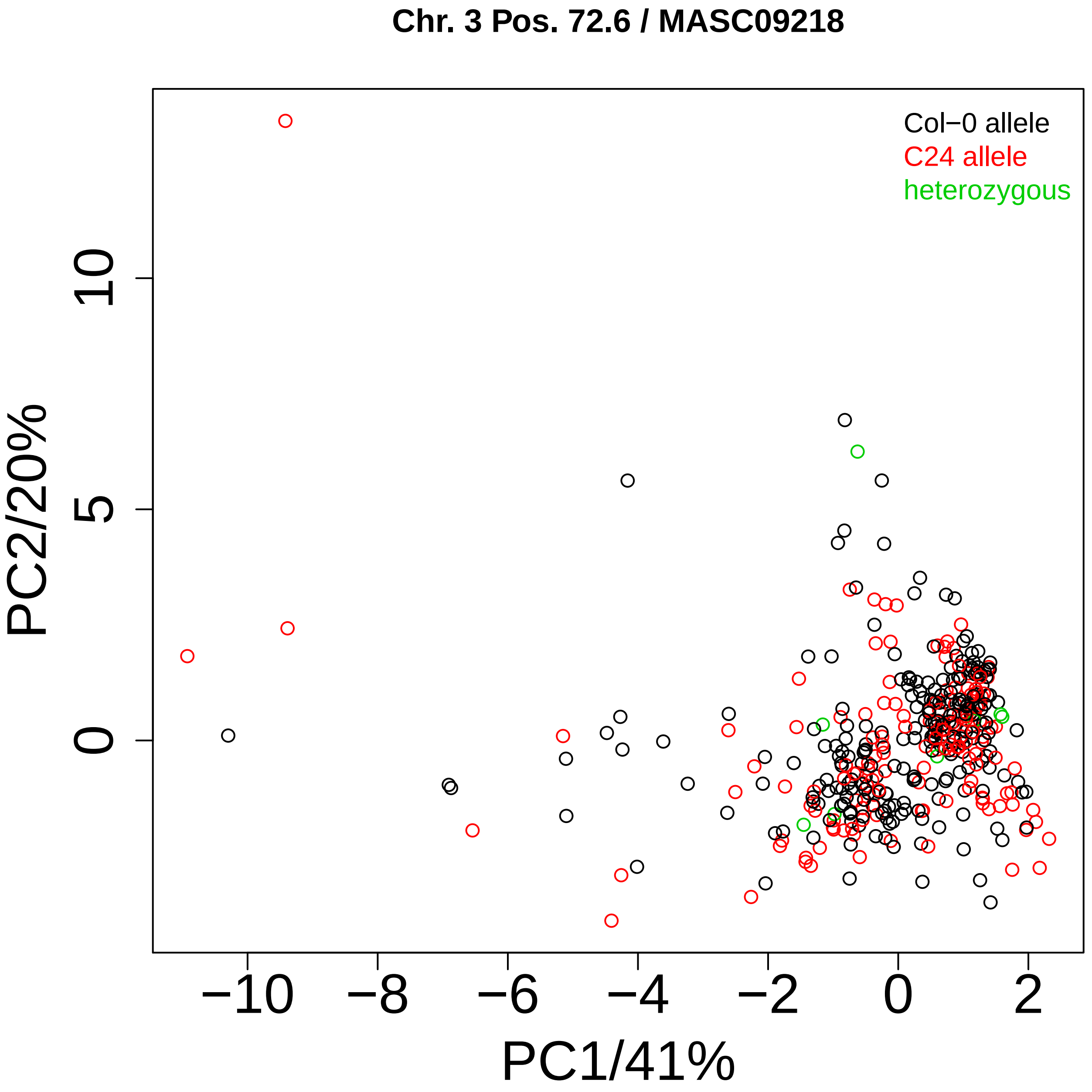
Chr. 3 Pos. 72.6 / MASC09218
Col−0 allele
C24 allele
heterozygous
PC2/20%
0
5
10
PC1/41%
−10
−8
−6
−4
−2
0
2

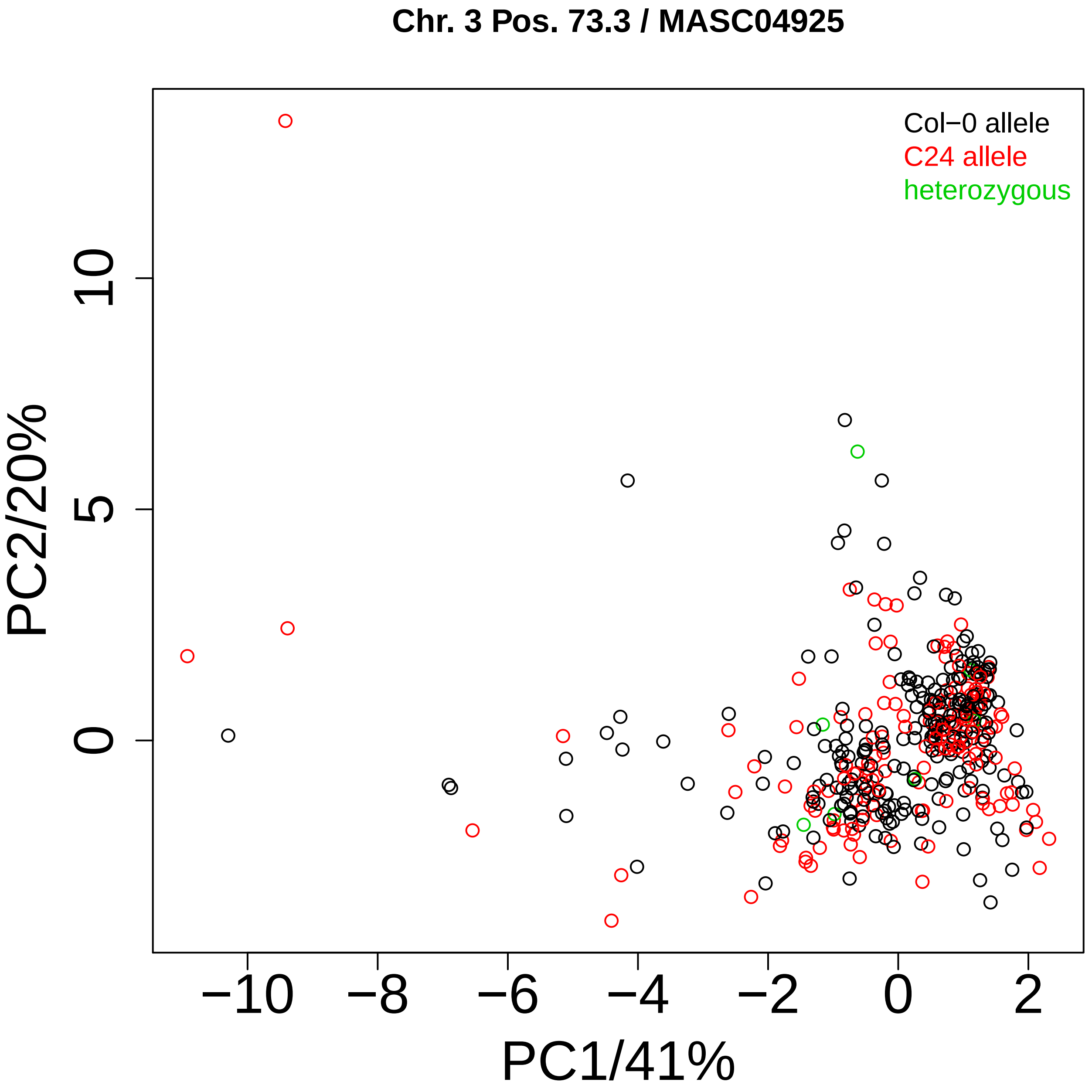

Chr. 3 Pos. 73.3 / MASC04925
Col−0 allele
C24 allele
heterozygous
PC2/20%
0
5
10
PC1/41%
−10
−8
−6
−4
−2
0
2

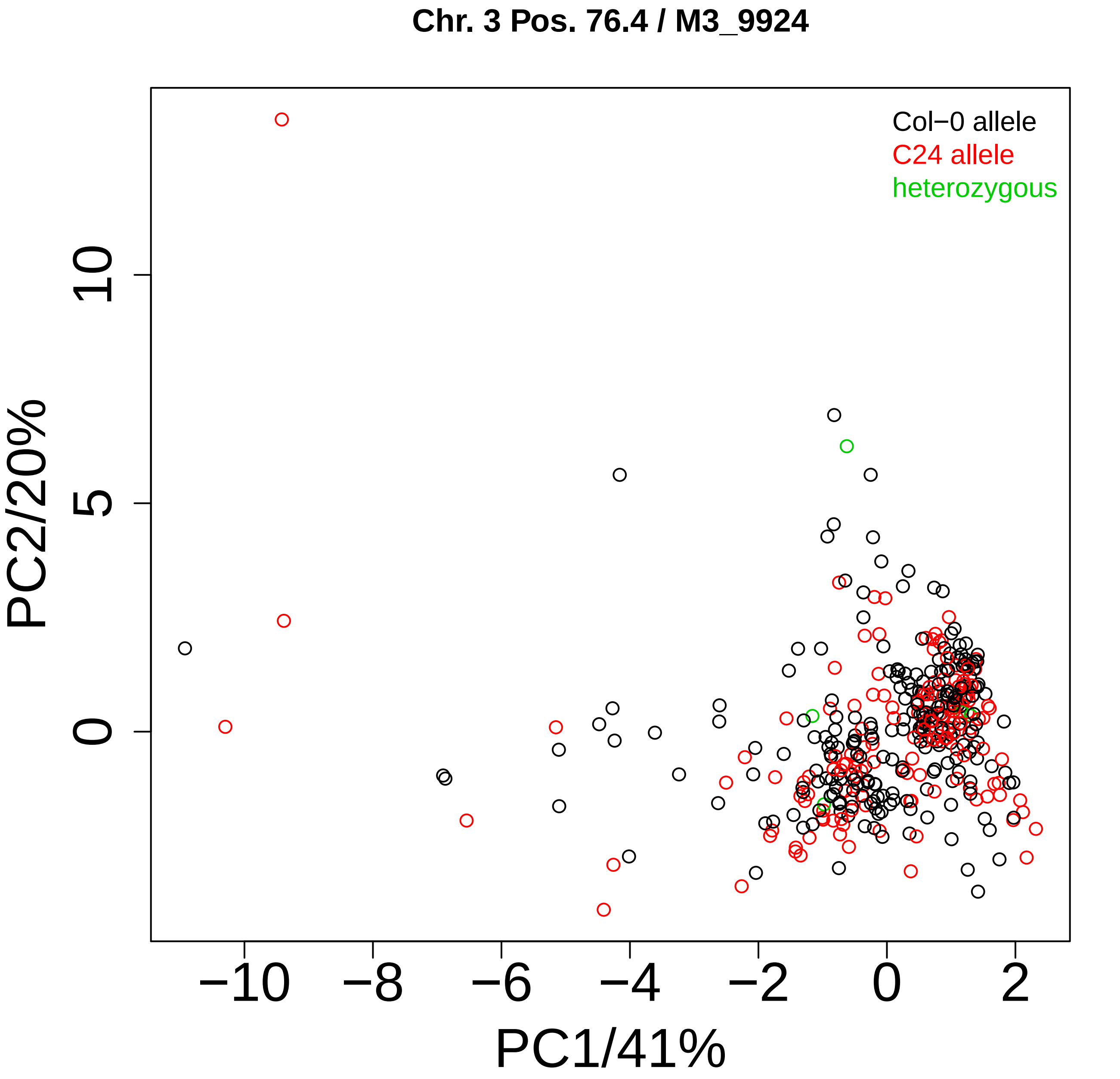
Chr. 3 Pos. 76.4 / M3_9924
Col−0 allele
C24 allele
heterozygous
PC2/20%
0
5
10
PC1/41%
−10
−8
−6
−4
−2
0
2

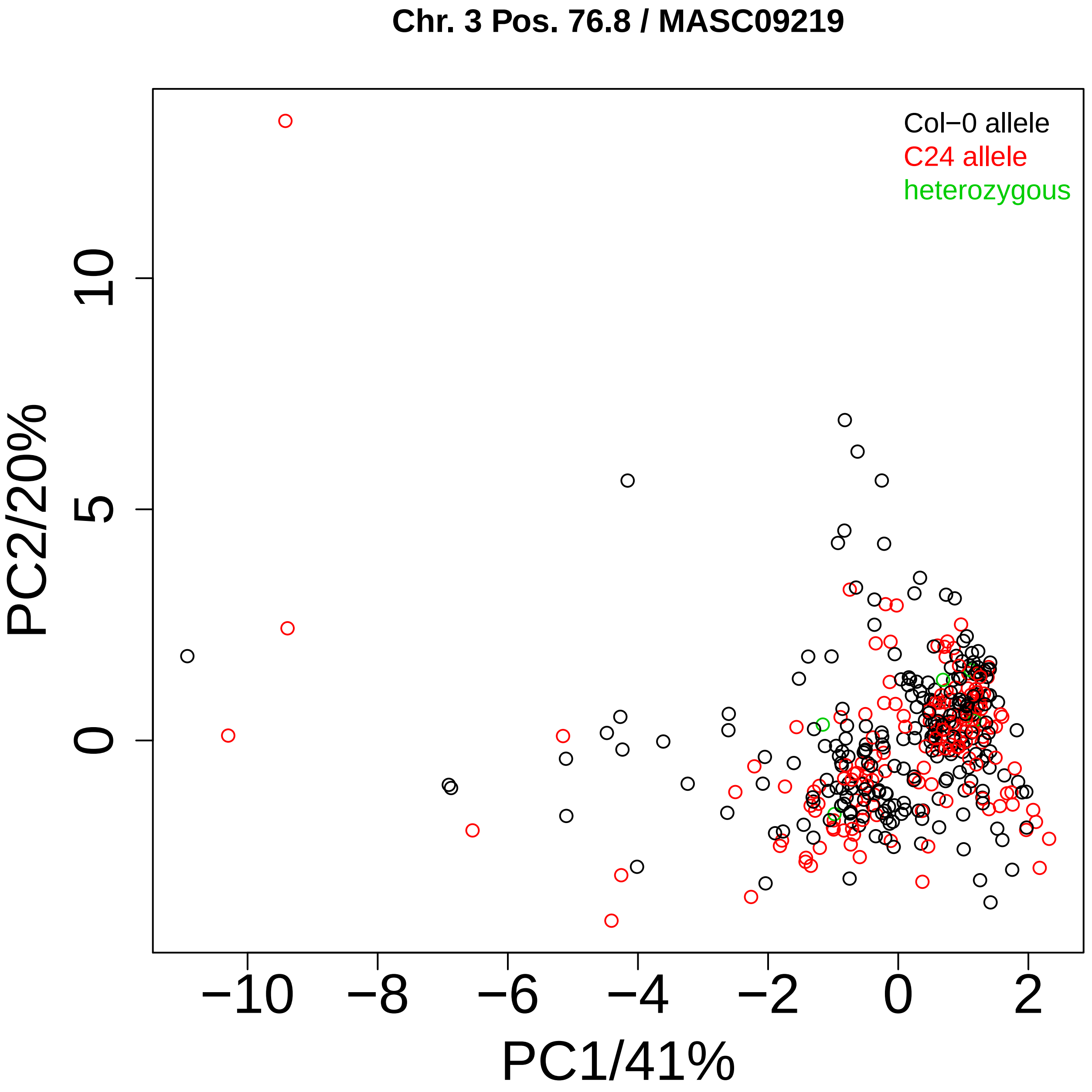

Chr. 3 Pos. 76.8 / MASC09219
Col−0 allele
C24 allele
heterozygous
PC2/20%
PC1/41%
10
5
0
−10
−8
−6
−4
−2
0
2

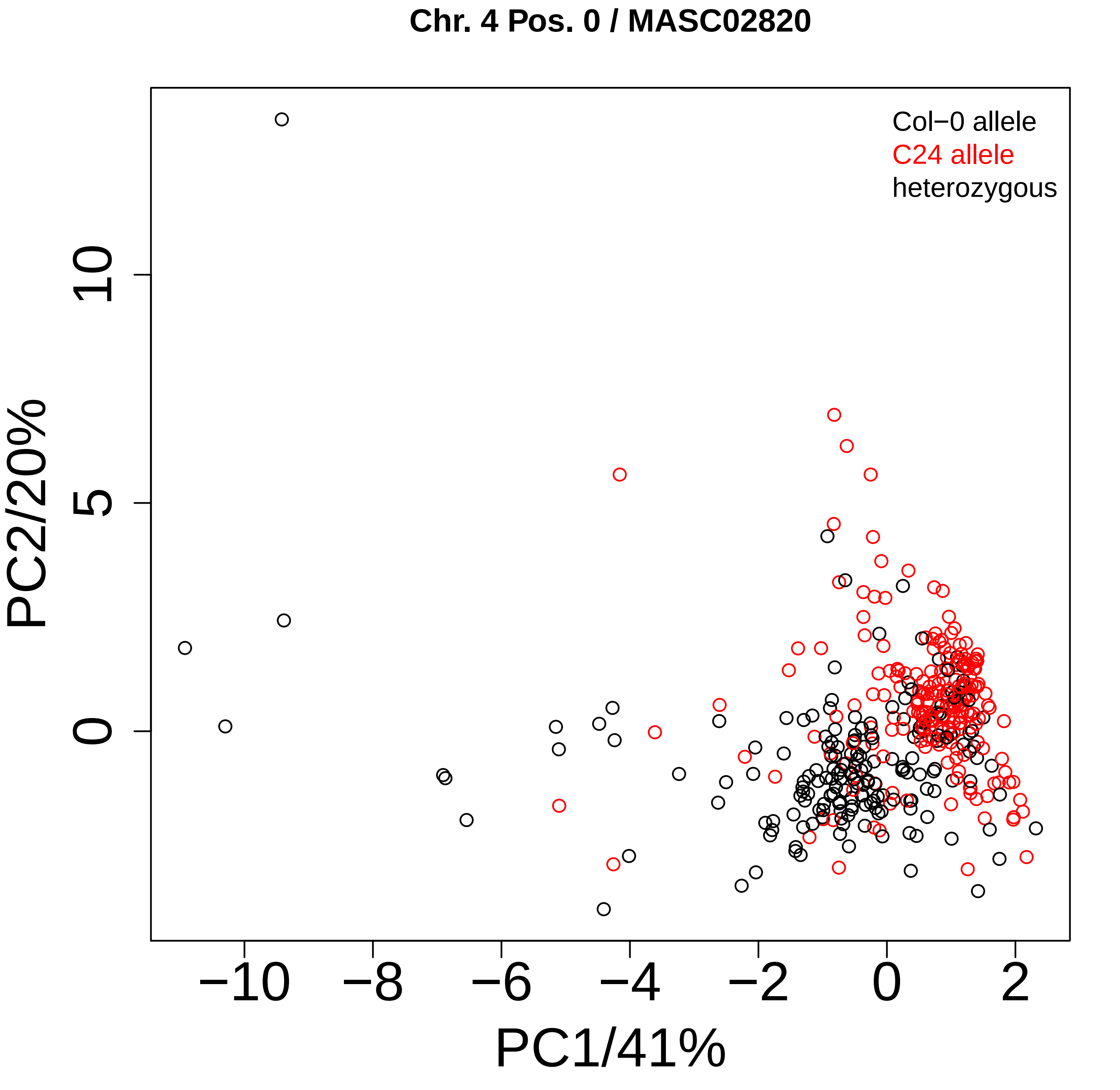
Chr. 4 Pos. 0 / MASC02820
Col−0 allele
C24 allele
heterozygous
PC2/20%
PC1/41%
0
5
10
−10
−8
−6
−4
−2
0
2

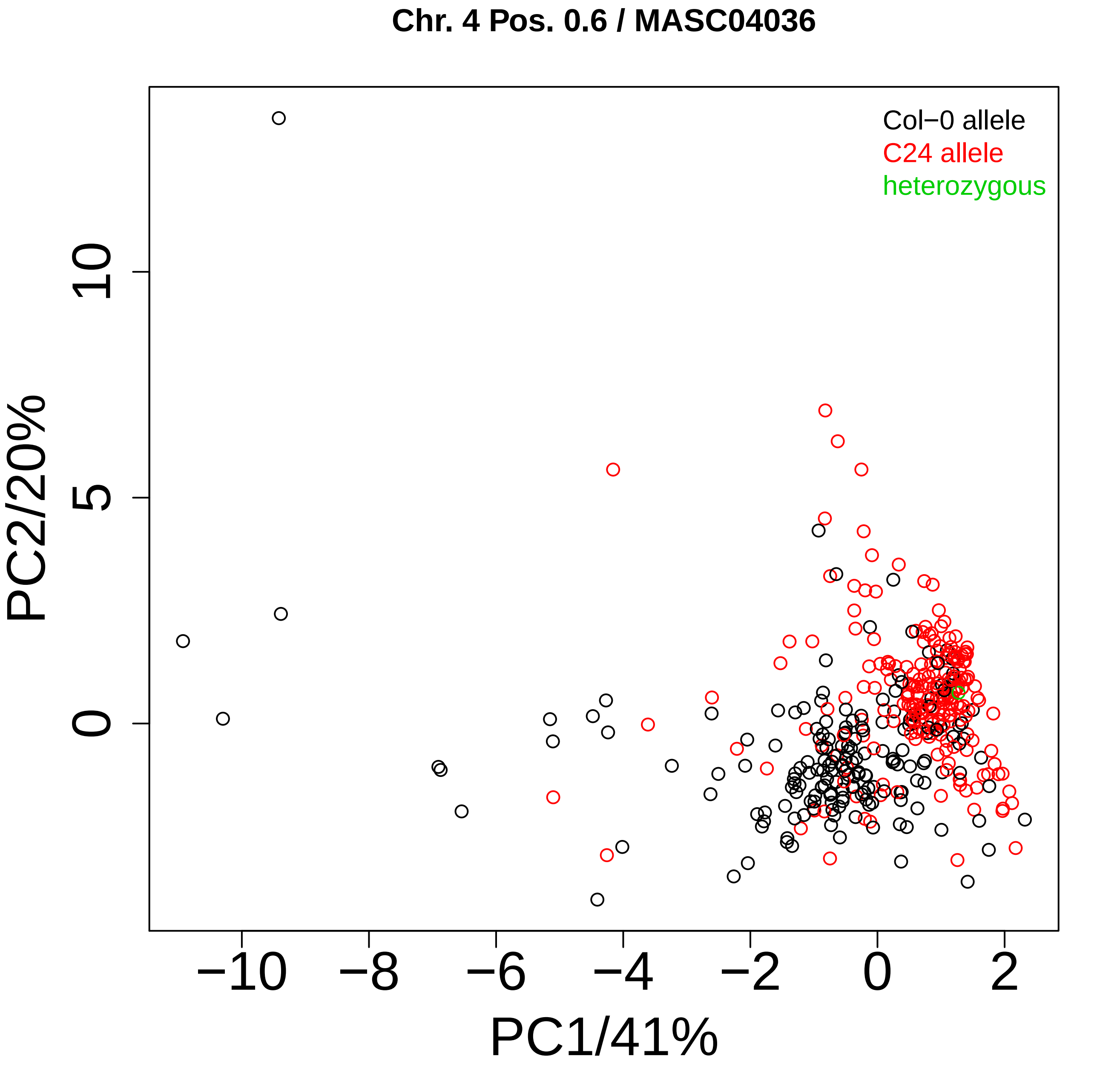

Chr. 4 Pos. 0.6 / MASC04036
Col−0 allele
C24 allele
heterozygous
PC2/20%
PC1/41%
0
5
10
−10
−8
−6
−4
−2
0
2

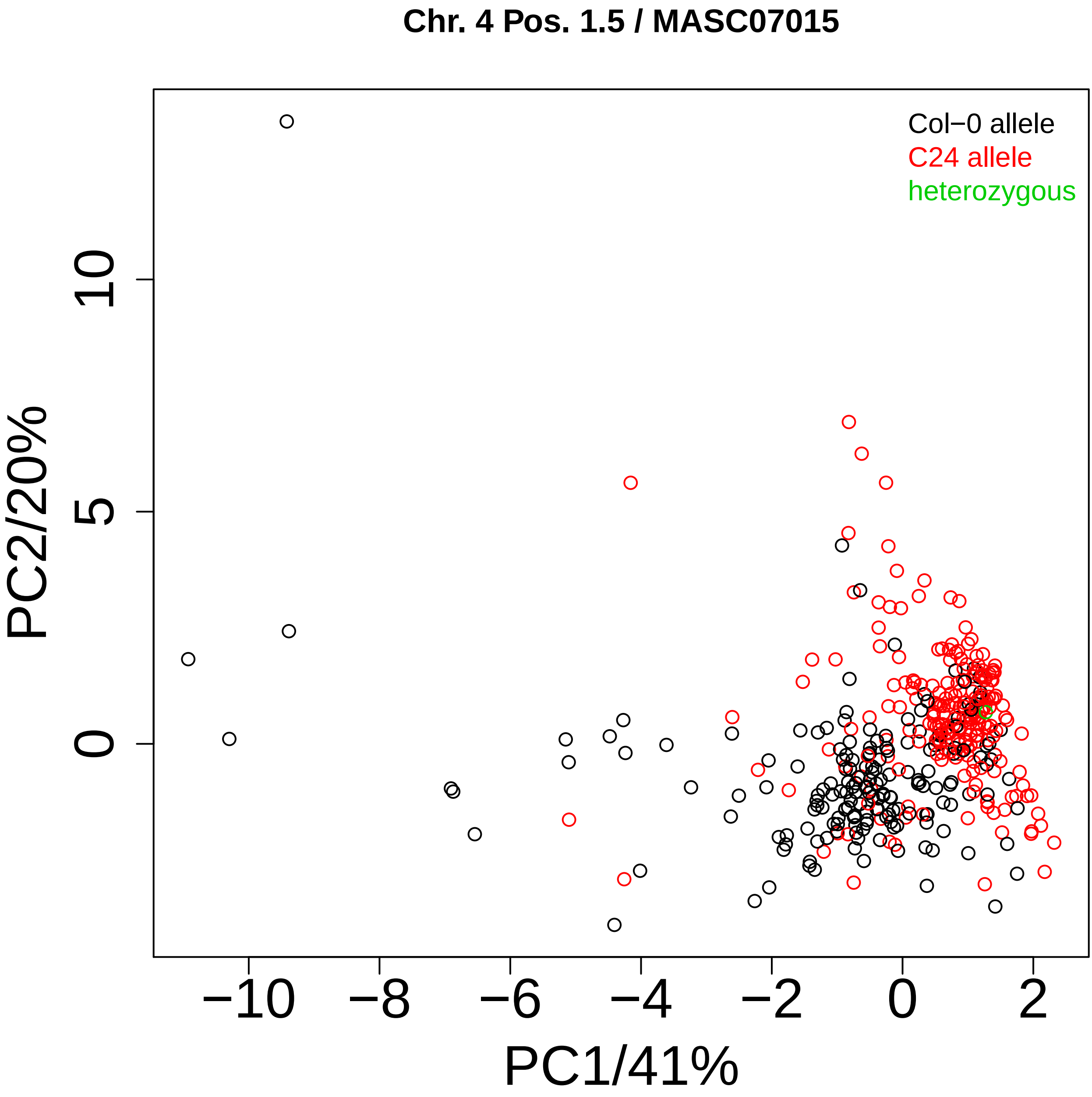
Chr. 4 Pos. 1.5 / MASC07015
Col−0 allele
C24 allele
heterozygous
PC2/20%
PC1/41%
0
5
10
−10
−8
−6
−4
−2
0
2

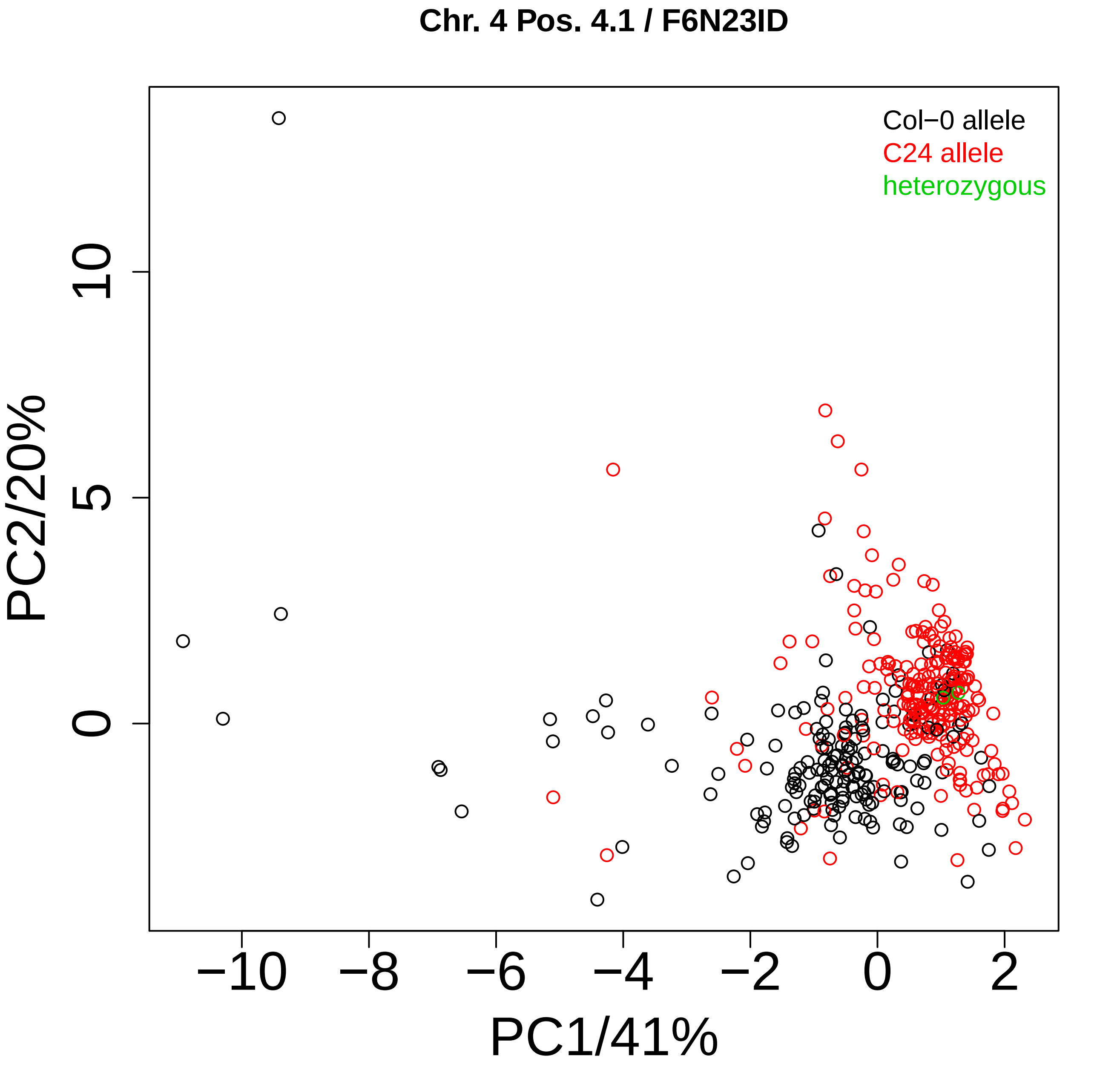
Chr. 4 Pos. 4.1 / F6N23ID
Col−0 allele
C24 allele
heterozygous
PC2/20%
0
5
10
PC1/41%
−10
−8
−6
−4
−2
0
2

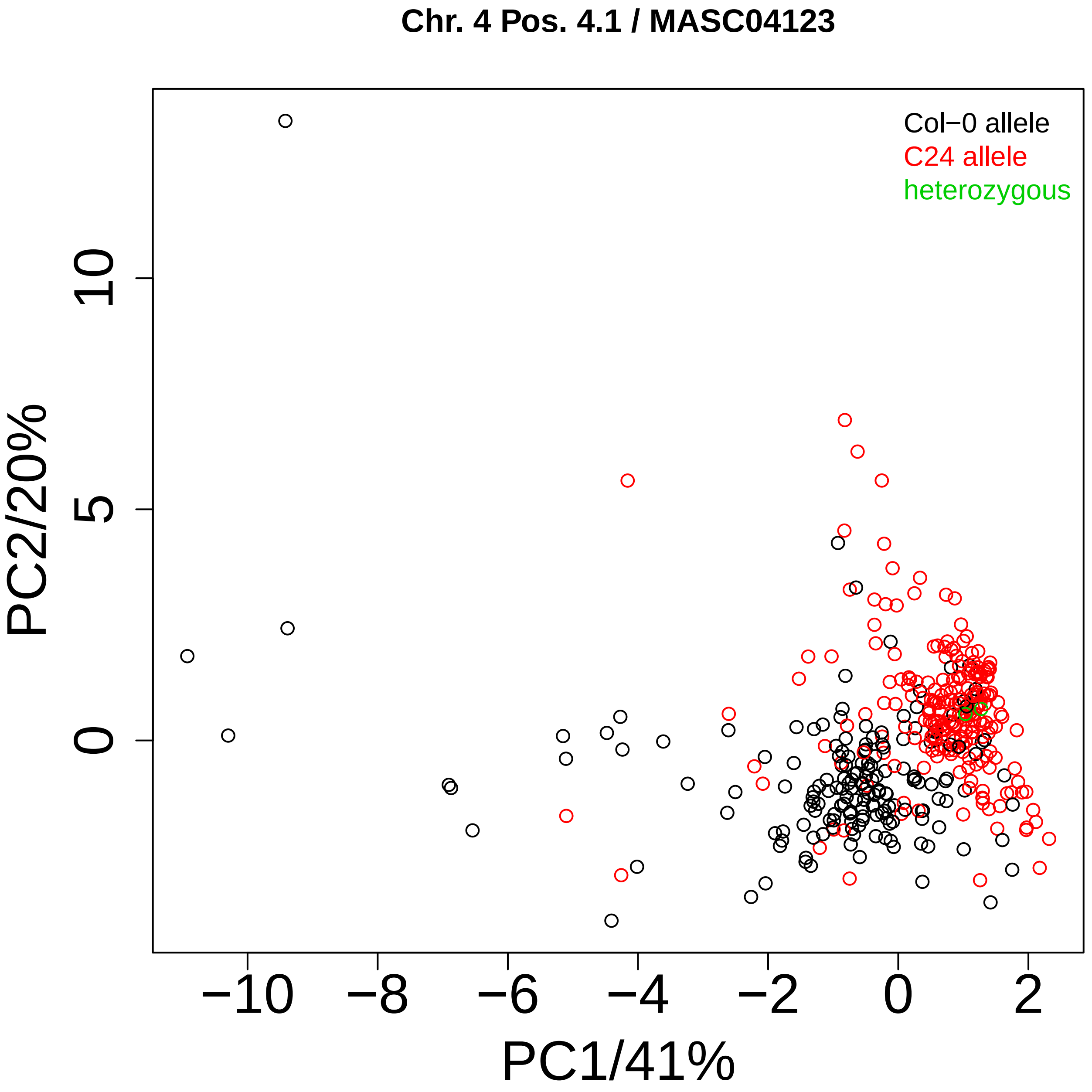
Chr. 4 Pos. 4.1 / MASC04123
Col−0 allele
C24 allele
heterozygous
PC2/20%
PC1/41%
0
5
10
−10
−8
−6
−4
−2
0
2

Chr. 4 Pos. 10.1 / MASC04725

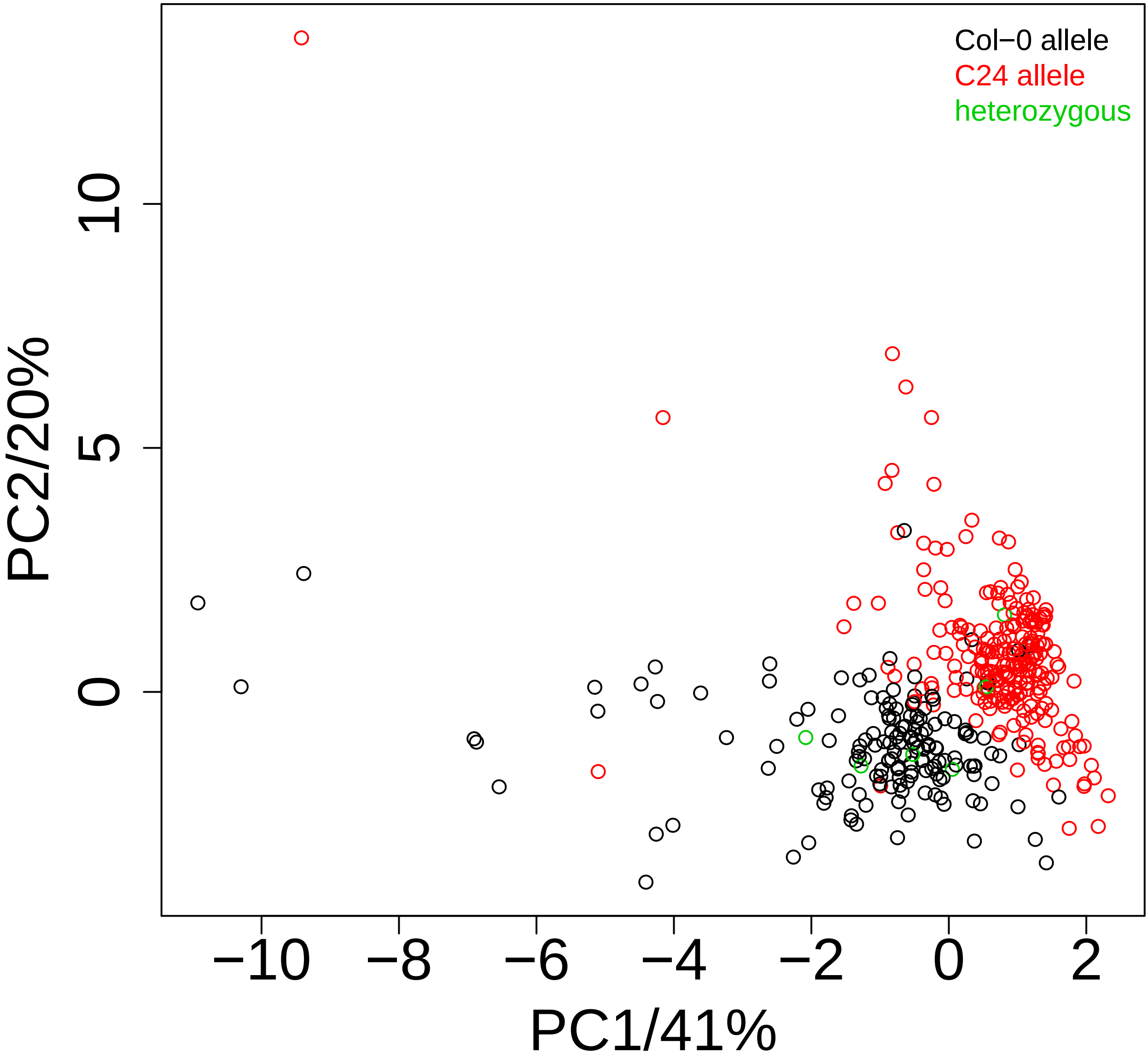

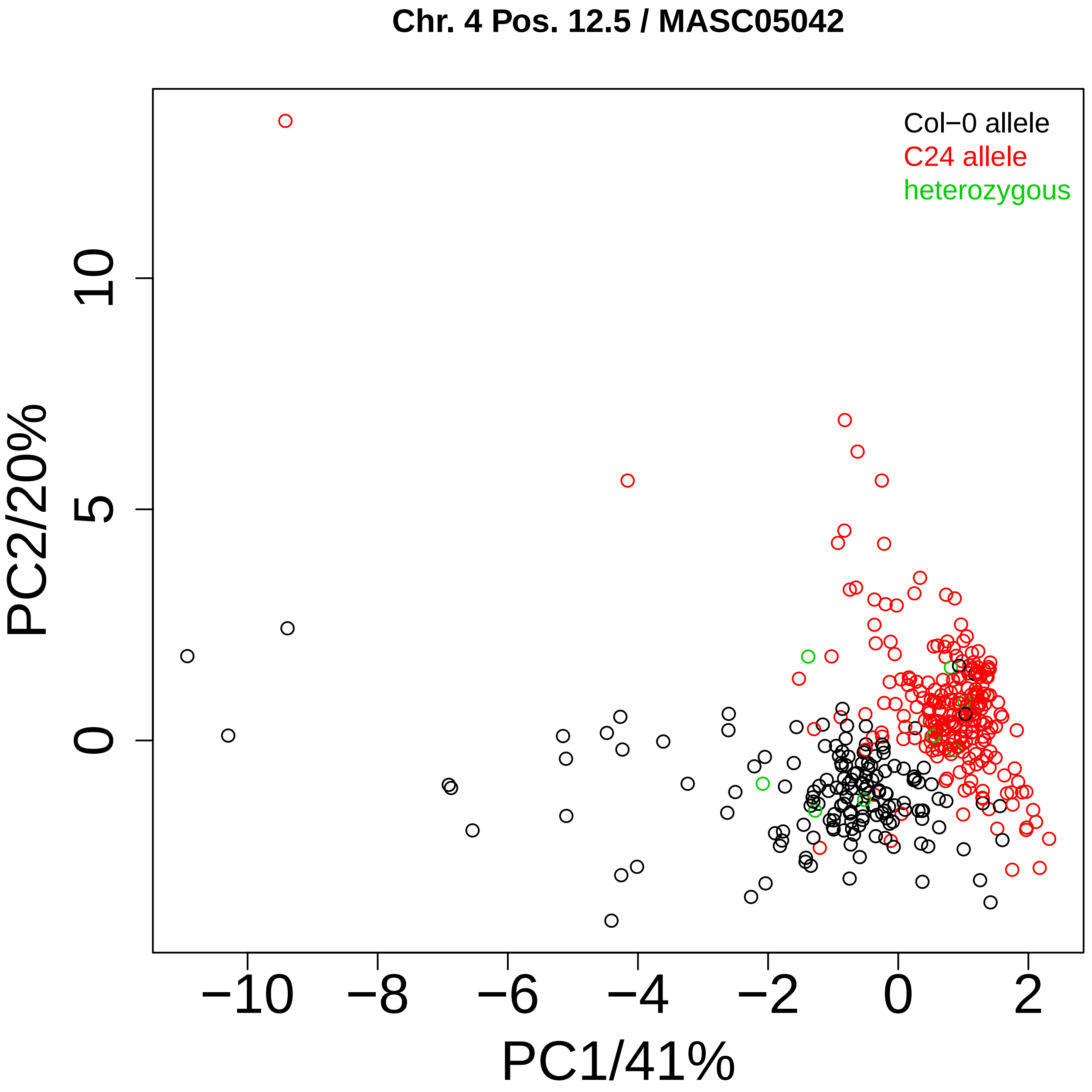
Chr. 4 Pos. 12.5 / MASC05042
Col−0 allele
C24 allele
heterozygous
PC2/20%
PC1/41%
−10
−8
−6
−4
−2
0
2
0
5
10

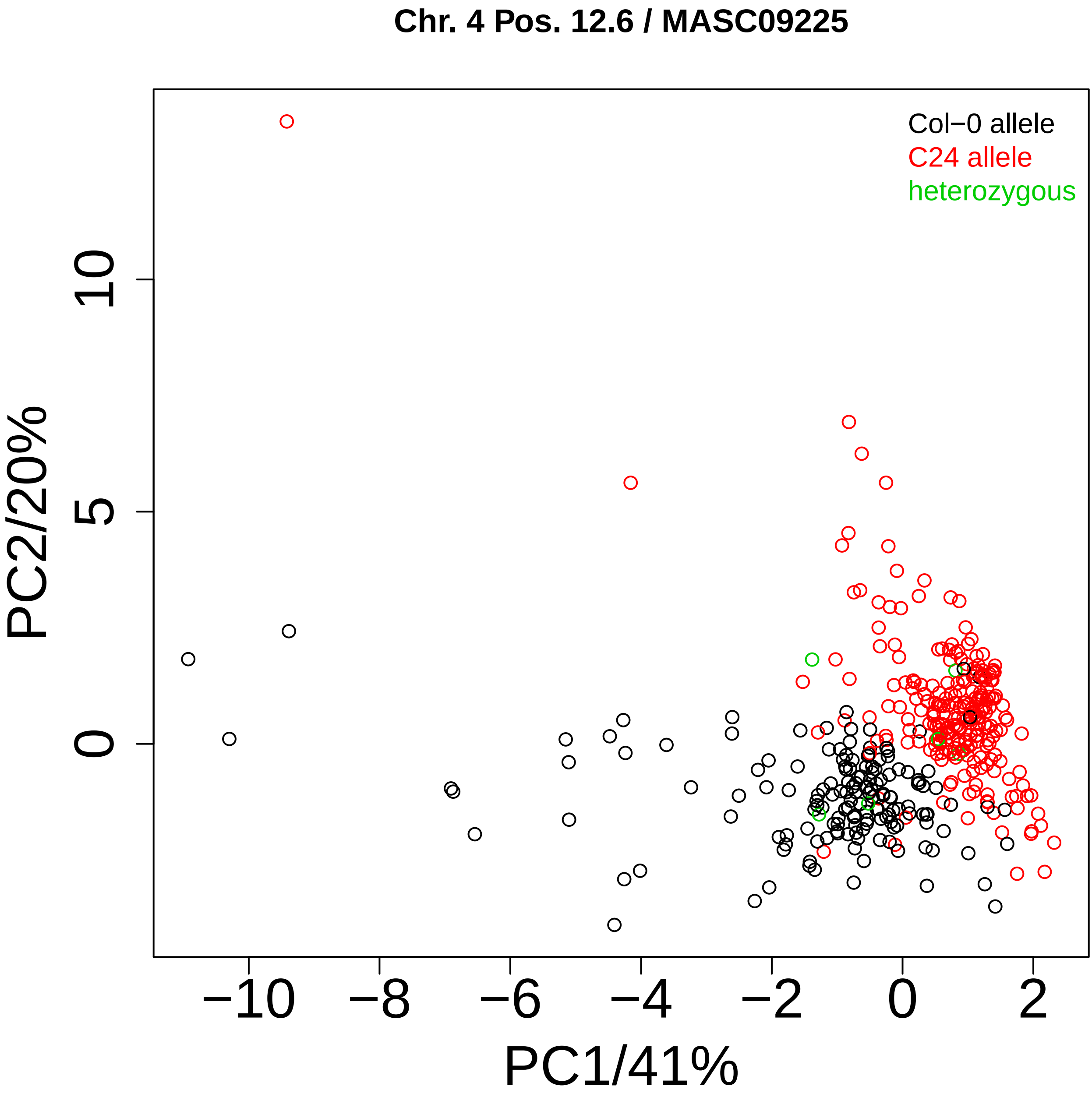
Chr. 4 Pos. 12.6 / MASC09225
Col−0 allele
C24 allele
heterozygous
PC1/41%
PC2/20%
−10
−8
−6
−4
−2
0
2
0
5
10

Chr. 4 Pos. 13.4 / MASC04685

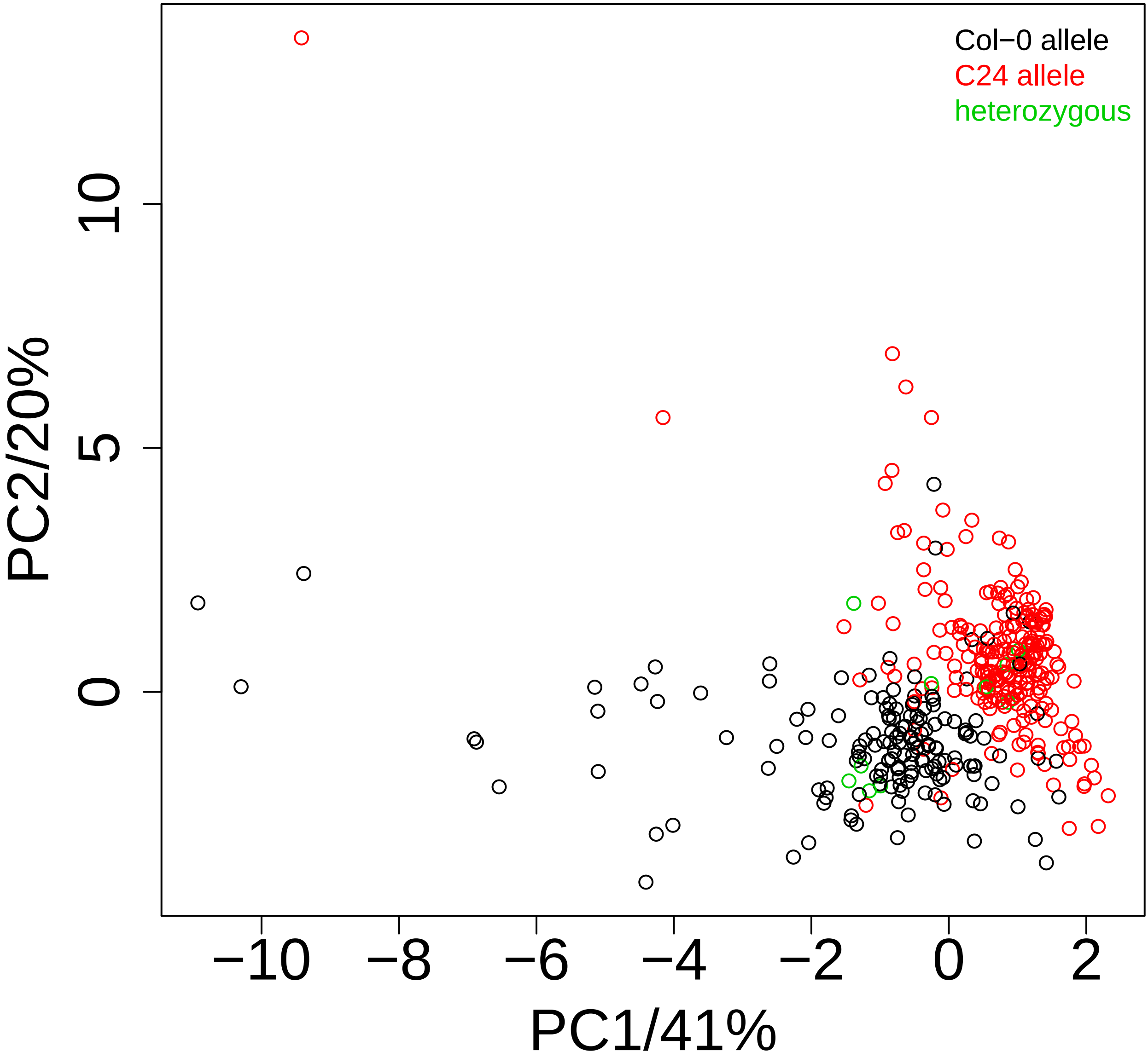

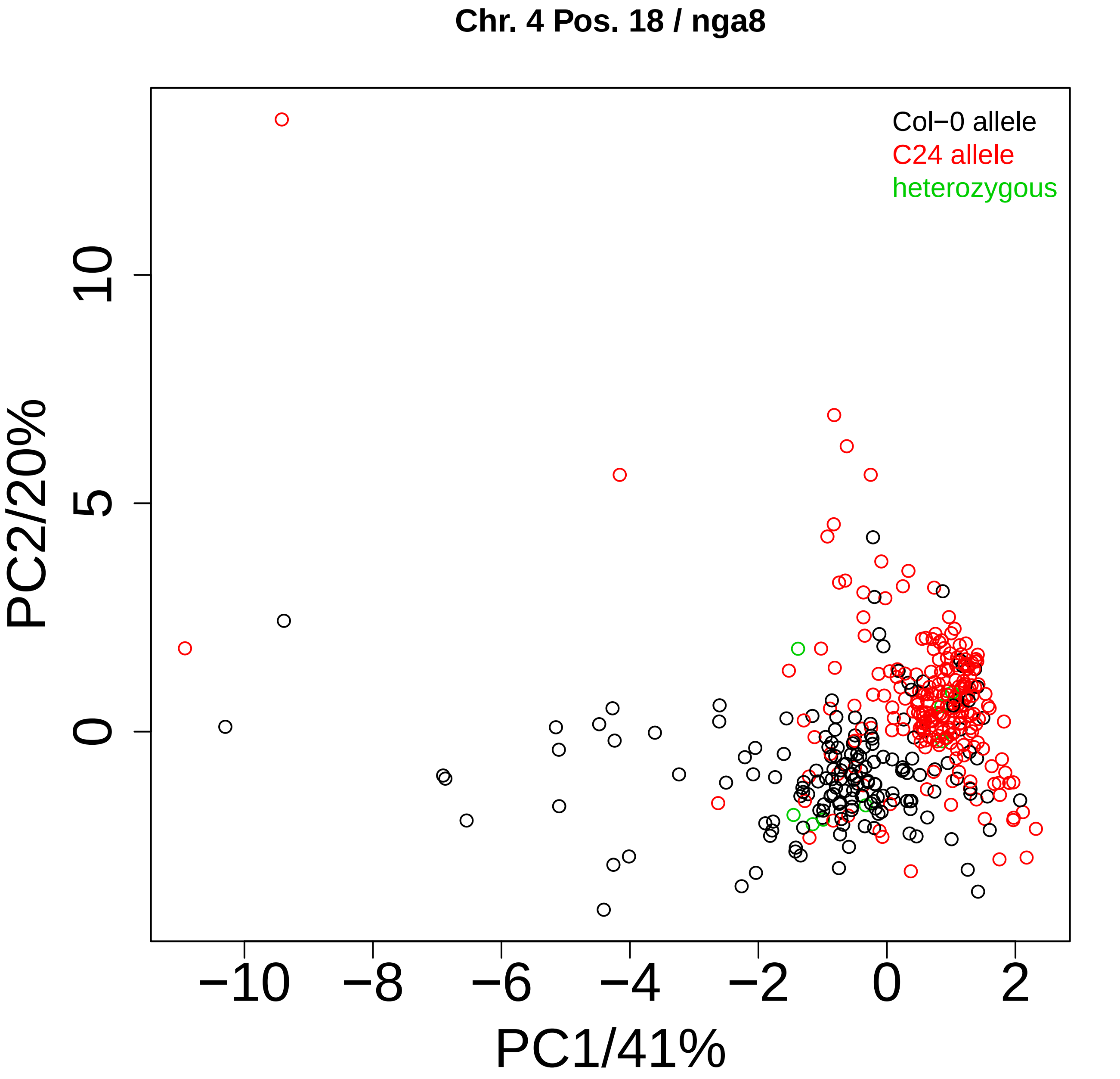

Chr. 4 Pos. 18 / nga8
Col−0 allele
C24 allele
heterozygous
PC2/20%
PC1/41%
0
5
10
−10
−8
−6
−4
−2
0
2

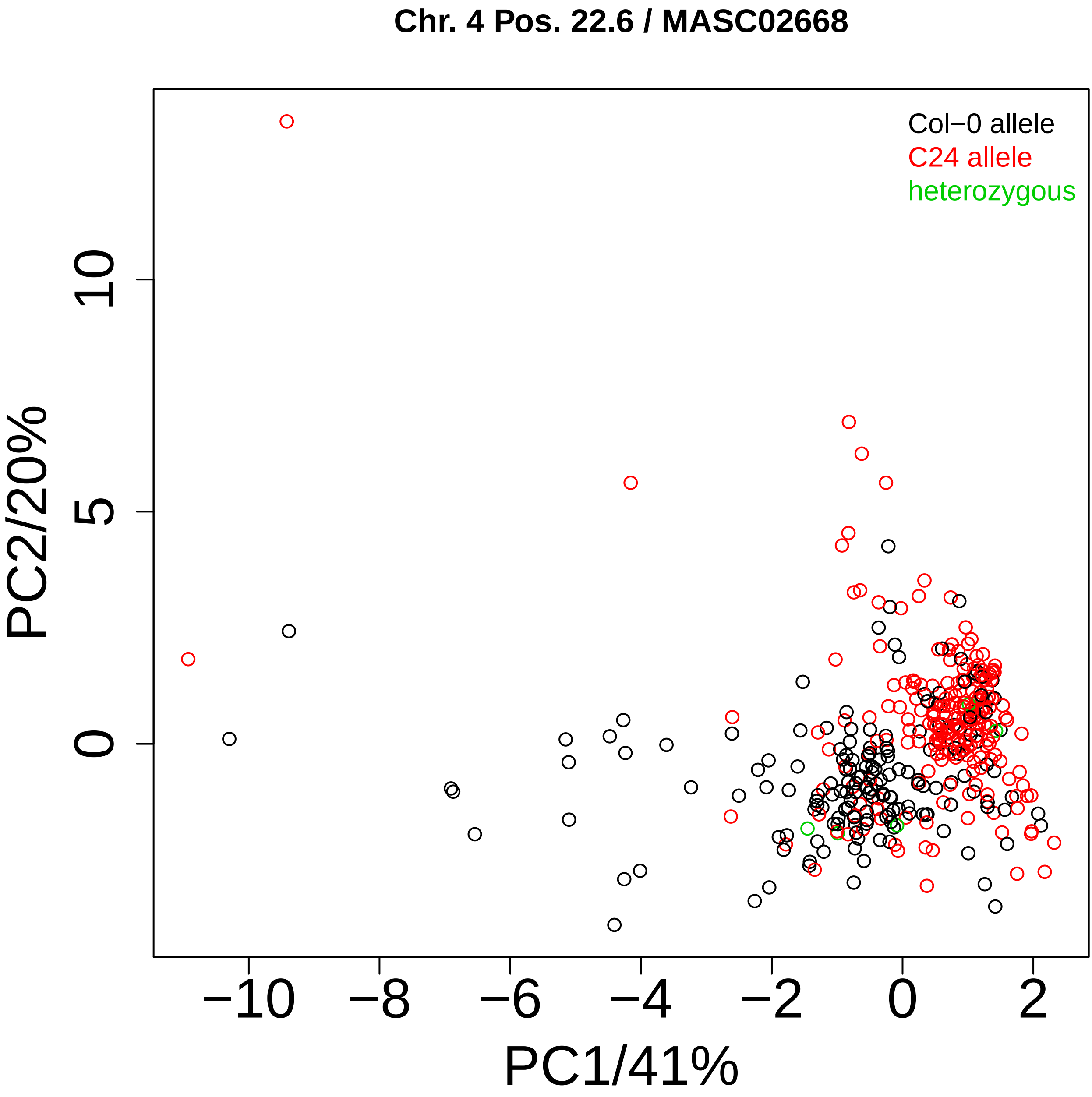
Chr. 4 Pos. 22.6 / MASC02668
Col−0 allele
C24 allele
heterozygous
PC2/20%
0
5
10
PC1/41%
−10
−8
−6
−4
−2
0
2

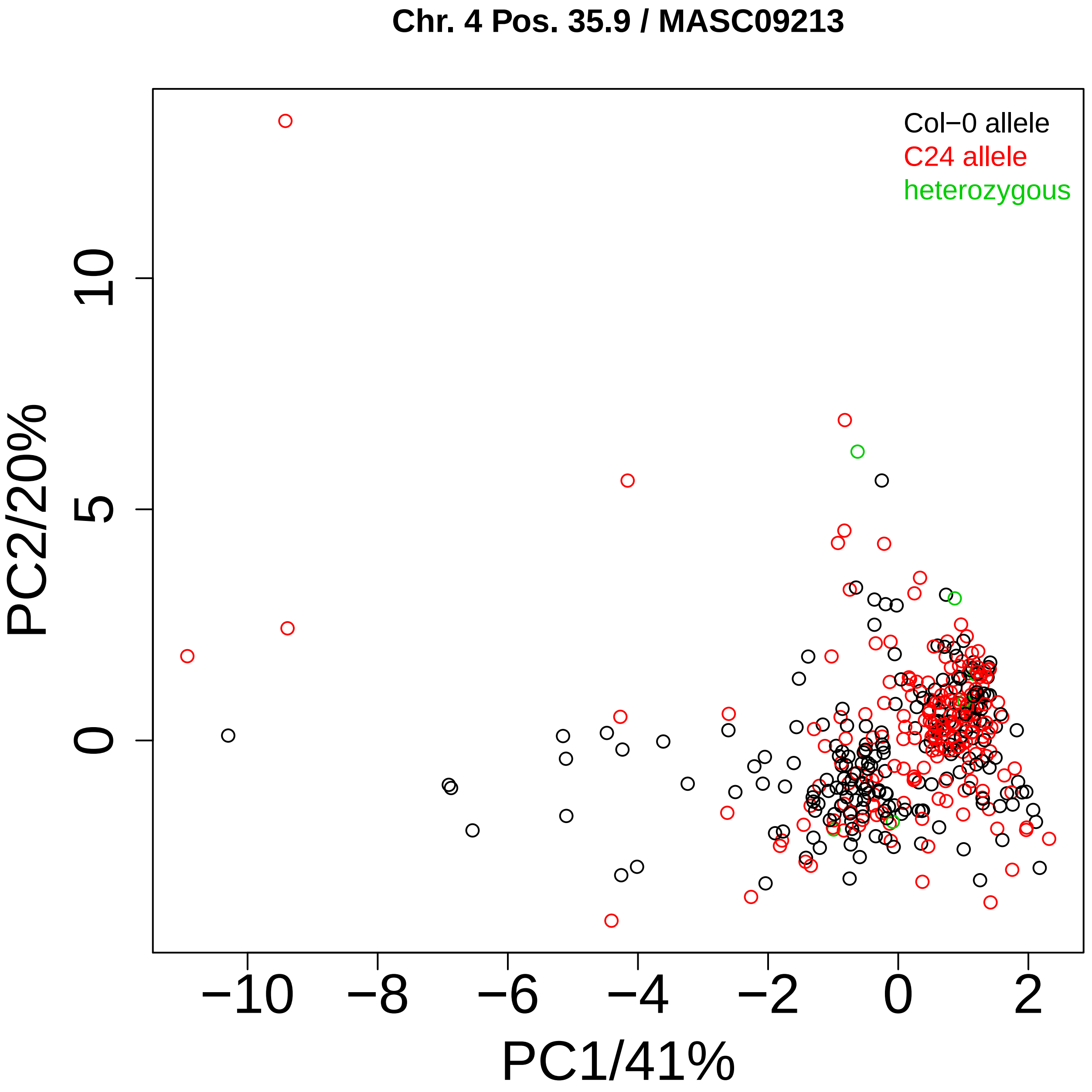
Chr. 4 Pos. 35.9 / MASC09213
Col−0 allele
C24 allele
heterozygous
PC2/20%
PC1/41%
0
5
10
−10
−8
−6
−4
−2
0
2

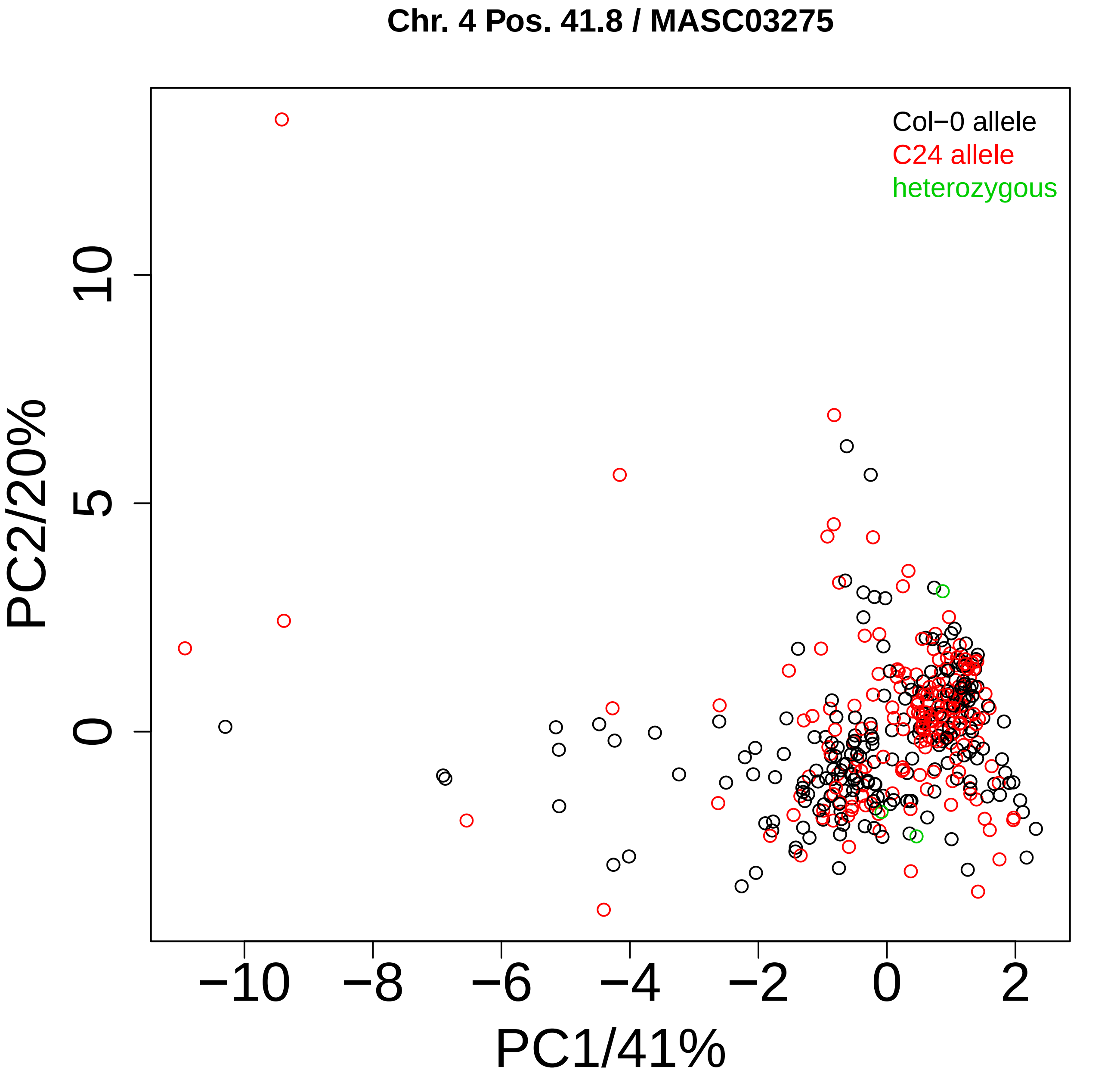

Chr. 4 Pos. 41.8 / MASC03275
Col−0 allele
C24 allele
heterozygous
PC2/20%
0
5
10
PC1/41%
−10
−8
−6
−4
−2
0
2

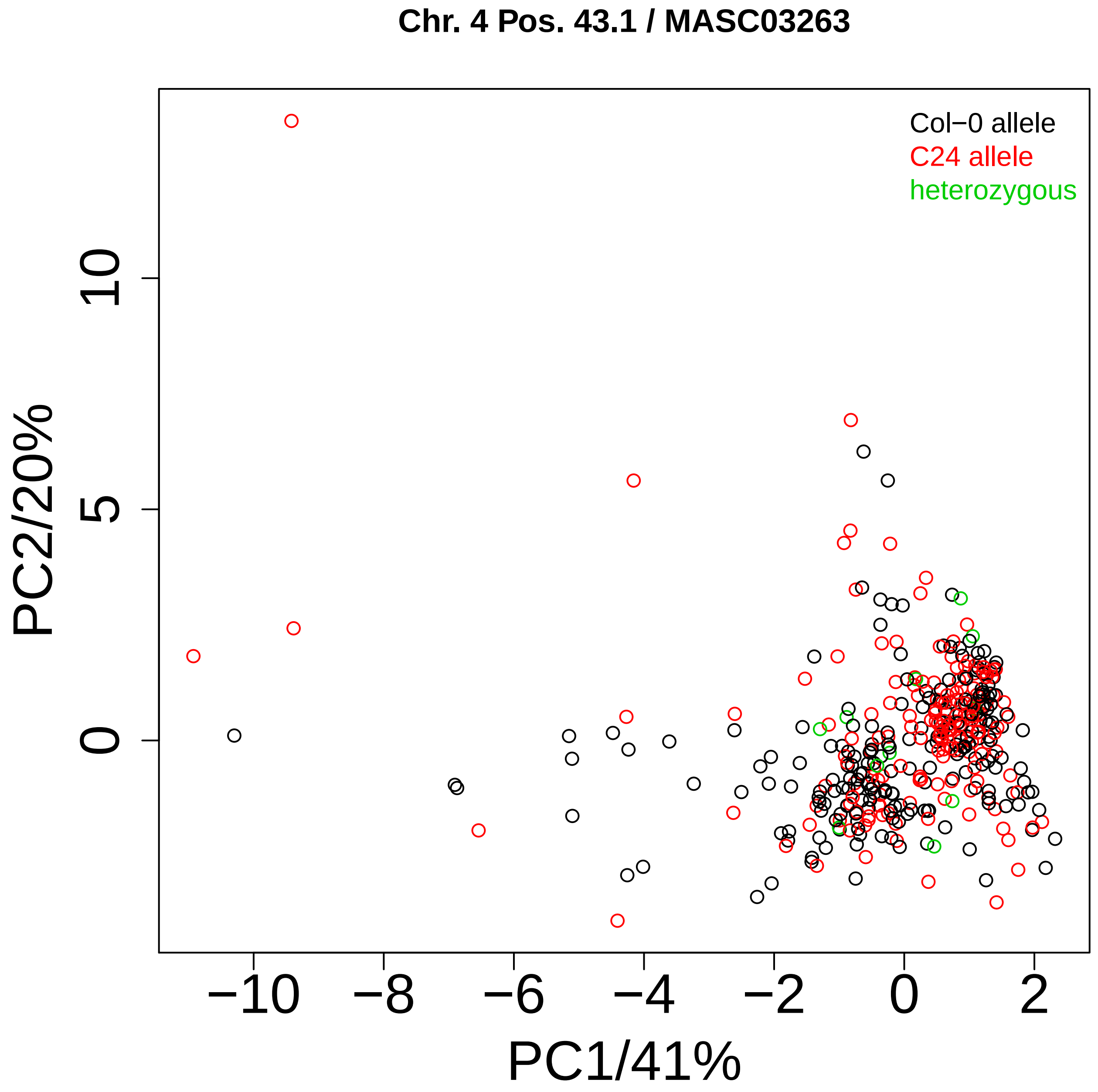
Chr. 4 Pos. 43.1 / MASC03263
Col−0 allele
C24 allele
heterozygous
PC2/20%
0
5
10
PC1/41%
−10
−8
−6
−4
−2
0
2

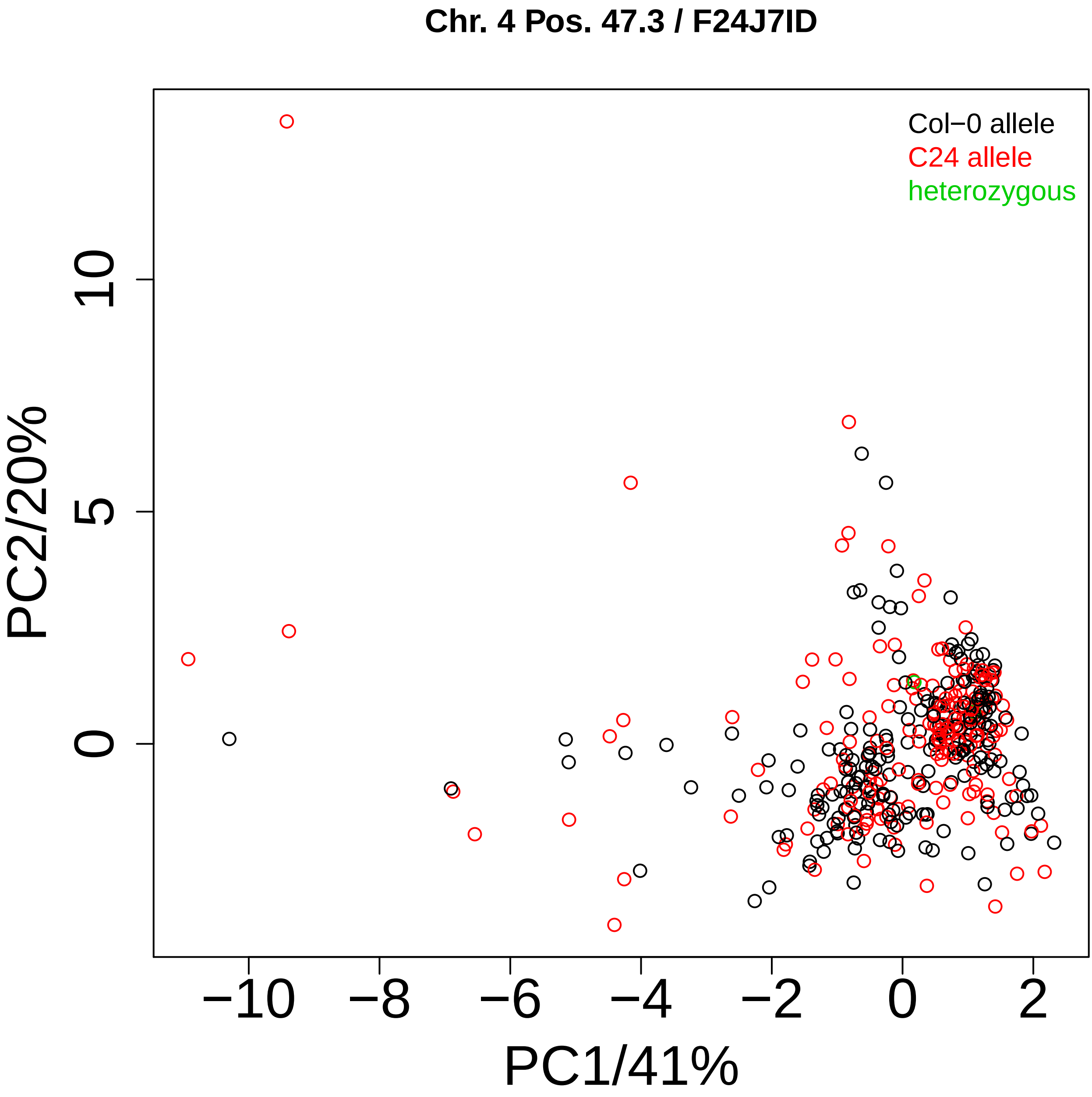

Chr. 4 Pos. 47.3 / F24J7ID
Col−0 allele
C24 allele
heterozygous
PC2/20%
0
5
10
PC1/41%
−10
−8
−6
−4
−2
0
2

Chr. 4 Pos. 47.3 / MASC02548

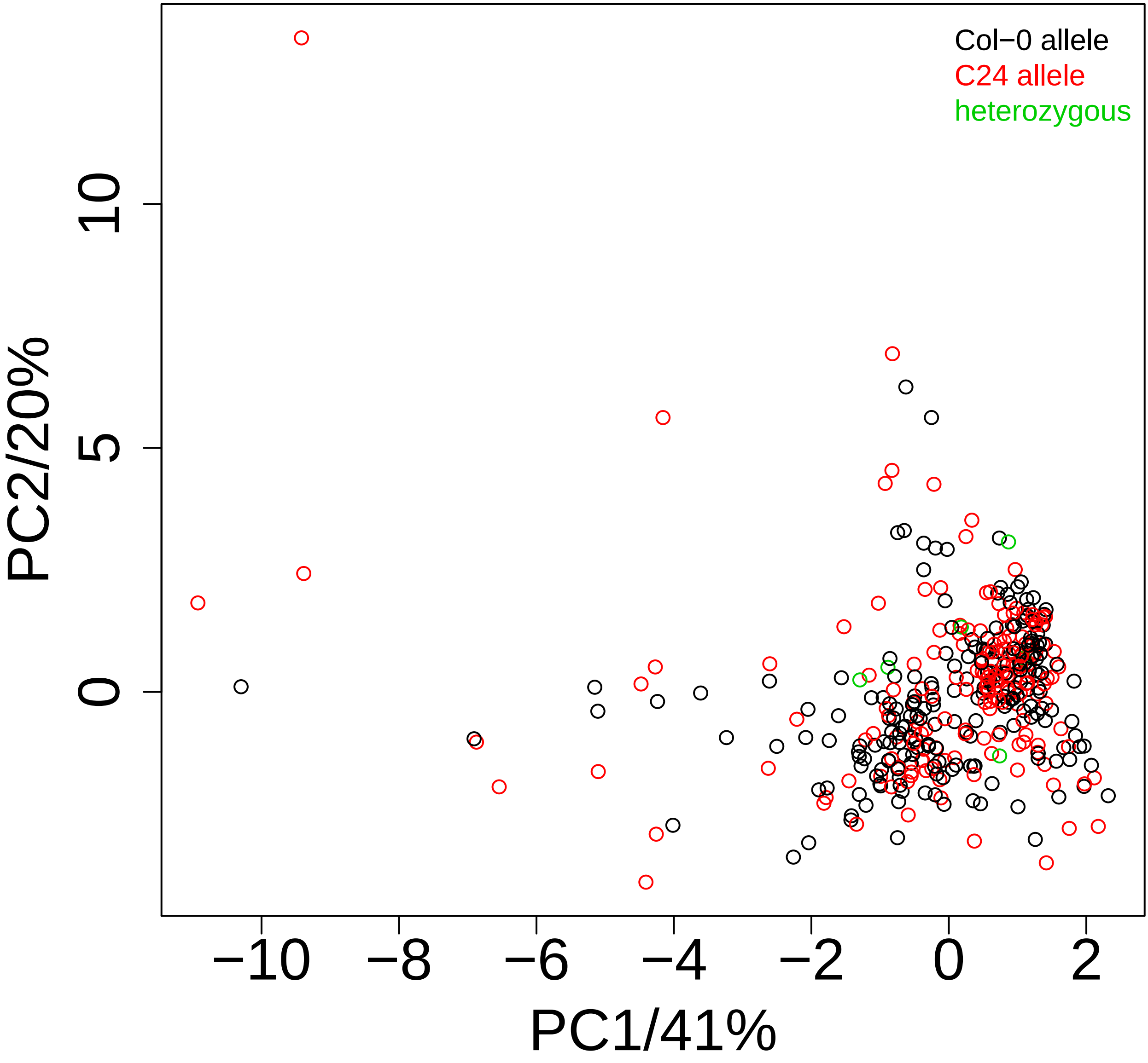

Chr. 4 Pos. 53.1 / MASC04642

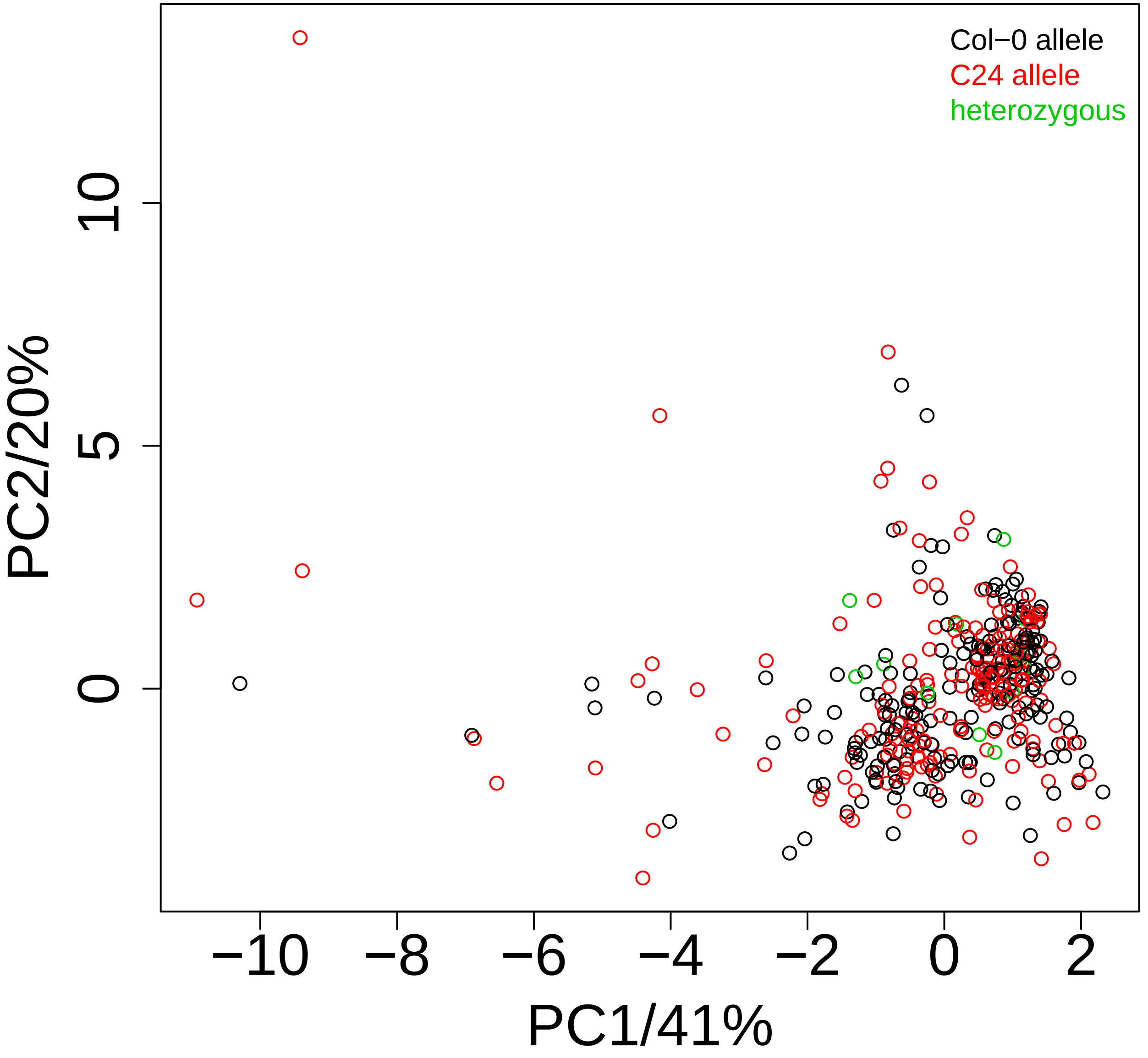

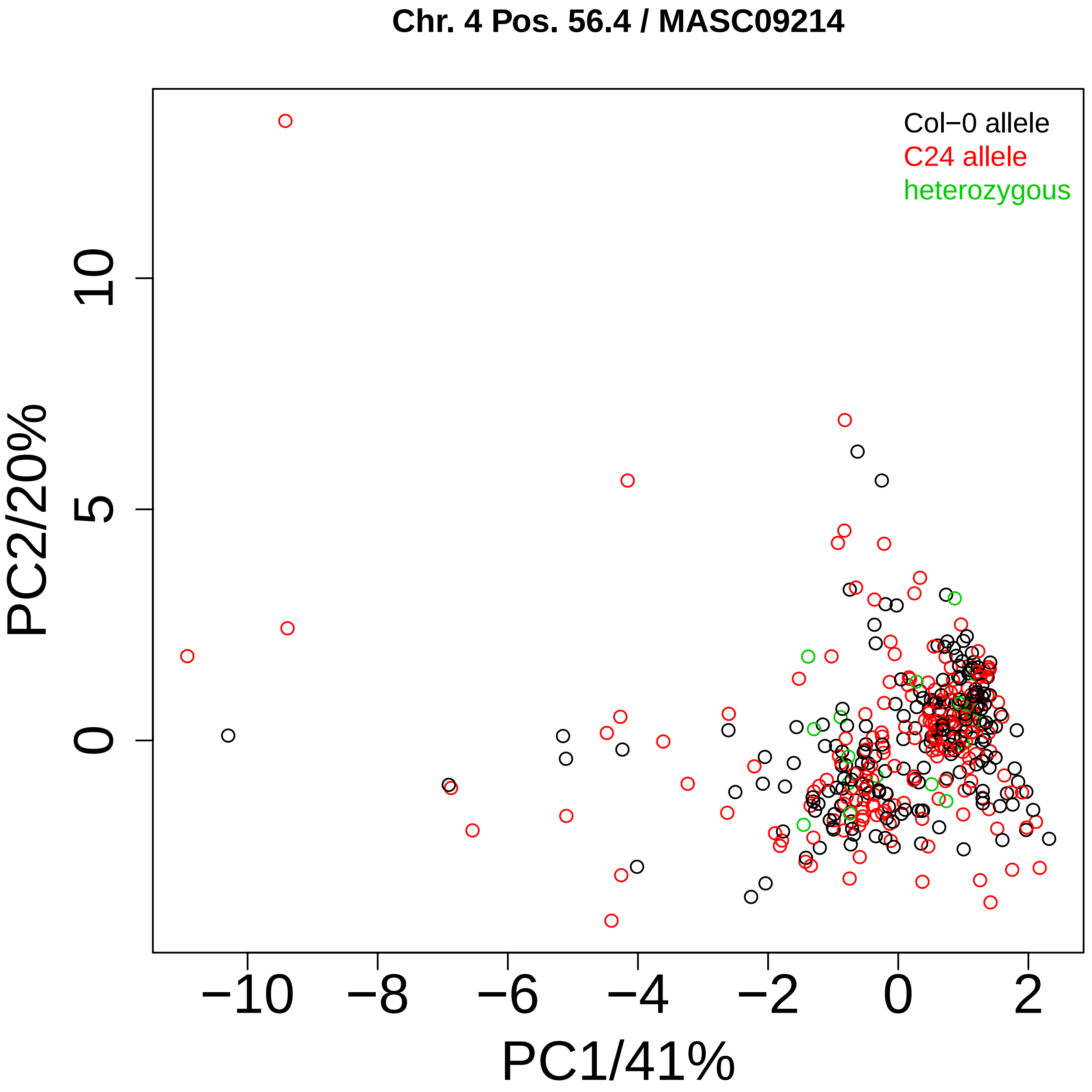

Chr. 4 Pos. 56.4 / MASC09214
Col−0 allele
C24 allele
heterozygous
PC2/20%
0
5
10
PC1/41%
−10
−8
−6
−4
−2
0
2

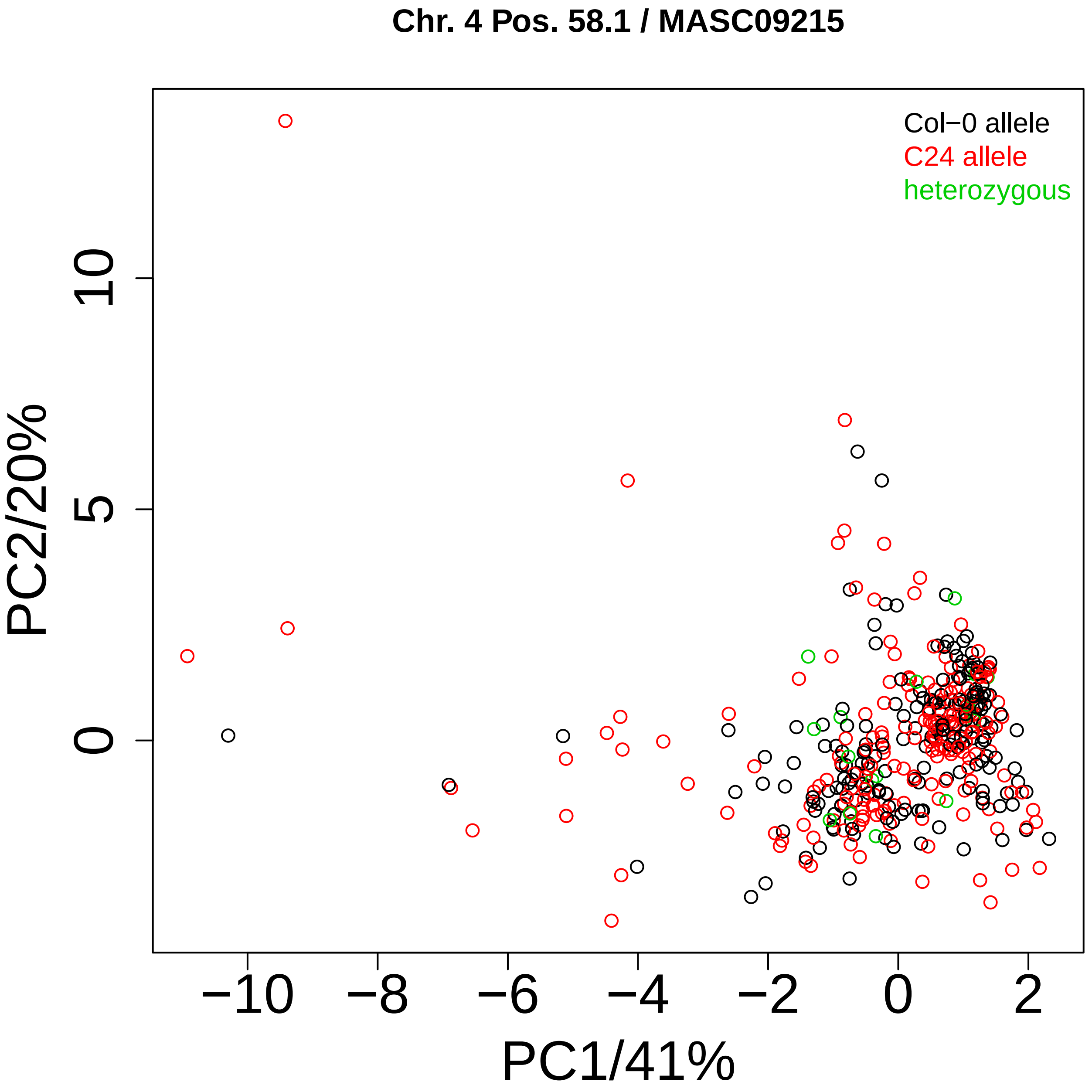
Chr. 4 Pos. 58.1 / MASC09215
Col−0 allele
C24 allele
heterozygous
PC2/20%
PC1/41%
0
5
10
−10
−8
−6
−4
−2
0
2

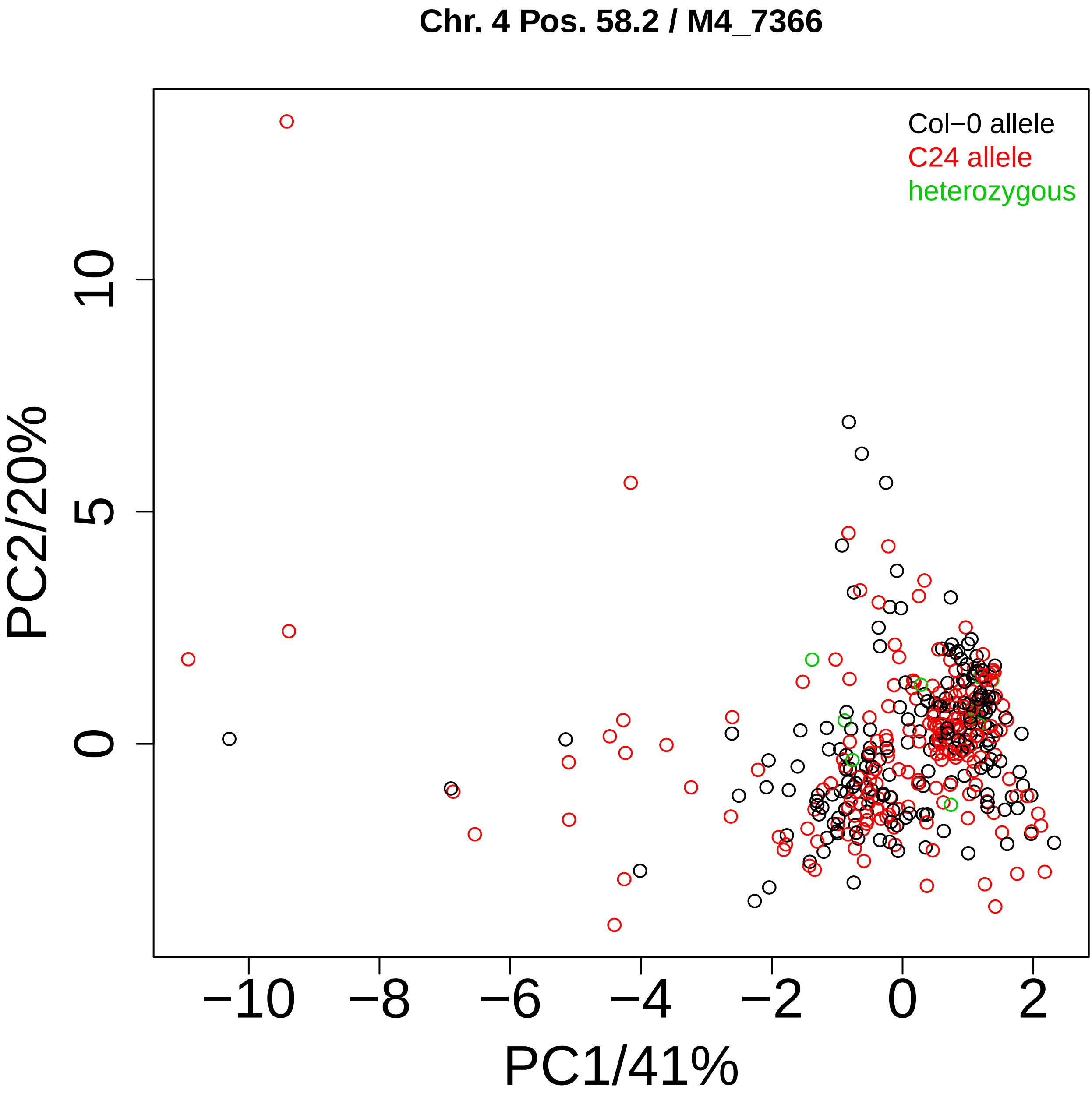
Chr. 4 Pos. 58.2 / M4_7366
Col−0 allele
C24 allele
heterozygous
PC2/20%
PC1/41%
0
5
10
−10
−8
−6
−4
−2
0
2

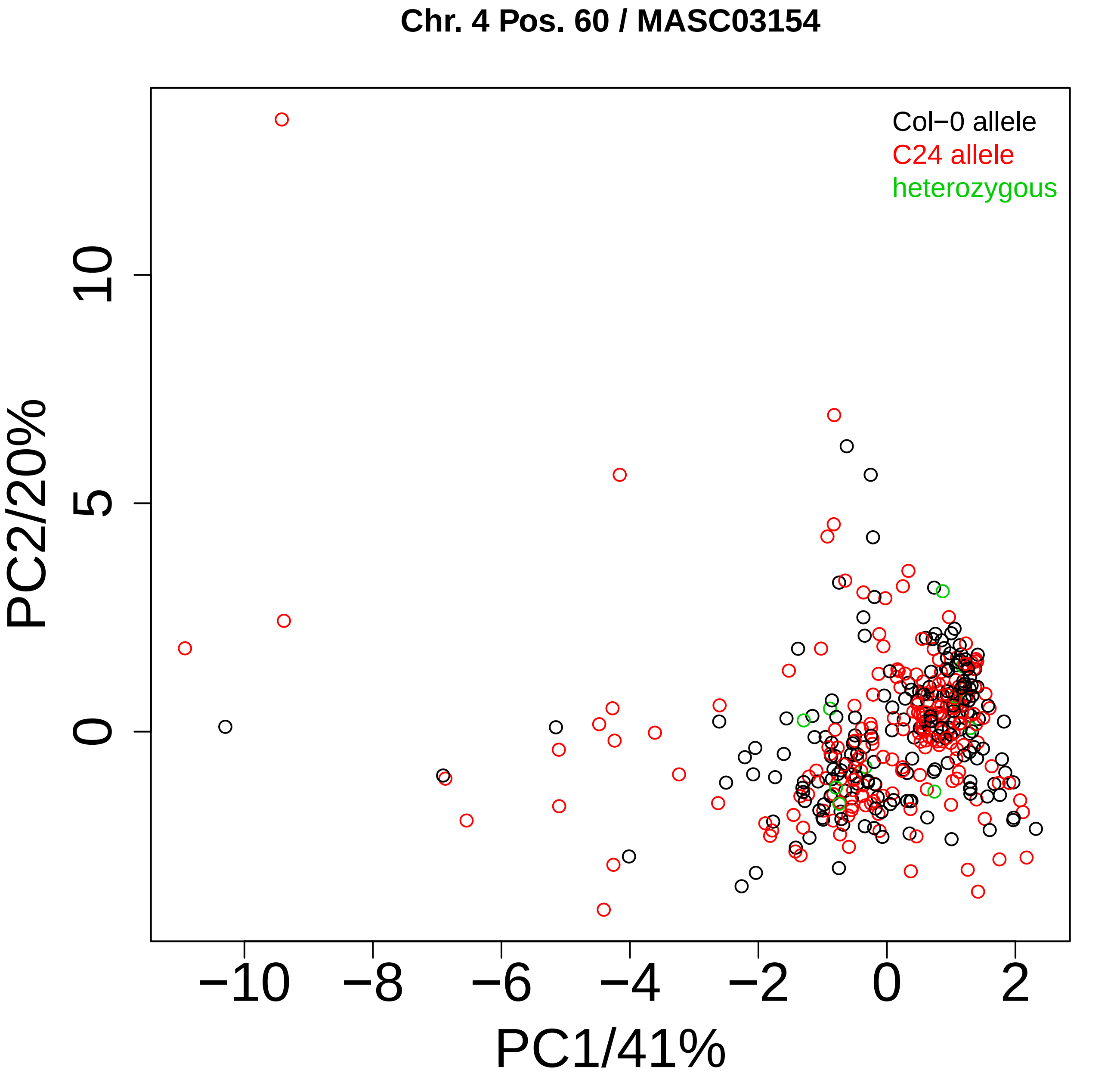

Chr. 4 Pos. 60 / MASC03154
Col−0 allele
C24 allele
heterozygous
PC2/20%
PC1/41%
0
5
10
−10
−8
−6
−4
−2
0
2

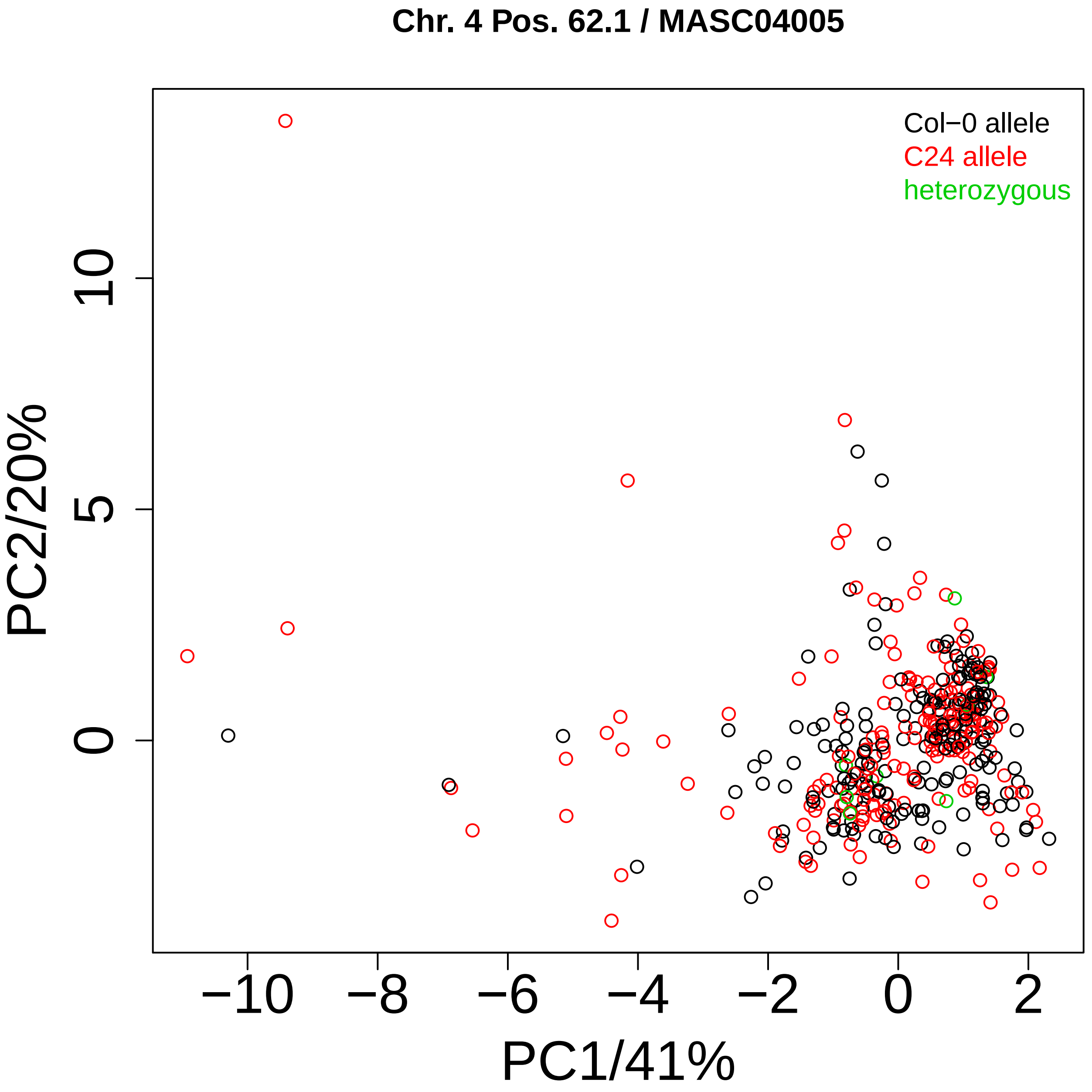
Chr. 4 Pos. 62.1 / MASC04005
Col−0 allele
C24 allele
heterozygous
PC2/20%
10
5
0
PC1/41%
−10
−8
−6
−4
−2
0
2

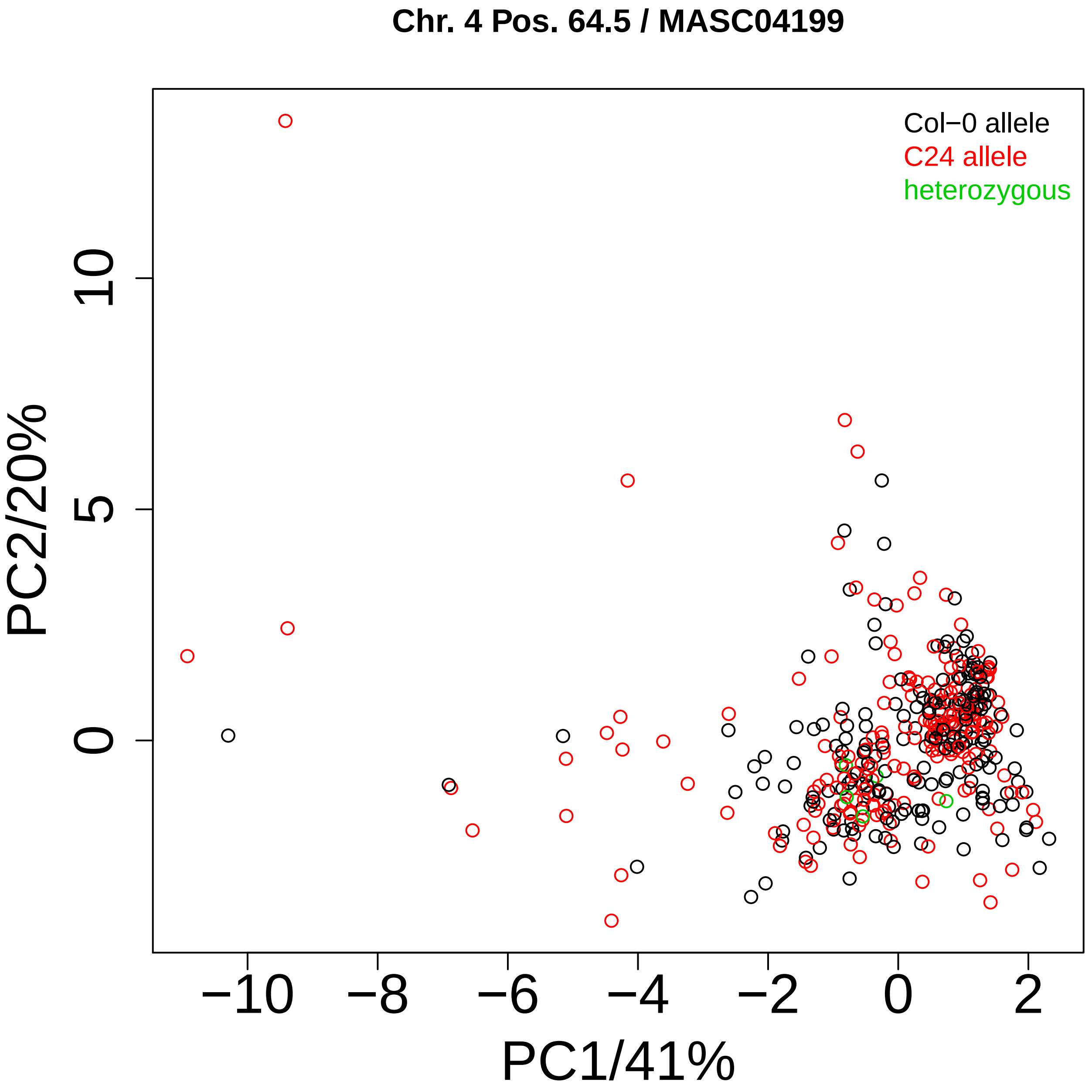

Chr. 4 Pos. 64.5 / MASC04199
Col−0 allele
C24 allele
heterozygous
PC2/20%
PC1/41%
0
5
10
−10
−8
−6
−4
−2
0
2

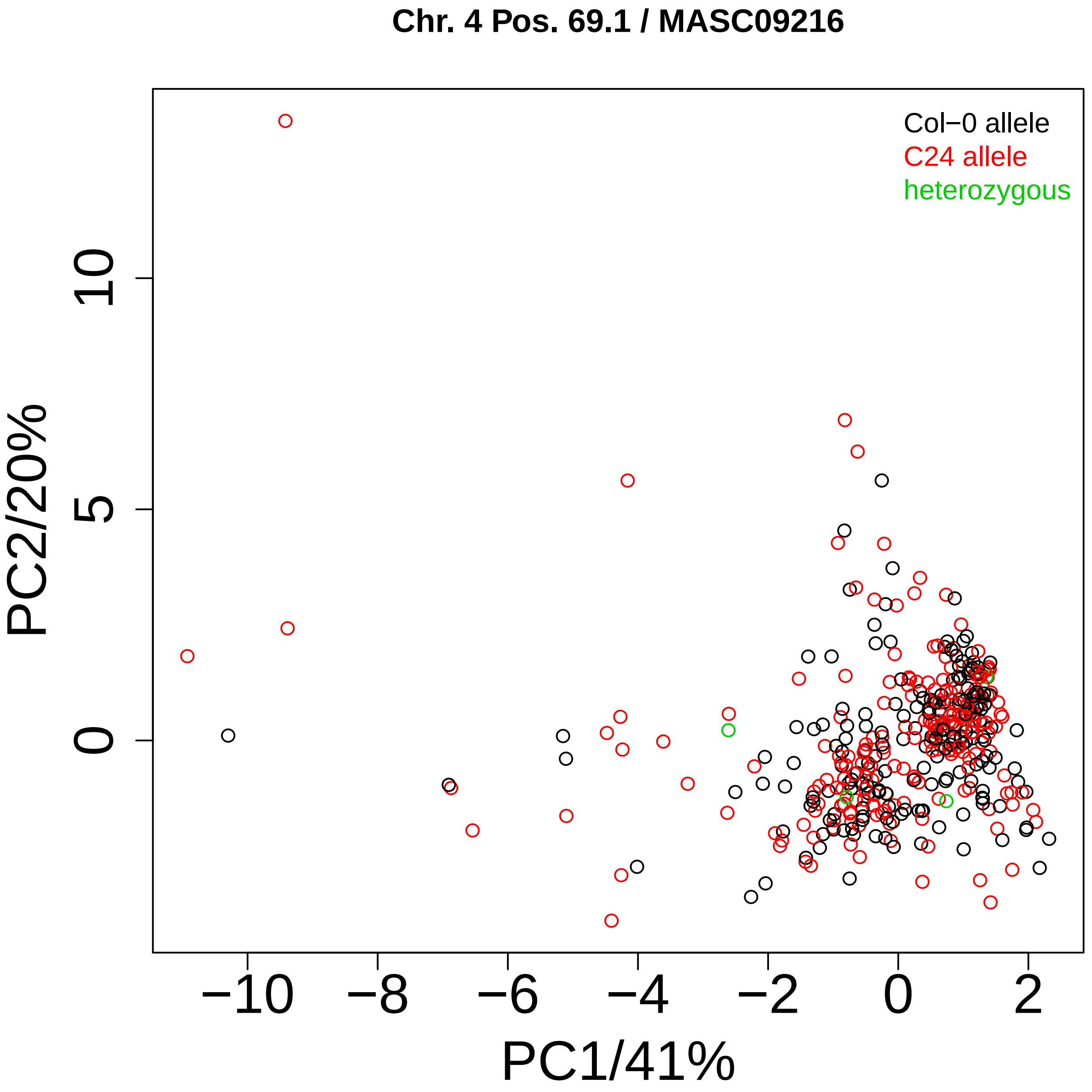
Chr. 4 Pos. 69.1 / MASC09216
Col−0 allele
C24 allele
heterozygous
PC2/20%
PC1/41%
10
5
0
−10
−8
−6
−4
−2
0
2

Chr. 4 Pos. 71.4 / M4_9963

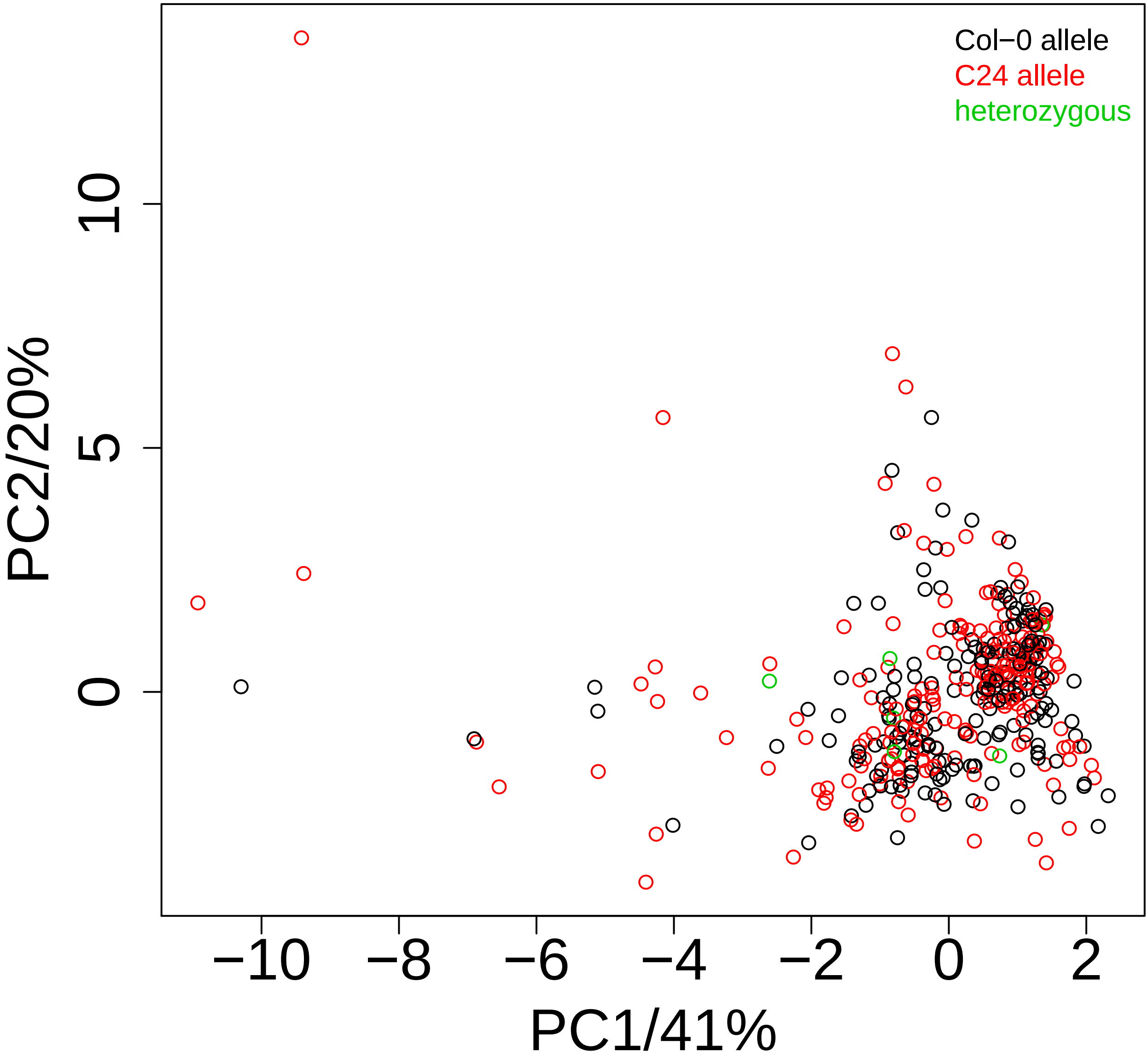

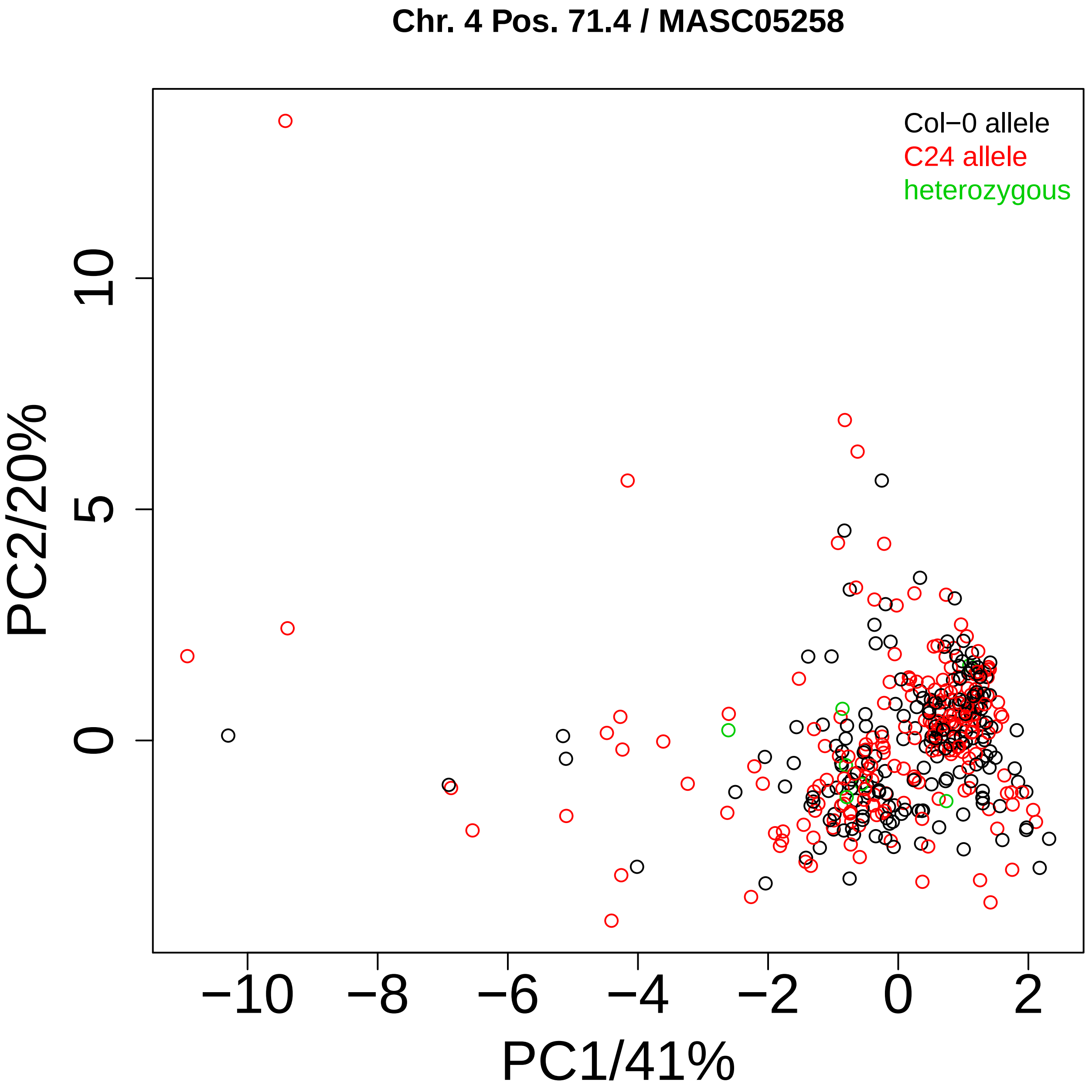
Chr. 4 Pos. 71.4 / MASC05258
Col−0 allele
C24 allele
heterozygous
10
5
0
PC2/20%
−10
−8
−6
−4
−2
0
2
PC1/41%

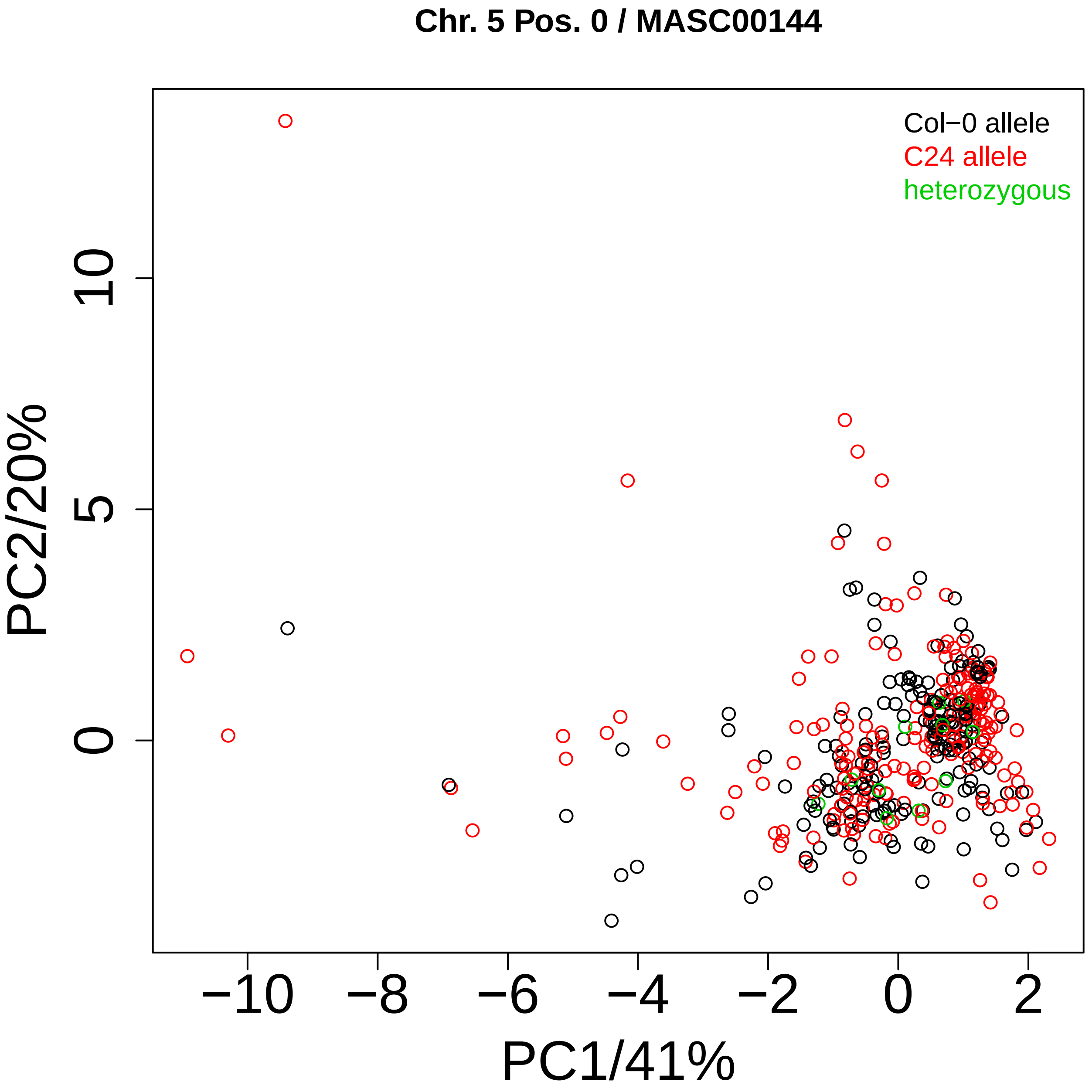

Chr. 5 Pos. 0 / MASC00144
Col−0 allele
C24 allele
heterozygous
PC2/20%
PC1/41%
0
5
10
−10
−8
−6
−4
−2
0
2

Chr. 5 Pos. 2.5 / MASC04860

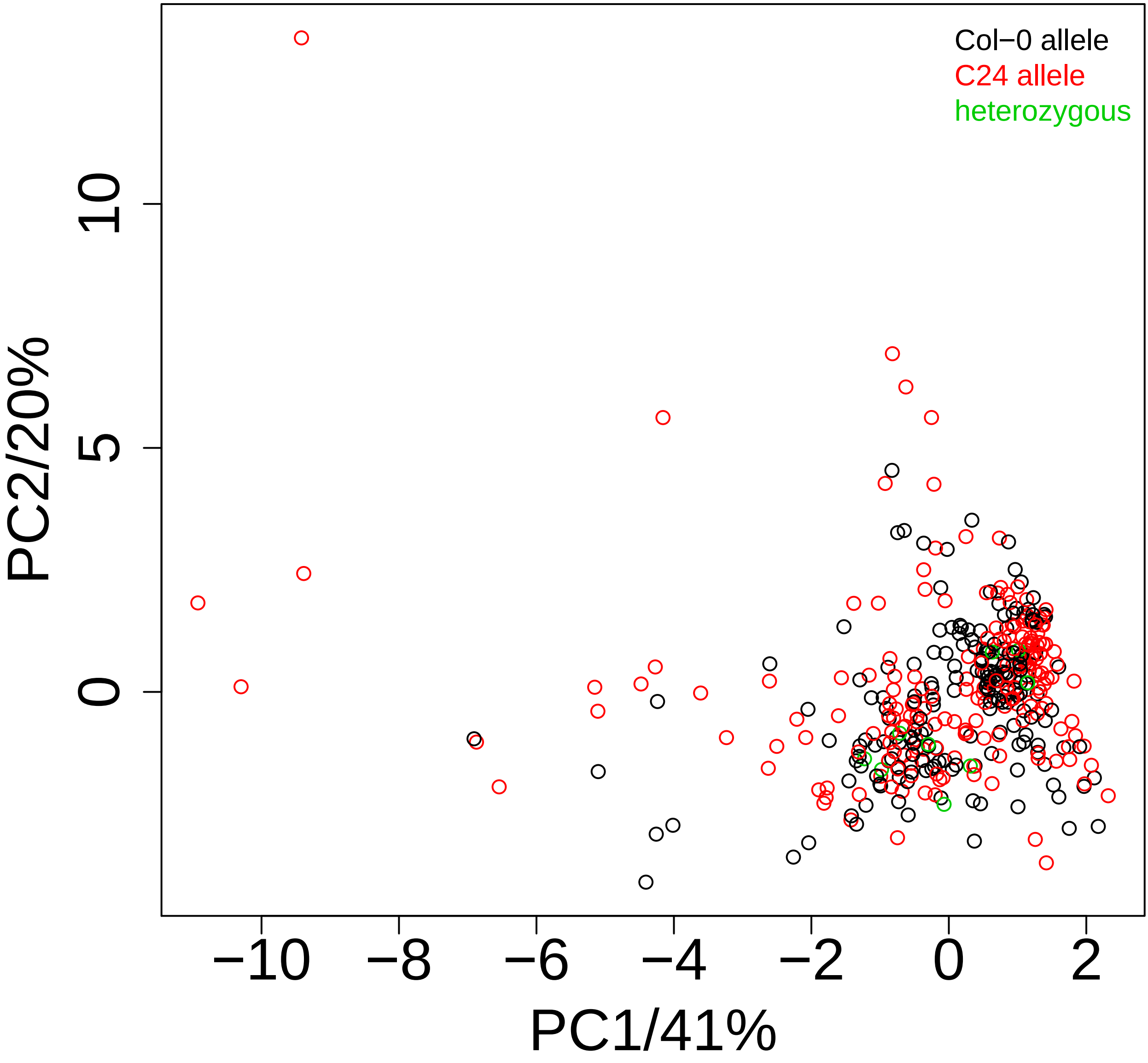

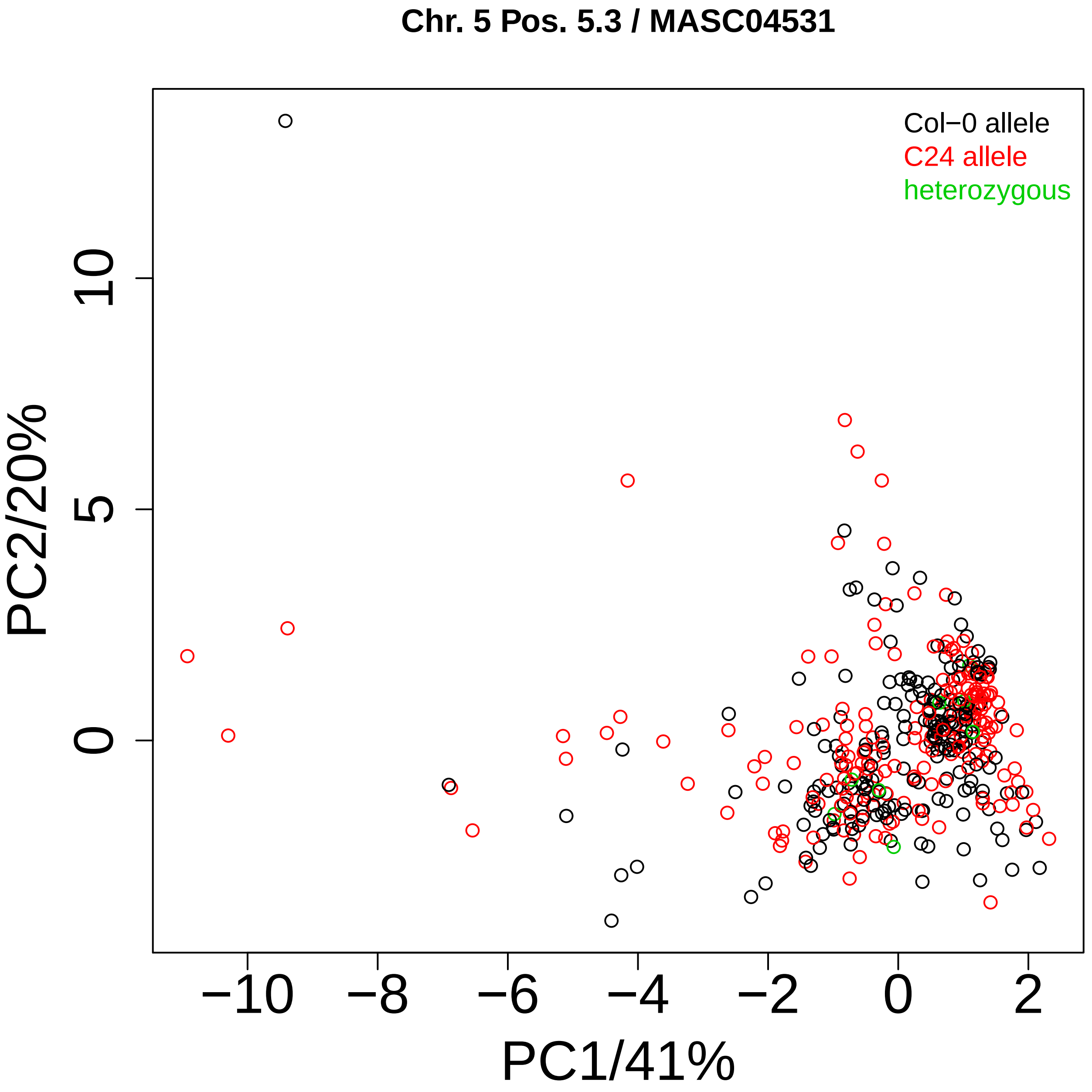
Chr. 5 Pos. 5.3 / MASC04531
Col−0 allele
C24 allele
heterozygous
PC2/20%
0
5
10
−10
−8
−6
−4
−2
0
2
PC1/41%

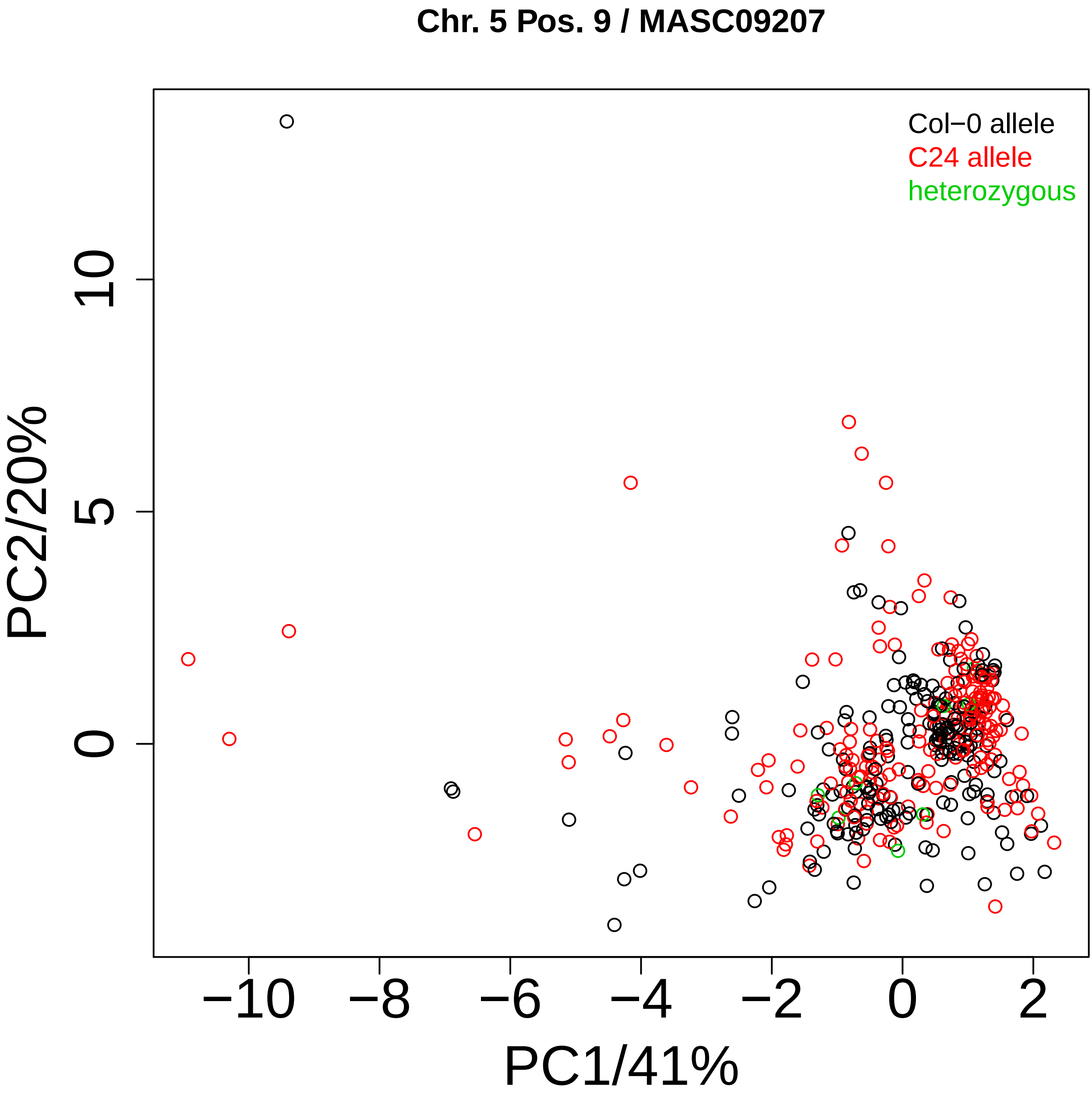
Chr. 5 Pos. 9 / MASC09207
Col−0 allele
C24 allele
heterozygous
PC2/20%
PC1/41%
0
5
10
−10
−8
−6
−4
−2
0
2

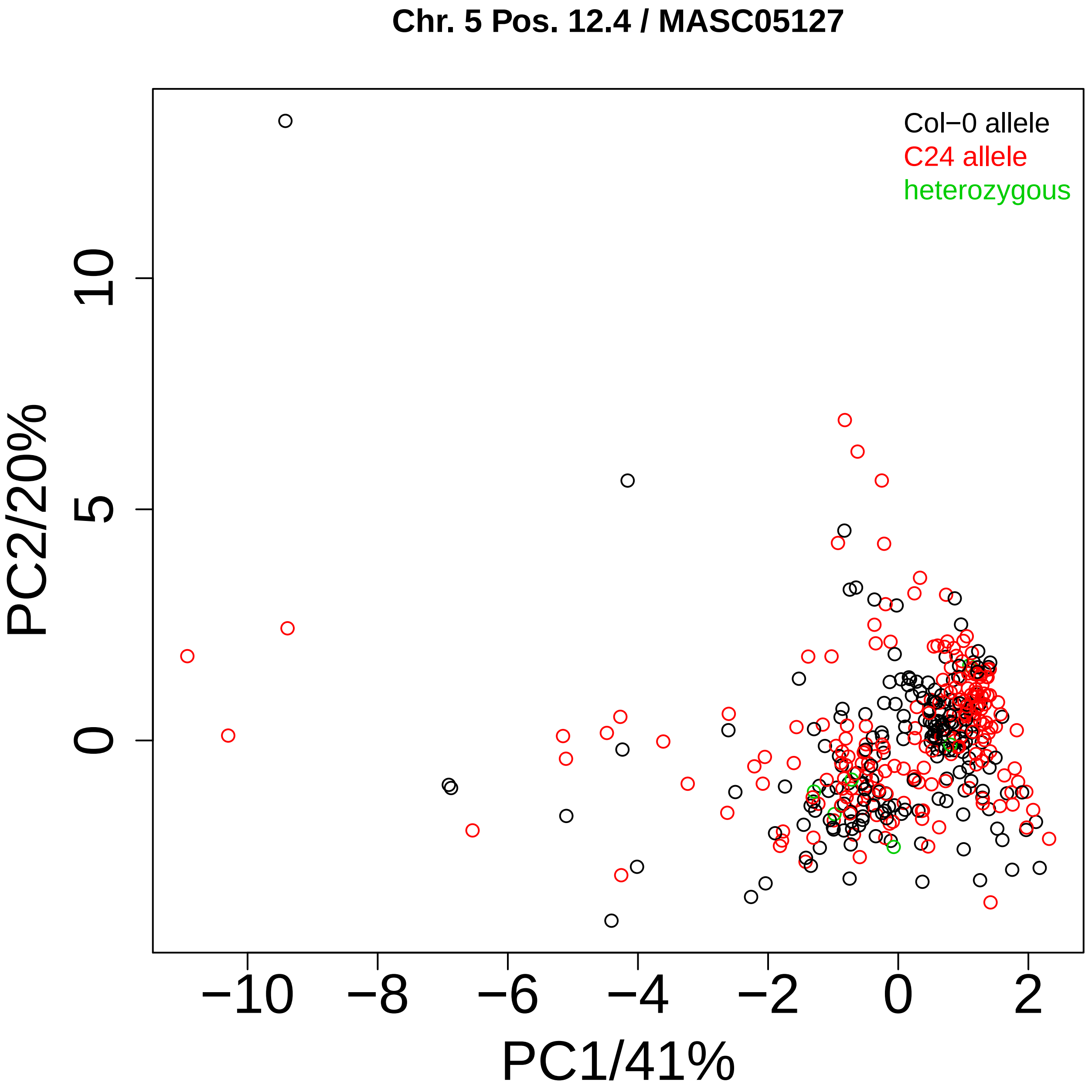
Chr. 5 Pos. 12.4 / MASC05127
Col−0 allele
C24 allele
heterozygous
PC1/41%
PC2/20%
−10
−8
−6
−4
−2
0
2
0
5
10

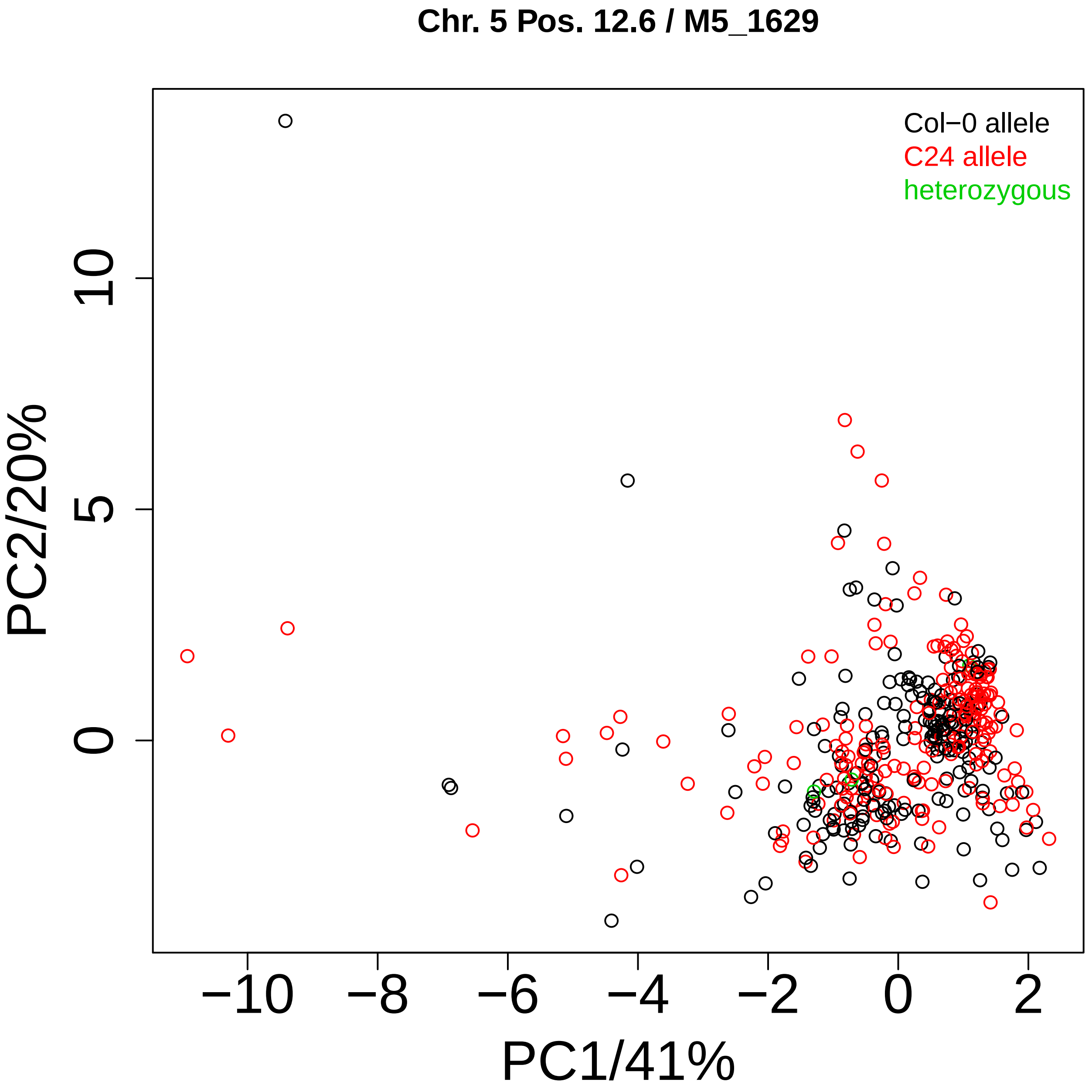
Chr. 5 Pos. 12.6 / M5_1629
Col−0 allele
C24 allele
heterozygous
10
5
0
PC2/20%
−10
−8
−6
−4
−2
0
2
PC1/41%

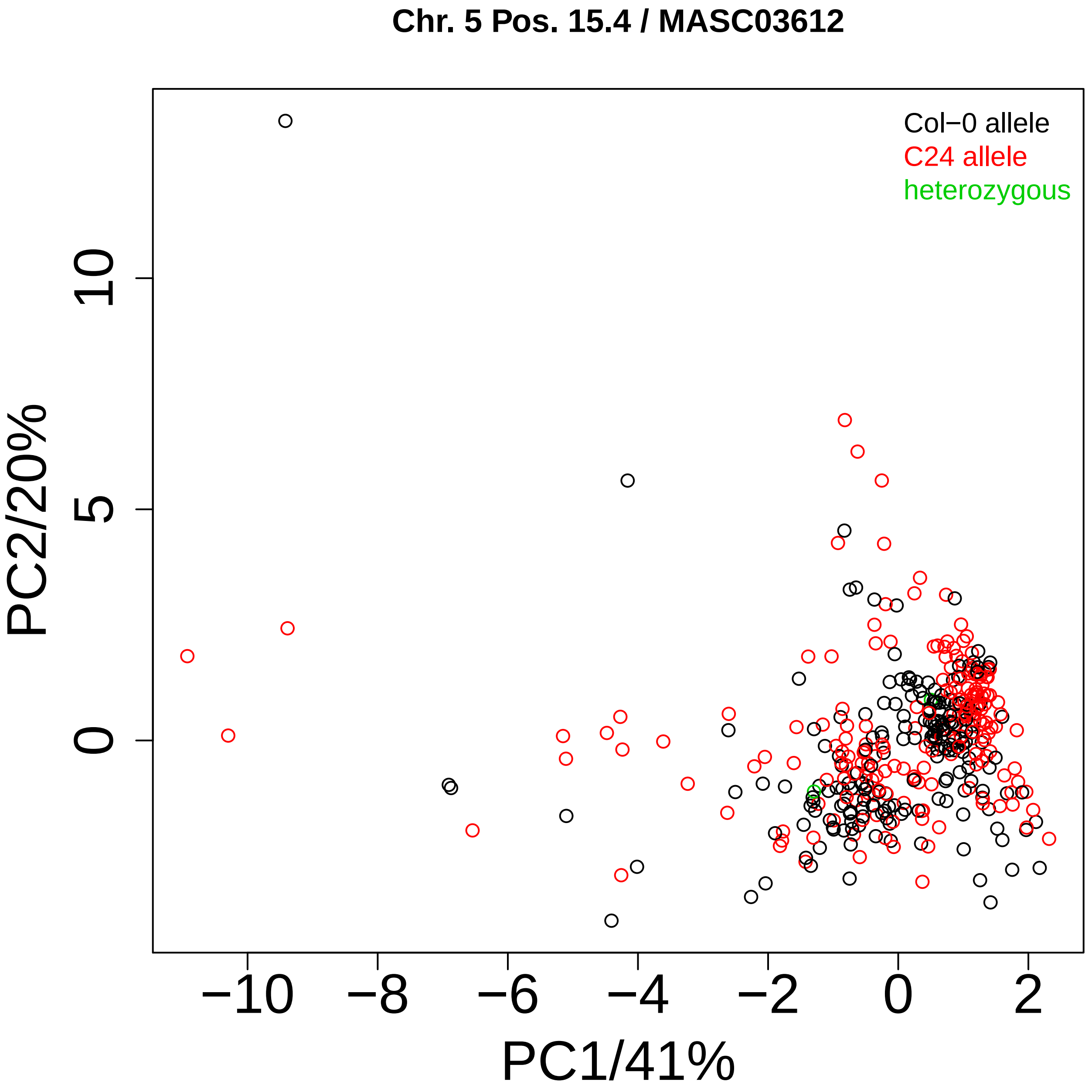

Chr. 5 Pos. 15.4 / MASC03612
Col−0 allele
C24 allele
heterozygous
PC2/20%
PC1/41%
0
5
10
−10
−8
−6
−4
−2
0
2

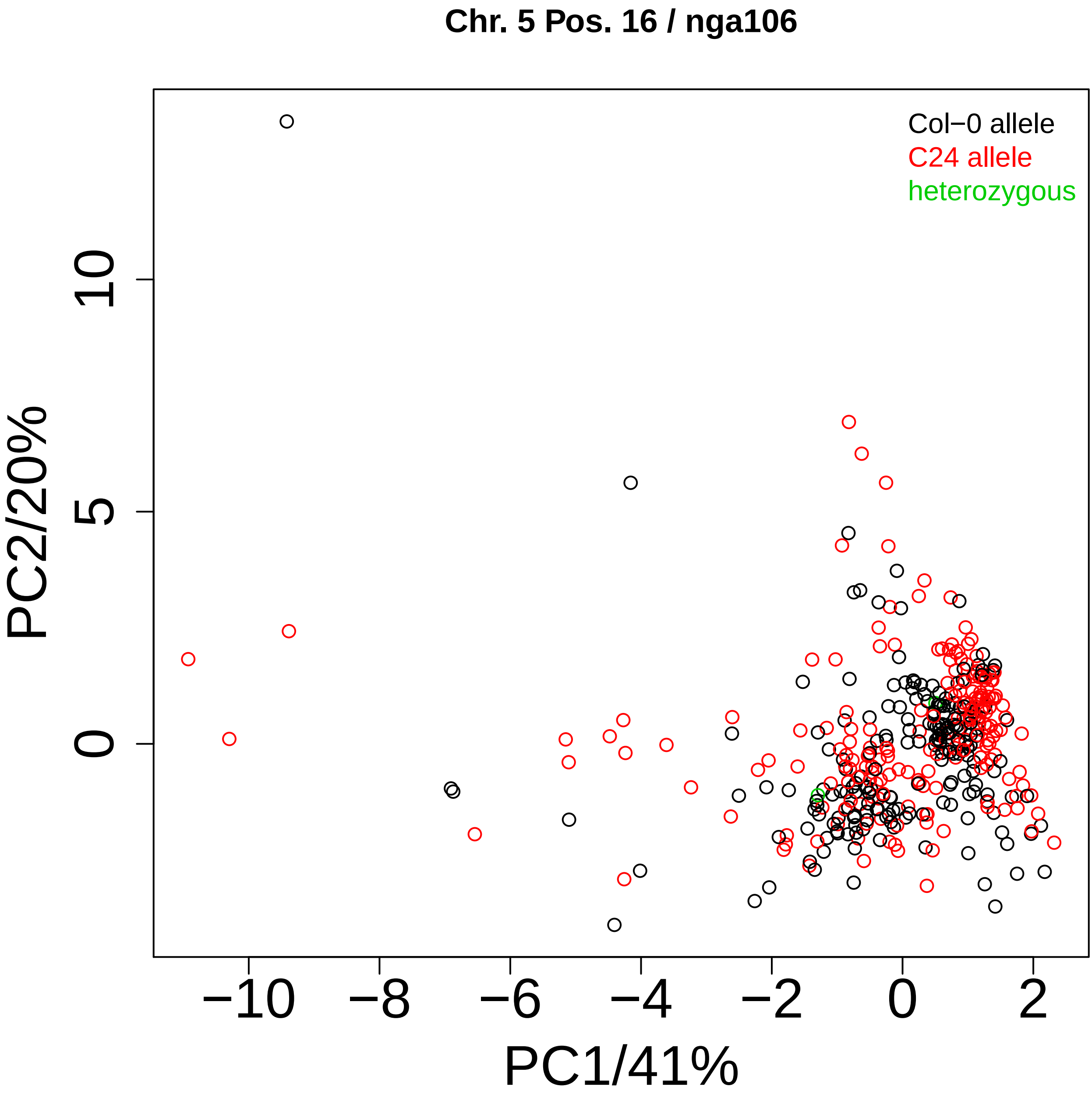

Chr. 5 Pos. 16 / nga106
Col−0 allele
C24 allele
heterozygous
PC2/20%
0
5
10
PC1/41%
−10
−8
−6
−4
−2
0
2

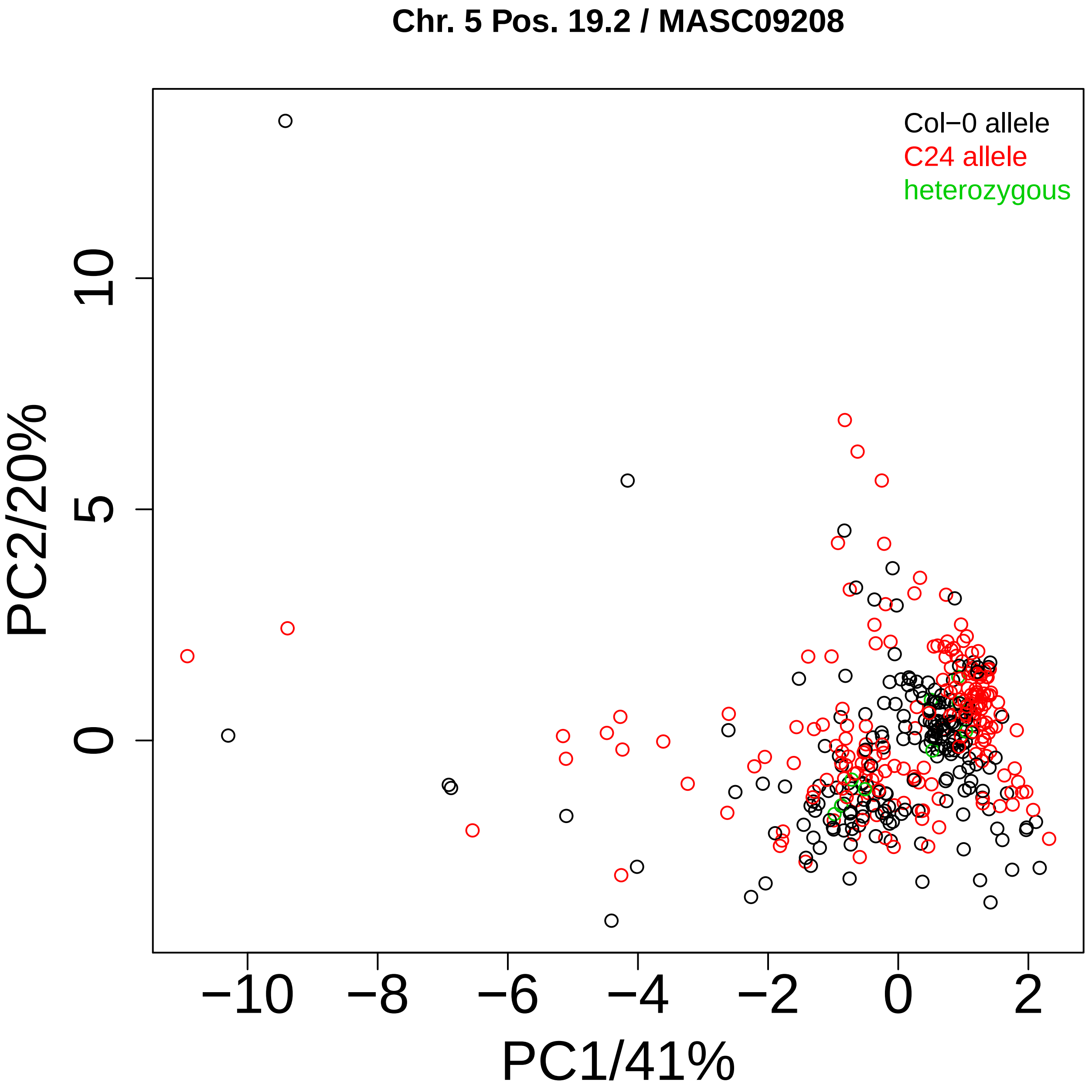
Chr. 5 Pos. 19.2 / MASC09208
Col−0 allele
C24 allele
heterozygous
PC2/20%
0
5
10
PC1/41%
−10
−8
−6
−4
−2
0
2

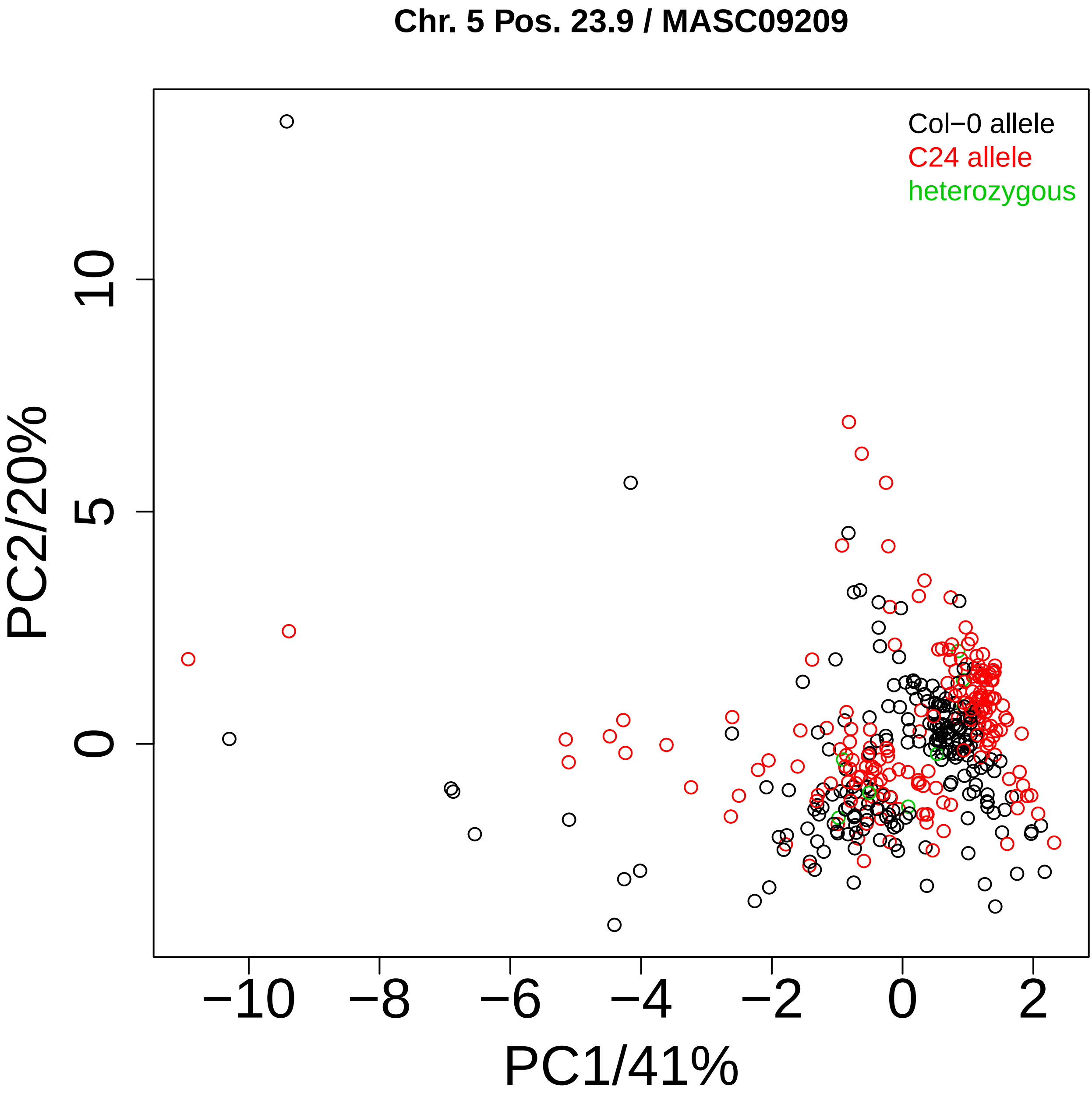
Chr. 5 Pos. 23.9 / MASC09209
Col−0 allele
C24 allele
heterozygous
PC2/20%
0
5
10
PC1/41%
−10
−8
−6
−4
−2
0
2

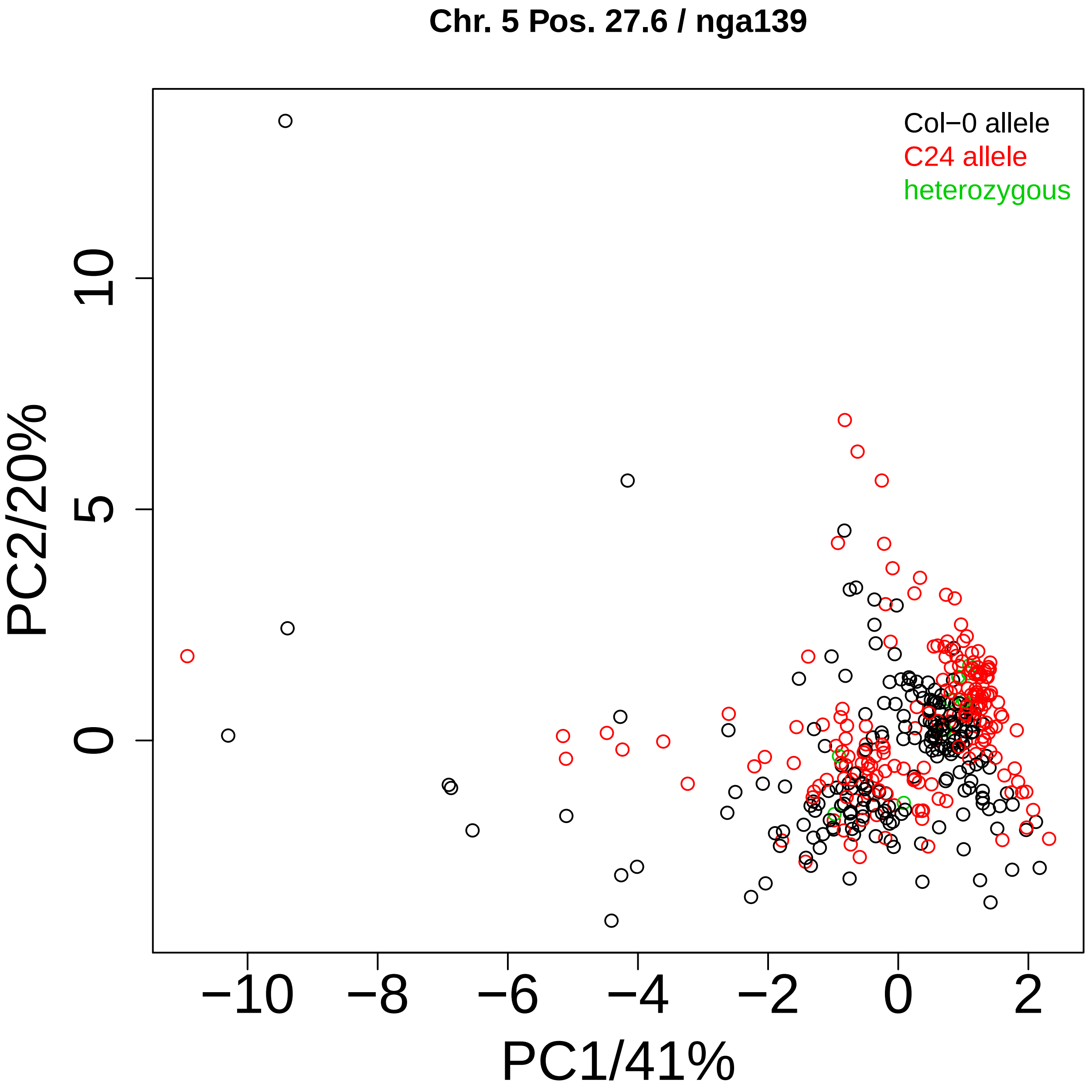
Chr. 5 Pos. 27.6 / nga139
Col−0 allele
C24 allele
heterozygous
PC2/20%
10
5
0
PC1/41%
−10
−8
−6
−4
−2
0
2

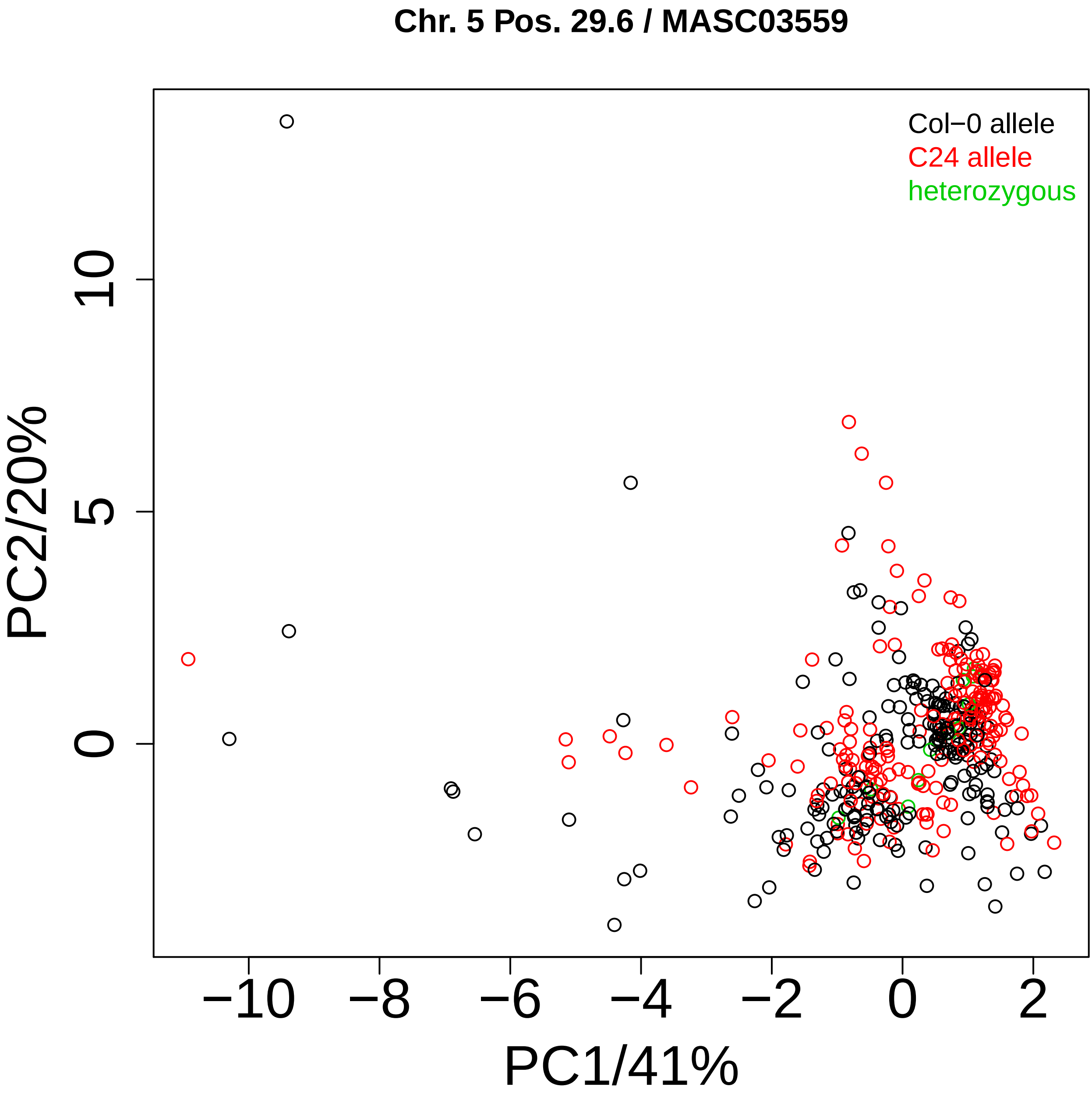

Chr. 5 Pos. 29.6 / MASC03559
Col−0 allele
C24 allele
heterozygous
PC2/20%
10
5
0
PC1/41%
−10
−8
−6
−4
−2
0
2

Chr. 5 Pos. 36.8 / MASC04983

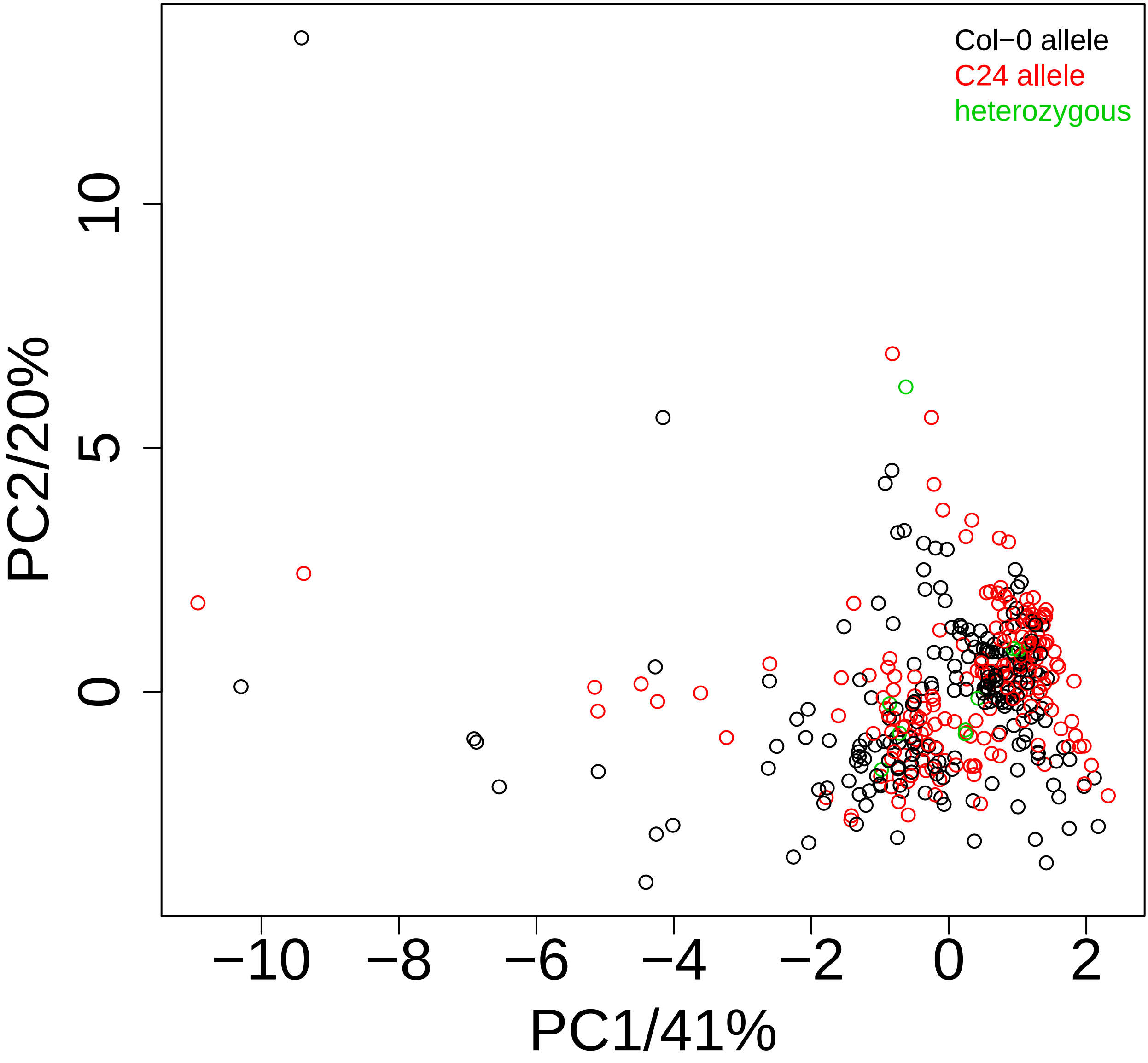

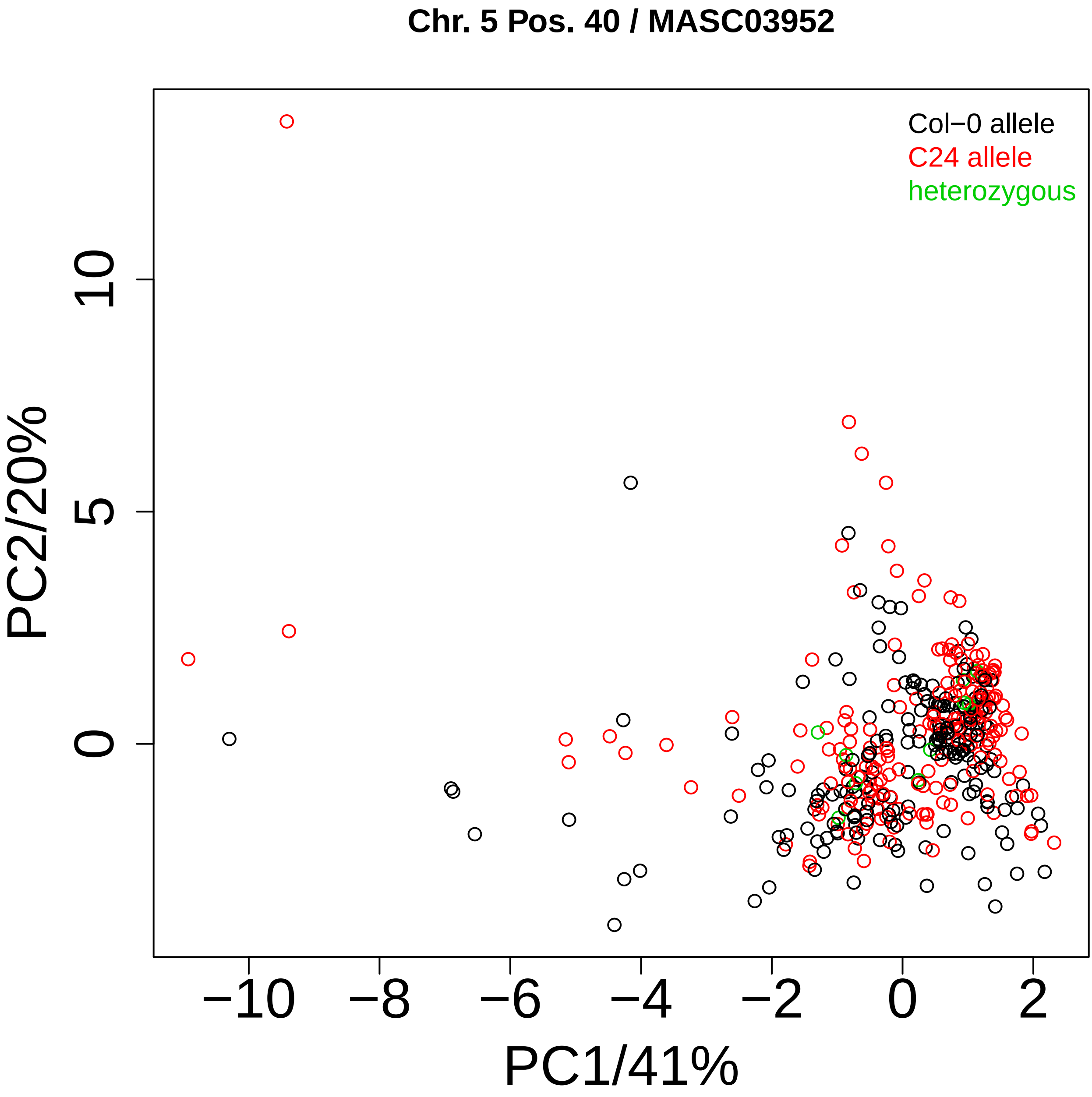
Chr. 5 Pos. 40 / MASC03952
Col−0 allele
C24 allele
heterozygous
PC2/20%
10
5
0
−10
−8
−6
−4
−2
0
2
PC1/41%

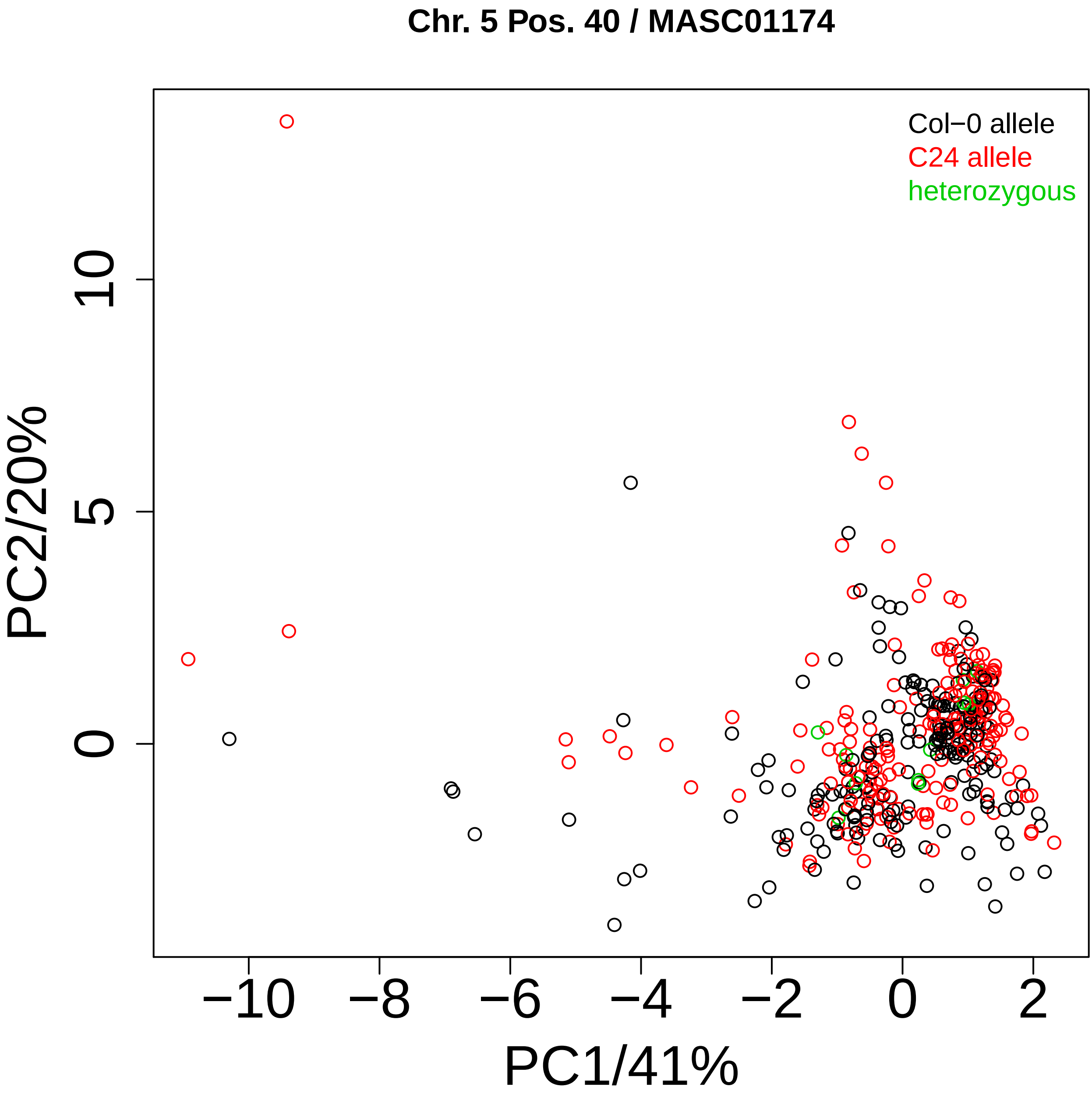

Chr. 5 Pos. 40 / MASC01174
Col−0 allele
C24 allele
heterozygous
PC1/41%
PC2/20%
−10
−8
−6
−4
−2
0
2
0
5
10

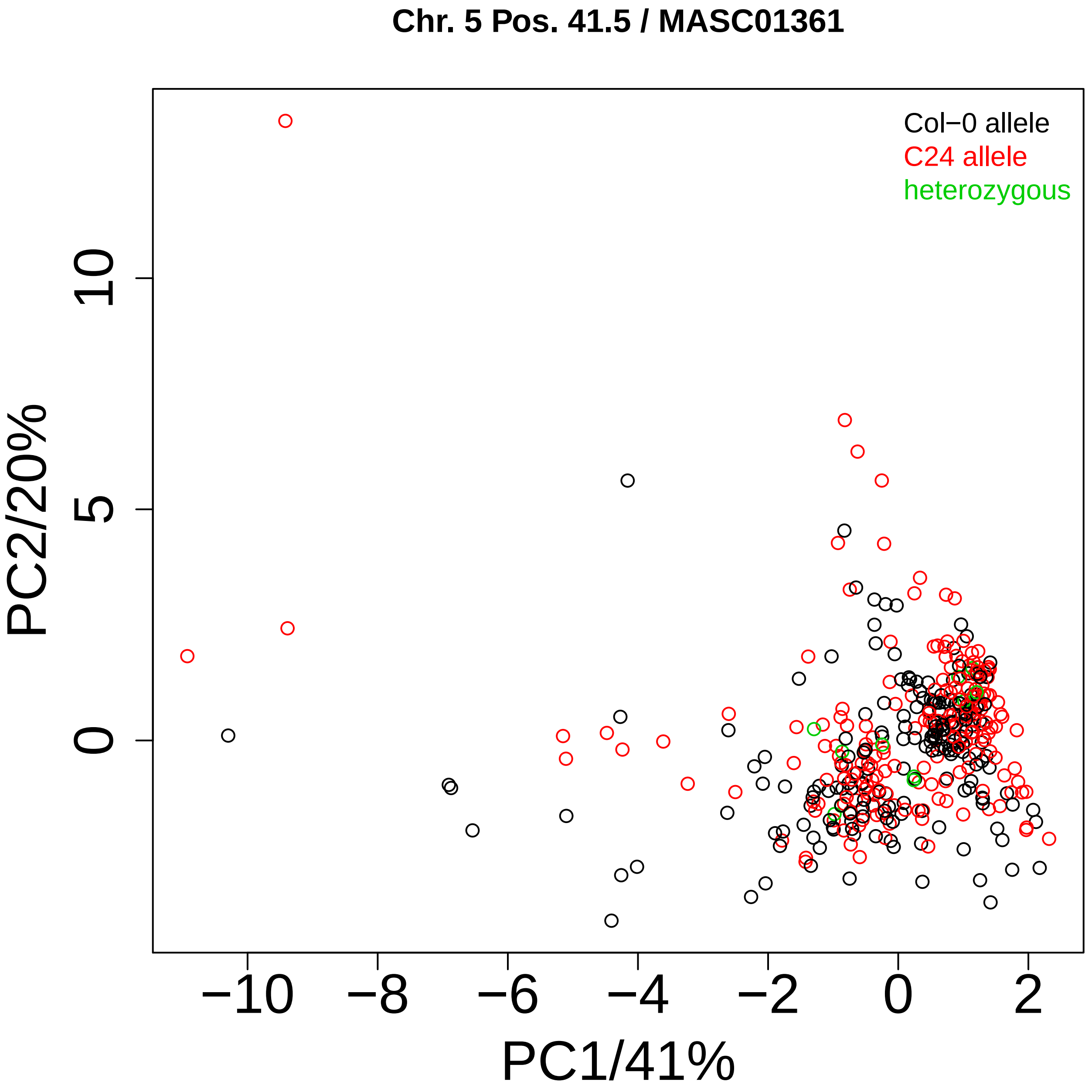
Chr. 5 Pos. 41.5 / MASC01361
Col−0 allele
C24 allele
heterozygous
PC2/20%
0
5
10
PC1/41%
−10
−8
−6
−4
−2
0
2

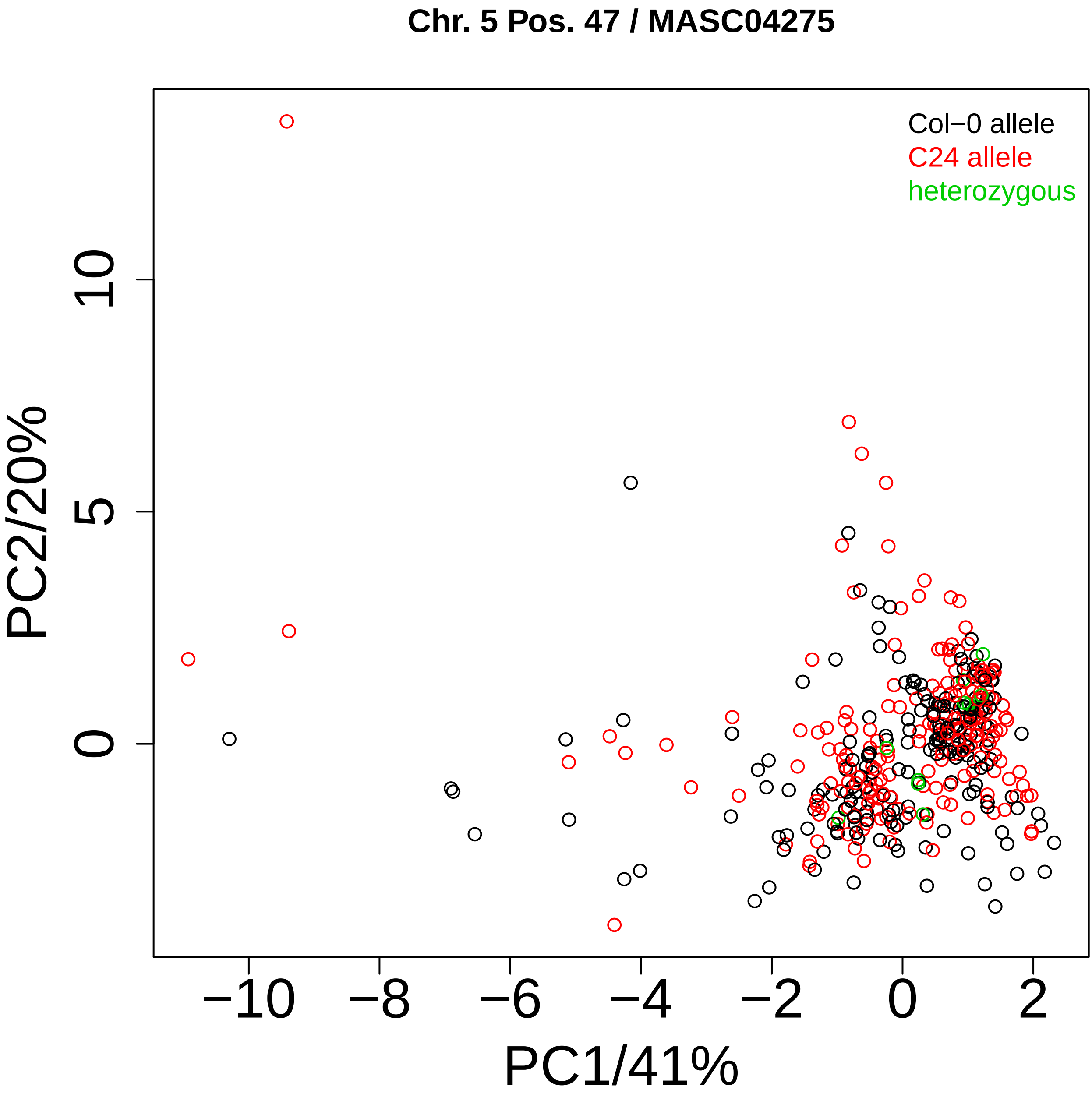
Chr. 5 Pos. 47 / MASC04275
Col−0 allele
C24 allele
heterozygous
PC2/20%
10
5
0
PC1/41%
−10
−8
−6
−4
−2
0
2

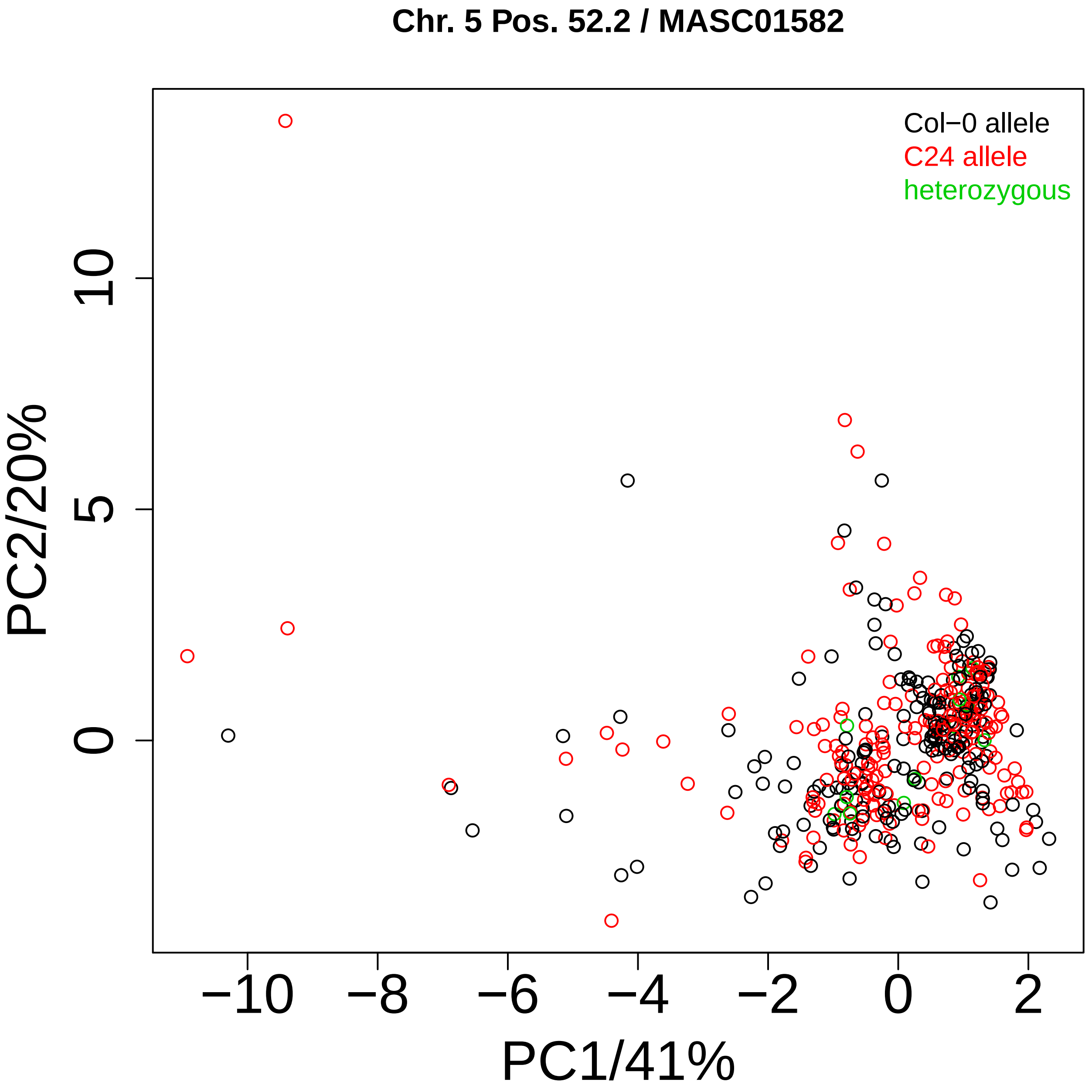
Chr. 5 Pos. 52.2 / MASC01582
Col−0 allele
C24 allele
heterozygous
10
5
0
−10
−8
−6
−4
−2
0
2
PC2/20%
PC1/41%

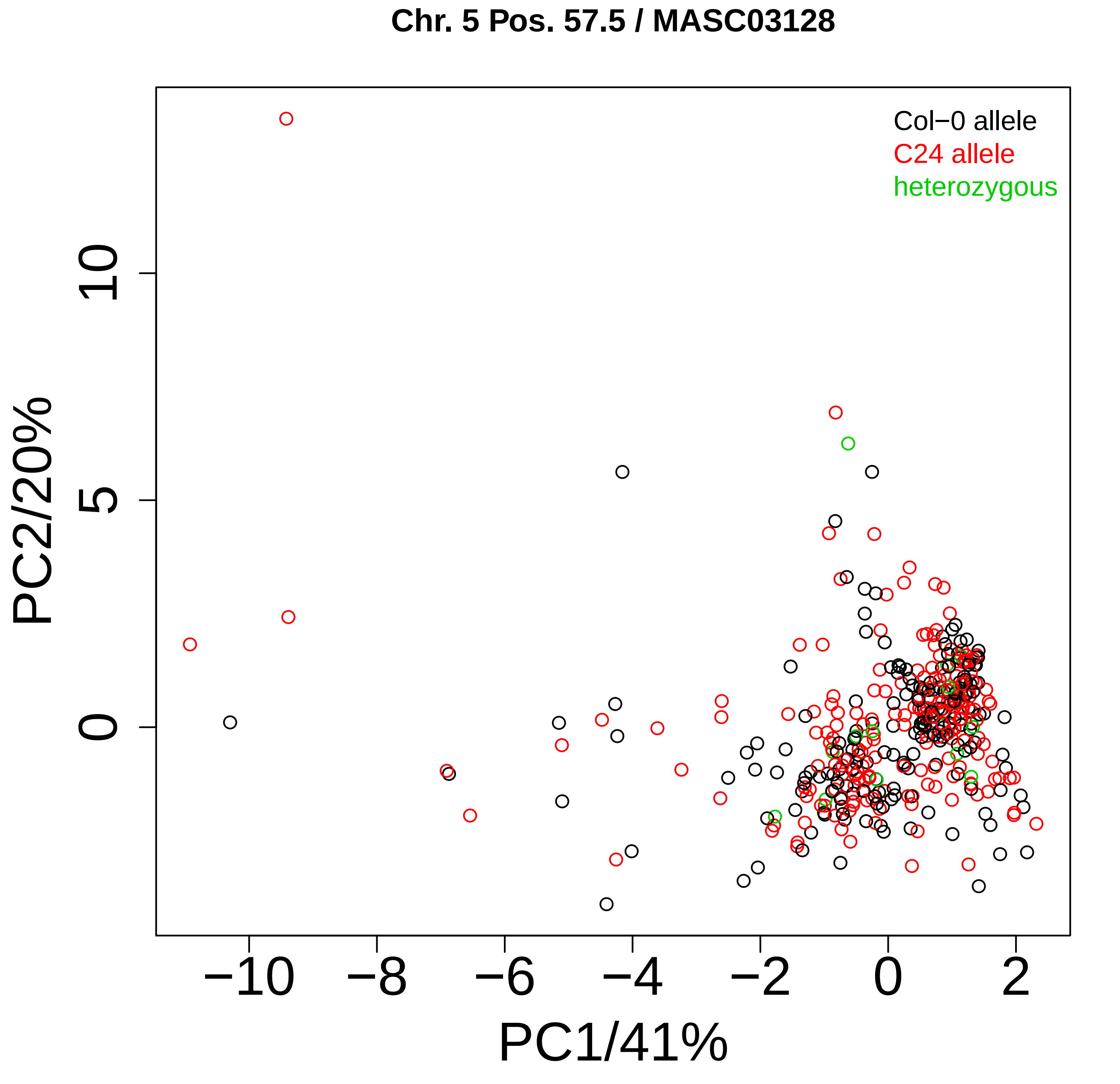
Chr. 5 Pos. 57.5 / MASC03128
Col−0 allele
C24 allele
heterozygous
PC2/20%
PC1/41%
0
5
10
−10
−8
−6
−4
−2
0
2

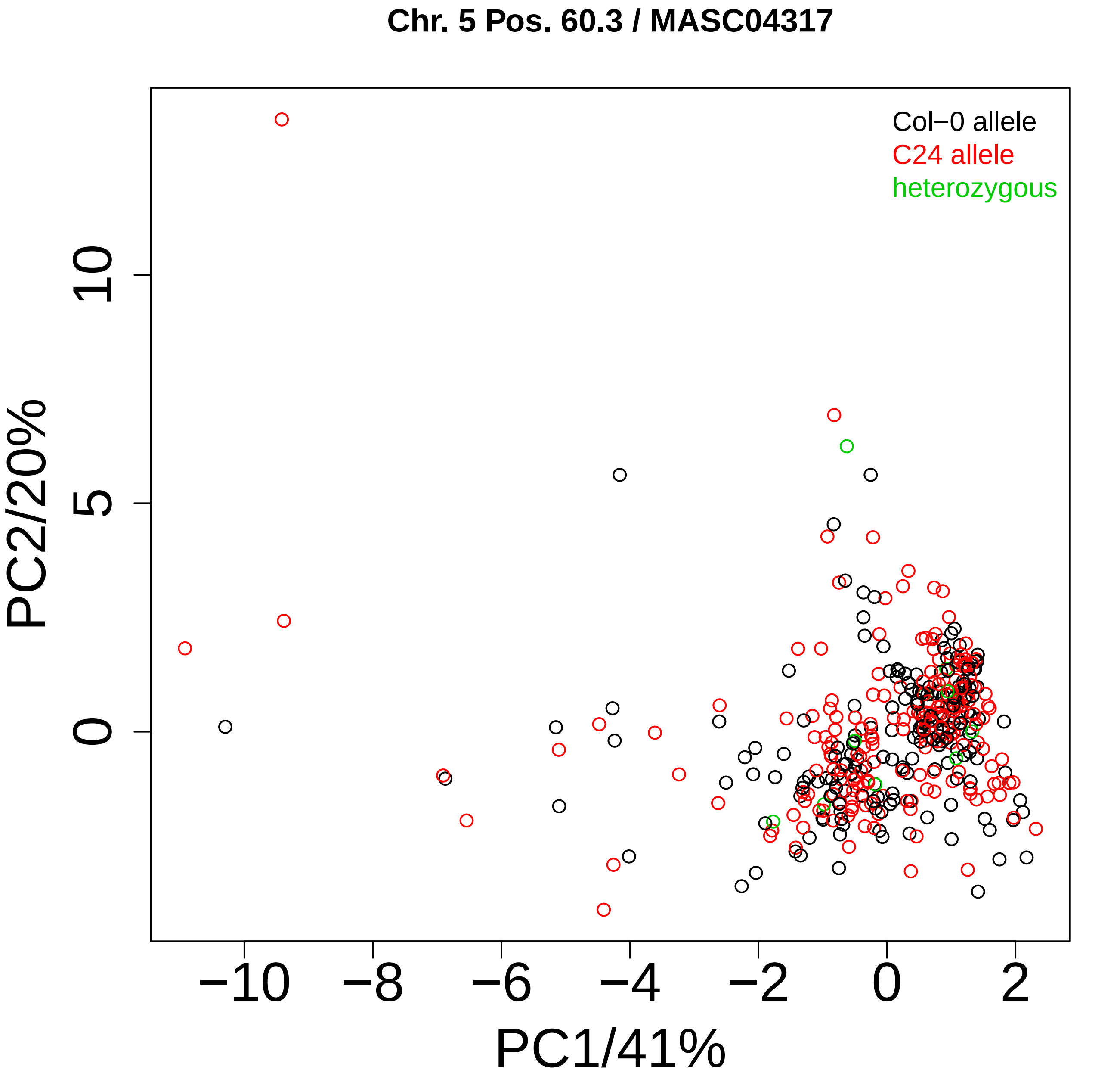
Chr. 5 Pos. 60.3 / MASC04317
Col−0 allele
C24 allele
heterozygous
PC2/20%
0
5
10
PC1/41%
−10
−8
−6
−4
−2
0
2

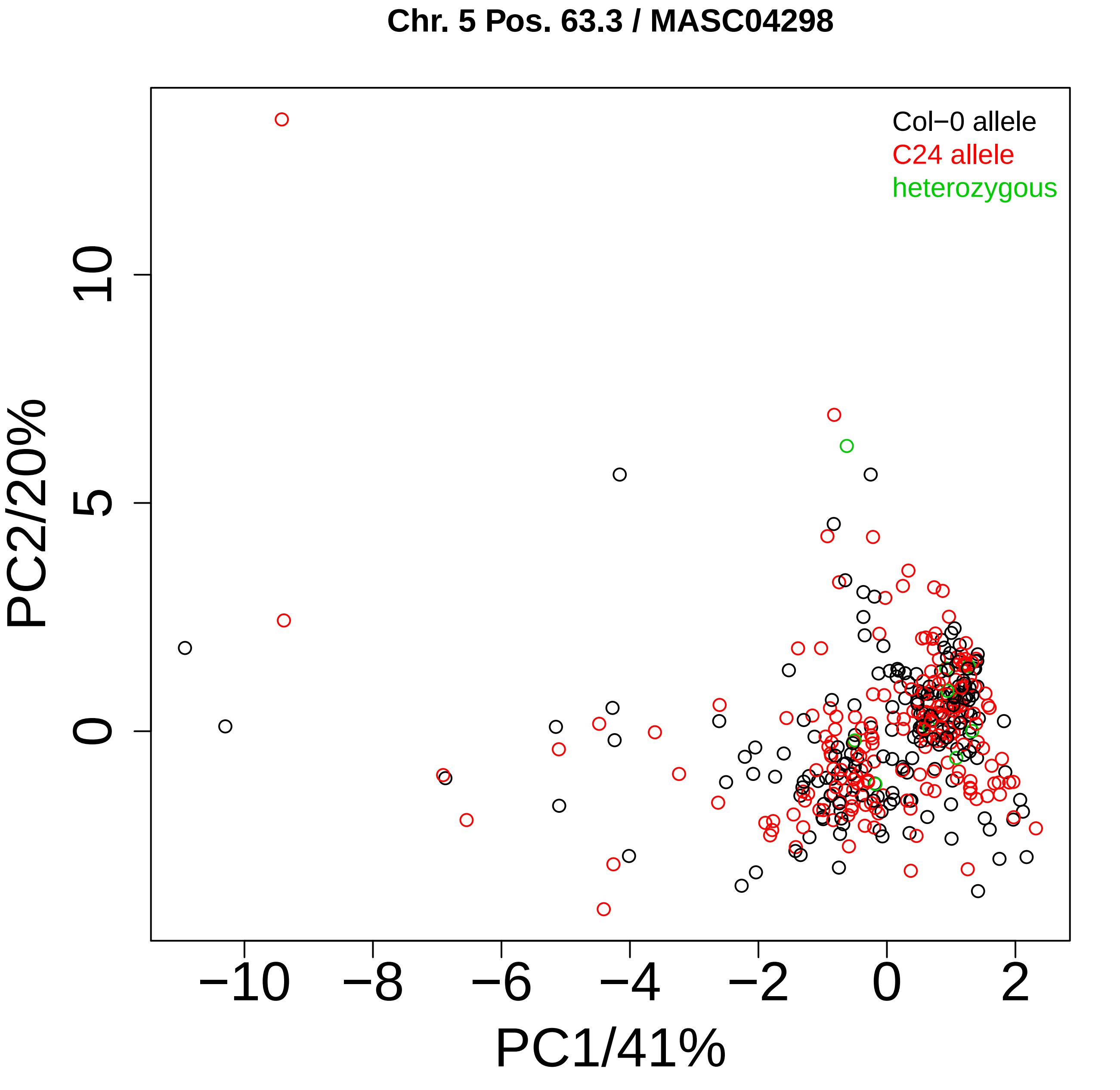

Chr. 5 Pos. 63.3 / MASC04298
Col−0 allele
C24 allele
heterozygous
PC2/20%
PC1/41%
10
5
0
−10
−8
−6
−4
−2
0
2

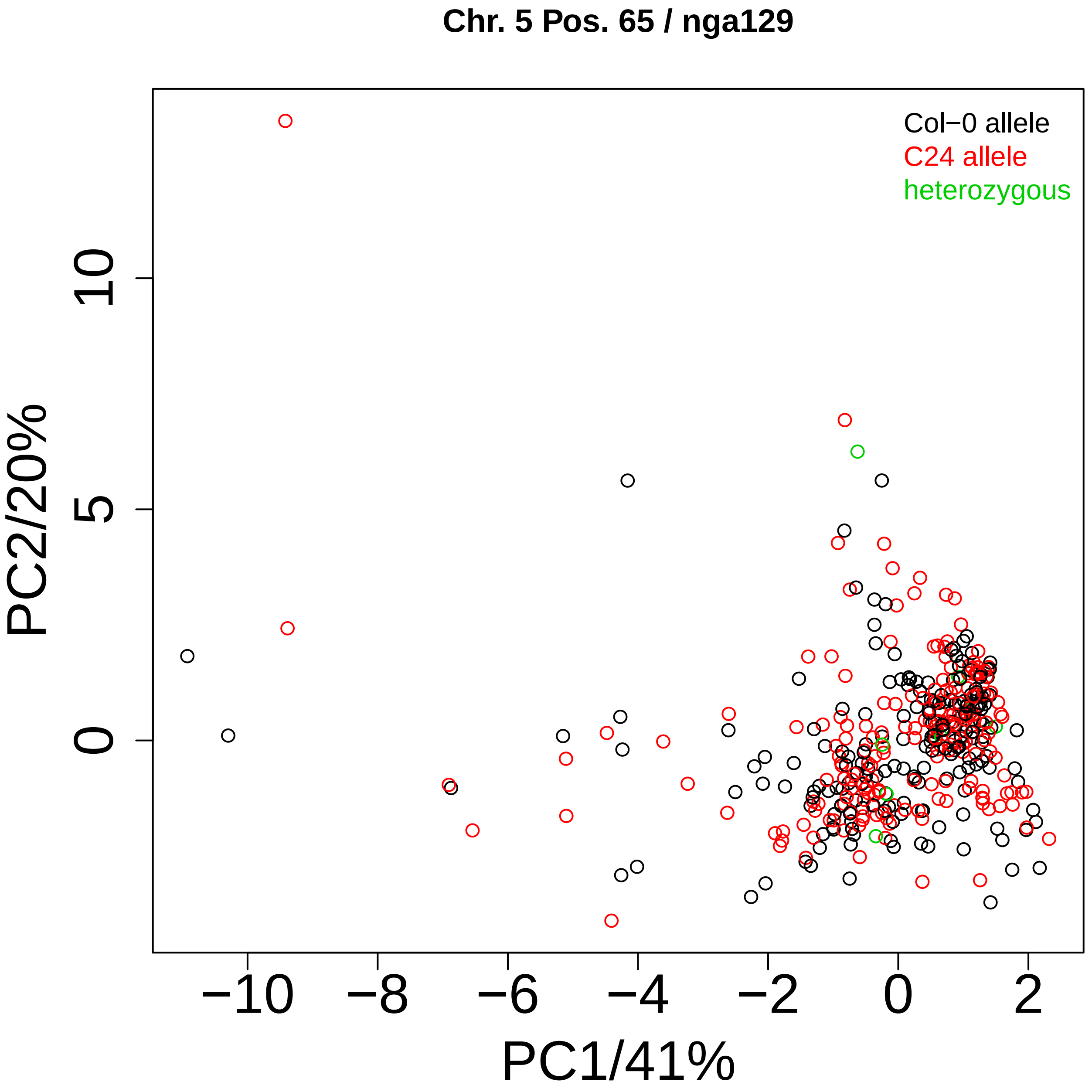

Chr. 5 Pos. 65 / nga129
Col−0 allele
C24 allele
heterozygous
PC2/20%
0
5
10
PC1/41%
−10
−8
−6
−4
−2
0
2

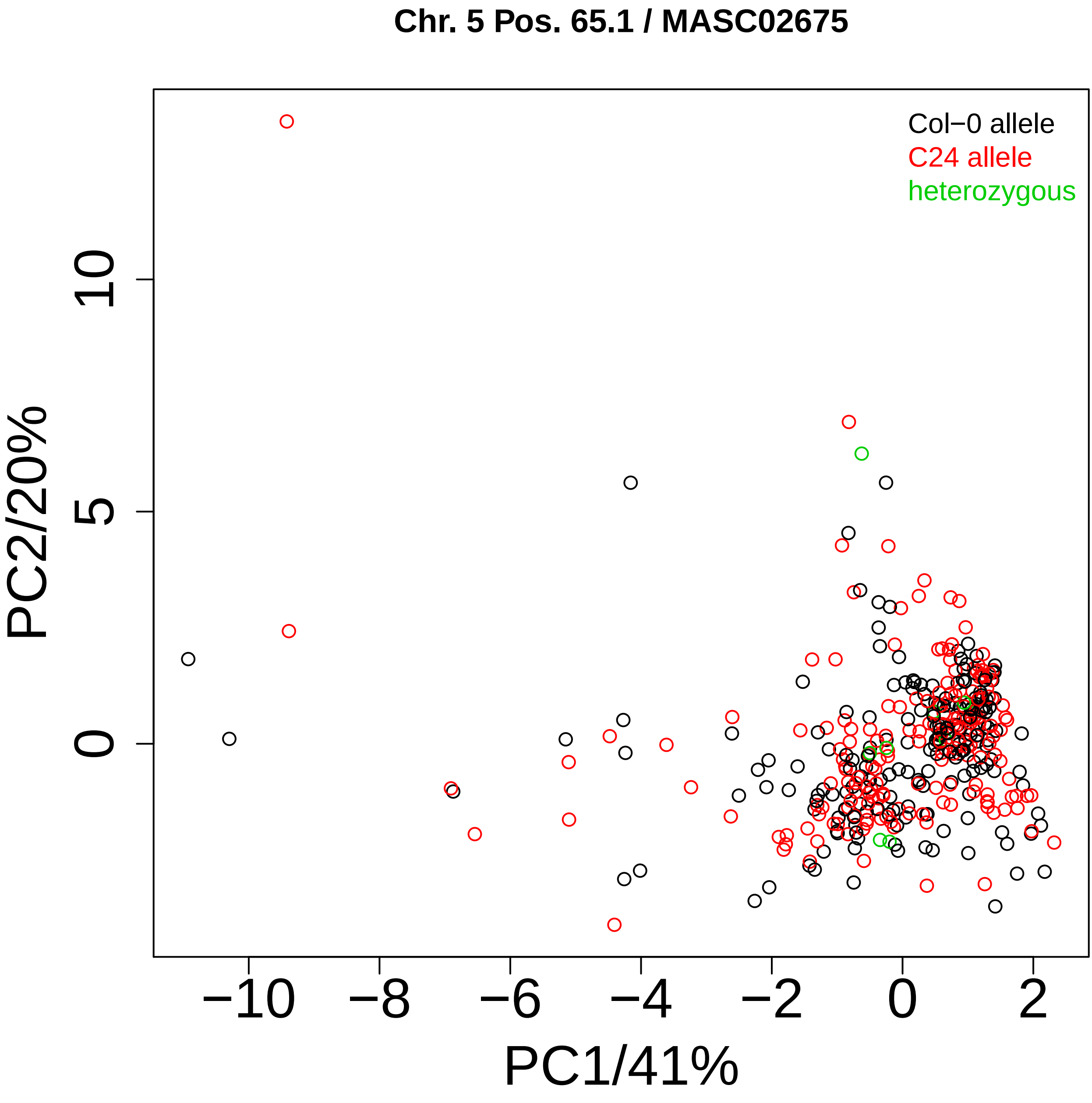

Chr. 5 Pos. 65.1 / MASC02675
Col−0 allele
C24 allele
heterozygous
PC2/20%
10
5
0
−10
−8
−6
−4
−2
0
2
PC1/41%

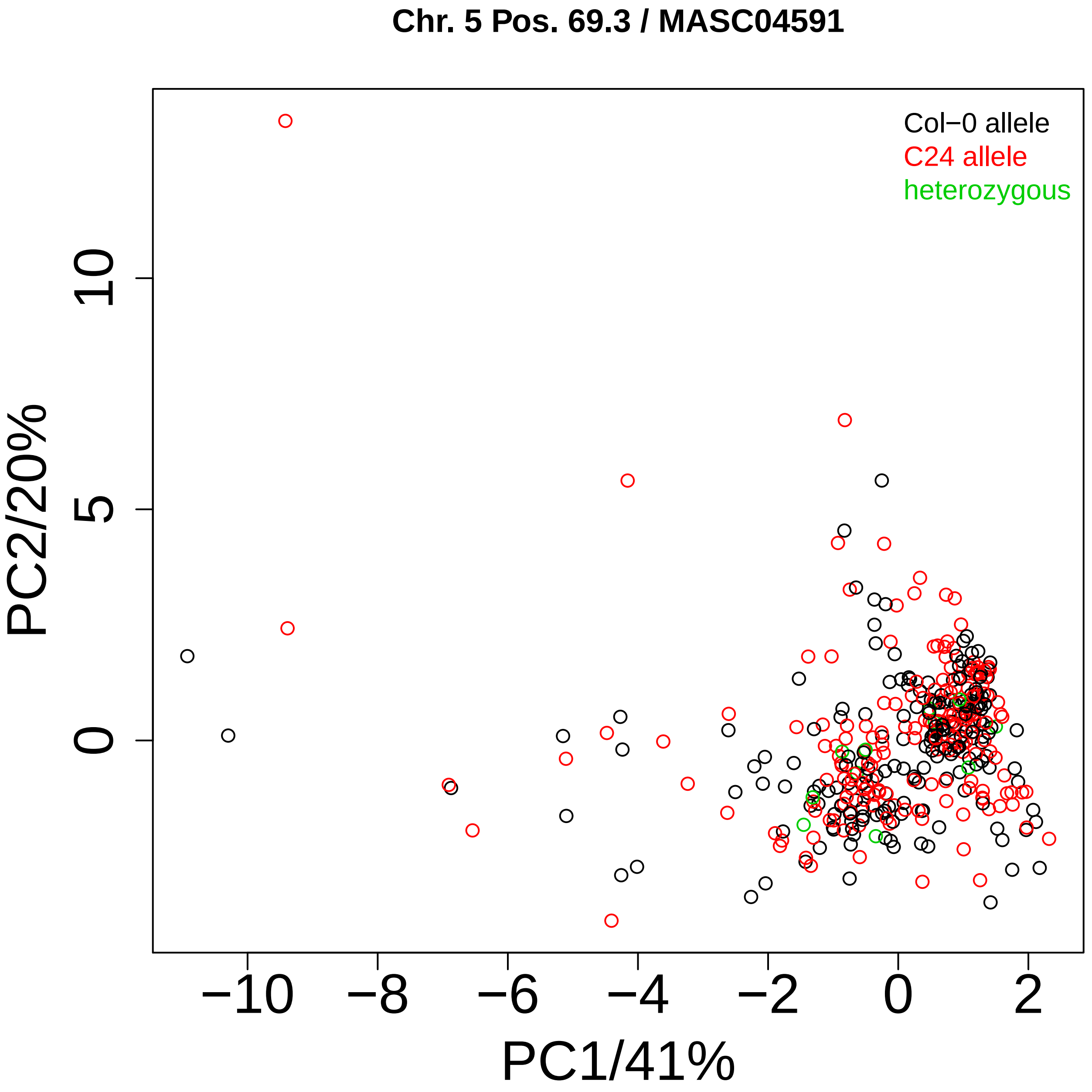
Chr. 5 Pos. 69.3 / MASC04591
Col−0 allele
C24 allele
heterozygous
PC2/20%
0
5
10
PC1/41%
−10
−8
−6
−4
−2
0
2

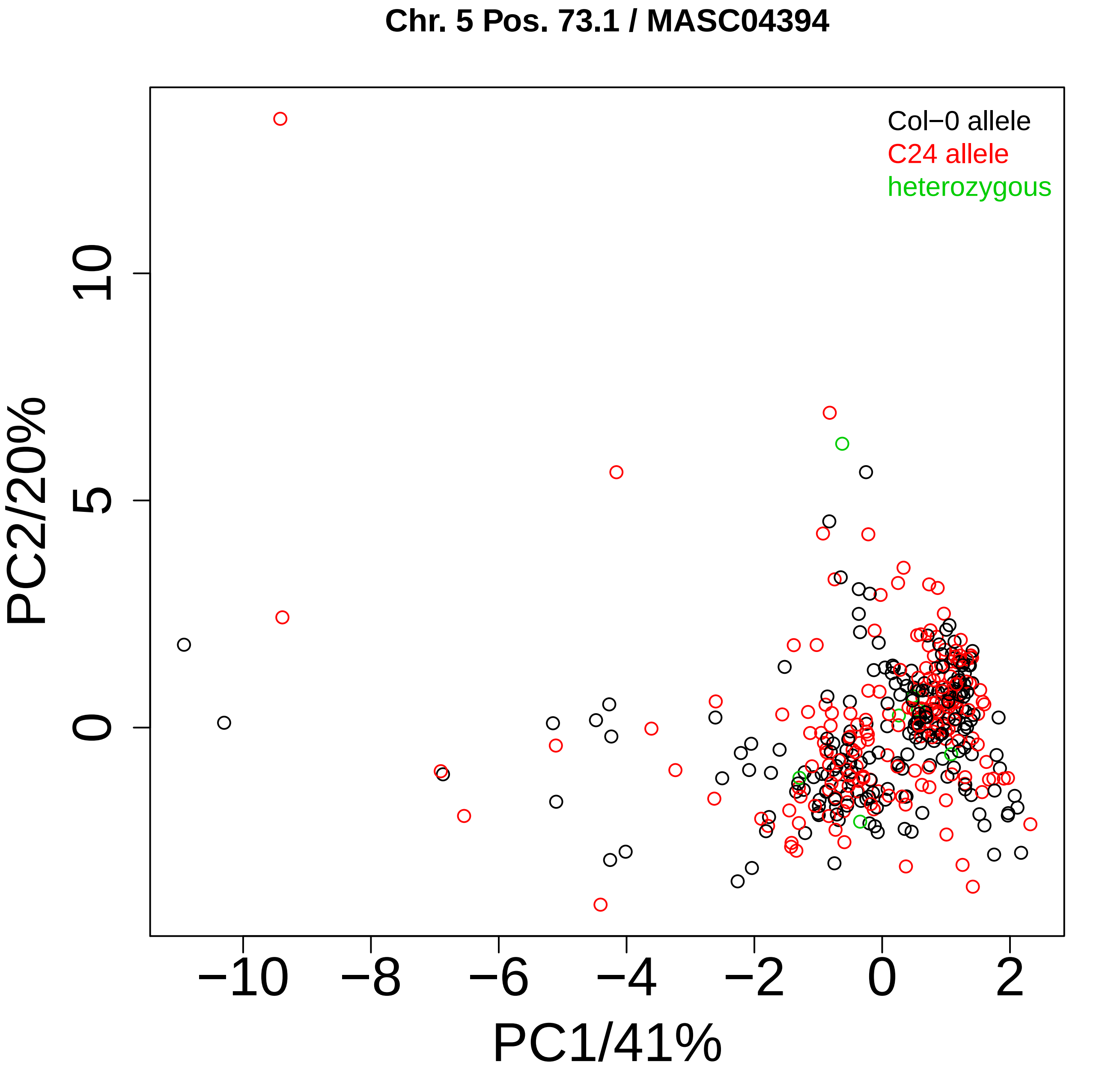
Chr. 5 Pos. 73.1 / MASC04394
Col−0 allele
C24 allele
heterozygous
PC2/20%
PC1/41%
10
5
0
−10
−8
−6
−4
−2
0
2

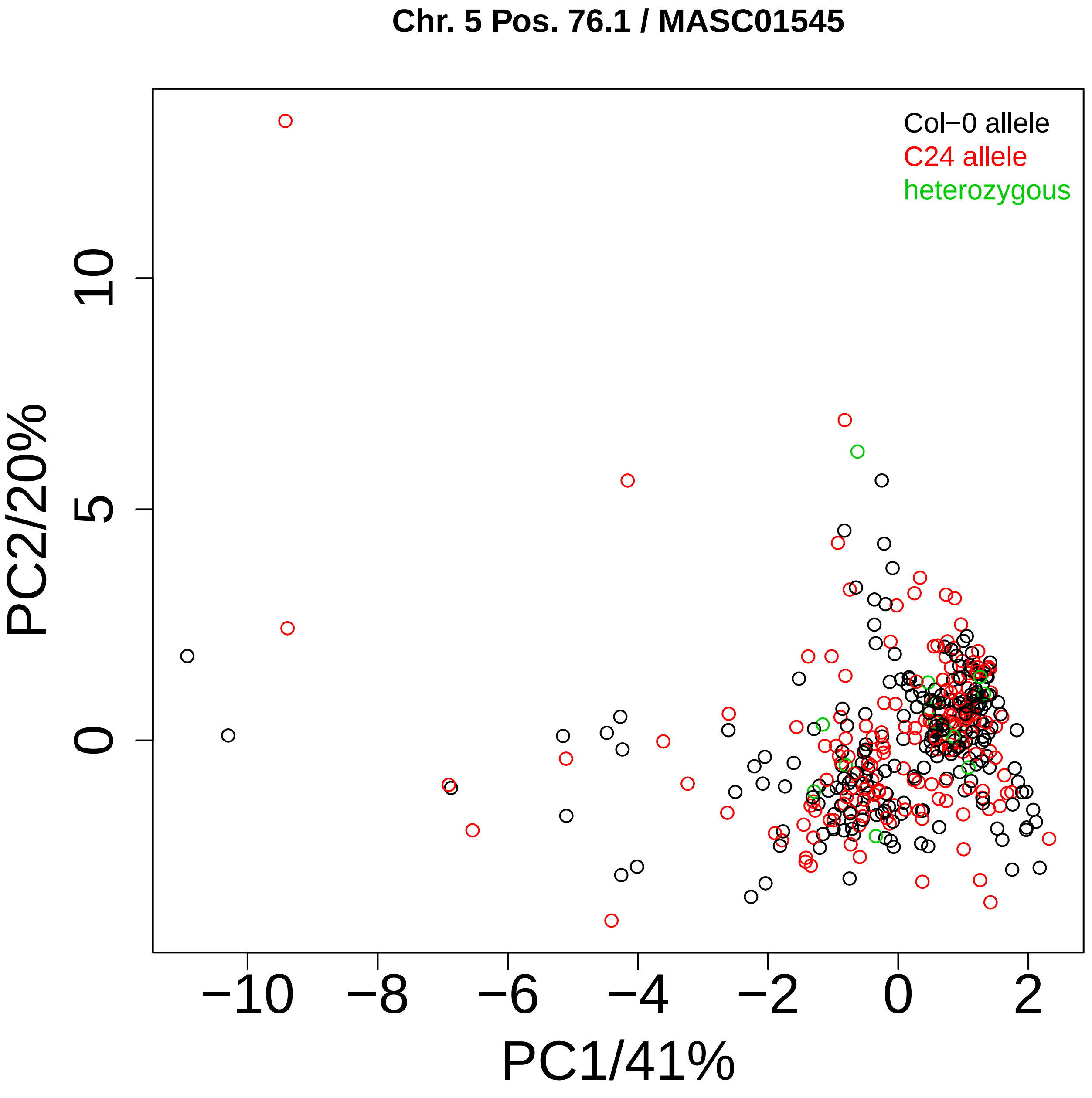
Chr. 5 Pos. 76.1 / MASC01545
Col−0 allele
C24 allele
heterozygous
PC2/20%
PC1/41%
10
5
0
−10
−8
−6
−4
−2
0
2

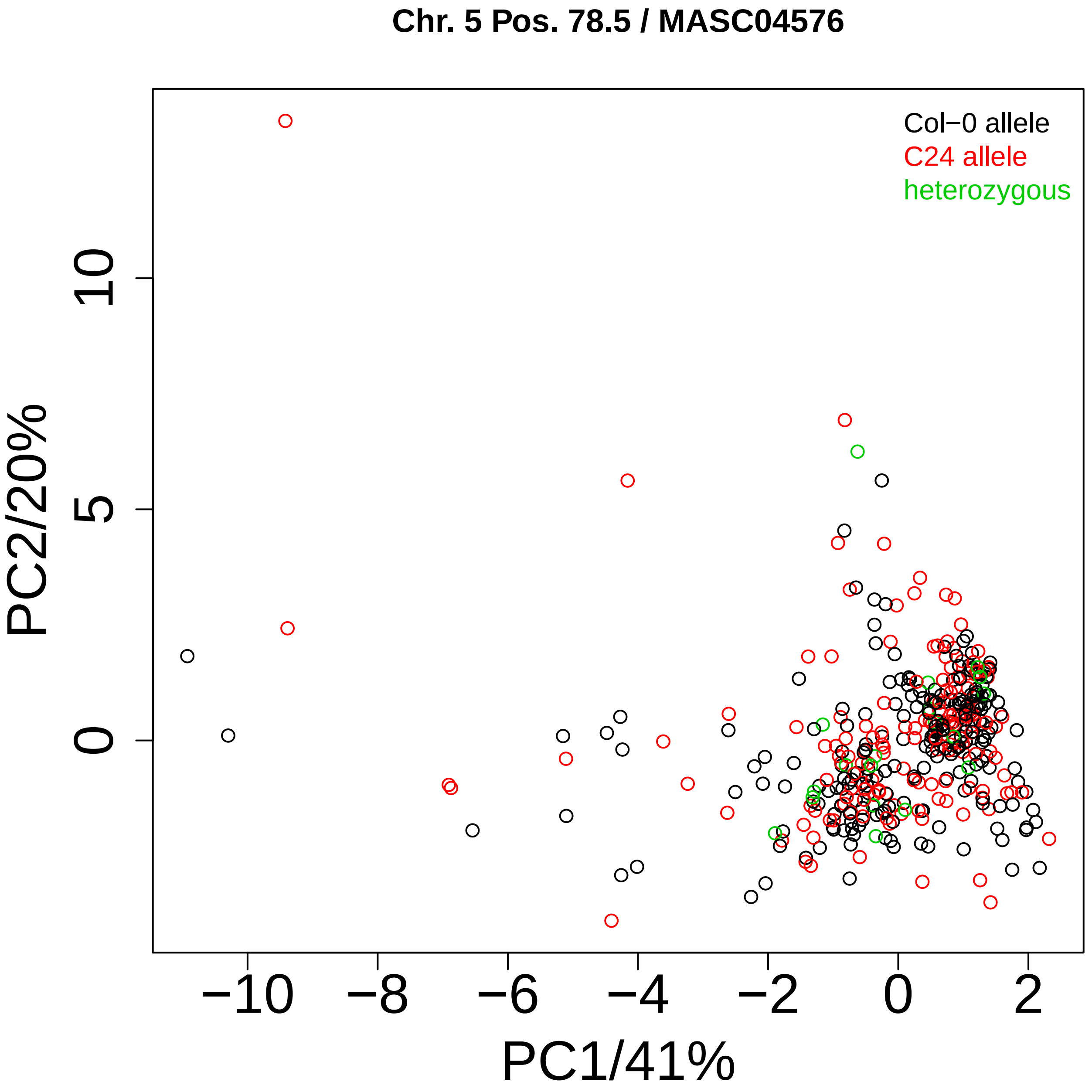
Chr. 5 Pos. 78.5 / MASC04576
Col−0 allele
C24 allele
heterozygous
PC2/20%
PC1/41%
10
5
0
−10
−8
−6
−4
−2
0
2

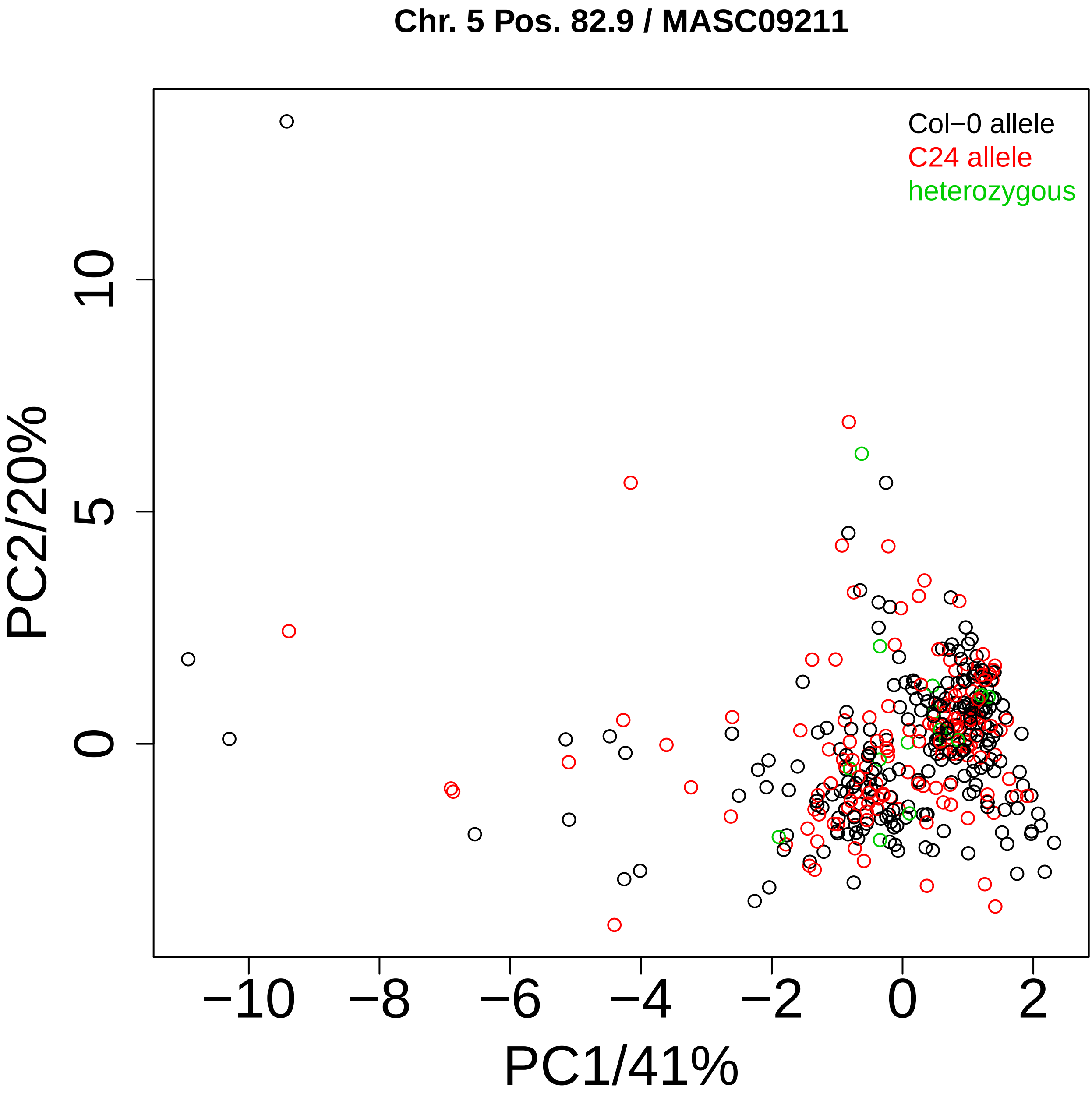
Chr. 5 Pos. 82.9 / MASC09211
Col−0 allele
C24 allele
heterozygous
PC2/20%
PC1/41%
10
5
0
−10
−8
−6
−4
−2
0
2

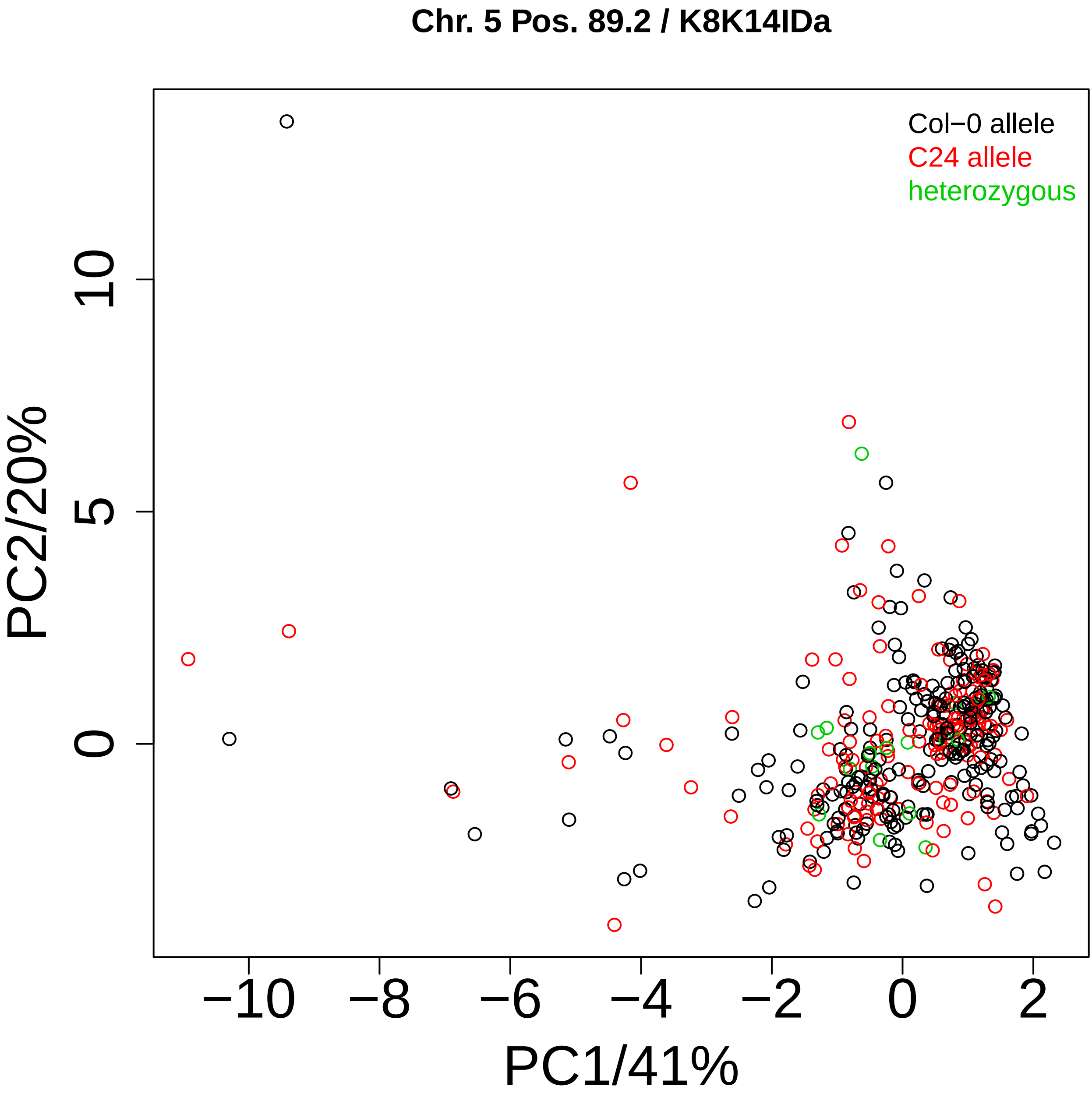

Chr. 5 Pos. 89.2 / K8K14IDa
Col−0 allele
C24 allele
heterozygous
PC2/20%
0
5
10
PC1/41%
−10
−8
−6
−4
−2
0
2

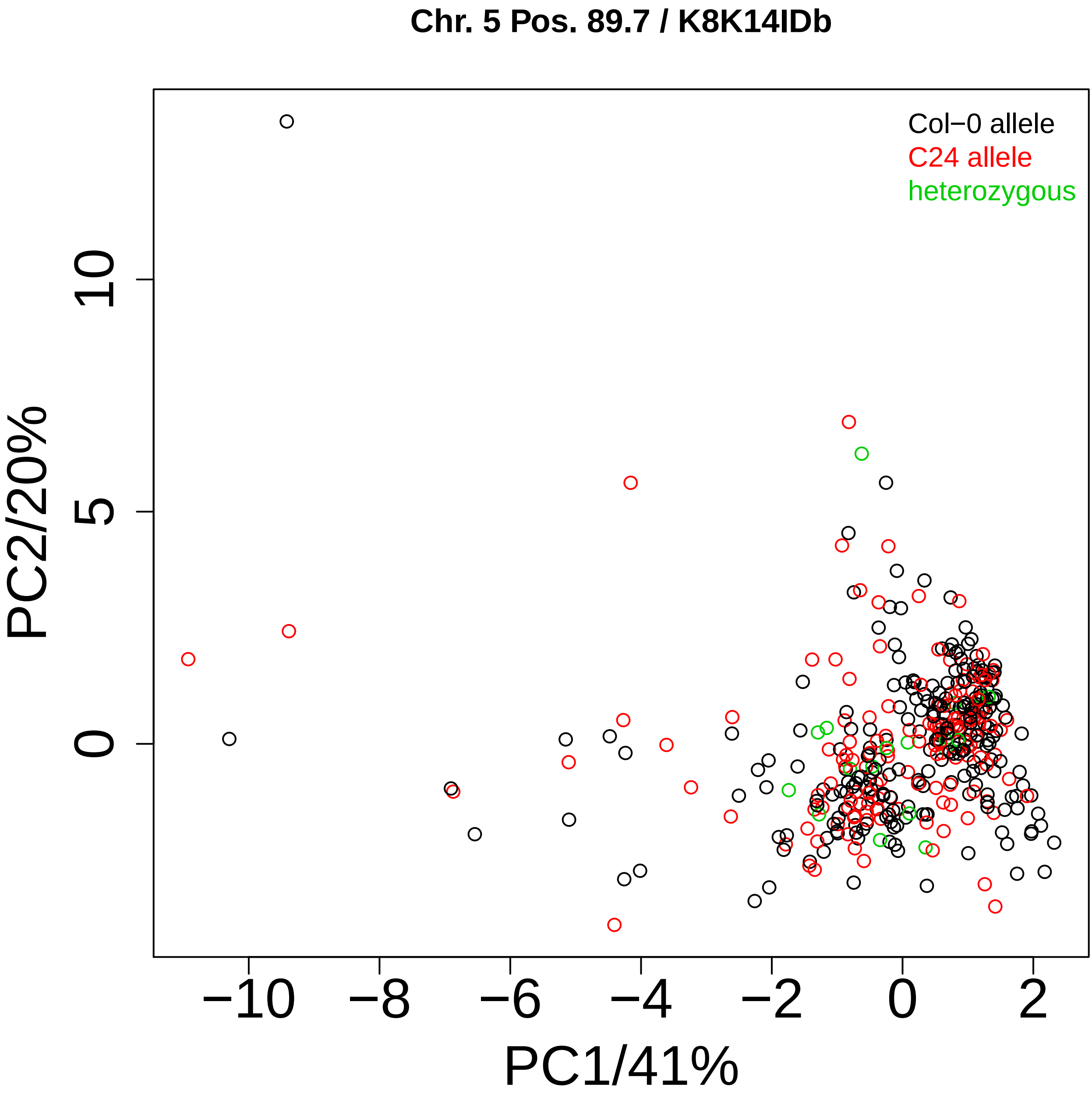
Chr. 5 Pos. 89.7 / K8K14IDb
Col−0 allele
C24 allele
heterozygous
PC2/20%
0
5
10
PC1/41%
−10
−8
−6
−4
−2
0
2
