# Supplementary material for: Genetic dissection of metabolite variation in Arabidopsis seeds: evidence for mQTL hotspots and a master regulatory locus of seed metabolism
Source: J Exp Bot. 2017 Mar 6;68(7):1655–67. doi: 10.1093/jxb/erx049 (PMC5444479; doi:10.1093/jxb/erx049)
Supplement: Supplementary_Figures_S1_S4 [file erx049_suppl_Supplementary_Figures_S1_S4.pdf]

Genetic dissection of metabolite variation in Arabidopsis seeds: evidence for mQTL hotspots and a master regulatory locus of seed metabolism

Dominic Knoch, David Riewe, Rhonda Christiane Meyer, Anastassia Boudichevskaia, Renate Schmidt and Thomas Altmann

SUPPLEMENTARY FIGURES

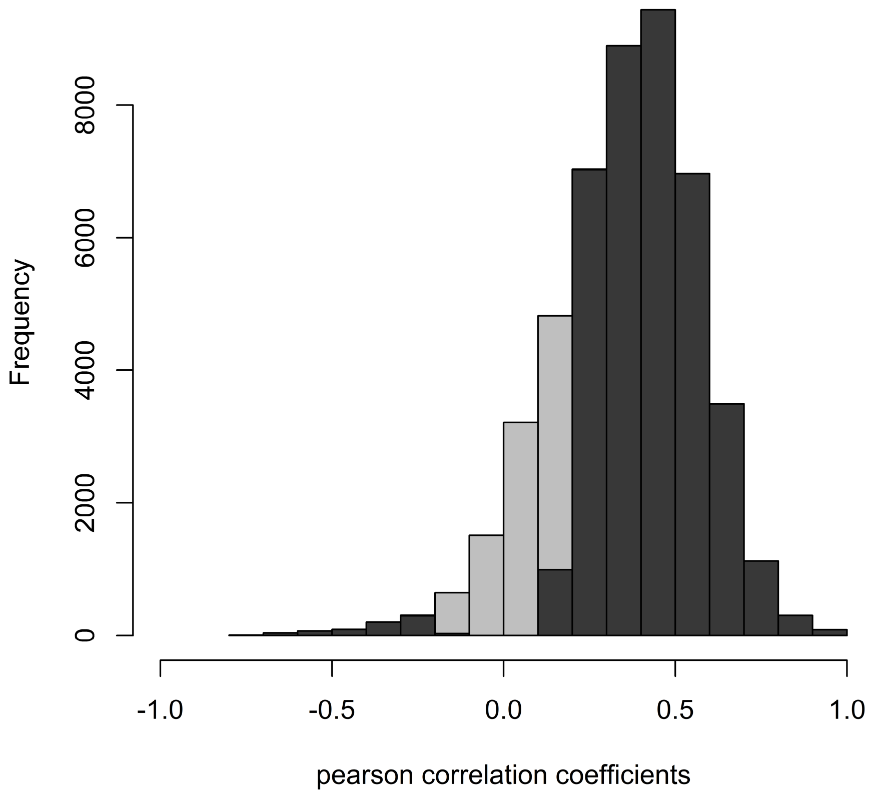

Figure S1. Histogram of metabolite correlations

Pairwise Pearson product moment correlation coefficients for all 311 detected metabolites were plotted against their frequency: all correlations (dark grey, n = 49,770) and statistically significant correlations (light grey, n = 40,261). Pearson correlations with a p-value lower than 0.05 (after FDR multiple testing correction) are regarded as statistically significant.

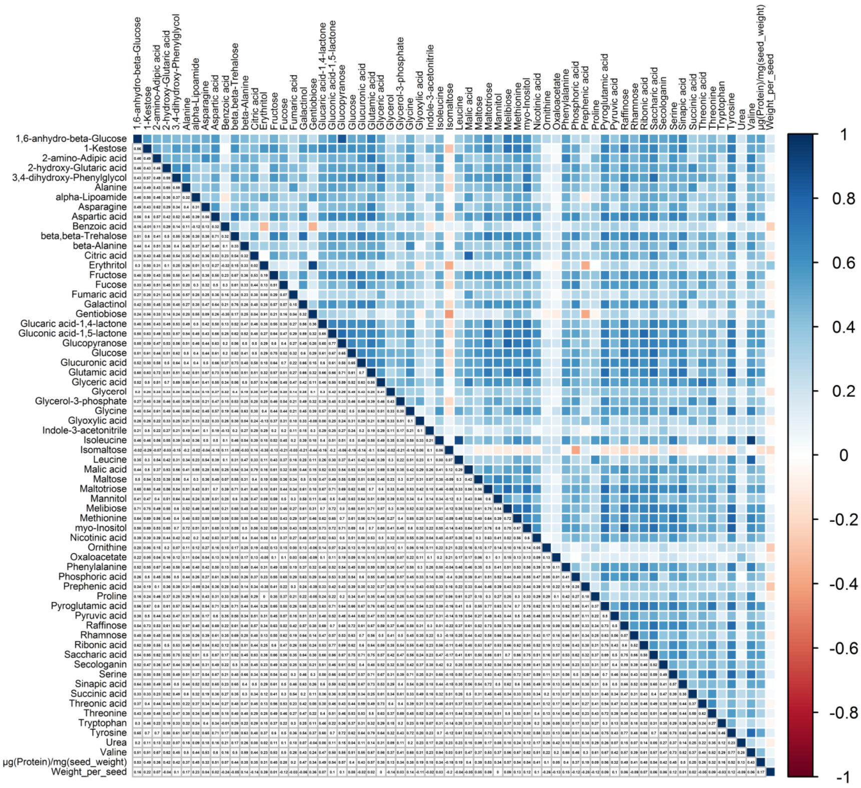

Figure S2. Correlation matrix

Correlation matrix of 64 metabolites of known structure, seed weight and total seed protein content. The upper right triangle represents color-coded Pearson product moment correlation coefficients between two traits (positive and negative correlations in blue and red, respectively). The lower left triangle shows the Pearson product moment correlation coefficients as numeric values.

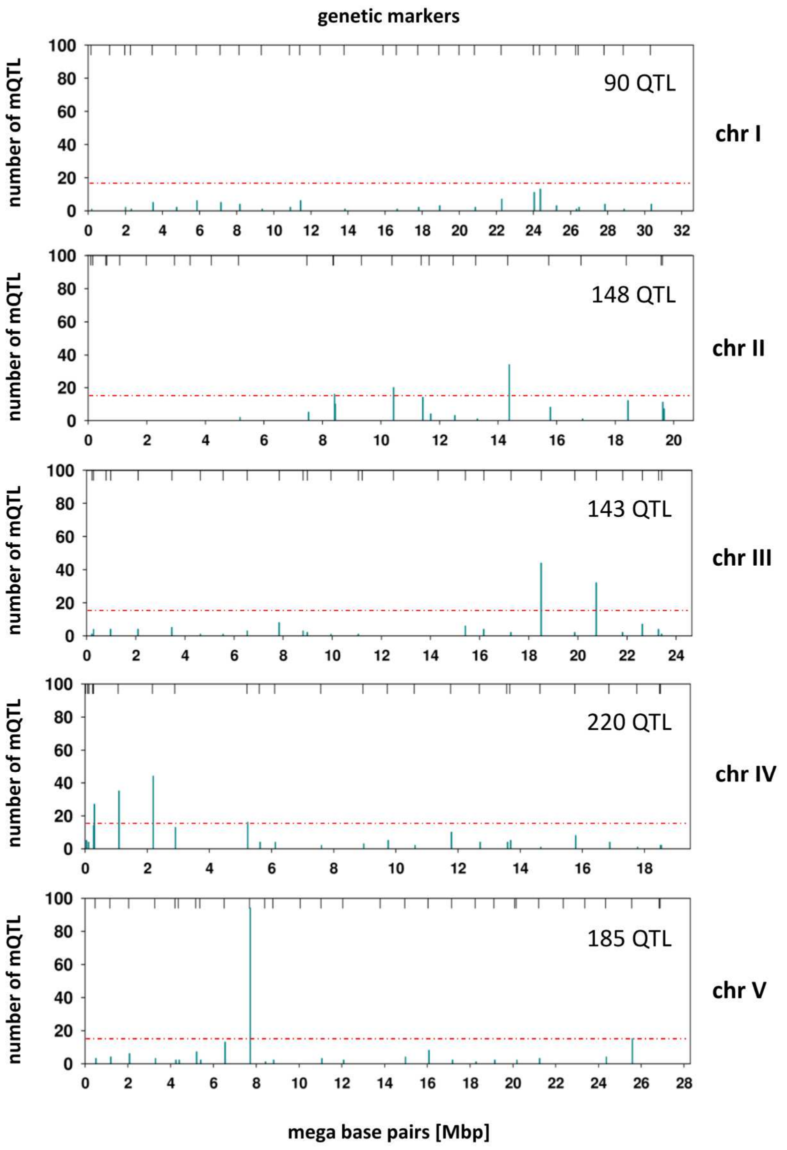

Figure S3. Overview of mQTL hotspots

For each mQTL the nearest genetic marker to the apex of the LOD profile was determined and the number of associated mQTL per marker was summed up and plotted against the physical location of the markers (cyan bars). The ticks above the plots indicate the locations of the genetic markers. Red dashed lines correspond to the mQTL hotspot threshold (n = 15) based on 10,000 permutations. The total numbers of detected mQTL per chromosome are shown in the upper right corner of each plot.

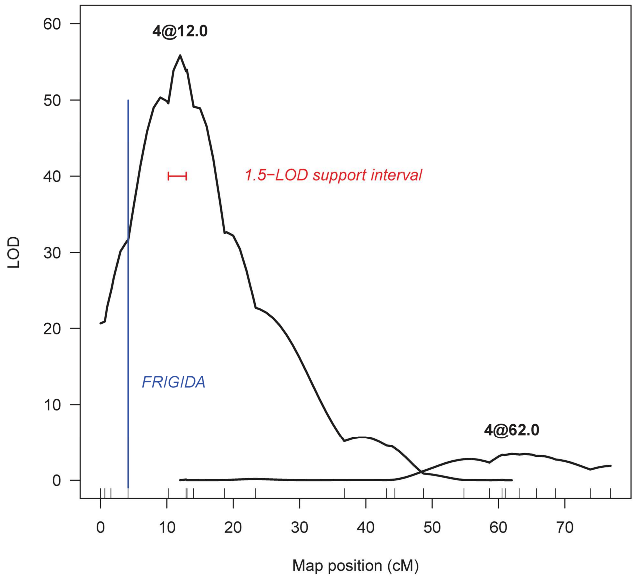

Figure S4. LOD profile of PC1 (chromosome IV)

The LOD profile for PC1 was plotted for chromosome IV. The solid black lines indicate LOD profiles calculated with the ‘stepwiseqtl’ function using a multiple QTL model. The apex positions for both QTL identified on chromosome IV are given in cM above the curves. The calculated 1.5-LOD support interval for the QTL at 12 cM is indicated in red. The blue vertical line indicates the position of the *FRIGIDA* (*FRI*; *AT4G00650*) locus.
